# Targeting PLD3 Reverses the Immunosuppressive Niche by Reprogramming Tumor‐Associated Macrophages and Potentiates Antitumor Immunity

**DOI:** 10.1002/advs.75730

**Published:** 2026-05-21

**Authors:** Xingtu Qin, Qiong Li, Xuemei Xu, Yuhan Wu, Yin Zhu, Jieqiong Wu, Yuan Fang, Qiubei Chen, Shuyang Wang, Jiang Yu, Yaping Ye, Hongli Jiao

**Affiliations:** ^1^ Department of Pathology Nanfang Hospital Southern Medical University Guangzhou Guangdong China; ^2^ Department of Pathology, School of Basic Medical Sciences Southern Medical University Guangzhou Guangdong China; ^3^ Department of General Surgery, Nanfang Hospital Southern Medical University Guangzhou Guangdong China; ^4^ Guangdong Province Key Laboratory of Molecular Tumor Pathology Guangzhou Guangdong China; ^5^ Guangdong Provincial Key Laboratory of Precision Medicine for Gastrointestinal Tumor Guangzhou Guangdong China; ^6^ The First School of Clinical Medicine Southern Medical University Guangzhou Guangdong China; ^7^ Department of Pathology Ganzhou People's Hospital Ganzhou Jiangxi China

**Keywords:** ageing, colorectal cancer, immunotherapy, macrophages, molecular targeted therapy

## Abstract

A challenge in treating colorectal cancer (CRC) is the resistance of mismatch repair‐proficient or microsatellite stable (MSS) tumors to immune checkpoint inhibitors (ICIs). Tumor‐associated macrophages (TAMs) are among key drivers of this resistance. Phospholipase D3 (PLD3) is mainly expressed in macrophages, and PLD3+ macrophage (PLD3+Macro) influences immunotherapeutic efficacy and patient prognosis. We sought to define the role of PLD3 in regulating macrophage phenotypes, CRC progression, and immunotherapeutic response, and to identify potential agents target PLD3. Immunofluorescence revealed that the spatial infiltration dynamics of PLD3+Macro dictate immunotherapy resistance in CRC. We generated myeloid‐specific Pld3‐knockout mice. Using single‐cell RNA sequencing (scRNA‐seq) and flow cytometry, we delineated the impact of PLD3 on the tumor microenvironment (TME). RNA sequencing (RNA‐seq) and mass spectrometry were applied to explore the role of PLD3 in macrophages. By integrating molecular docking with tumor models, we assessed the therapeutic potential of targeting macrophage‐specific PLD3 in tumor progression and immunotherapy resistance. Collectively, PLD3 promotes tumor progression and immunotherapy resistance by modulating lysosomal‐AKT‐NF‐κB axis, thereby driving macrophage senescence and anti‐inflammatory phenotype. This is accompanied by dysregulated crosstalk from macrophages to NK and T cells, ultimately forming an immunosuppressive TME. Abrine targets PLD3 in macrophages and effectively enhances immunotherapy response in CRC.

## Graphical abstract

PLD3 activates the lysosomal‐AKT‐NF‐κB axis to drive cellular senescence in macrophages, establishing an immunosuppressive TME by limiting the infiltration of cytotoxic T, NK, and NKT cells, which confers resistance to anti‐PD‐1 therapy. Abrine inhibits PLD3 expression, restoring antitumor immunity and synergizing with anti‐PD‐1 treatment.

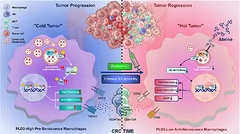

## 1 Introduction

CRC is one of the most prevalent gastrointestinal malignancies [1]. Immunotherapy has emerged as a cornerstone of cancer treatment over the past decade [2], fundamentally transforming the therapeutic landscape. ICIs have revolutionized the management of advanced or metastatic mismatch repair‐deficient (dMMR) or microsatellite instability‐high (MSI‐H) CRC, significantly extending progression‐free survival (PFS) [3].

TAMs as integral components of the TME, are among the key mediators of tumor immune evasion [4]. The diversity of TAMs has started to be clarified by developments in scRNA‐seq [5, 6]. For example, FOLR2+ macrophages within breast cancer stroma acquire T cell‐priming capacity during tumor progression, while intratumoral TREM2+ macrophages are linked to poor prognosis [7]. Similarly, GPNMB‐high macrophages suppress dendritic cell‐mediated activation of CD8^+^ T cells, suggesting that targeting particular TAMs subclusters may represent a promising therapeutic strategy [8]. Therefore, there is an urgent need to develop strategies to reprogram TAMs from immunosuppressive to antitumor phenotypes, thereby mitigating T cell exhaustion and revitalizing antitumor immunity. However, the heterogeneity of TAMs in CRC poses a major challenge for developing targeted therapies.

Analysis of publicly available scRNA‐seq databases revealed that PLD3 is significantly overexpressed in TAMs derived from CRC tissues. The abundance of PLD3+Macro was enriched in patients with poor responses to immunotherapy and unfavorable prognosis, indicating that PLD3 plays a critical role in fostering an immunosuppressive TME and promoting CRC progression.

Through molecular docking, we identified Abrine, a small molecule that suppresses PLD3 expression in macrophages. Previous research has indicated that Abrine inhibits the proliferation of several cancers, including hepatocellular carcinoma, breast cancer, prostate cancer, and non‐small cell lung cancer, while also enhancing the efficacy of anti‐PD‐1 therapy [9, 10, 11]. Nevertheless, the functional role of PLD3 in CRC progression, the mechanisms by which PLD3 facilitates TAMs reprogramming, and the safety profile of Abrine as a macrophage‐targeted PLD3 inhibitor remain to be fully elucidated.

Here, we demonstrate that Abrine suppresses PLD3 expression in macrophages, consequently delaying tumor progression, remodeling the TME, and enhancing the efficacy of anti‐PD‐1 therapy in CRC.

## 2 Results

### 2.1 PLD3+Macro Promotes Immunotherapy Resistance and Poor Prognosis

The treatment landscape for CRC has evolved from conventional chemotherapy to targeted therapy and, most recently, to immunotherapy. However, only a small fraction of patients with the MSI‐H immune‐inflamed subtype benefit from immunotherapy. Consequently, enhancing the sensitivity of immunotherapy‐resistant CRC patients and extending the clinical benefits to a broader patient population have emerged as pressing clinical challenges.

**FIGURE 1:**
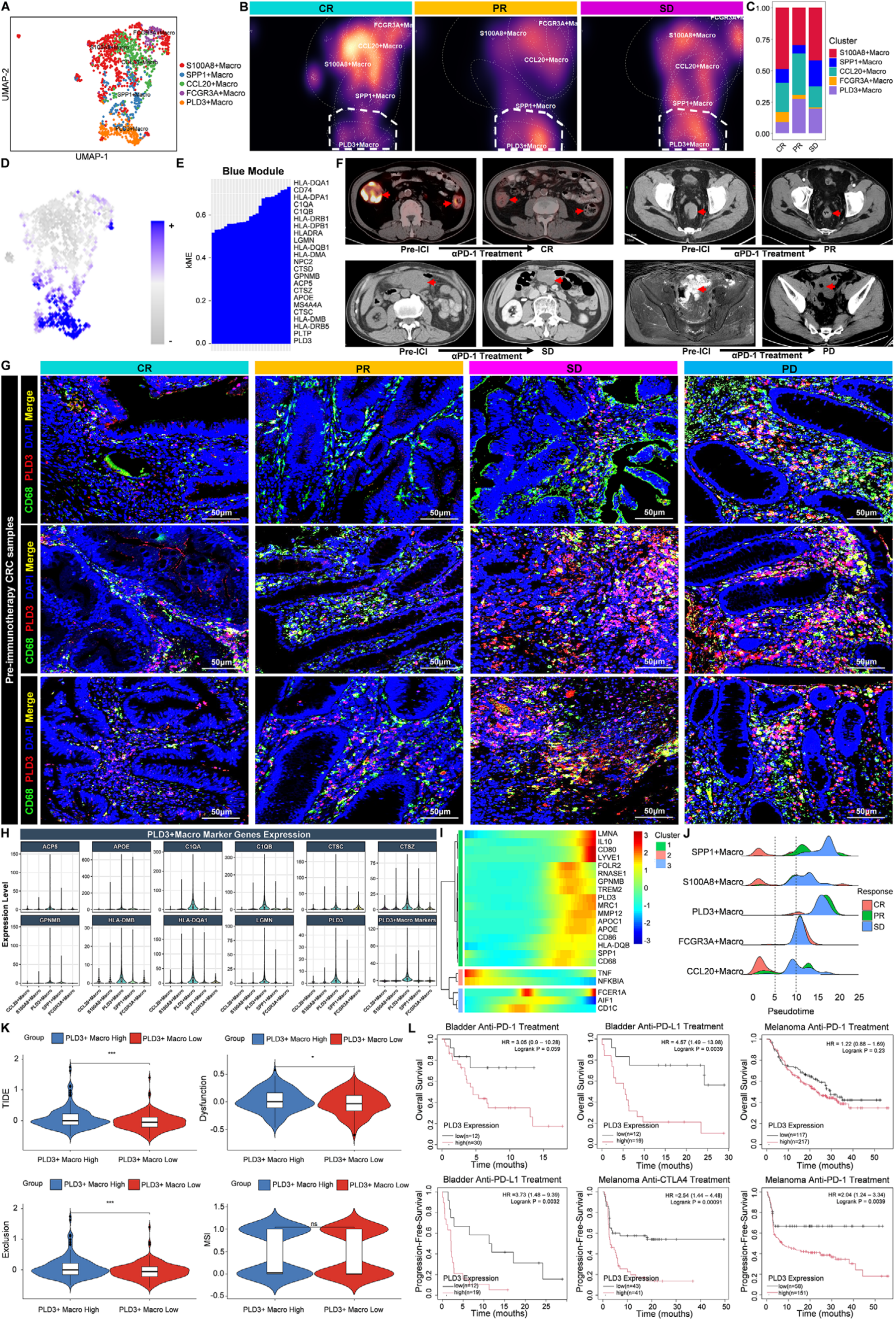
PLD3+Macro mediates immunotherapy resistance in colorectal cancer. (A) Uniform Manifold Approximation and Projection (UMAP) embedding of macrophage subclusters from scRNA‐seq data (GSE236581). (B) Differences in cell densities among CRC patients with different immunotherapy responses (CR versus PR versus SD). CR, Complete Response; PR, Partial Response; SD, Stable Disease. (C) Stack plot displaying the abundance of macrophage subclusters based on different immunotherapy responses. (D,E) HdWGCNA analysis of macrophages to identify the module and core genes associated to PLD3+Macro. (F) Representative radiological (CT) images from CRC patients with differing immunotherapy responses (CR, PR, SD, and PD). PD, Progressive Disease. (G) Representative images of multiplex immunofluorescence for DAPI, PLD3, and CD68 were performed on pre‐ immunotherapy colonoscopic biopsies (*n* = 24) from CRC patients with differing immunotherapy responses (CR, PR, SD, PD). Scale bar, 50 µm. (H) Violin plot comparing the expression of PLD3+Macro markers (ACP5, APOE, C1QA, C1QB, CTSC, CTSZ, GPNMB, HLA‐DMB, HLA‐DQA1, LGMN, and PLD3) in macrophage subclusters. (I) Temporal increase in the expression of tumor‐associated macrophages (TAMs) markers. (J) Temporal analysis of macrophage subclusters. (K) Tumor Immune Dysfunction and Exclusion (TIDE) algorithm predicts the immunotherapy effect of PLD3+Macro High and Low group based on TIDE, Dysfunction, Exclusion, and MSI score. (L) Kaplan‐Meier plots (https://kmplot.com/analysis/) showing the relationships between the level of PLD3 gene expression in tumors and the overall survival or progression‐free survival of patients with cancer treated with anti‐CTLA4, anti‐PD‐1 or anti‐PD‐L1 therapy in a cohort of patients with bladder cancer or melanoma.

Analysis of a single‐cell dataset (GSE236581) from immunotherapy‐treated CRC patients revealed increased myeloid cell infiltration in patients with stable disease (SD) compared to those with complete response (CR) or partial response (PR) (Figure S1A,B). Macrophages, a pivotal component of the myeloid cells, play a crucial role in CRC progression by modulating both innate and adaptive immune responses, with high TAMs density correlating with poor prognosis [12]. In patients with SD, two macrophage subclusters exhibited high infiltration: PLD3+Macro and SPP1+Macro (SPP1+ macrophage) (Figure 1A–C and Figure S1C,D). SPP1 is a well‐established macrophage marker, whereas the function of PLD3 in macrophages remains unclear. HdWGCNA algorithm revealed that genes in the blue module were closely associated with PLD3+Macro, with hub genes including human leukocyte antigens (HLA) and TAMs markers (Figure 1D,E and Figure S1E,F). Immunofluorescence confirmed that PLD3+Macro abundance was higher in patients with SD or Progressive Disease (PD) than in those with CR or PR (Figure 1F,G and Figure S2A and Table S1).

We also identified PLD3+Macro in an independent CRC single‐cell validation dataset (GSE161277) (Figure S3A,B). CIBERSORTx analysis revealed a strong positive correlation between PLD3+Macro infiltration and the abundance of exhausted CD8^+^ T cells (CD8+Tex) (Figure S3C). PLD3+Macro highly expressed TAMs marker genes and anti‐inflammatory markers, including TREM2, APOE, SPP1, C1QC, GPNMB, MS4A4A, and APOC1 [4]. (Figure S1G and S3D).

Additionally, elevated expression of TAM markers was observed in patients with high PLD3+Macro infiltration (Figure S4A). Analysis of the TCGA dataset further showed positive associations between PLD3 expression and TAMs markers (Figure S4B). Similarly, macrophages were classified into PLD3‐expressing (PLD3+Macro) and PLD3−negative (PLD3−Macro) subsets. Differentially expressed genes (DEGs) analysis revealed significant enrichment of TAMs markers and HLA‐related genes in PLD3+Macro compared to PLD3−Macro (Figure S4C,D).

Importantly, the infiltration of PLD3+Macro was enriched in tumor tissues compared to adjacent normal tissues (Figure S3E), with elevated infiltration observed in CMS1 and CMS4 subtypes (Figure S3F). Immunofluorescence indicated PLD3+Macro was more abundant in MSS CRC samples compared to MSI‐H samples (Figure S3G).

Therefore, we aimed to delineate a PLD3+Macro‐associated gene signature consisting of genes specifically enriched in PLD3+Macro relative to other macrophage subclusters, including lysosomal core enzymes (CTSC, CTSZ, ACP5, LGMN), complement system components (C1QA, C1QB), antigen presentation molecules (HLA‐DQA1, HLA‐DMB), APOE, GPNMB, and PLD3. This signature allowed for a more accurate definition of this subcluster (Figure 1H and Figure S1H,I PLD3+Macro Markers.csv).

The CytoTRACE algorithm demonstrated PLD3+Macro had higher differentiation capacity (Figure S1J–K). Intriguingly, antigen presentation‐related genes appeared to be critical for macrophage differentiation (Figure S1L). Pseudotime trajectory analysis revealed a temporal increase in PLD3 expression, accompanied by TAMs markers (Figure 1I and Figure S3H). Both PLD3+Macro and SPP1+Macro were localized to the terminal stages of macrophage differentiation and were predominantly enriched in SD patients (Figure 1J and Figures S1M, S3I,J). Additionally, PLD3+Macro exhibited higher M2‐like polarization scores than other macrophage subclusters (Figure S3K,L).

We further stratified the TCGA CRC cohort into PLD3+Macro high‐ and low‐ infiltration subgroups. The TIDE algorithm showed that the PLD3+Macro high‐infiltration group exhibited elevated TIDE immune scores, Exclusion scores, and Dysfunction scores, with MSI scores remained unchanged (Figure 1K). We extended this investigation to other cancer types receiving immunotherapy using the Kaplan‐Meier Plotter platform. In bladder cancer, high PLD3 expression in patients treated with anti‐PD‐1 showed poorer OS, while in those treated with anti‐PD‐L1, high PLD3 expression was linked to poorer OS and PFS. In melanoma patients treated with anti‐PD‐1 or anti‐CTLA4, high PLD3 expression correlated with worse PFS (Figure 1L).

**FIGURE 2:**
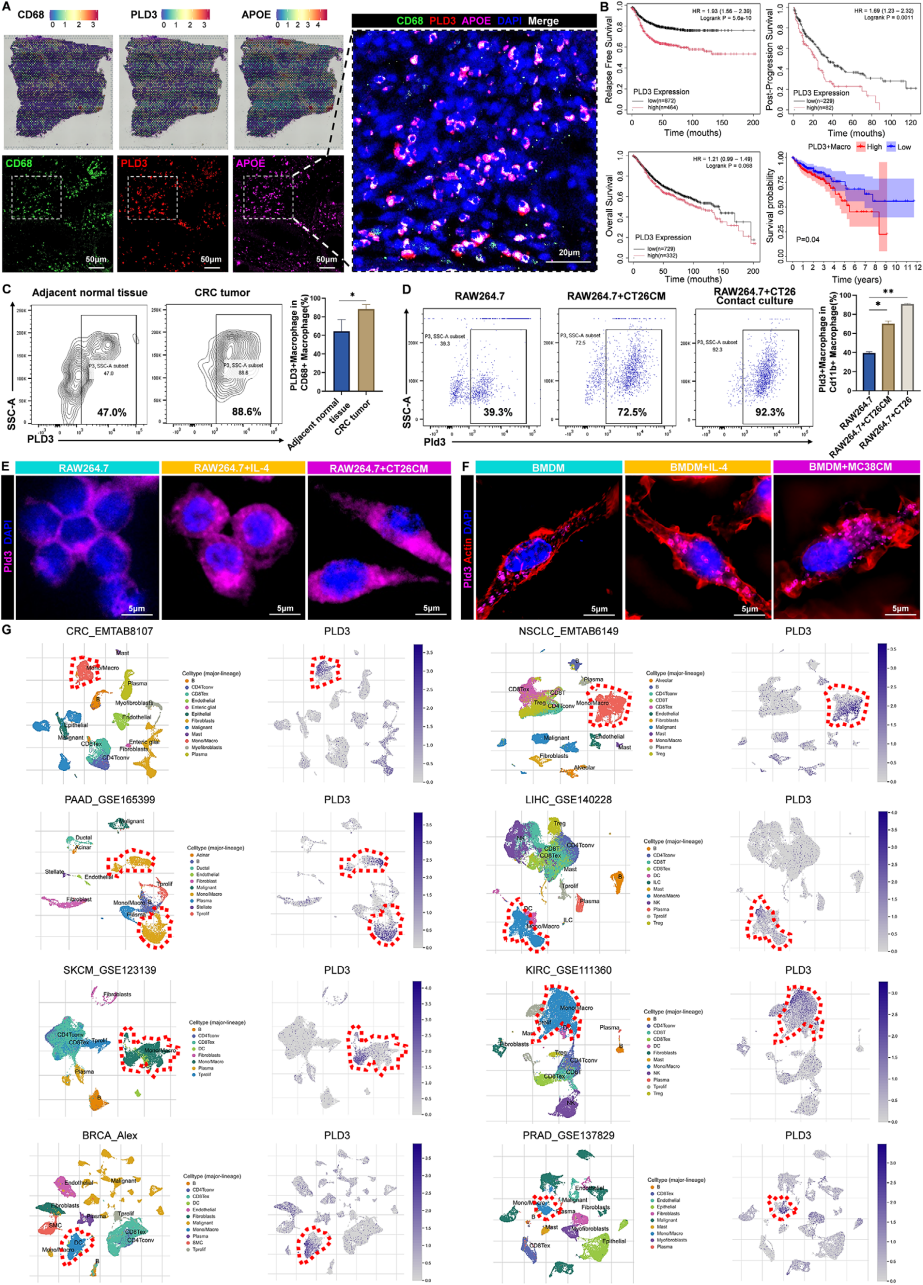
PLD3+Macro predicts poor prognosis in colorectal cancer, and PLD3 exhibits pan‐cancer enrichment in tumor‐associated macrophages. (A) Spatial transcriptomics and immunofluorescence analysis of APOE/PLD3 co‐localization in CD68+macrophages (GSE226997). Scale bar, 50 µm (left) and 20 µm (right). (B) The Kaplan‐Meier Plotter online tool (https://kmplot.com/analysis/) was used to analyse the association between PLD3 mRNA expression levels and Relapse Free Survival, Overall Survival or Post‐Progression Survival, and the KM analysis between PLD3+Macro infiltration high group and low group. (C) Flow cytometry analysis showing the proportion of PLD3+Macro in CRC tumor and adjacent normal tissue (*n* = 3 per group). (D) Flow cytometry analysis showing the proportion of Pld3+Macro in RAW264.7 under different culture conditions (*n* = 3 per group). (E,F) Representative confocal immunofluorescence images of Pld3 in RAW264.7 / BMDM under different culture conditions (*n* = 3 per group). Scale bar, 5 µm. BMDM, bone marrow‐derived macrophages. (G) Analysis of PLD3 gene expression in various tumors using the pan‐cancer single‐cell sequencing data set from the TISCH database (http://tisch.comp‐genomics.org/). Data are means ± SEM, and the P values were calculated by unpaired, two‐tailed Student's t test (C, D). *p < 0.05, **p < 0.01, ***p < 0.001, ****p < 0.0001 and ns, not significant.

Spatial transcriptomics and immunofluorescence showed that PLD3 was primarily localized in tumor stroma, co‐expressing with CD68 and APOE (Figure 2A and Figure S4E). Moreover, patients with high PLD3+Macro infiltration exhibited a poorer prognosis. Elevated PLD3 expression correlated with reduced overall survival (OS), recurrence‐free survival (RFS), and PFS (Figure 2B).

We found PLD3+Macro was significantly increased in CRC tissues relative to adjacent normal tissues (Figure 2C and Figures S4E,F, S9A,B). To investigate tumor‐induced reprogramming of macrophages, we cultured RAW264.7 macrophages in CT26‐conditioned medium or directly co‐cultured with CT26. Flow cytometry analysis revealed an increased proportion of Pld3+Macro within CD11b+ cells, along with elevated Il10 expression (Figure 2D and Figure S4G). Similar findings were obtained in THP‐1‐derived macrophages (Figure S4H). Immunofluorescence revealed IL‐4 stimulation and tumor‐conditioned supernatant upregulated Pld3 expression in macrophages, with predominant cytoplasmic localization (Figure 2E,F and Figure S4I). Furthermore, PLD3 was specifically expressed in TAMs across multiple malignancies (Figure 2G) and was associated with M2 macrophages in various tumors (Figure S4J). Collectively, these findings establish PLD3+Macro as a clinically relevant subset of TAMs that remodel the TME, influence immunotherapy response, and predict adverse prognosis in CRC patients.

### 2.2 PLD3 Deficiency Reverses the Immunosuppressive Tumor Microenvironment and Inhibits Tumor Progression

**FIGURE 3:**
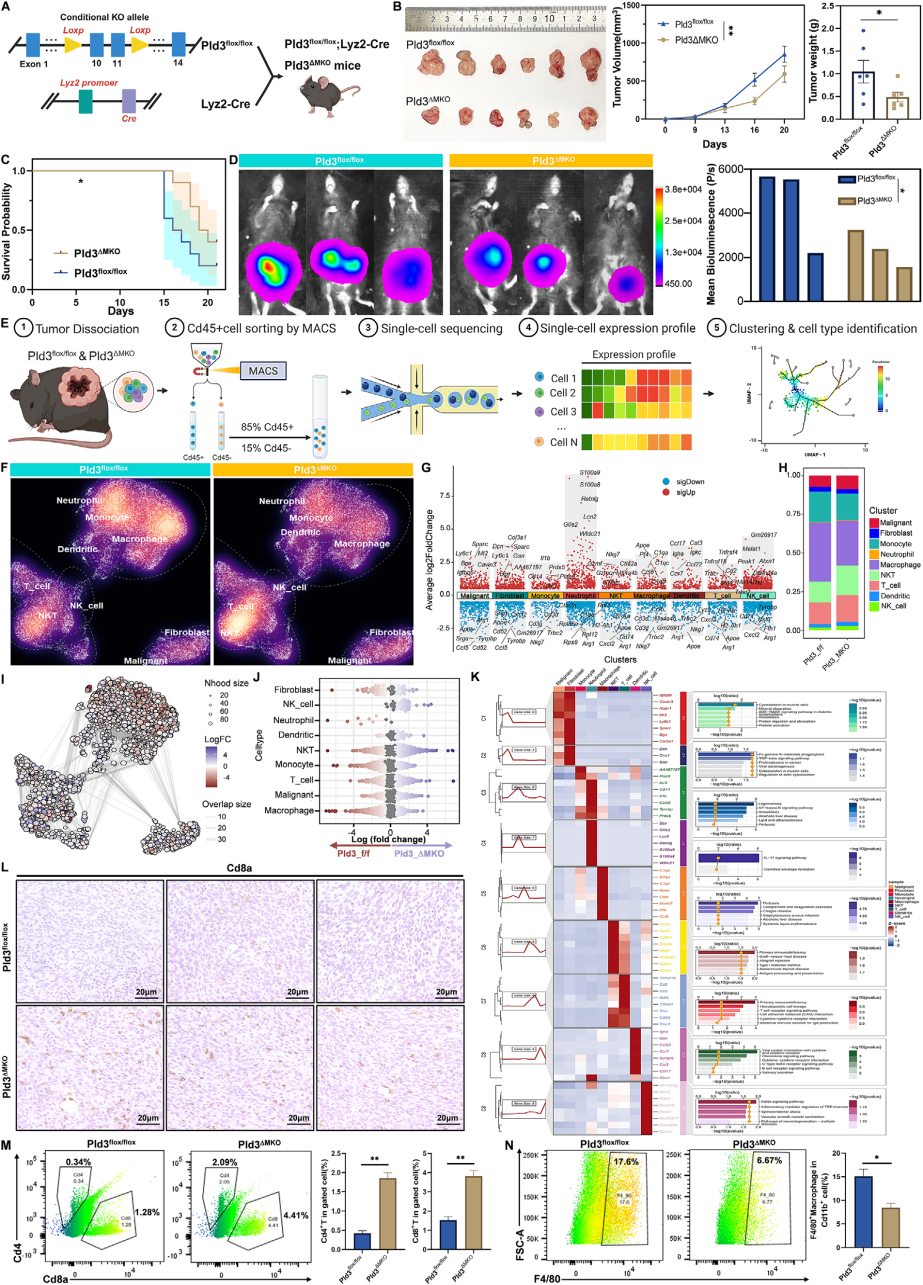
PLD3 deficiency reverses the immunosuppressive tumor microenvironment and inhibits tumor progression. (A) Schematic depicting the gene targeting strategy and crosses to generate myeloid‐specific Pld3 knockout (Pld3^ΔMKO^) mice. (B) MC38 were subcutaneously (sc) injected in Pld3^flox/flox^ and Pld3^ΔMKO^ mice (n = 6 per group) and tumor volume, tumor weight of the mice was shown. MC38 subcutaneous tumor growth was determined by the measurement of tumor volume (cubic millimeter). Data are means ± SEM, and the P values were calculated by two‐way ANOVA (tumor volume) and unpaired, two‐tailed Student's t test (tumor weight). *p < 0.05, **p < 0.01, ***p < 0.001, ****p < 0.0001 and ns, not significant. (C) MC38 were subcutaneously (sc) injected in Pld3^flox/flox^ and Pld3^ΔMKO^ mice (*n* = 10 per group) and overall survival of the mice was shown. (D) MC38‐luc colon cancer cells subcutaneous tumor fragments were inoculated orthotopically in the vascularized area of the cecum of Pld3^flox/flox^ and Pld3^ΔMKO^ mice (*n* = 3 per group). Tumor growth was observed by bioluminescent imaging. (E) Experimental schematics of scRNA‐seq. Briefly, MC38 subcutaneous tumor samples from Pld3^flox/flox^ and Pld3^ΔMKO^ mice (*n* = 2 per group) were dissected, and Cd45+ immune cells were sorted by Magnetic‐Activated Cell Sorting (MACS), and a controlled mixture (85% Cd45+ immune cells and 15% Cd45− non‐immune cells) was subjected to scRNA‐seq. (F) Differences in cell densities of subcutaneous tumors between Pld3^flox/flox^ and Pld3^ΔMKO^ mice. (G) The manhattan plot illustrates the differentially expressed genes among the 9 celltypes. (H) Stack plot displaying the abundance of subclusters. (I) Graph representation of Nhoods identified by MiloR in stromal cells. Nodes are Nhoods, coloured by their log2FC between Pld3^flox/flox^ (red) and Pld3^ΔMKO^ (blue). Non‐differentially abundant neighbourhoods (false discovery rate≥0.1) are coloured white, and sizes correspond to the number of cells in a neighbourhood. The graph edges depict the number of cells shared between adjacent Nhoods. (J) Beeswarm plot showing the distribution of adjusted log2 fold change (log2FC) in abundance between Pld3^flox/flox^ (red) and Pld3^ΔMKO^ (blue) in Nhoods according to cell type. (K) Heatmap exhibiting the KEGG pathways enriched in all cell clusters. (L) Representative images of immunohistochemical staining for Cd8a in subcutaneous tumors from Pld3^flox/flox^ and Pld3^ΔMKO^ mice (*n* = 3 per group). Scale bar, 20 µm. (M) Flow cytometry analysis showing the proportion of CD8^+^ T and CD4^+^ T cells in subcutaneous tumor samples from Pld3^flox/flox^ and Pld3^ΔMKO^ mice (*n* = 3 per group). (N) Flow cytometry analysis showing the proportion of macrophages in subcutaneous tumor samples from Pld3^flox/flox^ and Pld3^ΔMKO^ mice (*n* = 3 per group). Data are means ± SEM, and the P values were calculated by log‐rank (Mantel‐Cox) test (C) and unpaired, two‐tailed Student's t test (D, M,N). *p < 0.05, **p < 0.01, ***p < 0.001, ****p < 0.0001 and ns, not significant.

To clarify the phenotype of PLD3 in macrophages within CRC, we generated myeloid‐specific PLD3 knockout mice (Pld3^ΔMKO^) (Figure 3A and Figures S5A–E, S9C). In subcutaneous tumor models, Pld3^ΔMKO^ mice exhibited suppressed tumor growth (Figure 3B). Additionally, Pld3^ΔMKO^ mice exhibited extended survival compared to controls (Figure 3C). Consistently, this phenotype was replicated in an MC38 orthotopic cecal engraftment model (Figure 3D and Figure S5F). To evaluate the impact of PLD3 on the TME, we performed scRNA‐seq on single cells isolated from MC38 subcutaneous tumors of Pld3^fl/fl^ and Pld3^ΔMKO^ mice (Figure 3E). Following quality control and dimensionality reduction clustering, nine distinct clusters were annotated: Malignant (Satb2, Ankrd1, Mt2), Fibroblast (Dcn, Sparc, Col1a2), Dendritic (Cst3, Ccr7, Syngr2), Macrophage (Apoe, Trem2, C1qc), Monocyte (Il1b, Cd14, H2‐Ab1), Neutrophil (Retnlg, Lcn2, Csf3r), NK cell (Klrb1c, Ncam1, Malat1), T cell (Cd3d, Cd3g, Cd3e) and NKT (Nkg7, Cd3g, Cd8a) (Figure 3F,G and Figure S5G–J).

Notably, MiloR differential abundance analysis revealed reductions in macrophages and monocytes, alongside increases in NKT, T, and NK cells in Pld3^ΔMKO^ tumors (Figure 3H–J). Furthermore, pathway analysis demonstrated enhanced activation of antigen presentation and tumor‐killing effector pathways in NKT and T cells (Figure 3K and Figure S5K), suggesting that myeloid Pld3 knockout facilitates immune cell infiltration within the TME. Consistently, immunohistochemical staining confirmed a higher density of CD8^+^ T cells within the tumors of Pld3^ΔMKO^ mice (Figure 3L). Flow cytometry demonstrated that subcutaneous tumors from Pld3^ΔMKO^ mice exhibited increased infiltration of CD8^+^ and CD4^+^ T cells and reduced macrophage infiltration compared to Pld3^fl/fl^ mice (Figure 3M,N). Consistently, we established a Pld3‐knockdown RAW264.7 cell line (RAW264.7‐shPld3) (Figure S6A–D) and established a subcutaneous tumor model by co‐implanting CT26 and RAW264.7 cells. Flow cytometry analysis suggested that macrophages suppressed CD8^+^ T cell infiltration, which was reversed by PLD3 knockdown in macrophages (Figure S6E).

Additionally, to exclude the possibility that Pld3 deletion in neutrophils confounded the phenotypes observed in myeloid‐specific Pld3‐knockout mice, we profiled Pld3 expression in macrophages and neutrophils using flow cytometry, immunofluorescence, spatial transcriptomics, and scRNA‐seq. Notably, Pld3 expression was robustly detected in macrophages, whereas neutrophils showed little to no expression (Figure S7A–D and S8A–D).

Collectively, our findings define a critical role for macrophage‐derived PLD3 in forming immunosuppressive niches.

### 2.3 PLD3 Deficiency Activates T Cells Antitumor Immunity to Reverse the Immunosuppressive Tumor Microenvironment

**FIGURE 4:**
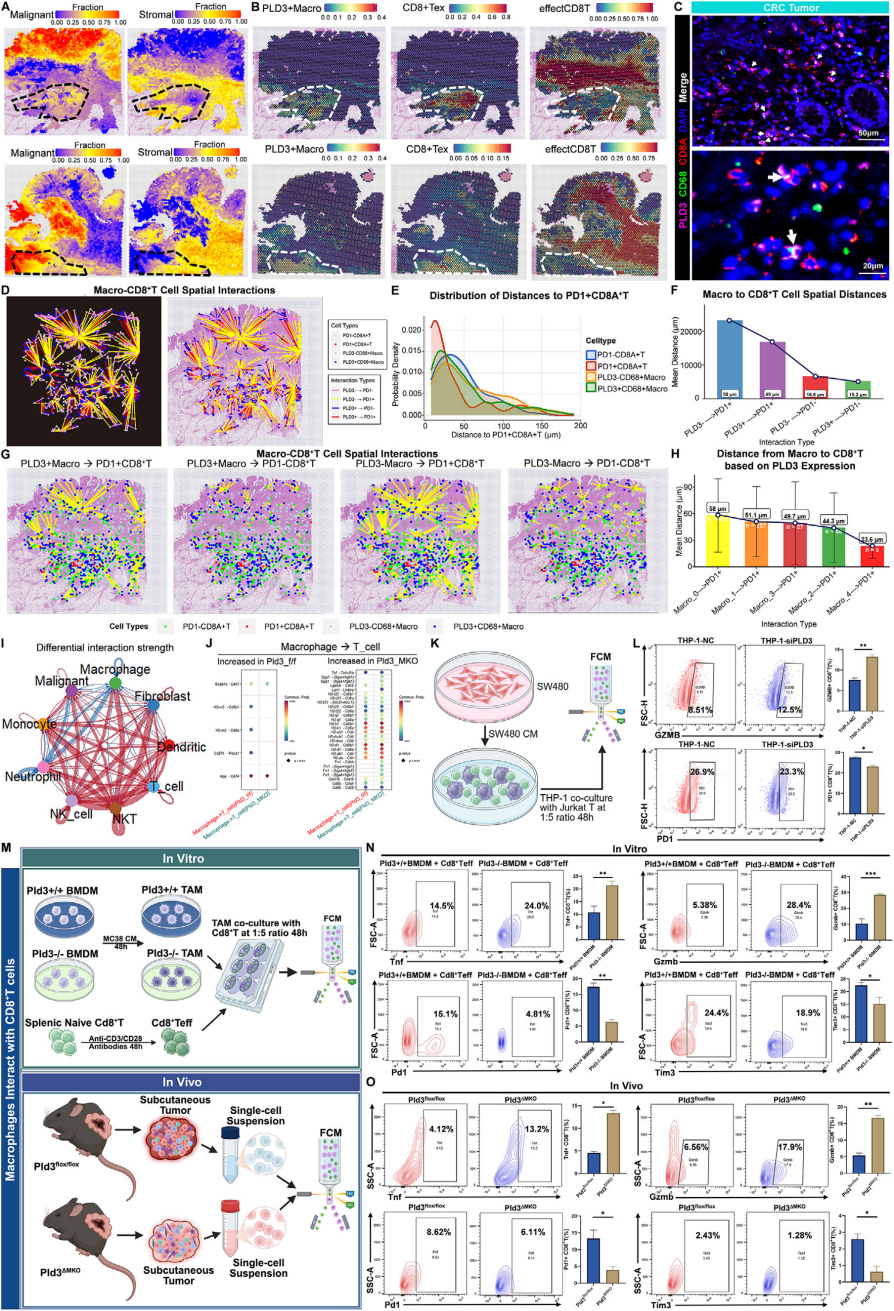
PLD3 deficiency activates antitumor immunity in T cells to reverse the immunosuppressive tumor microenvironment. (A) Spatial sections were segmented into tumor parenchyma and stroma using SpaCET algorithm (GSE226997). (B) Spatial transcriptomics analysis of the distribution of PLD3+Macro, CD8+Tex (exhausted CD8^+^ T) and effectCD8T (effective CD8^+^ T) (GSE161277, GSE226997). (C) Representative multiplex immunofluorescence images of DAPI, PLD3, CD68, and CD8A in the CRC samples (*n* = 3). Scale bar, 50 µm (top) and 20 µm (bottom). (D) Representative spatial transcriptomic plots showing the distribution of PLD3+CD68+ macrophages, PLD3‐CD68+ macrophages, PD1+CD8A+ T cells, and PD1‐CD8A+ T cells, with lines connecting each macrophage to its nearest CD8^+^ T cell (combined). (E) Density distribution plots of the nearest spatial distances to PD1+CD8A+ T cells from four cell phenotypes: PLD3+CD68+ macrophages, PLD3‐CD68+ macrophages, PD1+CD8A+ T cells, and PD1‐CD8A+ T cells. (F) Boxplots comparing the spatial distances from PLD3+CD68+ macrophages and PLD3‐CD68+ macrophages to PD1+CD8A+ T cells and PD1‐CD8A+ T cells, showing that PLD3+CD68+ macrophages were significantly closer to PD1+CD8A+ T cells and PD1‐CD8A+ T cells than PLD3‐CD68+ macrophages. (G) Representative spatial transcriptomic plots showing the distribution of PLD3+CD68+ macrophages, PLD3‐CD68+ macrophages, PD1+CD8A+ T cells, and PD1‐CD8A+ T cells, with lines connecting each macrophage to its nearest T cell (separately). (H) Quantitative analyses of spatial distances between macrophages and CD8^+^ T cells, binned by PLD3 expression levels in macrophages. (I) Strength of inferred interactions, comparing Pld3^flox/flox^ and Pld3^ΔMKO^. (J) Dot plot showing the cell‐cell interactions of macrophages with T cell, comparing Pld3^flox/flox^ and Pld3^ΔMKO^. (K) THP‐1 derived macrophages and CD8^+^ T cells co‐culture experimental design. (L) Flow cytometry analysis showing the proportion of GZMB^+^ and PD1^+^ CD8^+^ T cells, which were co‐cultured with THP‐1‐NC or THP‐1‐siPLD3 (*n* = 3 per group). (M) Experimental design to assess the role of macrophage‐intrinsic Pld3 in regulating CD8^+^ T cell activation in vitro and in vivo. (N) Flow cytometry analysis showing the proportion of Gzmb^+^, Tnf^+^, Tim3^+^, and Pd1^+^ Cd8^+^ T cells, which were co‐cultured with Pld3+/+ BMDM and Pld3‐/‐ BMDM in vitro (n = 3 per group). (O) Flow cytometry analysis showing the proportion of Gzmb^+^, Tnf^+^, Tim3^+,^ and Pd1^+^ Cd8^+^ T cells in subcutaneous tumor samples from Pld3^flox/flox^ and Pld3^ΔMKO^ mice (*n* = 3 per group). Data are means ± SEM, and the P values were calculated by unpaired, two‐tailed Student's t test (L, N,O). *p < 0.05, **p < 0.01, ***p < 0.001, ****p < 0.0001 and ns, not significant.

To further investigate the impact of PLD3 deficiency on the immune cells, we analyzed the spatial distribution of cell subclusters using spatial transcriptomics datasets. Interestingly, spatial transcriptomic analysis revealed co‐localization of PLD3+Macro with exhausted CD8^+^ T (CD8+Tex) and spatial complementarity between PLD3+Macro and effective CD8^+^ T (effectCD8T) (Figure 4A,B). Immunofluorescence analysis confirmed direct contacts between PLD3+CD68+ macrophages and CD8^+^ T cells (Figure 4C), suggesting PLD3+Macro may regulate effector function of CD8^+^ T cells.

Intriguingly, PLD3+CD68+ macrophages were consistently located in closer spatial proximity to PD1+CD8A+ T cells than PLD3−CD68+ macrophages (Figure 4D–G). Quantitative distance analysis demonstrated that the spatial distance between CD68+ macrophages and PD1+CD8A+ T cells decreased progressively with increasing PLD3 expression levels (Figure 4H and Figure S11A) [13].

Additionally, the NicheNet algorithm demonstrated PLD3+Macro interacts with CD8+Tex through IGF1‐RPL, CCL2‐TGFB1, and APOE‐VCAM1 axes (Figure S11B,C). Moreover, in subcutaneous tumors derived from Pld3^ΔMKO^ mice, cell communication analysis revealed enhanced interactions between cell subclusters (Figure 4I and Figure S11D–G), with increased interaction from macrophages to NKT and T cells (Figure 4J and Figure S11H). These findings indicate that PLD3 knockout in macrophages restructures cellular crosstalk within the TME, potentially facilitating improved tumor cell recognition and elimination.

Consistently, in vitro, PLD3 knockdown in THP‐1‐derived macrophages co‐cultured with Jurkat T cells promoted GZMB expression in T cells, while PD1 expression was downregulated (Figure 4K,L and Figure S11I). In agreement with our findings in human macrophages, we co‐cultured murine macrophages with splenic Cd8^+^ T cells and found that Pld3 knockdown in macrophages enhanced effector function (Gzmb, Tnf, Ifng, Perforin) and reduced exhausted phenotype (Pd1, Tim3) in CD8^+^ T cells in vitro (Figure 4M,N and Figures S9D, S11J–L). In line with these in vitro findings, in vivo analysis of Cd8^+^ T cells within MC38 subcutaneous tumors recapitulated the same phenotype (Figure 4M,O).

These results suggested that Pld3 deletion facilitates the recruitment of cytotoxic immune cells, including T, NKT, and NK cells, thereby impeding tumor progression.

### 2.4 PLD3‐Deficient Macrophage Reduces the Anti‐Inflammatory Phenotype of Tumor‐Associated Macrophages

**FIGURE 5:**
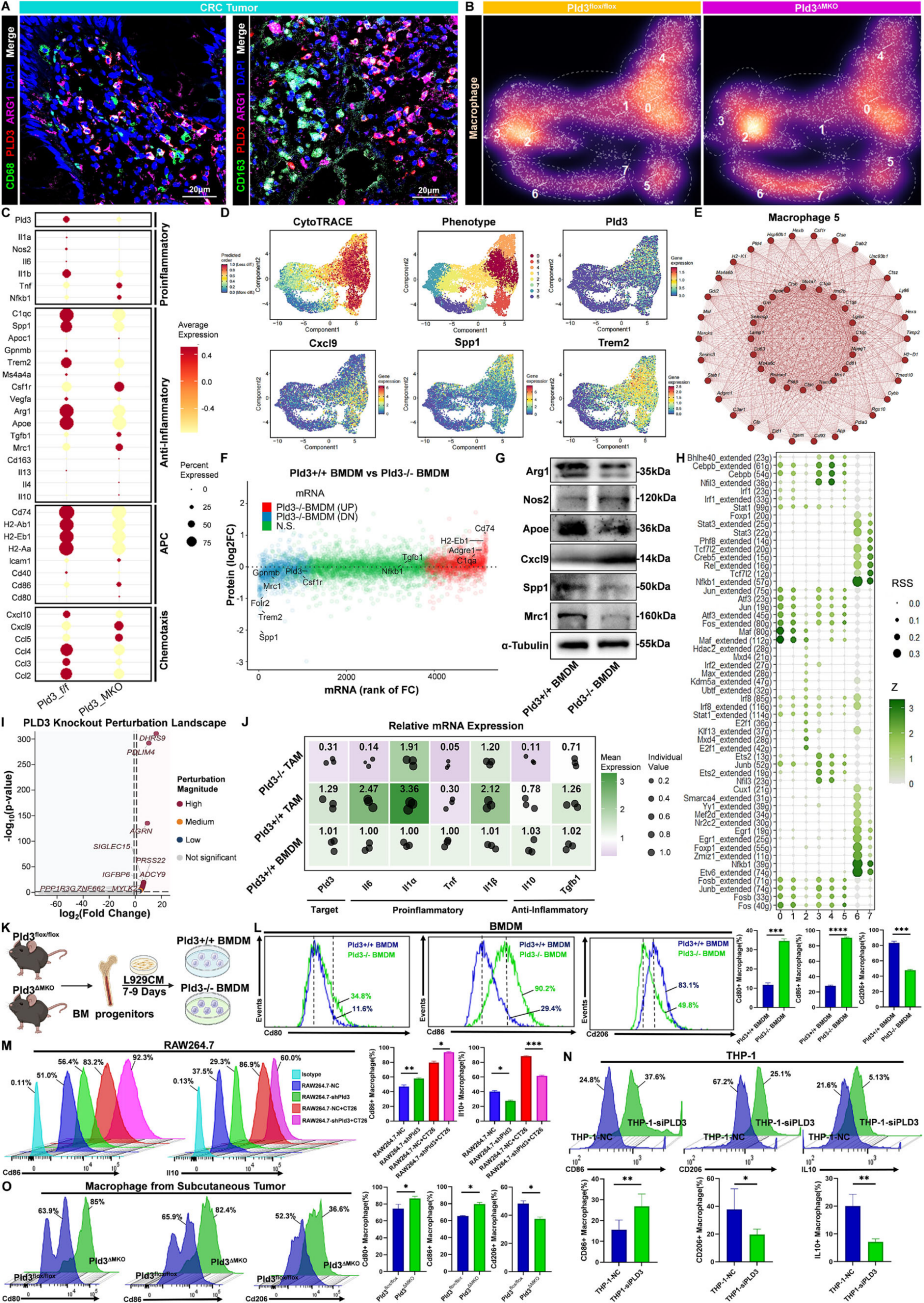
PLD3‐deficient macrophage reduces the anti‐inflammatory phenotype of tumor‐associated macrophages. (A) Representative multiplex immunofluorescence images of DAPI, PLD3, ARG1, and CD163/CD68 in the CRC samples (*n* = 2). Scale bar, 20 µm. (B) Differences in macrophage subcluster densities of subcutaneous tumors between Pld3^flox/flox^ and Pld3^ΔMKO^ mice. (C) Dot plot showing the RNA expression of marker genes in macrophages of subcutaneous tumors, used to define the function of Proinflammatory, Anti‐inflammatory, APC, and Chemotaxis. (D) Cell trajectories of macrophages subclusters inferred by CytoTRACE. (E) HdWGCNA analysis of macrophages to identify the module and core genes associated to Pld3^flox/flox^ (Macrophage 5). (F) Comparison of change in protein abundance (y‐axis) with change in mRNA expression (x‐axis) following the differentially expressed genes (DEGs) from Pld3+/+ BMDM and Pld3‐/‐ BMDM. (G) Protein expressions of Arg1, Nos2, Apoe, Cxcl9, Spp1, and Mrc1 in Pld3+/+ BMDM and Pld3‐/‐ BMDM detected by western blotting. (H) Regulon Specificity Score (RSS) analysis quantifying the specificity of key transcriptional regulators across macrophage subclusters. (I) scTenifoldKnk algorithm revealed that PLD3 deletion led to pronounced dysregulation of previously identified polarization‐related genes, including AGRN, PDLIM4, MYLK2, ADCY9, and SIGLEC15 (GSE178341). (J) Heatmap showing the differential expression of proinflammatory and anti‐Inflammatory markers detected by RT‐qPCR in Pld3+/+ BMDM, Pld3+/+ TAM, and Pld3‐/‐ TAM (*n* = 3 per group). BMDMs were stimulated with MC38 conditioned medium (MC38‐CM) to induce TAMs for 24 h. (K) Schematic of BMDM isolation. (L) Flow cytometry analysis showing the proportion of Cd80 (APC), Cd86 (APC), and Cd206 (M2 marker) expression in Pld3+/+ BMDM and Pld3‐/‐ BMDM (*n* = 3 per group). (M) Flow cytometry analysis showing the proportion of Cd86 (APC) and Il10 (anti‐inflammatory) expression in RAW264.7‐NC or RAW264.7‐shPld3 under different culture conditions (*n* = 3 per group). RAW264.7 cells were co‐cultured with CT26 at 2:1 ratio. (N) Flow cytometry analysis showing the proportion of CD86 (APC), CD206 (M2 marker), and Il10 (anti‐inflammatory) expression in THP‐1‐NC and THP‐1‐siPLD3 (*n* = 3 per group). (O) Flow cytometry analysis showing the proportion of Cd80 (APC), Cd86 (APC), and Cd206 (M2 marker) expression of macrophages in subcutaneous tumor samples from Pld3^flox/flox^ and Pld3^ΔMKO^ mice (*n* = 3 per group). Data are means ± SEM, and the P values were calculated by unpaired, two‐tailed Student's *t* test (L‐O). *p < 0.05, **p < 0.01, ***p < 0.001, ****p < 0.0001 and ns, not significant. [Correction added on 1 June 2026, after first online publication: Figure 5 has been updated in this version.]

To investigate whether PLD3 regulates macrophage anti‐inflammatory polarization and tumor‐promoting functions, we first verified PLD3 co‐localizes with ARG1 and CD163 in macrophages using immunofluorescence (Figure 5A and Figure S13A,B). Subsequently, we analyzed TAMs of scRNA‐seq from subcutaneous tumors. Utilizing dimensionality reduction clustering, we identified eight clusters (0–7), with cluster 2, 6, and 7 enriched in Pld3^ΔMKO^ mice, while clusters 0, 1, 3, 4, and 5 were enriched in Pld3^fl/fl^ mice (Figure 5B and Figure S12A,B). Clusters 0, 1, 4, and 5 exhibited enhanced anti‐inflammatory markers, antigen presentation, and chemokine secretion, whereas clusters 2, 3, 6, and 7 displayed elevated pro‐inflammatory signatures (Figure S12D). Essentially, macrophages from Pld3^ΔMKO^ demonstrated reduced anti‐inflammatory and augmented pro‐inflammatory phenotypes (Figure 5C). Macrophages from Pld3^ΔMKO^ mice demonstrated lower differentiation capacity compared to Pld3^fl/fl^ mice, whereas the latter displayed elevated expression of TAMs markers (Trem2, Spp1, Ms4a4a, C1qc, Apoe, Gpnmb) and Pld3, showing a complementary relationship between Spp1‐high and Cxcl9‐high subclusters (Figure 5D and Figure S12C,E). CXCL9:SPP1 polarity could define macrophage polarity, suggesting Pld3 could be a key driver in macrophage anti‐inflammatory conversion [14].

Using hdWGCNA, we analyzed eight macrophage module genes. The blue (Macrophage1) and brown (Macrophage5) modules were enriched in Pld3^fl/fl^ mice, with the blue module (Macrophage1) enriched for ribosomal translation genes (RPS, RPL) and the brown module (Macrophage5) encoding canonical TAMs markers (Apoe, Trem2, Mrc1, C1qc, Csf1r), indicating that Pld3 promotes the anti‐inflammatory programming of TAMs (Figure 5E and Figure S12F–H).

Moreover, SCENIC transcription factor analysis suggested that E2f1, Nfkb1, and Etv6 were more active in Pld3^ΔMKO^ mice, while Maf, Myc, Nfil3, and E2f2 played more important roles in Pld3^fl/fl^ mice (Figure 5H and Figure S12I). Using scTenifoldKnk algorithm, we identified that deletion of PLD3 led to pronounced dysregulation of the polarization‐related genes previously identified, including AGRN, PDLIM4, MYLK2, ADCY9, and SIGLEC15 (Figure 5I).

To further elucidate the role of Pld3 in macrophages, we isolated bone marrow‐derived macrophage (BMDM) from Pld3^fl/fl^ (Pld3+/+BMDM) and Pld3^ΔMKO^ mice (Pld3‐/‐BMDM) for RNA‐seq and quantitative proteomics (4D‐DIA). Integrated analysis revealed strong concordance between gene and protein expression, with downregulation of TAMs markers (Spp1, Trem2, Folr2, Mrc1, Gpnmb, and Csf1r) in Pld3‐/‐ BMDM (Figure 5F). IL‐4 stimulation enhanced Pld3 expression in macrophages (Figure S13C). Pld3 knockout downregulated anti‐inflammatory markers (Mrc1, Spp1, Apoe, and Arg1) while upregulating pro‐inflammatory mediators (Nos2 and Cxcl9) in macrophages (Figure 5G and S6F and Figure S13D). RT‐qPCR analysis demonstrated macrophages exposed to tumor supernatant increased expression of selected inflammatory and immunosuppressive markers, but Pld3 deletion reversed this trend (Figure 5J).

Consistently, in vitro flow cytometry analysis of murine and human macrophages confirmed that Pld3 knockdown increased Cd86 and Cd80, while decreasing Il10 and Cd206 (Figure 5K–N and Figure S9E,F). Similarly, in vivo experiments using MC38 subcutaneous tumors in mice revealed the same trend (Figure 5O).

To preliminarily investigate the role of PLD3 in immunosuppression, we performed co‐immunoprecipitation coupled with mass spectrometry (Co‐IP/MS) to screen potential protein‐binding partners in macrophages (Figure S13E, CoIP‐MS.csv). Strikingly, the results revealed that PLD3 interacts with TAMs markers, including Csf1r, C1qb, Gpnmb, Apoc1, Folr2, Mrc1, and Arg1. Co‐IP validated Pld3‐Arg1 interaction in macrophages (Figure S13F). Immunofluorescence confirmed the co‐localization of PLD3 with ARG1 (Figure S13A). Moreover, AlphaFold3 corroborated these findings (Figure S13G), suggesting that PLD3 may interact with TAMs markers and regulate macrophage polarization toward an immunosuppressive phenotype.

### 2.5 PLD3 Exerts Pro‐Tumorigenic Immunosuppressive Capacity by Orchestrating the Lysosomal‐AKT‐NF‐κB Axis

**FIGURE 6:**
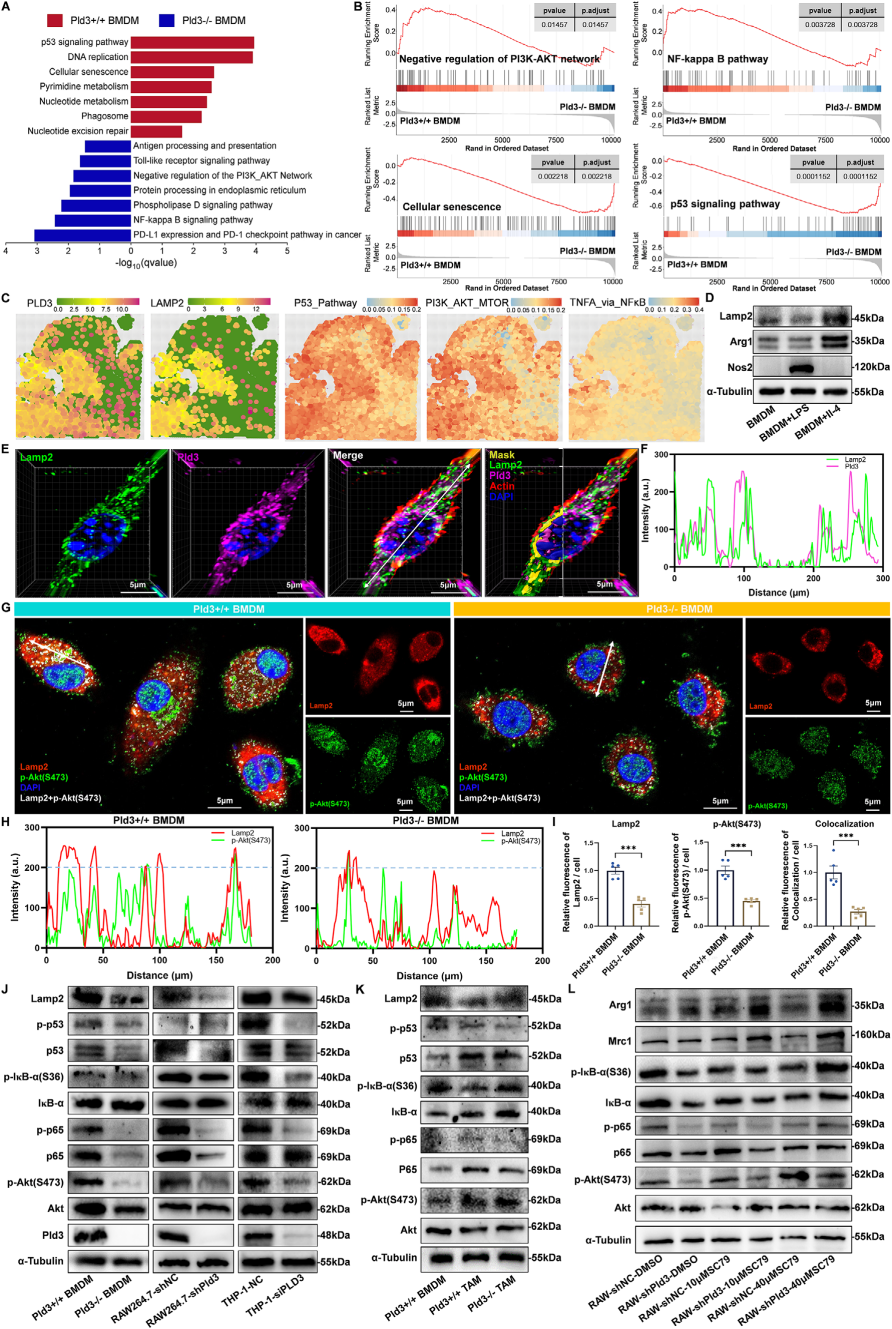
PLD3 exerts pro‐tumorigenic immunosuppressive capacity by orchestrating the lysosomal‐AKT‐NF‐κB axis. (A,B) The Gene set enrichment analysis (GSEA) indicated that PLD3 was associated with the PI3K‐AKT, NF‐kappa B, p53, and Cellular senescence signaling pathway. (C) Spatial colocalization of PLD3 and LAMP2 correlates with activation of the P53 Pathway, PI3K_AKT_mTOR Pathway, and TNFA_via_NFκB pathways (GSE226997). (D) IL‐4 (40 ng/mL) was used to stimulate M0 macrophages to polarise into the M2‐phenotype for 48 h, and LPS (100 ng/mL) was used to stimulate M0 macrophages to polarise into the M1‐phenotype for 24 h. Western blotting detects the expression of Lamp2, M2 markers (Arg1), and M1 markers (Nos2) in BMDM. (E,F) Representative confocal immunofluorescence images and statistical analysis of Pld3, Lamp2, and their co‐localization in BMDM. Scale bar, 5 µm. Histograms showing the normalized fluorescence intensity of Pld3 and Lamp2 were obtained from cross‐sectional lines through the cell cytoplasm (F). (G–I) Representative confocal immunofluorescence images and statistical analysis of Lamp2, p‐Akt(S473), and their co‐localization, comparing Pld3+/+ BMDM and Pld3‐/‐ BMDM (*n* = 5 per group). Scale bar, 5 µm. Histograms showing the normalized fluorescence intensity of Lamp2 and p‐Akt (S473) were obtained from cross‐sectional lines through the cell cytoplasm (H). Data are means ± SEM, and the P values were calculated by unpaired, two‐tailed Student's t test (I). *p < 0.05, **p < 0.01, ***p < 0.001, ****p < 0.0001 and ns, not significant. (J) Western blotting detects the expression of Pld3, Lamp2, Akt, p‐Akt (Ser473), p65, p‐p65, Ikb‐α, p‐Ikb‐α(S36), p53 and p‐p53 of macrophages with different PLD3 expression levels of BMDM, RAW264.7 and THP‐1 cells. (K) Western blotting detects the expression of Lamp2, Akt, p‐Akt (Ser473), p65, p‐p65, Ikb‐α, p‐Ikb‐α(S36), p53 and p‐p53 of BMDM and TAMs. BMDMs were stimulated with MC38 conditioned medium to induce TAMs for 24 h. (L) RAW264.7‐shNC and RAW264.7‐shPld3 were treated with or without the AKT activator SC79 (10 µm or 40 µm) for 24 h. Western blotting detects the expression of Akt, p‐Akt (Ser473), p65, p‐p65, Ikb‐α, p‐Ikb‐α(S36), Mrc1, and Arg1.

To elucidate the molecular mechanism by which PLD3 orchestrates anti‐inflammatory programming in macrophages, we performed pathway enrichment analysis on DEGs from RNA‐seq of Pld3+/+BMDM and Pld3‐/‐BMDM, as well as macrophages isolated from scRNA‐seq of Pld3^fl/fl^ and Pld3^ΔMKO^ subcutaneous tumors. The analysis revealed Pld3 was enriched in p53 signaling, cellular senescence, lysosome, and nucleotide metabolism pathways, while Pld3‐deficient macrophages showed activation of NF‐κB signaling, negative regulation of PI3K‐AKT, and Toll‐like receptor signaling (Figure 6A–C and Figure S14A, C–E). HdWGCNA analysis of macrophages from Pld3^fl/fl^ subcutaneous tumors revealed that the blue module was enriched in ribosomal pathways, whereas the brown module was enriched in lysosomal pathways (Figures S12F–G, S14B).

Previous studies have demonstrated PLD3 is predominantly localized within lysosomes and functions as a bifunctional enzyme, exhibiting exonuclease and lysophospholipase activities [15, 16]. Immunofluorescence confirmed cytoplasmic Pld3 co‐localizes with lysosomal protein Lamp2 (Figure 6E,F and Figure S15A–C and Video S1). Stimulation with IL‐4 promoted M2 macrophage polarization and enhanced Lamp2 expression (Figure 6D), whereas Pld3 knockout suppressed Lamp2 expression (Figure 6G–K). Based on these findings, we propose Pld3 may modulate the anti‐inflammatory phenotype of macrophages through lysosomal regulation.

Lysosomes situated at the cell periphery exhibit a greater capacity for activating the AKT signaling pathway compared to their perinuclear counterparts, with AKT being partially localized to lysosomes [17]. We demonstrated p‐Akt(S473) co‐localizes with Lamp2 in macrophages, and Pld3 knockout suppresses p‐Akt(S473) expression and the colocalization of p‐Akt(S473) and Lamp2 (Figure 6G). We validated PLD3‐dependent regulation of the AKT‐NF‐κB axis in murine and human macrophages, with Pld3 knockout suppressing pathway activation (Figure 6J). Tumor‐conditioned supernatant promoted activation of the AKT‐NF‐κB pathway, whereas Pld3 knockdown attenuated this tumor‐induced signaling (Figure 6K). Subsequently, we stimulated RAW264.7‐shPld3 cells with SC79, and found that it activated the AKT pathway, rescued the impaired AKT‐NF‐κB pathway activation caused by Pld3 knockdown, and enhanced the anti‐inflammatory phenotype of macrophages (Figure 6L).

### 2.6 PLD3 Drives Cellular Senescence to Suppress Antitumor Immunity via the AKT–NF‐κB Axis

**FIGURE 7:**
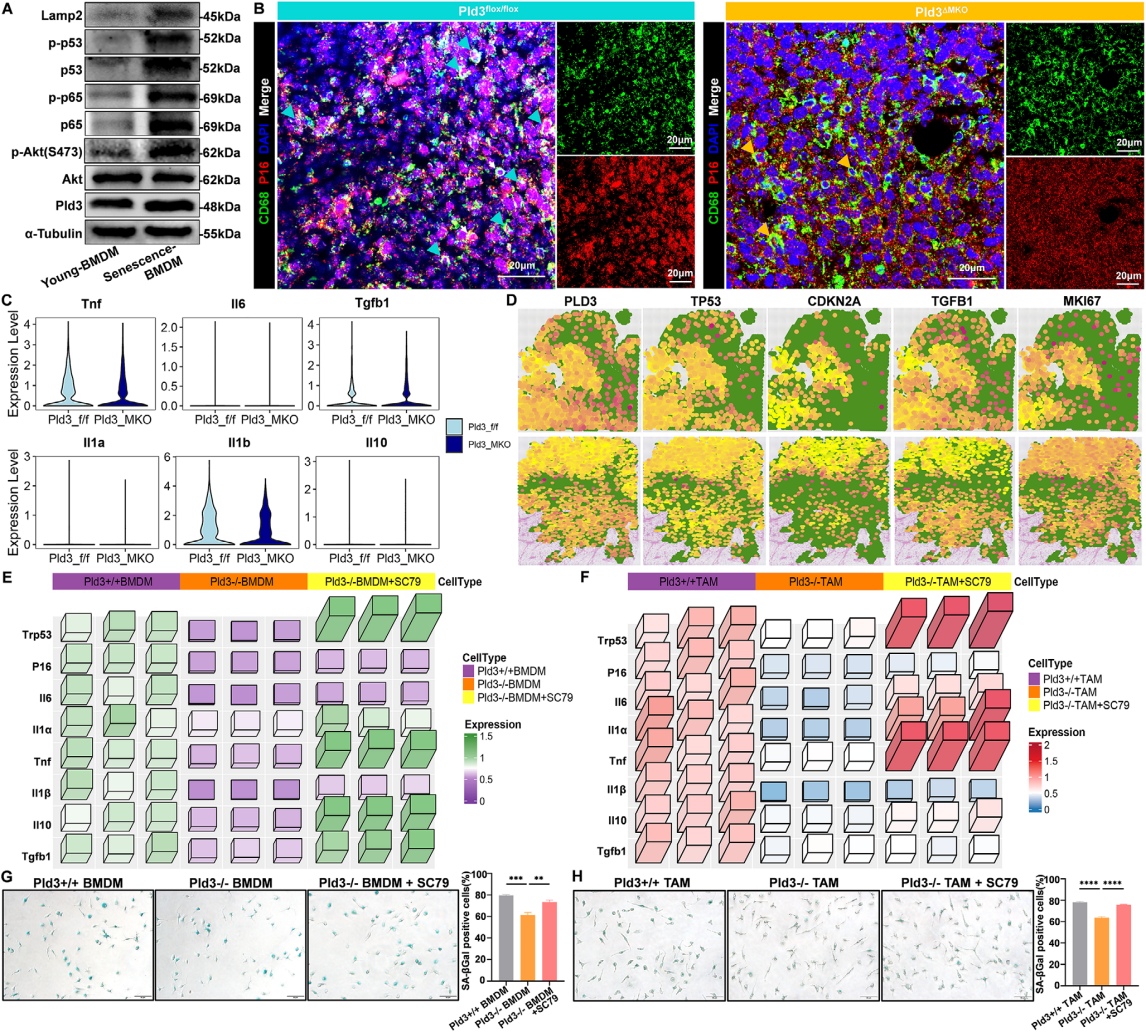
PLD3 drives cellular senescence to suppress antitumor immunity via the AKT–NF‐κB axis. (A) Western blotting detects the expression of Pld3, Lamp2, Akt, p‐Akt (Ser473), p65, p‐p65, p53, and p‐p53 of BMDM from young (12–16 weeks) and senescence (over 60 weeks) mice. (B) Representative multiplex immunofluorescence images of DAPI, P16 (CDKN2A), and CD68 in subcutaneous tumor samples from Pld3^flox/flox^ and Pld3^ΔMKO^ mice (*n* = 3 per group). Scale bar, 20 µm. (C) Violin plot comparing the expression of the senescence‐associated secretory phenotype (SASP) markers of macrophages from Pld3^flox/flox^ and Pld3^ΔMKO^ mice. (D) Spatial transcriptomic analysis revealed a strong spatial co‐localization between PLD3 and senescence‐associated genes (TP53, CDKN2A, TGFB1, MKI67) (GSE226997). (E, F) Heatmap showing the SASP markers of BMDM/TAMs under different culture conditions detected by RT‐qPCR. BMDMs were stimulated with MC38‐CM for 24 h to induce TAMs. BMDM/TAMs were additionally stimulated with AKT activator SC79 (40 µM) for 24 h in BMDM/TAM+SC79 group. (G,H) Senescence‐associated β‐Galactosidase (SA‐β‐Gal) staining detects the senescence phenotype of macrophages under different culture conditions. BMDMs were stimulated with MC38‐CM for 24 h to induce TAMs, and BMDM/TAM were additionally stimulated with AKT activator SC79 (40 µM) for 24 h in BMDM/TAM+SC79 group (*n* = 3 per group). Senescent cell numbers of macrophages by SA‐β‐Gal staining under different culture conditions are listed.

Importantly, macrophages derived from aged mice exhibited heightened activation of the AKT‐NF‐κB and senescence‐associated p53 pathways, along with elevated expression of Lamp2 and Pld3, compared to young mice (Figure 7A and Figure S15D). Macrophages from Pld3^ΔMKO^ mice exhibited a reduced senescent phenotype compared with those from Pld3^fl/fl^ mice (Figure 7B,C). Spatial transcriptomic analysis revealed strong spatial co‐localization between PLD3 and senescence‐associated genes (Figure 7D). Furthermore, tumor supernatant induced a senescent phenotype in macrophages, whereas Pld3 knockout inhibited p53 pathway activation and mitigated tumor supernatant‐induced senescence (Figure S15E).

Further validation through RT‐qPCR and SA‐β‐Gal staining confirmed SC79 restored the diminished senescence‐associated secretory phenotype (SASP) resulting from Pld3 knockout in macrophages (Figure 7E–H).

These findings indicate that PLD3 deficiency alters lysosomal dynamics, thereby suppressing macrophage senescence and enhancing antitumor immunity through the AKT‐NF‐κB axis.

### 2.7 Abrine Enhances Antitumor Immunity by Targeting PLD3 in Tumor‐Associated Macrophages

**FIGURE 8:**
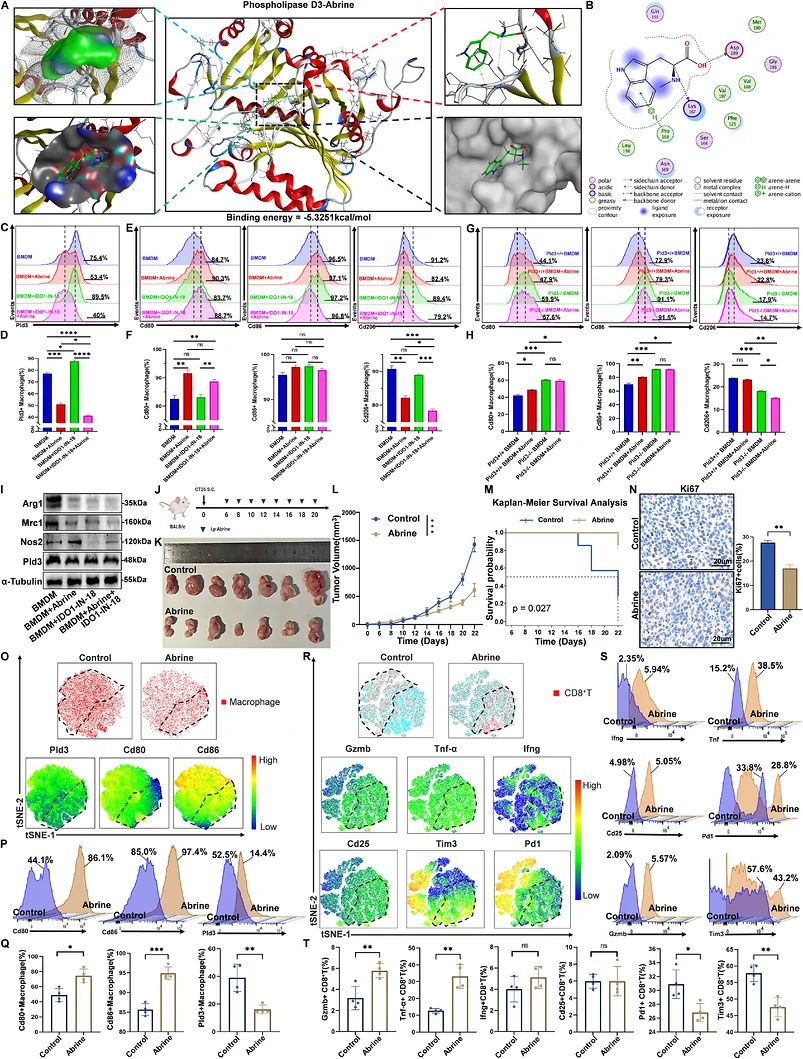
Abrine enhances antitumor immunity by targeting PLD3 in tumor‐associated macrophages. (A,B) Molecular docking of Abrine with PLD3 using Molecular Operating Environment (MOE) and docking sites. (C–F) Representative histograms and bar plots showing the fluorescence intensity distribution of Pld3 (C,D), Cd80 (E,F, Left), Cd86 (E,F, Middle), and Cd206 (E,F, Right) of BMDM under different culture conditions. BMDM was additionally treated with Abrine (40 µm) in BMDM+Abrine group and treated with IDO1‐IN‐18 (10 µM) in BMDM+IDO1‐IN‐18 group for 48 h (*n* = 3 per group). (G,H) Representative histograms and bar plots showing the fluorescence intensity distribution of Cd80 (Left), Cd86 (Middle), and Cd206 (Right) of Pld3+/+ BMDM and Pld3‐/‐ BMDM stimulated with Abrine for 48 h (*n* = 3 per group). (I) Western blotting detects the expression of Pld3, Nos2, Mrc1, and Arg1 of BMDM stimulated with Abrine or IDO1‐IN‐18 for 48 h. (J–M) Tumor growth of CT26 MSS CRC tumor‐bearing mice treated with PBS control or Abrine (50 mg/kg) (*n* = 7 per group), and overall survival of the mice was shown (M). (N) Representative images of immunohistochemical staining for Ki67 in subcutaneous tumors from mice treated with PBS control or Abrine (*n* = 3 per group). Scale bar, 20 µm. (O–Q) tSNE embeddings, histograms, and bar plots of flow cytometry analysis of Pld3, Cd86, and Cd80 expression in Cd11b+F4/80+ macrophages of subcutaneous tumors from mice treated with PBS control or Abrine (*n* = 4 per group). (R–T) tSNE embeddings, histograms, and bar plots of flow cytometry analysis of effect markers (Gzmb, Tnf‐a, Ifng, and Cd25) and exhausted markers (Tim3 and Pd1) in Cd8^+^ T cell of subcutaneous tumors from mice treated with PBS control or Abrine (*n* = 4 per group). Data are means ± SEM, and the P values were calculated by two‐way ANOVA (L), log‐rank (Mantel‐Cox) test (M), one‐way ANOVA (D, F, H), and unpaired, two‐tailed Student's t test (Q, T). *p < 0.05, **p < 0.01, ***p < 0.001, ****p < 0.0001 and ns, not significant. [Correction added on 1 June 2026, after first online publication: Figure 8 has been updated in this version.]

Therapeutic targeting of macrophage‐specific PLD3 may represent a promising strategy to potentiate antitumor immunity in CRC. Capitalizing on the lysosomal localization of PLD3 and the tendency of targeted drugs to accumulate in lysosomes, we prioritized inhibitors of PLD3 predicted by the Comparative Toxicogenomics Database (CTD). Abrine was identified through MOE and AutoDock simulations as a high‐potency binder to PLD3, with specific interaction sites mapped (Figure 8A,B and Figure S16A,B and Table S2). CCK‐8 assays showed Abrine (≤40 µM) inhibited RAW264.7 cell growth by less than 20% after 48 h, with no observed cytotoxicity (Figure S16C).

Notably, Abrine suppressed Pld3 expression while downregulating Apoe, Mrc1, and Arg1, and upregulating Nos2 (Figure 8C,D, I and Figure S16D–F). Abrine suppresses CD47 expression by inhibiting IDO, thereby limiting tumor immune evasion [9]. To determine whether Abrine inhibits PLD3 independently of IDO, macrophages were treated with an IDO inhibitor in combination with Abrine. Under these conditions, PLD3 expression remained reduced, and the antitumor phenotype was further enhanced (Figure 8C–F,I). Furthermore, in Pld3‐deficient macrophages, Abrine treatment did not significantly alter the antitumor phenotype (Figure 8G,H).

In vivo, Abrine demonstrated potent antitumor activity, significantly suppressed the growth of MSS subcutaneous tumors, prolonged overall survival (Figure 8J–M), and reduced the Ki67 index (Figure 8N and Figure S16G). Flow cytometry analysis of macrophages isolated from subcutaneous tumors revealed Abrine downregulated Pld3 expression while upregulating Cd86 and Cd80 (Figure 8O–Q and Figure S10A,B). Concurrently, CD8^+^ T cells exhibited upregulated effector molecules (Gzmb, Tnf) and downregulated exhaustion markers (Tim3, Pd1) following Abrine treatment (Figure 8R–T and Figure S10C,D). Following RNA extraction from subcutaneous tumors, RT‐qPCR analysis demonstrated Abrine treatment significantly reduced mRNA levels of Pld3, Arg1, p65, and Apoe (Figure S16H). Evaluation of the potential drug‐induced organ damage showed no significant damage in the liver, kidney, and spleen after Abrine treatment (Figure S16I–N and Tables S3–S5).

### 2.8 Abrine Reprograms Macrophages to Remodel the immune Microenvironment and Boost Antitumor Immunity

**FIGURE 9:**
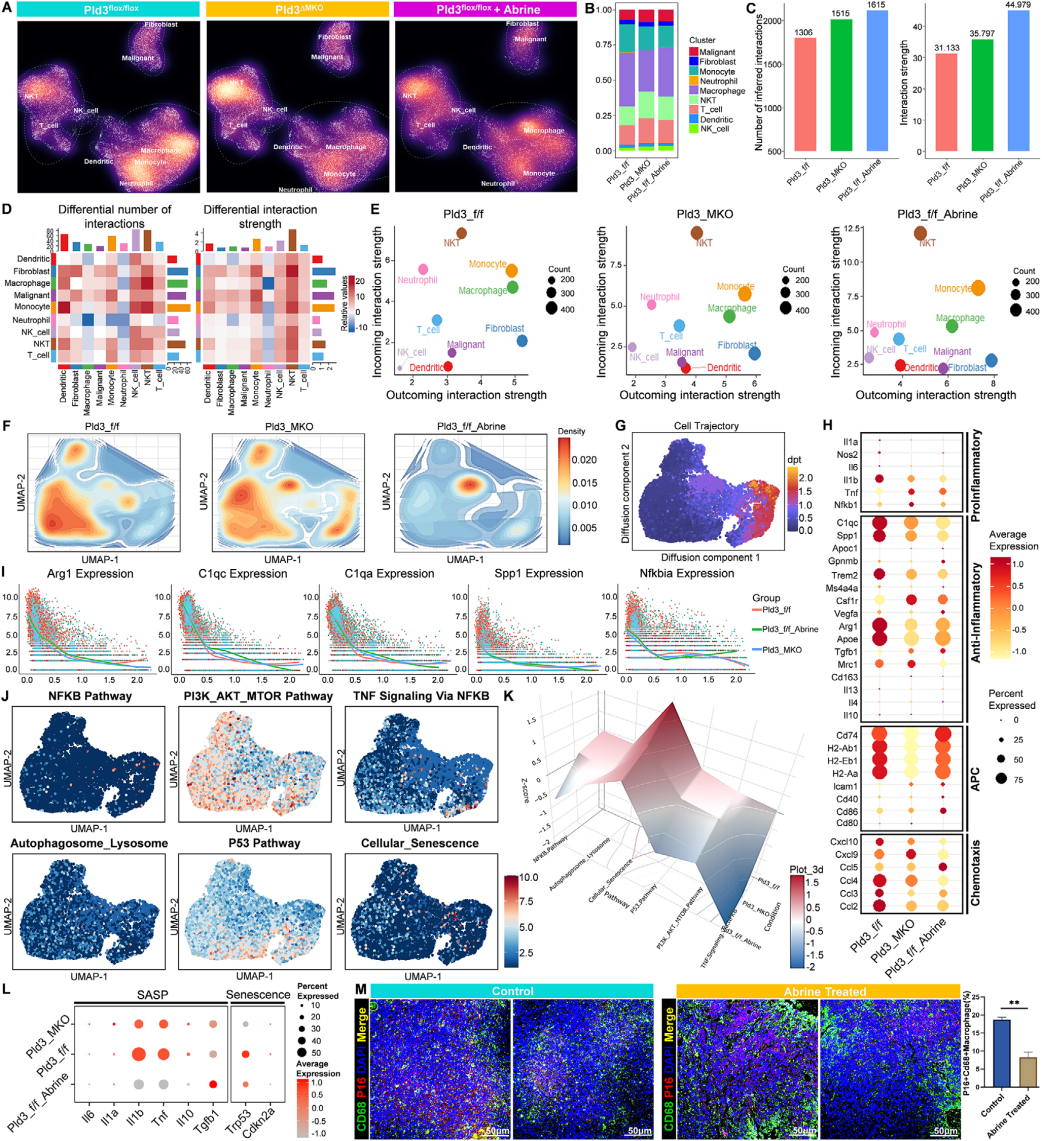
Abrine reprograms macrophages to remodel the immune microenvironment and boost antitumor immunity. (A) Differences in cell densities among Pld3^flox/flox^, Pld3^ΔMKO^ and Pld3^flox/flox^+Abrine mice using scRNA‐seq data. (B) Stack plot displaying the abundance of subclusters. (C) Number and strength of inferred interactions, comparing Pld3^flox/flox^, Pld3^ΔMKO^ and Pld3^flox/flox^+Abrine group. (D) Heatmap showing the differential communication among various cell types between Pld3^flox/flox^ and Pld3^flox/flox^+Abrine (red indicates upregulated cellular communication in Pld3^flox/flox^+Abrine, while blue represents downregulated interactions in Pld3^flox/flox^+Abrine). (E) Bubble plot showing Incoming and Outcoming interaction strength among various cell types from Pld3^flox/flox^, Pld3^ΔMKO^ and Pld3^flox/flox^+Abrine mice. (F) Differences in macrophage subcluster densities among Pld3^flox/flox^, Pld3^ΔMKO^ and Pld3^flox/flox^+Abrine from subcutaneous tumors. (G) Diffusion map analysis reveals the developmental trajectory of macrophages. (H) Dot plot of macrophage of subcutaneous tumors from Pld3^flox/flox^, Pld3^ΔMKO^ and Pld3^flox/flox^+Abrine mice, showing the RNA expression of marker genes used to define the function of Proinflammatory, Anti‐inflammatory, APC, and Chemotaxis. (I) Temporal expression dynamics of inflammatory and polarization‐associated genes in macrophages of subcutaneous tumors from Pld3^flox/flox^, Pld3^ΔMKO^ and Pld3^flox/flox^+Abrine mice. (J) UMAP embedding of macrophages, colored by pathway enrichment scores, including NF‐κB pathway, PI3K‐AKT‐mTOR pathway, TNF signaling via NF‐κB, Autophagosome‐Lysosome pathway, P53 signaling pathway, and Cellular senescence pathway. (K) 3D heatmap showing the average pathway activity scores of macrophages of subcutaneous tumors from Pld3^flox/flox^, Pld3^ΔMKO^ and Pld3^flox/flox^+Abrine mice. (L) Dot plot of macrophages of subcutaneous tumors from Pld3^flox/flox^, Pld3^ΔMKO^ and Pld3^flox/flox^+Abrine mice, showing the RNA expression of marker genes used to define SASP and Senescence. (M) Representative multiplex immunofluorescence images of DAPI, P16 (CDKN2A), and CD68 in MC38 subcutaneous tumor samples from Control and Abrine‐treated mice (*n* = 3 per group). Scale bar, 20 µm. Data are means ± SEM, and the P values were calculated by unpaired, two‐tailed Student's *t* test (M). *p < 0.05, **p < 0.01, ***p < 0.001, ****p < 0.0001 and ns, not significant.

To further explore the impact of Abrine on the TME, scRNA‐seq was performed on single cells isolated from MC38 subcutaneous tumors of Abrine‐treated Pld3^fl/fl^ mice. The results revealed increased proportions of NKT, T, and NK cells (Figure 9A,B) and also confirmed Abrine specifically targets Pld3 in TAMs (Figure S17A–C).

We subsequently analyzed cell–cell communication networks within subcutaneous tumors derived from Pld3^fl/fl^, Pld3^ΔMKO^, and Abrine‐treated Pld3^fl/fl^ mice. Our findings indicate Abrine treatment significantly enhanced the overall communication intensity among cell subclusters (Figure 9C and Figure S17D). Compared with Pld3^fl/fl^ mice, macrophage interactions with NKT and T cells were amplified following Abrine treatment, aligning with the increased communication in Pld3^ΔMKO^ (Figure 9D, Figure S11E), suggesting Abrine targets PLD3 in macrophages, creating a TME similar to Pld3^ΔMKO^ mice.

Intriguingly, Abrine treatment enhanced global communication networks more substantially than Pld3^ΔMKO^. Compared with Pld3^ΔMKO^, enhanced signaling from macrophages, monocytes, and NKT cells toward dendritic cells was observed, suggesting Abrine may further potentiate dendritic cell‐mediated tumor recognition and killing functions (Figure S17E). Moreover, we observed that NKT and T cells concurrently demonstrated an intensification of their overall communication signals after Abrine treatment (Figure 9E and Figure S17F–H).

Additionally, macrophages were categorized into clusters 0 to 8, and clusters 4, 5, and 7 showed increased infiltration in Pld3^ΔMKO^ and Abrine‐treated mice compared to the control group (Figure 9F and Figure S17I,J). Macrophages from both Abrine‐treated Pld3^fl/fl^ and Pld3^ΔMKO^ mice exhibited reduced differentiation plasticity compared with those from Pld3^fl/fl^ mice (Figure 9G and Figure S17K). Consistently, scRNA‐seq confirmed Abrine decreased the expression of anti‐inflammatory factors (Figure 9H,I).

Furthermore, compared to the control group, macrophages from Pld3^ΔMKO^ and Abrine‐treated mice demonstrated enrichment in NF‐κB signaling, p53 pathway, PI3K_AKT_mTOR Pathway, cellular senescence, autophagosome‐lysosome, and nucleotide metabolism (Figure 9J,K and Figure S17L). GSVA analysis indicated PI3K‐AKT‐MTOR pathway in macrophages was downregulated, as were the DNA repair and MYC target gene pathways in NKT cells, while MTORC1 signaling in T cells was upregulated following Abrine treatment, matching the trends observed in Pld3^ΔMKO^ mice (Figure S17M). Consistently, Abrine treatment was confirmed to attenuate the senescent phenotype in macrophages (Figure 9L,M).

Collectively, these findings reveal that Abrine targets PLD3 in macrophages, alleviates macrophage senescence, and facilitates intercellular communication to enhance immune cell recognition of tumor cells, thereby dismantling the immune barrier established by interactions such as PD‐1‐PD‐L1 engagement.

### 2.9 Targeting PLD3 Synergizes With Immunotherapy to Enhance Antitumor Efficacy

**FIGURE 10:**
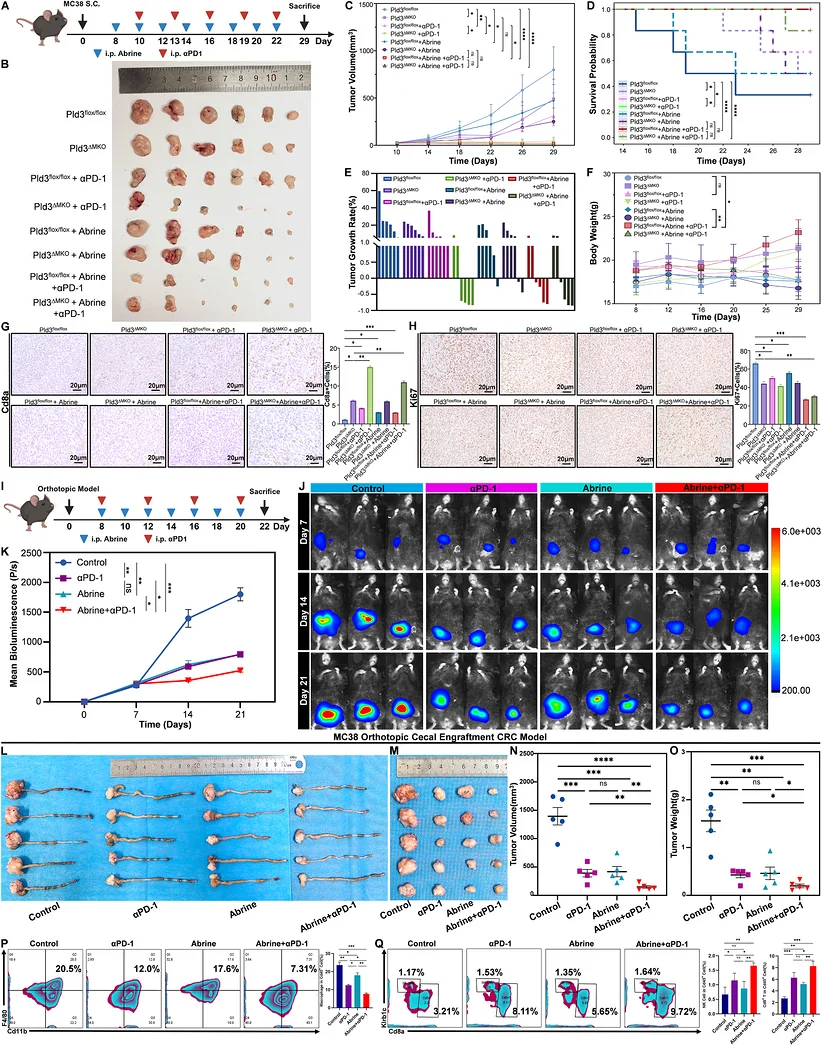
Targeting PLD3 synergizes with immunotherapy to enhance antitumor efficacy. (A–F) Tumor growth of MC38 subcutaneous tumors from Pld3^flox/flox^ and Pld3^ΔMKO^ mice treated with PBS control, anti‐PD‐1(200 µg/animal, q3d) or Abrine treatment (50 mg/kg, q2d) (*n* = 6 per group). Experimental design with eight groups: Pld3^flox/flox^, Pld3^ΔMKO^, Pld3^flox/flox^+αPD‐1, Pld3^ΔMKO^+αPD‐1, Pld3^flox/flox^+Abrine, Pld3^ΔMKO^+Abrine, Pld3^flox/flox^+Abrine+αPD‐1 and Pld3^ΔMKO^+Abrine+αPD‐1. (A) Experiment design. (B) Macroscopic appearance of subcutaneous tumors from different treatment groups. (C) Tumor Volume. (D) Overall survival. (E) Tumor Growth Rate. (F) Body Weight. (G) Representative images and statistical analysis of immunohistochemical staining for Cd8a in subcutaneous tumors of eight groups (*n* = 6 per group). Scale bar, 20 µm. (H) Representative images and statistical analysis of immunohistochemical staining for Ki67 in subcutaneous tumors of eight groups (*n* = 6 per group). Scale bar, 20 µm. (I) Experiment design of MC38 / CMT93 orthotopic cecal engraftment model. (J–O) Tumor growth of MC38 orthotopic cecal engraftment model from C57BL/6 mice treated with PBS control, anti‐PD‐1(200 µg/animal, q4d) or Abrine treatment (50 mg/kg, q2d) (*n* = 5 per group). (J,K) Tumor growth was monitored by bioluminescent imaging on days 7, 14, and 21. (L,M) Macroscopic appearance of MC38 orthotopic MSI‐H CRC tumors for each indicated treatment. (N) Tumor Volume. (O) Tumor weight. (P) Flow cytometry analysis showing the proportion of F4/80+Cd11b+Macrophages in MC38 orthotopic CRC tumors (*n* = 3 per group). (Q) Flow cytometry analysis showing the proportion of Klrb1c^+^ NK cells and Cd8a^+^ T cells in MC38 MSI‐H orthotopic CRC tumors (*n* = 3 per group). Data are means ± SEM, and the P values were calculated by two‐way ANOVA (C, F, K), log‐rank (Mantel‐Cox) test (D) and one‐way ANOVA (G‐H, N‐Q). *p < 0.05, **p < 0.01, ***p < 0.001, ****p < 0.0001 and ns, not significant.

The infiltration proportion of PLD3+Macro is increased in SD patients undergoing CRC immunotherapy, suggesting its role in immune resistance. We performed a combined treatment with Abrine and anti‐PD‐1 in Pld3^fl/fl^ and Pld3^ΔMKO^ mice (Figure 10A,B). Initially, the results confirmed macrophage‐specific Pld3 knockout suppresses tumor growth and enhances the antitumor efficacy of Abrine. Furthermore, the results suggest Abrine ablated the function of Pld3 in macrophages, thereby promoting antitumor responses. Additionally, we confirmed the antitumor effects of anti‐PD‐1 and validated the suppression of macrophage Pld3 enhanced the efficacy of ICIs. In addition, Abrine augmented anti‐PD‐1 efficacy through macrophage‐selective inhibition of Pld3. Ultimately, anti‐PD‐1 treatment, myeloid‐specific Pld3 knockout, Abrine treatment, and their combination all significantly prolonged mouse survival (Figure 10B–E and Figure S18A,B).

Moreover, compared with tumor‐bearing mice without intervention, the combination therapy of Abrine and anti‐PD‐1 treatment not only inhibited tumor growth but also increased mouse body weight, thereby alleviating the detrimental impact of tumor burden on overall physiological condition (Figure 10F). Pld3 knockout in macrophages, Abrine, and anti‐PD‐1 treatment all enhanced CD8^+^ T cell infiltration, with combined PLD3 knockout and anti‐PD‐1 treatment further augmenting infiltration (Figure 10G and Figure S19A). Additionally, the Ki67 proliferation index positively correlated with tumor growth, matching tumor volume measurements (Figure 10H and Figure S19B).

Consistently, in MC38 MSI‐H and CMT93 MSS CRC orthotopic cecal engraftment models [18], both Abrine and anti‐PD‐1 treatment suppressed tumor growth, and Abrine further enhanced the therapeutic efficacy of PD‐1 blockade (Figure 10I–O and Figure S19C–F). Flow cytometric analysis of MC38 orthotopic cecal tumors revealed that both Abrine and anti‐PD‐1 treatment reduced macrophage infiltration while promoting NK cell and CD8^+^ T cell infiltration. Notably, the combination of Abrine and anti‐PD‐1 further potentiated these effects (Figure 10P,Q and Figure S10E).

Additionally, in the MC38 subcutaneous tumor model, we further analyzed the weights of mouse colon, liver, spleen, and kidney, which revealed no significant alterations in these vital organs after PLD3 knockout in macrophages, Abrine treatment, and anti‐PD‐1 treatment (Figure S18C–H). Moreover, assessment of drug‐induced side effects on the liver, kidneys, and spleen showed no irreversible adverse effects resulting from Pld3 knockout, Abrine treatment, or anti‐PD‐1 treatment (Figure S18I and Table S6–S8).

These findings strongly suggest targeting PLD3 could enhance immunotherapy efficacy in CRC patients resistant to ICIs. Specifically, for patients with SD or PD exhibiting high PLD3+Macro infiltration, combining PLD3 inhibitors like Abrine with existing immunotherapies may reverse resistance and improve survival outcomes.

## 3 Discussion

This study demonstrates that suppression of PLD3 expression in macrophages, or pharmacological inhibition using Abrine, significantly enhances the infiltration of inflammatory cells within the TME, suppresses CRC progression, and synergistically improves the efficacy of anti‐PD‐1 immunotherapy. Currently, only a limited proportion of patients benefit from immunotherapy, primarily due to the complexity of the TME and the heterogeneity of immune cell populations. Identifying cellular subclusters that mediate resistance to immunotherapy has become a critical research priority.

TAMs are the most abundant innate immune cells, showing an anti‐inflammatory phenotype within TME [4, 19, 20]. ScRNA‐seq has enabled the identification of multiple TAMs subclusters. SPP1+Macro have been linked to progression in gastric cancer [21]. head and neck squamous cell carcinoma [22]. prostate cancer [23], oral squamous cell carcinoma [24], and colorectal cancer [25]. TREM2+ macrophages drive progression in pancreatic cancer [26], hepatocellular carcinoma [27], glioblastoma [28], and breast cancer [29], Macrophage polarisation (CXCL9:SPP1 ratio) is critical in TME, predicting antitumor immune cell abundance and immunotherapy response [14], Established TAMs markers include GPNMB, SPP1, C1QC, TREM2, APOE, and MS4A4A [30].

Enrichment of PLD3+Macro in SD or PD patients correlates with tumor immune evasion and diminished response to ICIs. Mechanistically, we identified a novel PLD3+Macro subcluster driving CRC progression and immunotherapy resistance. By establishing a subcutaneous tumor model in myeloid‐specific Pld3‐knockout mice and utilizing techniques such as scRNA‐seq and RNA‐seq, we found that Pld3 knockout in macrophages inhibits the anti‐inflammatory phenotype of macrophages and TAMs markers, while enhancing the infiltration of T, NK, and NKT cells, thereby fostering an immune‐inflammatory TME.

Given that Pld3 expression was predominantly restricted to macrophages with negligible detection in neutrophils, the phenotypes observed in myeloid‐specific Pld3‐knockout mice can be attributed primarily to Pld3 deficiency in macrophages rather than in neutrophils. These findings confirm the specificity of the myeloid‐specific Lyz2‐Cre‐mediated Pld3 knockout model and support the interpretation that the observed phenotypes are attributable to macrophage‐intrinsic Pld3 function.

The phospholipase D (PLD) protein family is primarily recognized for its canonical members, PLD1 and PLD2, which hydrolyze phosphatidylcholine to generate phosphatidic acid and choline [31]. PLD1 and PLD2 regulate membrane trafficking, cytoskeletal reorganization, receptor endocytosis, phagocytosis, and cellular processes [32, 33]. PLD3, a type II transmembrane protein, lacks classical phospholipase activity [34, 35]. It has been found to function as a lysosomal 5′–3′ exonuclease that degrades DNA [16, 36]. PLD3 is linked to Alzheimer's disease through β‐amyloid plaque formation [37, 38]. Its deficiency causes mitochondrial DNA (mtDNA) accumulation in lysosomes, leading to the accumulation of amyloid precursor protein C‐terminal fragments and cholesterol [15]. Our study shows PLD3 colocalises with lysosomal protein Lamp2 in macrophages, and that PLD3 depletion reduces Lamp2 expression. These findings demonstrate the role of PLD3 in lysosomal function, vesicular trafficking, and tumor progression, warranting further investigation.

The interaction between PLD3 and ARG1 appears counterintuitive given their distinct subcellular localizations: PLD3 is a lysosomal type II transmembrane protein [34, 35]. whereas ARG1 is primarily cytosolic. Nevertheless, recent studies have demonstrated that ARG1 can localize to the mitochondrial membrane [39]. Despite this, orthogonal evidence supports their functional association. Their colocalization in CD68^+^ macrophages, Co‐IP/MS validation, and reduced Arg1 expression upon Pld3 knockdown collectively argue for their interaction. Notably, immunofluorescence revealed that PLD3 does not fully colocalize with the lysosomal marker LAMP2, suggesting that PLD3 may localize to subdomains or dynamic compartments beyond canonical lysosomes. This association may be facilitated by dynamic membrane remodeling, such as at lysosome‐ER contact sites, enabling transient or indirect interactions. However, based on a comprehensive literature review and cross‐sectional experimental evidence, we suggest that functional interactions may exist between PLD3 and ARG1 in macrophages, which is worthy of further in‐depth exploration.

Cellular senescence, a terminally differentiated state, contributes to tumorigenesis [40] and ageing [41]. PLD3 was identified as a marker of cellular senescence [42]. The AKT‐NF‐κB pathway is pivotal in aging‐related inflammation. Radiotherapy induces cellular senescence by degrading AKT phosphatase in liver cancer cells, thereby activating the AKT‐p53‐p21 pathway [43]. Under chronic inflammatory, NF‐κB activation promotes senescence through telomere shortening [44]. Our study demonstrates PLD3 modulates macrophage senescence via the lysosome‐AKT‐NF‐κB axis. Lysosomes located at the cell periphery, as opposed to perinuclear lysosomes, exhibit greater competence in activating the AKT signaling pathway, with AKT being partially localized to lysosomes [17]. Excessive PLD3 expression suppresses AKT phosphorylation in C2C12 myoblasts [45]. Additionally, PLD3 deficiency leads to mitochondrial DNA accumulation within lysosomes, leading to subsequent cytosolic leakage [15], thereby activating TLR9 and NF‐κB signalling [46]. NF‐κB activation is essential for macrophage responses [47, 48] and tumor development. During chronic inflammation, macrophages release cytokines, triggering prosurvival signals via NF‐κB or STAT3 activation [49]. PLD3 links to p53 pathway and senescence regulation. Aged mice macrophages show enhanced p53 activation, while Pld3 deletion reduces senescence. AKT pathway agonist SC79 rescues diminished senescence from Pld3 knockout, suggesting PLD3 regulates senescence via the lysosomal‐AKT‐NF‐κB axis.

Targeting TAMs has emerged as a promising strategy to enhance immunotherapy sensitivity. Current approaches include depleting existing macrophages, inhibiting monocyte recruitment, and re‐educating TAMs toward an “immune‐supportive” phenotype [50]. However, depletion and recruitment inhibition may disrupt the immune functions of TAMs, worsening tumor progression. By contrast, targeting TAMs marker genes with the small molecule offers a refined approach while preserving their beneficial roles.

Abrine, a major alkaloid extracted from Abrus andoniensis and Abrus precatorius Linn, exhibits hepatoprotective properties and inhibits IDO [51, 52]. By inhibiting IDO, Abrine reduces CD47 expression to limit tumor immune evasion [9]. Meanwhile, Abrine enhanced CD8^+^ T‐cell infiltration, reduced Treg abundance and PD‐L1/CD47 expression, potentiating the efficacy of anti‐PD‐1 therapy against hepatocellular carcinoma [9, 53]. Our study first demonstrated that Abrine targets PLD3 in macrophages and promotes the infiltration of CD8^+^ T cells, enhancing tumor immunotherapy. Our preliminary assessment confirms the biosafety of abrine in vivo, as evidenced by the absence of obvious organ damage in treated mice.

Notably, Abrine's effect on PLD3 is independent of IDO, indicating a distinct regulatory axis. As PLD3 shares structural homology with PLD4, another family member involved in immune modulation, it remains possible that Abrine may have potential effect on PLD4. Further structural studies are warranted to define the molecular basis of Abrine's target specificity. Additionally, while systemic administration of Abrine yielded promising antitumor outcomes in mice, we acknowledge that the compound may exert direct effects on tumor cells or other immune populations beyond macrophages. Dissecting these cell‐type‐specific contributions will be an important focus of future studies. Moreover, further preclinical and clinical investigations are necessary to establish the therapeutic potential of Abrine.

Additionally, as PLD3 is also highly expressed in macrophages across various malignancies, this study provides insights relevant to other cancer types.

## 4 Conclusion

**FIGURE 11:**
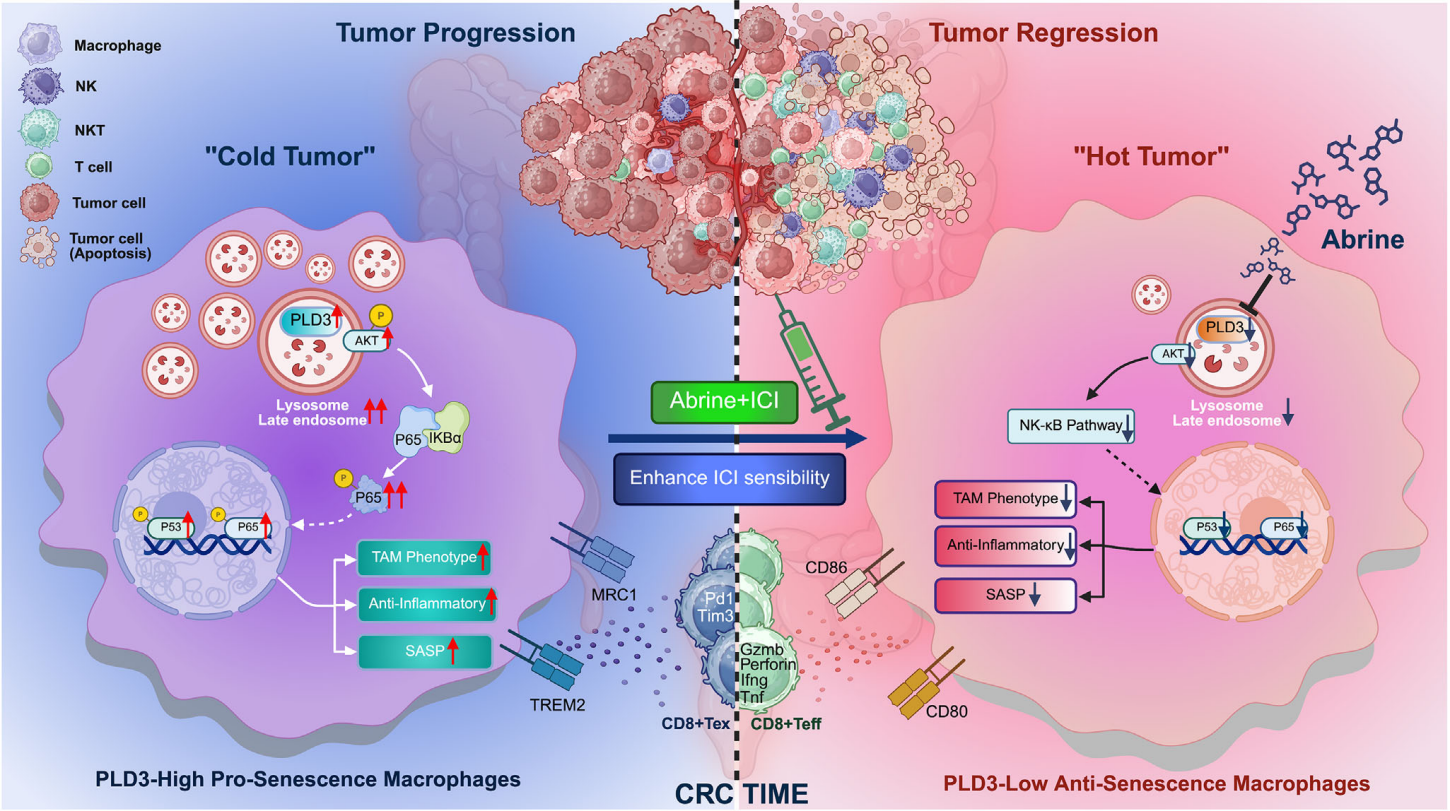
Proposed working model based on this study. PLD3 triggers senescence and immunosuppressive TME via the lysosomal‐AKT‐NF‐κB axis, curbing cytotoxic T, NK, and NKT cells infiltration and fostering anti‐PD‐1 resistance. Abrine inhibits PLD3 expression in TAMs, restoring antitumor immunity and synergising with anti‐PD‐1 therapy.

In conclusion, our findings identify PLD3+Macro as a pivotal regulator of CRC immune evasion. PLD3 induces senescence and immunosuppressive TME through the lysosomal‐AKT‐NF‐κB axis in macrophages, thereby inhibiting cytotoxic T, NK, and NKT cells infiltration and promoting resistance to anti‐PD‐1 therapy. Abrine, by inhibiting PLD3 expression, restores antitumor immunity and synergizes with anti‐PD‐1 therapy to improve survival (Figure 11). Collectively, our findings elucidate the impact of PLD3 in CRC TME and reveal novel therapeutic avenues for overcoming resistance to immunotherapy.

## 5 Experimental Section

### 5.1 Data Acquisition and Preprocessing

RNA transcriptome sequencing (RNA‐seq) and four‐dimensional data‐independent acquisition(4D‐DIA) protein quantification sequencing were performed on Pld3+/+ BMDM and Pld3‐/‐ BMDM. RNA was extracted and sequenced at Berry Genomics Co. Ltd. with an Illumina NovaSeq X Plus sequencer. Briefly, reads were aligned to GRCm38 (mm10) annotations using Hisat2. Protein samples were enzymatically digested and fractionated, followed by DIA‐based proteomic analysis on the Orbitrap Astral mass spectrometer. DIA raw data were processed using spectral library‐based algorithms for protein identification and quantification.

ScRNA‐seq was performed on 5 mouse subcutaneous tumor samples at Shanghai Xu Ran Biotechnology Co., Ltd with a DNBSEQ‐T7 PE150 sequencer, including 2 Pld3^flox/flox^ (Pld3_f/f, Pld3^fl/fl^) samples, 2 Pld3^flox/flox^Lyz2‐Cre (Pld3_MKO, Pld3^ΔMKO^) samples and 1 Pld3^flox/flox^ + Abrine (Pld3_fl/fl_ Abrine) sample. Following tumor tissue dissociation into single‐cell suspensions, CD45+ cells were magnetically isolated (Miltenyi Biotec, 130‐052‐301), and a controlled mixture consisting of 85% CD45+ immune cells and 15% CD45− non‐immune cells was prepared before sequencing. The raw proteomic data were processed using DNBC4tools (version 2.1.3). Batch effect was corrected, and the merged object was integrated by running Harmony (version 1.2.0). Quality control measures were implemented, with cells having fewer than 200 or more than 4000 expressed genes, more than 15000 total UMIs, over 10% mitochondrial genes, more than 1% hemoglobin genes, and over 50% ribosomal genes being excluded. The LogNormalize method ensured data normalization. The FindVariableFeatures function was used to identify the top 2000 variable genes. Principal component analysis and UMAP were applied for dimensionality reduction, followed by cell clustering using the FindNeighbors and FindClusters functions. Cell annotation was facilitated using the R package SingleR (version 2.0.0) (Aran et al., 2019). Module scores for pathway activation and gene signatures were calculated using the AddModuleScore function from Seurat (version 4.4.0).

To comprehensively investigate the molecular characteristics of CRC, we obtained bulk RNA‐sequencing data from a total of 362 patients with Colon Adenocarcinoma (COAD) along with gene expression profiles of 391 primary tumor tissues and 39 paired adjacent normal tissues, together with corresponding clinical information, from The Cancer Genome Atlas (TCGA) database (https://portal.gdc.cancer.gov/). To collect single‐cell datasets for CRC, immunotherapy‐related single‐cell datasets, and spatial transcriptomics datasets for colorectal cancer, we selected the GSE236581, GSE161277, GSE178341, and GSE226997 datasets from the GEO database (http://www.ncbi.nlm.nih.gov/geo).

The pan‐cancer single‐cell analysis of PLD3 was performed on the Tumor Immune Single‐Cell Hub (Tisch) website (http://tisch.compgenomics.org/) with the following conditions: Gene: PLD3; cell type annotation: major lineage; all lineage. PLD3‐expressing cell subsets in colorectal cancer were identified using the Single‐cell Colorectal Cancer Atlas (https://crc.icbi.at). Survival analysis of PLD3 in colorectal cancer and its impact on survival in immunotherapy‐treated patients was performed on Kaplan‐Meier Plotter (https://kmplot.com/analysis/index.php). Prediction of ICIs response based on PLD3 expression was performed on the TIDE Platform (http://tide.dfci.harvard.edu).

### 5.2 Spatial Transcriptomics Analysis

Processing of Visium spatial transcriptomics data from the GSE226997 dataset was conducted using Seurat. Spots were filtered based on a minimum of 200 detected genes, and lowly expressed genes (expressed in < 3 spots or with < 10 total reads) were excluded. Normalization was performed with the LogVMR method to correct for technical variation. Following PCA dimensionality reduction, the first 30 principal components (PCs) were selected for clustering analysis. Spatial feature visualization was achieved using the SpatialFeaturePlot function. Tumor parenchyma and stroma were distinguished in spatial transcriptomic sections using the SpaCET R package (version 1.4.0).

For spatial distance analysis, CD68+ macrophages were classified as PLD3+ if their normalized expression value of PLD3 was >0, and as PLD3‐ if the value was ≤ 0. PD1+CD8A+ T cells were defined as cells with normalized expression of both PD1 and CD8A >0. Spatial distances between CD68+ macrophages and CD8+ T cells were computed using the phenoptr R package (version 0.3.2), calculating the euclidean distance from each macrophage centroid to the nearest CD8+ T cell centroid. Group comparisons were performed using the Wilcoxon rank‐sum test, and distance distributions were visualized with density plots and spatial visualization.

### 5.3 Animal Experiments

We crossed Lyz2‐Cre recombinase transgenic C57BL/6 mice and Pld3^flox/flox^ C57BL/6 mice (purchased from Shulaibao (Wuhan) Biotechnology Co., Ltd., contract number: SLB‐DG‐2024) and generated myeloid‐specific conditional Pld3 knockout mice (Lyz2‐Cre+, Pld3^flox/flox^ mice, abbreviated as Pld3^ΔMKO^). Mouse genotyping was performed using the Mouse Genotyping Kit (Vazyme, PD101‐01), and the mouse genotyping primers used are provided in Supplementary Table S10. BALB/c mice were obtained from the Animal Center of Southern Medical University, Guangzhou, China. Age‐ and gender‐matched mice were used for further experiments.

For the subcutaneous tumor xenograft models, CT26 colon cancer cells (5 × 10^5^ cells per animal) or MC38/MC38‐luc colon cancer cells (5 × 10^5^ cells per animal) were subcutaneously (s.c.) injected into experimental mice to form solid tumors. To preliminarily evaluate the impact of macrophages on tumor cells, CT26 cells and RAW264.7‐shNC/shPld3 cells were mixed at 5:1 ratio and subcutaneously (s.c.) injected into BALB/c mice. All the mice were sacrificed before the subcutaneous tumor volume reached 2000 mm^3^. Survival was monitored for 21 days, with euthanasia criteria defined as: (1) tumor diameter >15 mm, or (2) severe clinical deterioration (e.g., >20% weight loss, lethargy, or impaired mobility).

For the orthotopic cecal engraftment CRC mouse model, the MC38‐luc or CMT93 subcutaneous solid tumors were dissected into 1 mm^3^ fragments and maintained in ice‐cold PBS. Under anesthesia 1% sodium pentobarbital, the tumor fragments were inoculated orthotopically in the vascularized area of the cecum. After 3–4 weeks, the mice were sacrificed, and the colons were harvested to assess the tumor burden.

For treatment experiments, mice received i.p. injection of anti‐mouse PD‐1 antibodies (200 µg/animal, q3d or q4d) to block PD‐1, or received i.p. injection of Abrine (50 mg/kg, q2d) to target Pld3 in macrophages. Mice received i.p. injection of PBS as controls.

During an experiment, subcutaneous tumor growth was monitored twice or three times per week by measuring tumor size using a digital caliper; tumor volumes (length × (width^2^) /2) were calculated. For in vivo imaging, anesthetized mice received i.p. injection of D‐luciferin (15 mg/mL, 150 mg/kg), followed by a 10–20 min incubation to allow the bioluminescent signal to reach peak stability before imaging (Bruker).

At study endpoints, solid tumors were collected and analyzed by RT‐qPCR, IHC, scRNA‐seq or flow cytometry. Mice are raised under SPF conditions. Animal experiments were approved by the Laboratory Animal Ethics Committee of Southern Medical University (approval No. SMUL202512053) and conducted in accordance with good veterinary practice guidelines established by the Southern Medical University Laboratory Animal Center.

### 5.4 RNA Extraction and RT‐qPCR

Total RNA was extracted using the SevenFast Total RNA Extraction Kit (Seven Innovation Biotechnology), and cDNA was subsequently synthesized through reverse transcription using a Takara kit. The levels of RNA transcripts were analyzed using the ABI7500 Real‐time PCR system. With Gapdh/β‐actin as an interval control, relative target RNA levels were estimated utilizing 2‐ΔΔCT formula. Primer sequences are provided in Supplementary Table S9.

### 5.5 Western Blot (WB)

Protein lysates were prepared, subjected to SDS‐PAGE, transferred onto PVDF membranes, and incubated with primary antibodies according to standard methods. The membranes were then incubated with species‐specific secondary antibodies. Signals were visualized using an ECL detection kit (Pierce) and visualized with a qTouch Western Blot Imager (RWD Life Science Co. Ltd, Shenzhen, China).

### 5.6 Cell Proliferation and Cytotoxicity Assay

For cell proliferation assay, cells were seeded into 96‐well plates at a density of 1 × 10^3^ cells per well, and 10 µL of CCK8 reagent (Dojindo) was added to each well. Following a 2‐h incubation, cell viability was measured by absorbance at 450 nm. For cell cytotoxicity assay, a compound was considered to have low toxicity if it exhibited <20% growth inhibition after 48 h of exposure compared with vehicle‐treated controls.

### 5.7 Histopathological Analyses

Tissue specimens were fixed with 10% formalin, embedded in paraffin, and sectioned for hematoxylin and eosin (H&E) staining. For immunohistochemical (IHC) staining, the sections were incubated with specific primary antibodies at 4°C overnight, followed by incubation with subsequent HRP‐conjugated secondary antibodies at room temperature. The sections were stained with 3,3‐diaminobenzidine solution and hematoxylin. Images were captured and examined under a microscope (Leica). The use of human tissue specimens was approved by the Medical Ethics Committee of NanFang Hospital of Southern Medical University (approval No. NFEC‐2025‐546). Written informed consent was obtained from all participants.

Multiplexed immunofluorescence (IF) was performed using the PerkinElmer‐Opal‐Kit (Akoya Biosciences, NEL811001KT) according to the manufacturer's instructions. The sections were stained with primary antibodies, HRP‐conjugated polymers, and opal fluorophores; cycles were repeated until all markers were stained. Finally, the nuclei were counterstained with DAPI. Especially, for cell IF, cells were fixed in 4% paraformaldehyde for 20 min at room temperature, and then incubated with primary antibodies for 4 hours. The cells were subsequently incubated with fluorescence‐labeled secondary antibodies, and the nuclei were stained by DAPI. Images were acquired under a confocal microscope (LSM 880 with Airyscan, N‐SIM) and analyzed using Imaris (Version 9.0.1) or ZEN (Version 2.1).

### 5.8 Cell–Cell Interaction Analysis

The CellChat (version 1.5.0) and Nichenetr (version 1.1.1) algorithms were used to study cell–cell interactions among various TME components. The input files for the statistical analysis included the raw count matrix and cell type annotation files derived from the Seurat object. Based on their average expression, visualizations of the predicted interaction intensity between ligands and receptors were generated. Significant ligand–receptor pairs were extracted for illustration.

### 5.9 SCENIC Analysis and Regulatory Networks

The SCENIC algorithm was conducted utilizing the motifs database for RcisTarget and GRNboost. The softwares of SCENIC (version 1.3.1), AUCell (version 1.20.2), and RcisTarget (version 1.18.2) were used. In detail, overrepresented transcription factor (TF) binding motifs were identified through the gene list in RcisTarget package. The AUCell package was used to score the activity of each group of regulons in every single cell.

### 5.10 Differentiation Potential and Trajectory Analysis

The Monocle2 (version 2.26.0) algorithm, CytoTRACE (version 0.3.3) algorithm, Destiny (version 3.14.0) algorithm, and Slinshot (version 2.6.0) algorithm were applied to infer cell state interconversions and evolutionary trajectories, thereby examining the plasticity and dynamic differentiation of individual cells.

The importCDS function was employed to convert the raw counts from a Seurat object into a CellDataSet object in Monocle2. The differentialGeneTest function was used to select ordering genes (qval < 0.01). The dimensional reduction clustering analysis was performed using the reduceDimension function, followed by trajectory inference with the orderCells function. Gene expression dynamics were tracked along pseudotime using the plot_genes_in_pseudotime function.

CytoTRACE is a robust computational framework for predicting differentiation states from scRNA‐seq data, validated in large‐scale datasets and shown to outperform previous computational approaches for stemness evaluation. The R package CytoTRACE was leveraged to compute the CytoTRACE scores for subclusters, wherein scores ranging from 0 to 1 were assigned, with elevated scores indicative of heightened stemness (lower differentiation), and conversely, diminished scores reflecting lower stemness.

Diffusion maps were constructed using the Destiny algorithm, which implements dimensionality reduction based on the diffusion kernel to capture the intrinsic manifold structure of single‐cell transcriptomic data.

Slingshot was applied to fit a minimum spanning tree (MST) to these clusters and determine the approximate trajectory structure. This piecewise linear trajectory was smoothed using simultaneous principal curves to arrive at the final trajectories and pseudotime values.

### 5.11 High‐Dimensional Weighted Gene Co‐expression Network Analysis(hdWGCNA)

HdWGCNA (version 0.4.03) package was applied to delineate key molecular characteristics associated with Pld3+Macro, which are implicated in ICIs treatment and resistance. A soft threshold of 5 was applied to construct a scale‐free network with optimal connectivity, leading to the identification of 20 gene modules.

We identified differentially expressed genes (DEGs) between PLD3+ and PLD3‐ macrophage populations (|log2FC| > 1, FDR < 0.05) and imported these into the STRING database (https://cn.string‐db.org/) to retrieve protein‐protein interaction information. The interaction network was subsequently analyzed in Cytoscape (version 3.9.1), and core genes were defined based on degree centrality.

### 5.12 scTenifoldKnk Virtual Knockout Analysis

Virtual knockout of PLD3 in macrophages was performed using the scTenifoldKnk package (version 1.0.1) on the GSE178341 CRC scRNA‐seq dataset. The analysis was conducted according to the default pipeline to identify gene regulatory changes following PLD3 deletion.

### 5.13 Immune Cell Infiltration

Leveraging the single cell‐derived signature matrix (GSE161277) from CRC single cell dataset described above, we then applied CIBERSORTx to dissect CRC RNA‐seq profiles from TCGA, aiming to perform the comparative analysis of immune cell infiltration patterns among CRC molecular subtypes, followed by functional characterization of the identified cell subpopulations.

### 5.14 Differential Abundance Analysis

MiloR (version 2.5.1) was employed to test for the differential cell abundance within defined neighborhoods between two conditions (Pld3^fl/fl^ versus Pld3^ΔMKO^). We first used the buildGraph function to construct a KNN graph (k = 15) using 15 principal components (*d*  =  15). The makeNeighborhoods function was then used to assign cells to neighborhoods based on their connectivity within the KNN graph. Differential abundance analysis was performed in MiloR by fitting a negative binomial generalized linear model (GLM) to cell counts in each neighborhood, with TMM normalization applied to account for inter‐sample variation in cell numbers. Significance was assessed using quasi‐likelihood (QL) F‐tests with a 10% spatial false discovery rate (FDR) threshold, and results were visualized by log2 fold change (log2FC) and spatial FDR values.

### 5.15 Differential Analysis and Functional Enrichment

Using R software's edgeR package (version 3.40.0), DEGs between Pld3+/+ BMDM and Pld3‐/‐ BMDM were compared, with the following criterion: adjust *p* value <0.05 and |log fold change (FC)| > 0.5. Gene Ontology (GO), Kyoto Encyclopedia of Genes and Genomes (KEGG) and Gene Set Enrichment Analysis (GSEA) analyses were performed on significant DEGs using the “clusterProfiler” and “org.Mm.eg.db” packages.

The FindMarkers function within the R package Seurat was used to maker genes of cell subclusters. Gene Set Variation Analysis (GSVA), a non‐parametric and unsupervised method, was applied to estimate pathway activity changes in hub marker genes of subclusters. The activities of the 50 hallmark pathways were quantified with the GSVA R package (version 1.53.3) to identify subcluster‐associated metabolic pathways, with p < 0.05 considered statistically significant. Pearson correlation analysis was performed to evaluate associations between hub marker genes and metabolic pathways. Pheatmap R packages were used for visualization. ClusterGVis package (version 0.1.1) was employed for one‐step integration of gene expression clustering, dynamic trend visualization, and functional annotation.

### 5.16 Co‐Immunoprecipitation/Mass Spectrometry (COIP/MS) and Alphafold3 Protein Interaction Prediction

Immunoprecipitation was performed using anti‐PLD3 antibody or IgG on RAW264.7 cells for High‐Performance Liquid Chromatography‐Tandem Mass Spectrometry (HPLC‐MS/MS) analysis. Briefly, cells were lysed and pre‐cleared with Protein A/G agarose beads. The supernatants were incubated with PLD3/IgG antibodies overnight at 4°C, followed by addition of Protein A/G beads. After washing, immunoprecipitated complexes were eluted in loading buffer at 95°C. Western blot confirmed specific PLD3 protein pulldown in IP‐PLD3 compared to IP‐IgG control. The samples were analyzed on Q Exactive HF‐X platform, and mass spectrometry data were searched against protein databases to identify PLD3‐interacting proteins.

Further, we use Alphafold3 to predict protein interaction. Briefly, the canonical amino acid sequence of the target protein was obtained from UniProt, and the sequence was submitted to AlphaFold Server (https://alphafoldserver.com) for protein‐protein interaction prediction using the multimer mode. The predicted template modeling (pTM) score greater than 0.5 indicated that the overall predicted fold of the complex was likely similar to the native structure. The interface predicted template modeling (ipTM) score was used to evaluate the accuracy of the predicted relative positioning among subunits within the complex.

### 5.17 Flow Cytometry

Tumors were harvested at indicated time points. Briefly, 100 mm^3^ of each tumor was minced and digested using a cocktail of collagenase IV (Solarbio, C8160), hyaluronidase (Solarbio, H8030), and DNase I (Solarbio, 9003‐98‐9) for 45–60 min at 37°C, and filtered through a 70 µm cell strainer to generate a single‐cell suspension. After red blood cell lysis (Solarbio, R1010), cells were counted and plated in phosphate‐buffered saline (PBS). Cell surface molecule staining was performed at 4°C for 30 min in PBS in the dark. For intracellular staining, cells were fixed/permeabilized in 50 µL of a saponin‐containing buffer (BD Biosciences, 3 213 870) and incubated at 4°C for 30 min in the dark. Cells were then washed with saponin‐containing buffer (BD Biosciences, 2 096 607) and resuspended in staining buffer, followed by antibody staining. Flow cytometry data were collected with a BD Fortessa cell analyzer and analyzed using FlowJo Software (Version 10.8.1, FlowJo).

### 5.18 Bone Marrow‐Derived Macrophage (BMDM) Isolation and Cell Culture

C57BL/6 mice were euthanized by cervical dislocation and immersed in 75% ethanol for 20 min. The skin was removed to expose femurs, and surrounding muscles were removed. The bones were washed with PBS and immersed in 75% ethanol. Femur ends were cut, and bone marrow cells were flushed out using DMEM medium. The cell suspension was centrifuged at 1200 rpm for 10 min and supernatant discarded. Red blood cell lysis buffer was added for 5 min, then terminated with DMEM medium and centrifuged. The cell pellets were resuspended in DMEM with 20% L929‐conditioned medium (L929 CM), counted, and plated. The culture medium was replaced every 3 days. Typically, BMDMs reached maturity after 7–9 days of culture.

Mouse CRC cell line (MC38, CMT93) and human cell line (SW480, THP‐1, Jurkat T) were cultured in RPMI1640 medium (Gibco) supplemented with 10% FBS (Gibco). Mouse CRC cell lines (CT26), mouse macrophage cell line (RAW264.7) and mouse connective tissue cell line (L929) were cultured in DMEM medium (Gibco) with 10% FBS. All cells were maintained at 37°C in a humidified incubator with 5% CO_2_.

For the human macrophage and CD8^+^ T cell co‐culture experiment, THP‐1‐derived macrophages were co‐cultured with Jurkat T cells at a 1:5 ratio in 20% SW480 conditional medium for 48 h. For murine macrophage and CD8^+^ T cell co‐culture experiment, murine splenic CD8^+^ T cells were enriched by magnetic bead sorting (Vazyme, CS103‐01) and activated with CD3/CD28 stimulation (BioGems,17A2;37.51) for 48 h. Macrophages were stimulated with 20% tumor‐conditioned medium to induce TAMs. Then, macrophages and CD8^+^ T cells were co‐cultured at a 1:5 ratio for 48 h. Then the cells were harvested (1700 rpm, 5 min), followed by flow cytometric analysis.

### 5.19 Senescence‐Associated β‐Galactosidase (SA‐β‐Gal) Staining

Cellular senescence was assessed using the Senescence‐Associated β‐Galactosidase (SA‐β‐Gal) Staining Kit (Solarbio, G1580) following the manufacturer's instructions. Briefly, cells seeded in 6‐well plates were fixed with 1 mL β‐Gal Fixative per well for 15 min at room temperature, followed by two washes with PBS. Subsequently, cells were then incubated with 1 mL of freshly prepared Staining Working Solution per well and maintained overnight at 37°C in a dry incubator (without CO_2_). Senescent cells were visualized under bright‐field microscopy and identified by the presence of blue cytoplasmic staining.

### 5.20 Plasmid and RNA Interference Constructs

The plasmids used in this study were as follows: pLKO.1‐CMV‐shRNA (Pld3)‐copGFP‐PURO, pLKO.1‐CMV‐copGFP‐PURO (Normal Control) (Tsingke Biotechnology). The sequences of shRNA of Mouse Pld3 was as follows: shPld3, 5′‐ CCTTGAATGAAATCGAGGCAT‐3′. Human PLD3 knockdown was performed using the PLD3‐targeted siRNA (sequence: 5′‐UUCUGCAUGUCCACCAUGC‐3′, Tsingke Biotechnology).

### 5.21 Screening of Drugs Targeting PLD3

Potential small‐molecule interactors of PLD3 (e.g., abrine and bisphenol A) were identified from the Comparative Toxicogenomics Database (CTD) based on their association with PLD3+Macro. Three‐dimensional(3D) conformers (SDF format) were downloaded from PubChem. We then retrieved and downloaded the 3D structure of the PLD3 gene product from the UniProt database. Following standard docking protocols, we performed automated molecular docking between the PLD3 protein and candidate compounds using AutoDock (version 4.2.6) and Molecular Operating Environment (MOE) Docking (version 2019.0102). Based on the lowest binding energy calculations, abrine was identified as the optimal compound, demonstrating substantial interaction with PLD3 and inhibiting its protein expression.

### 5.22 Scoring Criteria for Drug Induced Organ Toxicity

1. **Liver pathological changes and scoring**
  Liver histopathological changes were evaluated using a modified Ishak scoring system. The liver injury was graded based on four parameters: A. Hepatic lobular architecture; B. Intralobular inflammation and focal necrosis; C. Portal inflammation; D. Inflammatory cell infiltration around central veins. Each parameter was semi‐quantitatively scored on a 0–4 scale: 0‐None or minimal injury, 1‐Mild injury, 2‐Moderate injury, 3‐Severe injury, 4‐Very severe injury. The total liver injury score was obtained by summing the scores across all four parameters.
2. **Renal pathological changes and scoring**
  The r2enal histopathological evaluation was performed based on two key parameters: A. Degree of glomerular dilatation; B. Increase in intraglomerular cellularity. Each parameter was semi‐quantitatively scored on a 0–3 scale: A. Glomerular dilatation: 0‐None or insignificant, 1‐Mild, 2‐Moderate, 3‐Severe. B. Intraglomerular cellularity: 0‐Normal cellularity, 1‐Slight increase, 2‐Moderate increase, 3‐Marked proliferation. The total renal injury score was calculated as the sum of both parameters.
3. **Splenic pathological changes and scoring**
  The splenic histopathological evaluation was performed based on four key parameters: A. Inflammatory cell infiltration; B. White pulp atrophy with red pulp expansion; C. Congestion; D. Degree of splenomegaly. Each parameter was semi‐quantitatively scored on a 0–4 scale: 0‐None or minimal changes, 1‐Mild changes, 2‐Moderate changes, 3‐Severe changes, 4‐Very severe changes. The total spleen injury score was obtained by summing the scores across all four parameters.

Three random H&E‐stained sections per group were independently scored by pathologists. Each section was evaluated using organ‐specific criteria. Individual section scores were summed to yield a total injury score per group. The final scores were compared to evaluate drug‐induced organ toxicity.

### 5.23 Statistical Analysis

scRNA‐seq data were processed and normalized using Seurat's default LogNormalize algorithm. Bulk transcriptome sequencing data were normalized using the FPKM method. Outliers were assessed by the ROUT method (Q = 1%). Details are provided in the corresponding Methods section. All quantitative data are presented as mean ± Standard Error of the Mean (SEM). Each experiment was independently repeated at least three times, unless otherwise indicated. The sample size (n) for each statistical analysis or experiment is specified in the corresponding figure legend.

Normality of data distribution was assessed using the Shapiro–Wilk test. For normally distributed data, two‐group comparisons used unpaired two‐tailed Student's t‐tests; multiple group comparisons used one‐way ANOVA followed by Tukey's post‐hoc test; comparisons across multiple time points between two groups used two‐way ANOVA; and survival analysis was performed using the log‐rank (Mantel‐Cox) test. For high‐dimensional transcriptomic or proteomic analyses, P‐values were adjusted for multiple testing using the Benjamini–Hochberg false discovery rate (FDR) method. The significance threshold was *p* < 0.05. Asterisks signs denote significance levels as follows: *p < 0.05, **p < 0.01, ***p < 0.001, ****p < 0.0001, and ns, not significant.

Statistical analysis was performed using GraphPad Prism 9, R program 4.2.1. Flow cytometry data were analyzed using FlowJo v10.8.1. Confocal microscopy images were processed using ZEN 2.1, ImageJ 1.53t, and Imaris 9.0.1. Molecular docking was performed using AutoDock 4.2.6 and Molecular Operating Environment (MOE) 2019.0102. In vivo bioluminescence imaging data were analyzed using Bruker MI 7.2.1.22758.

## Author Contributions

X.T.Q., Q.L. and X.M.X. contributed equally to this work. X.T.Q. contributed to conceptualization, writing – review & editing, methodology, software, data curation, investigation, validation, visualization, writing – original draft, and formal analysis. Q.L. was responsible for conceptualization, methodology, software, data curation, validation, investigation, and formal analysis. X.M.X. participated in conceptualization, methodology, software, data curation, formal analysis, validation, visualization, and investigation. Y.H.W. contributed to supervision, writing – original draft, writing – review & editing, and formal analysis. Y.Z. participated in supervision, formal analysis, and software. J.Q.W. participated in software, formal analysis, supervision, and validation. Q.B.C. and Y.F. both contributed to methodology, validation, formal analysis, and supervision. S.Y.W., J.Y. and Y.P.Y. provided writing – original draft, writing – review & editing, funding acquisition, project administration, and resources. Finally, H.L.J. contributed to funding acquisition, writing – original draft, writing – review & editing, visualization, project administration, resources, supervision, conceptualization, and methodology.

## Funding

This work was supported by grants from the National Natural Science Foundation of China (82173185), Guangdong Provincial Natural Science Foundation of China (2026A1515010882, 2025A1515012614, 2024A1515010302).

## Ethics Statement

This study involves human participants and was approved by Medical Ethics Committee of NanFang Hospital of Southern Medical University (NFEC‐2025‐546) and conducted in accordance with the Declaration of Helsinki. Participants gave informed consent to participate in the study before taking part. And the animal experiments were approved by Ethical Committee of Southern Medical University (SMUL202512053) and conformed to the legal mandates and national guidelines for the care and maintenance of laboratory animals.

## Conflicts of Interest

The authors declare no conflicts of interest.

## Supporting information




**Supporting File 1**: advs75730‐sup‐0001‐SuppMat.docx.

**Supporting File 2**: advs75730‐sup‐0002‐Data.zip.

**Supporting File 3**: advs75730‐sup‐0003‐MovieS1.mp4.

**Supporting File 4**: advs75730‐sup‐0004‐FigureS1–S19.pdf.

## Data Availability

The data that support the findings of this study are available from the corresponding author upon reasonable request.
