# Supplementary material for: Targeting PLD3 Reverses the Immunosuppressive Niche by Reprogramming Tumor‐Associated Macrophages and Potentiates Antitumor Immunity
Source: Adv Sci (Weinh). 2026 May 21;13(45):e75730. doi: 10.1002/advs.75730 (PMC13335905; doi:10.1002/advs.75730)
Supplement: Supplementary file 2 [file ADVS-13-e75730-s002.docx]

**Supplementary figure legends**

**Figure S1. PLD3+Macro predicts CRC immunotherapy outcomes, related to Figure 1.**

(A) UMAP embedding of all cell clusters from scRNA-seq data (GSE236581). (B) Stack plot displaying the abundance of all cell clusters based on different immunotherapy responses. (C) Stack plot displaying the abundance of macrophage subclusters based on different immunotherapy responses. (D) Dot plot showing the RNA expression of marker genes used to define the macrophage subclusters. (E-F) HdWGCNA analysis of macrophages to identify the module and core genes associated to macrophage subclusters. (G) Violin plot comparing the expression of TAMs markers (APOE, SPP1, C1QC, TREM2, GPNMB, MS4A4A, APOC1) in macrophage subclusters. (H) Identification of PLD3+Macro marker genes by Venn diagram analysis. (I) Functional annotation of PLD3+Macro marker genes. (J-L) Cell trajectories of macrophages subclusters and antigen presentation-related genes are decisive for macrophage differentiation inferred by CytoTRACE. (M) Pseudotime trajectory analysis of macrophages coloured by MSS/MSI, subcluster and response to immunotherapy inferred by monocle2.

**Figure S2. Spatial infiltration dynamics of PLD3+Macro dictate immunotherapy outcomes in colorectal cancer, related to Figure 1.**

(A) Representative images and statistical analysis of multiplex immunofluorescence for DAPI, PLD3 and CD68 were performed on pre- immunotherapy colonoscopic biopsies (n=24) from CRC patients with differing immunotherapy responses (CR, PR, SD, PD). Scale bar, 50 μm.

**Figure S3. Single-cell sequencing validation of PLD3+Macro in colorectal cancer, related to Figure 1-2.**

(A) UMAP embedding of monocyte/macrophage subclusters, effective CD8^+^ T (effectCD8T), exhausted CD8^+^ T (CD8+Tex) and cancer cell from scRNA-seq dataset (GSE161277). (B) Dot plot showing the RNA expression of marker genes used to define cell subclusters. (C) Correlation analysis of cell subclusters infiltration proportions. (D) Violin plot comparing the expression of TAMs markers (APOE, TREM2, GPNMB, LYVE1 and APOC1) and cluster markers (FCER1A, S100A8 and IL1B) in monocyte/macrophage subclusters. (E) CIBERSORTx analysis of the difference of cell subclusters infiltration proportions between CRC Tumor and adjacent normal tissue. (F) CIBERSORTx analysis of the difference of cell subclusters infiltration proportions among consensus molecular subtype (CMS) classification from CRC Tumor. (G) Representative multiplex immunofluorescence images of DAPI, PLD3, CD68 in the CRC patients with MSS or MSI-H (n=2 per group). Scale bar, 20 μm. (H) Temporal increase or decrease in the expression of markers driving trajectory changes. (I) Pseudotime trajectory analysis of monocyte/macrophage coloured by subcluster inferred by monocle2. (J) Pseudotime trajectory analysis of monocyte/macrophage coloured by subcluster inferred by Slingshot. (K) Dot plot of analyzing the association of M1 signature and M2 signature genes to monocyte/macrophage subclusters. (L) Heatmap of analyzing the association of M1 signature and M2 signature genes to monocyte/macrophage subclusters.

**Figure S4. TME promotes PLD3 expression in macrophages correlating positively with TAMs markers, related to Figure 1-2.**

(A) Volcano of differentially expressed genes (DEGs) between PLD3+Macro High and PLD3+Macro Low Group in CRC tissue from TCGA. (B) Correlation analysis of M1 signature, M2 signature, PLD3 and TAMs markers. (C) Volcano of DEGs between PLD3+Macro (PLD3 expression>0) and PLD3-Macro (PLD3 expression=0) in CRC scRNA-seq data (GSE161277). (D) Hub genes of DEGs expressed higher in PLD3+Macro compared with PLD3-Macro. (E) Representative confocal immunofluorescence images showed PLD3 expression in adjacent normal tissue and CRC Tumor. Scale bar, 100 μm (n=2 per group). (F) Flow cytometry analysis showing the proportion of PLD3+Macro in CRC tumor and adjacent normal tissue (n=3 per group). (G) Flow cytometry analysis of Il10 expression in RAW264.7 under different culture conditions (n=3 per group). (H) Flow cytometry analysis showing the proportion of Pld3+Macro in THP-1 derived macrophages under different culture conditions (n=3 per group). (I) Representative confocal immunofluorescence images of Pld3 in BMDM under different culture conditions (n=3 per group). Scale bar, 5 μm. (J) PLD3 gene expression was significantly upregulated in M2 macrophages in various tumors using the GEPIA2021 online tool (http://gepia2021.cancer-pku.cn/). Data are means ± SEM, and the P values were calculated by unpaired, two-tailed Student’s t test (G-I). *P < 0.05, **P < 0.01, ***P < 0.001, ****P < 0.0001 and ns, not significant.

**Figure S5. Construction of myeloid-specific Pld3 knockout mouse model and scRNA-seq analysis of subcutaneous tumors, related to Figure 3.**

(A) The verification of myeloid specific Pld3-KO in mice by genotyping. (B) Mature BMDM harvested at 7 days. Scale bar, 50 μm. (C) Flow cytometry analysis identifies the differentiation of bone marrow cells into mature Cd11b+F4/80+macrophages. (D-E) RT-qPCR and western blotting detect mRNA and protein expression of Pld3 from Pld3+/+ BMDM (Pld3^flox/flox^) and Pld3-/- BMDM (Pld3^ΔMKO^) (n = 3 per group). Data are means ± SEM, and the P values were calculated by unpaired, two-tailed Student’s t test. *P < 0.05, **P < 0.01, ***P < 0.001, ****P < 0.0001 and ns, not significant. (F) Macroscopic appearance of MC38 orthotopic MSI-H CRC tumors from Pld3^flox/flox^ and Pld3^ΔMKO^ mice (n=3 per group). (G) UMAP embedding showing the expression of cell type markers. (H) UMAP embedding of all cell clusters from scRNA-seq of Pld3^flox/flox^ and Pld3^ΔMKO^ mice. (I) Marker genes of all cell clusters from scRNA-seq of Pld3^flox/flox^ and Pld3^ΔMKO^ mice. (J) Dot plot showing the RNA expression of marker genes used to define all cell clusters. (K) GSVA analysis of all cell clusters, comparing Pld3^flox/flox^ and Pld3^ΔMKO^.

**Figure S6. Construction and functional analysis of RAW264.7 stable Pld3 knockout cell line, related to Figure 3-5.**

(A-C) Validation of Pld3 knockdown efficiency in RAW264.7 cells by fluorescence, RT-qPCR, and western blotting. (D) Cell viability of RAW264.7-shNC and RAW264.7-shPld3 at distinct time points (n = 5 per group). (E) CT26 cells and RAW264.7-shNC/shPld3 cells were mixed at 5:1 ratio and subcutaneously (s.c.) injected into BALB/c mice. Tumors were harvested at 23 days. Flow cytometry analysis showing the proportion of CD8^+^ T and CD4^+^ T cells in tumors from positive control, CT26+RAW264.7-shNC and CT26+RAW264.7-shPLD3 group (n=3 per group). (F) RT-qPCR detects the differential expression of tumor-associated macrophage markers (Trem2, Spp1 and Apoe) (n =3 per group). Data are means ± SEM, and the P values were calculated by unpaired, two-tailed Student’s t test (B, F), two-way ANOVA (D) and one-way ANOVA (E). *P < 0.05, **P < 0.01, ***P < 0.001, ****P < 0.0001 and ns, not significant.

**Figure S7. PLD3 is selectively expressed in macrophages but not neutrophils, related to Figure 3.**

(A) Flow cytometry analysis showing Pld3 expression in tumor-infiltrating macrophages and neutrophils from MC38 subcutaneous tumors. (B) Representative confocal immunofluorescence images of Pld3, F4/80 and Ly6g in MC38 subcutaneous tumors. Scale bar, 100 μm (top) and 50 μm (bottom). (C) Spatial transcriptomics analysis of PLD3, Macrophages markers, Neutrophil markers and their co-localization (GSE226997). (D) PLD3-expressing cell subsets in colorectal cancer were identified using the Single-cell Colorectal Cancer Atlas (https://crc.icbi.at). Myeloid cells are largely devoid of neutrophils due to the very short half-life of neutrophils.

**Figure S8. Single-cell validation reveals PLD3 is selectively expressed in macrophages but not neutrophils, related to Figure 3.**

(A-B) PLD3 is selectively expressed in macrophages but not neutrophils in human scRNA-seq datasets (GSE236581 and GSE161277). (C-D) Pld3 is selectively expressed in macrophages but not neutrophils in murine scRNA-seq datasets from our laboratory.

**Figure S9. The gating strategy for flow cytometry data, related to Figure 2-5.**

(A) Validation of PLD3 antibody for flow cytometry application using isotype control IgG. (B) Flow cytometry gating strategy for PLD3+ macrophages in human CRC tissues. (C) Flow cytometry validation of Cd11b^+^F4/80^+^Ly6g^-^ macrophages differentiation from bone marrow cells in vitro. (D) Flow cytometry gating strategy for Cd8^+^ T cells in vitro. (E-F) Flow cytometry gating strategy for macrophages cells in vitro.

**Figure S10. The gating strategy for flow cytometry data, related to Figure 8 and Figure 10.**

(A-B) Flow cytometry gating strategy for macrophages in subcutaneous tumors from mice treated with PBS control or Abrine. (C-D) Flow cytometry gating strategy for Cd8^+^ T cells in subcutaneous tumors from mice treated with PBS control or Abrine. (E) Flow cytometry gating strategy for macrophages, NK cells and Cd8^+^ T cells in MC38 orthotopic MSI-H CRC tumors.

**Figure S11. Pld3-deficient macrophage restores T cell-mediated antitumor immunity, related to Figure 4.**

(A) Density distribution plots of the nearest spatial distances to PD1+CD8A+ T cells from macrophages (binned by PLD3 expression levels in macrophages) and CD8^+^ T cells. (B-C) Cell communication from macrophage subclusters to exhausted CD8+ T (CD8+Tex) using NicheNet algorithm. (D) Differential communication hubs and weights among various cell types based on interaction number between Pld3^flox/flox^ and Pld3^ΔMKO^ (red arrows indicate upregulated cellular communication in Pld3^ΔMKO^, while blue arrows represent downregulated interactions in Pld3^ΔMKO^). (E) Heatmap showing the differential number and strength of interactions between Pld3^flox/flox^ and Pld3^ΔMKO^ (red indicates upregulated cellular communication in Pld3^ΔMKO^, while blue represents downregulated interactions in Pld3^ΔMKO^). (F) Information flow of cell-cell interactions, comparing Pld3^flox/flox^ and Pld3^ΔMKO^. (G) Number and strength of inferred interactions, comparing Pld3^flox/flox^ and Pld3^ΔMKO^. (H) Dot plot showing the cell-cell interactions of macrophages with NKT cell, comparing Pld3^flox/flox^ and Pld3^ΔMKO^. (I) Flow cytometry analysis showing the proportion of IFNG^+^ CD8^+^ T cells, which were co-cultured with THP-1-NC or THP-1-siPLD3 (n=3 per group). (J) Flow cytometry analysis showing the proportion of Perforin^+^ Cd8^+^ T cells, which were co-cultured with Pld3+/+ BMDM and Pld3-/- BMDM (n=3 per group). (K) Macrophage (RAW264.7) and Cd8^+^ T cells co-culture experimental design. (L) Flow cytometry analysis showing the proportion of Gzmb^+^ and Ifng^+^ Cd8^+^ T cells, which were co-cultured with RAW264.7-shNC or RAW264.7-shPld3 (n=3 per group), RAW264.7 cells were stimulated with CT26-CM for 48 hours to induce TAMs. Data are means ± SEM, and the P values were calculated by unpaired, two-tailed Student’s t test (I-J, L). *P < 0.05, **P < 0.01, ***P < 0.001, ****P < 0.0001 and ns, not significant.

**Figure S12. Pld3-deficient macrophage reduces the anti-inflammatory phenotype of tumor-associated macrophages, related to Figure 5.**

(A) UMAP embedding of macrophage subclusters of subcutaneous tumors from Pld3^flox/flox^ and Pld3^ΔMKO^ mice. (B) Stack plot displaying the abundance of macrophage subclusters. (C) Cell trajectories of macrophages subclusters inferred by CytoTRACE. (D) Dot plot showing the RNA expression of marker genes in macrophage subclusters used to define the function of Proinflammatory, Anti-inflammatory, APC and Chemotaxis. (E) UMAP embedding of the expression of TAMs markers. (F-G) HdWGCNA analysis of macrophage to identify the module and core genes associated to macrophage subclusters. (H) HdWGCNA analysis of macrophage to identify the module and core genes associated to Pld3^flox/flox^ (Macrophage 1). (I) The heatmap from SCENIC analysis showing the gene expression of active transcription factors in macrophage subclusters.

**Figure S13. PLD3 interacts with TAMs markers to drive immunosuppressive macrophage polarization, related to Figure 5.**

(A-B) Representative multiplex immunofluorescence images of DAPI, PLD3, ARG1 and CD163/CD68 in the CRC samples (n=2). Scale bar, 20 μm. (C) IL-4 (40 ng/mL) was used to stimulate M0 macrophages to polarise into the M2-phenotype for 48 hours, and LPS (100 ng/mL) was used to stimulate M0 macrophages to polarise into the M1-phenotype for 24 hours. Western blotting detects the expression of Pld3, TAMs markers (Arg1, Apoe, Mrc1) and pro-inflammatory markers (Nos2) in RAW264.7. (D) RT-qPCR detects the differential expression of proinflammatory and anti-inflammatory markers in Pld3+/+ BMDM and Pld3-/- BMDM (n =3 per group). Data are means ± SEM, and the P values were calculated by unpaired, two-tailed Student’s t test. *P < 0.05, **P < 0.01, ***P < 0.001, ****P < 0.0001 and ns, not significant. (E) Co-immunoprecipitation / Mass spectrometry (COIP/MS) detecting the proteins interact with Pld3 in RAW264.7. (F) Co-immunoprecipitation and western blotting verify the interaction between Pld3 and Arg1. (G) Alphafold3 predicts PLD3 protein interaction with TAMs markers.

**Figure S14. PLD3 drives cellular senescence, exerts pro-tumorigenic immunosuppressive capacity by orchestrating the lysosomal-AKT-NF-κB signaling axis, related to Figure 6.**

(A) GO (Biological Process, Molecular Function and Cellular Component) and KEGG analysis revealed that the p53 signaling pathway and Cellular senescence signalling pathway were significantly enriched in Pld3+/+ BMDM. (B) KEGG analysis of the module and core genes associated to macrophage subclusters from Pld3^flox/flox^ and Pld3^ΔMKO^ mice by hdWGCNA. (C) GSEA analysis revealed that the Lysosome pathway was significantly enriched in PLD3+Macro compared to PLD3-Macro (GSE161277). (D) GO (Biological Process, Molecular Function and Cellular Component) and KEGG analysis of the macrophage subclusters of subcutaneous tumors from Pld3^flox/flox^ and Pld3^ΔMKO^ mice. (E) Spatial colocalization of PLD3 and LAMP2 correlates with activation of P53 Pathway, PI3K_AKT_MTOR Pathway, and TNFA_via_NFκB pathways (GSE226997).

**Figure S15. Localization of Pld3 in macrophages and its relationship with Lamp2, and effect of Pld3 on senescent macrophages, related to Figure 6-7.**

(A) Representative confocal immunofluorescence images analysis of Pld3 and Lamp2 co-localization in BMDM (Model diagram). Scale bar, 5 μm. (B) Representative confocal immunofluorescence images analysis of Pld3 and Lamp2 co-localization in BMDM. Scale bar, 5 μm. (C) Nuclear-cytoplasmic fractionation assay and western blotting verify Pld3 localization in the cytoplasm of BMDM. (D) RT-qPCR detects the expression of Pld3 of BMDM from young (12-16 weeks) and senescence (over 60 weeks) mice (n=3 per group). (E) RT-qPCR detects the differential expression of senescence-associated markers (P16, Tp53). BMDMs were stimulated with MC38-CM for 24 hours to induce TAMs (n=3 per group).

**Figure S16. Abrine enhances antitumor responses by targeting PLD3 in tumor-associated macrophages, related to Figure 8.**

(A) Molecular structural formula of Abrine. (B) Molecular docking of Abrine with PLD3 using Autodock and the docking site. (C) Suppression rate and toxicity of Abrine at different concentrations on RAW264.7 cell proliferation for 24 hours and 48 hours. (D) Western blotting detects the expression of Pld3, Nos2, Apoe and Arg1 of RAW264.7 stimulated with Abrine (10 μM or 40 μM) for 48 hours. (E) Dot plot of macrophages in subcutaneous tumors from Pld3^flox/flox^, Pld3^ΔMKO^ and Pld3^flox/flox^+Abrine mice, showing the RNA expression of Pld family (Pld1, Pld2, Pld3, Pld4) and Ido family (Ido1, Ido2). (F) RT-qPCR detects Pld family (Pld1, Pld2, Pld3, Pld4) and Ido1 of BMDM under different conditions (n = 3 per group). BMDM was additionally treated with Abrine (40 μM) in BMDM+Abrine group and treated with IDO1-IN-18 (10 μM) in BMDM+IDO1-IN-18 group for 48 hours. BMDMs were stimulated with MC38-CM for 24 hours to induce TAMs (BMDM-CM). (G) Representative immunohistochemical staining for Ki67 in subcutaneous tumors from PBS control and Abrine Treatment (n=3 per group). Scale bar, 20 μm. (H) RT-qPCR detects RNA expression level of Pld3 and polarization markers (Arg1, Nos2, p65 and Apoe) of subcutaneous tumors from mice treated with or without Abrine (n = 16 per group). (I-J) Livers from mice treated with or without Abrine and representative images of hematoxylin-eosin staining are shown (n = 7 per group). Scale bar, 100 μm. (K-L) Kidneys from mice treated with or without Abrine and representative images of hematoxylin-eosin staining are shown (n = 7 per group). Scale bar, 100 μm. (M-N) Spleens from mice treated with or without Abrine and representative images of hematoxylin-eosin staining are shown (n = 7 per group). Scale bar, 100 μm.

**Figure S17. Abrine-mediated modulation of immune cells in the tumor microenvironment, related to Figure 9.**

(A-B) UMAP embedding showing Pld3 expression in all cell clusters of subcutaneous tumors from Pld3^flox/flox^, Pld3^ΔMKO^ and Pld3^flox/flox^+Abrine mice. (C) Dot plot showing the RNA expression of Pld3 in macrophages of subcutaneous tumors from Pld3^flox/flox^, Pld3^ΔMKO^ and Pld3^flox/flox^+Abrine mice. (D) Information flow of cell-cell interactions, comparing Pld3^flox/flox^, Pld3^ΔMKO^ and Pld3^flox/flox^+Abrine. (E) Heatmap showing the differential number and strength of interactions between Pld3^ΔMKO^ and Pld3^flox/flox^+Abrine (red indicates upregulated cellular communication in Pld3^flox/flox^+Abrine, while blue represents downregulated interactions in Pld3^flox/flox^+Abrine). (F) Dot plot showing the cell-cell interactions from macrophages to NKT and T cell, comparing Pld3^flox/flox^, Pld3^ΔMKO^ and Pld3^flox/flox^+Abrine. (G) Dot plot showing the cell-cell interactions from NKT to Macrophage and Malignant cells, comparing Pld3^flox/flox^, Pld3^ΔMKO^ and Pld3^flox/flox^+Abrine. (H) Dot plot showing the cell-cell interactions from T cell to Macrophage and Malignant cells, comparing Pld3^flox/flox^, Pld3^ΔMKO^ and Pld3^flox/flox^+Abrine. (I) UMAP embedding of macrophage subclusters of subcutaneous tumors from Pld3^flox/flox^, Pld3^ΔMKO^ and Pld3^flox/flox^+Abrine mice. (J) Stack plot displaying the abundance of macrophage subclusters. (K) The differentiation plasticity of macrophages from Pld3^flox/flox^, Pld3^ΔMKO^ and Pld3^flox/flox^+Abrine mice inferred by CytoTRACE. (L) GO (Biological Process) and KEGG analysis of the macrophage subclusters from Pld3^flox/flox^, Pld3^ΔMKO^ and Pld3^flox/flox^ + Abrine mice. (M) GSVA analysis of all cell clusters among Pld3^flox/flox^, Pld3^ΔMKO^ and Pld3^flox/flox^+Abrine.

**Figure S18. Targeting PLD3 improves the therapeutic benefit of anti-PD-1 immunotherapy to enhance antitumor efficacy, related to Figure 10.**

(A) Tumor of MC38 tumor-bearing mice of eight groups were harvested at day 29 after MC38 inoculation (n = 6 per group). (B) Tumor weight of MC38 subcutaneous tumors of eight groups. (C-D) Colons images and weight(g) of eight groups. (E) Livers images of eight groups. (F) Spleens images of eight groups. (G) Kidneys images of eight groups.

(H) Livers, Kidneys and Spleens weight(g) of eight groups. (I) Representative images of hematoxylin-eosin staining for Liver, Kidney and Spleen across eight groups. Scale bar, 100 μm.

**Figure S19. Effect of Abrine and PD-1 inhibitors on Cd8^+^ T cell infiltration and tumor growth, related to Figure 10.**

(A) Representative images of immunohistochemical staining for Cd8a in subcutaneous tumors of eight groups. Scale bar, 20 μm. (B) Representative images of immunohistochemical staining for Ki67 in subcutaneous tumors of eight groups. Scale bar, 20 μm. (C-D) Macroscopic appearance of CMT93 MSS orthotopic CRC tumors for each indicated treatment (n=3 per group). (E) Tumor Volume. (F) Tumor weight. Data are means ± SEM, and the P values were calculated by one-way ANOVA (E-F). *P < 0.05, **P < 0.01, ***P < 0.001, ****P < 0.0001 and ns, not significant.

**Supplementary Table**

**Supplementary Table 1. Clinical characteristics of immunotherapy patients**

| **Name** | **Gender** | **Age** | **MSI/MSS** | **Treatment** | **TNM** | **Metastasis** | **Response** | | |
| --- | --- | --- | --- | --- | --- | --- | --- | --- | --- |
| Patient 1 | Male | 70 | MSI-H | ‌Pembrolizumab, 9 cycles | T3N1M1 | Liver | | CR |  |
| Patient 2 | Male | 46 | MSI-H | Pembrolizumab, 5 cycles | T3N1M0 | None | | CR |  |
| Patient 3 | Male | 69 | MSI-H | Pembrolizumab, 3 cycles | T2N0M0 | None | | CR |  |
| Patient 4 | Male | 43 | MSI-H | Pembrolizumab, 5 cycles | T4N2M0 | None | | CR |  |
| Patient 5 | Male | 45 | MSI-H | Tislelizumab+CAPEOX, 4 cycles | T4N2M0 | None | | CR |  |
| Patient 6 | Male | 38 | MSI-H | Tislelizumab, 3 cycles | T4N1M1 | Bladder, Prostate | | CR |  |
| Patient 7 | Female | 31 | MSI-H | Tislelizumab+CAPEOX, 12 cycles | T3N1M0 | None | | CR |  |
| Patient 8 | Male | 52 | MSI-H | Pembrolizumab, 4 cycles | T3N0M0 | None | | CR |  |
| Patient 9 | Male | 53 | MSI-H | ‌Toripalimab+CAPEOX, 3 cycles | T3N1M0 | None | | PR |  |
| Patient 10 | Male | 50 | MSI-H | Toripalimab+CAPEOX, 2 cycles | T4N2M1 | Peritoneum | | PR |  |
| Patient 11 | Male | 34 | MSI-H | Toripalimab, 2 cycles | T3N2M0 | None | | PR |  |
| Patient 12 | Male | 47 | MSI-H | Toripalimab, 5 cycles | T3N0M0 | None | | PR |  |
| Patient 13 | Female | 28 | MSI-H | Toripalimab, 2 cycles;  Tislelizumab, 7 cycles | TisN0M0 | None | | PR |  |
| Patient 14 | Male | 55 | MSI-H | Tislelizumab, 6 cycles | T3N1M0 | None | | PR |  |
| Patient 15 | Male | 59 | MSI-H | Pembrolizumab, 6 cycles | T4N2M0 | None | | PR |  |
| Patient 16 | Male | 66 | MSI-H | ‌Toripalimab, 4 cycles | T4N0M1 | Pancreas | | SD |  |
| Patient 17 | Male | 68 | MSI-H | Tislelizumab+CAPEOX, 3 cycles | T4N1M0 | None | | SD |  |
| Patient 18 | Female | 46 | MSI-H | Tislelizumab+CAPEOX+ Bevacizumab, 6 cycles | T4N1M0 | None | | SD |  |
| Patient 19 | Male | 36 | MSI-H | Toripalimab, 9 cycles | T4N0M0 | None | | SD |  |
| Patient 20 | Female | 53 | MSI-H | FOLFOXIRI+Bevacizumab, 4 cycles  Tislelizumab, 3 cycles | T4NxM0 | None | | SD |  |
| Patient 21 | Male | 37 | MSI-H | CAPEOX+Bevacizumab, 6 cycles  Toripalimab, 15 cycles | TxNxM1 | Peritoneum | | SD |  |
| Patient 22 | Male | 60 | MSI-H | Toripalimab, 5 cycles | T4N1M0 | None | | SD |  |
| Patient 23 | Male | 55 | MSI-H | Toripalimab+CAPEOX, 8 cycles  Cadonilimab‌, 8 cycles  Cadonilimab+ Bevacizumab, 6 cycles | T4N2M1 | Peritoneum, Bladder, ‌Small intestine | | PD |  |
| Patient 24 | Male | 51 | MSI-H | CAPEOX+Bevacizumab+Toripalimab, 10 cycles | T4N1M1 | Bladder | | PD |  |
| **CAPEOX**: Capecitabine+Oxaliplatin; **FOLFOXIRI**: Leucovorin/Folinic acid+Oxaliplatin+Irinotecan; **CR**: Complete Response; **PR**: Partial Response; **SD**: Stable Disease; **PD**: Progressive Disease | | | | | | | | |  |

**Supplementary Table 2. Molecular operating environment (MOE) docking**

|  | **mol** | **rseq** | **mseq** | **S** | **rmsd_ refine** | **E_conf** | **E_place** | **E_score1** | **E_refine** | **E_score2** |
| --- | --- | --- | --- | --- | --- | --- | --- | --- | --- | --- |
| 1 | 160511 | 1 | 1 | -5.3251 | 0.9962 | 23.5172 | -49.6577 | -7.2751 | -24.4005 | -5.3251 |
| 2 | 160511 | 1 | 1 | -5.1656 | 2.4890 | 24.3269 | -43.6601 | -8.0962 | -24.4435 | -5.1656 |
| 3 | 160511 | 1 | 1 | -4.9063 | 2.0901 | 28.9077 | -34.7994 | -6.7017 | -24.7546 | -4.9063 |
| 4 | 160511 | 1 | 1 | -4.8837 | 2.8928 | 19.9452 | -41.8036 | -7.5751 | -23.2744 | -4.8837 |
| 5 | 160511 | 1 | 1 | -4.7724 | 1.7271 | 27.2945 | -43.7923 | -6.7918 | -17.9824 | -4.7724 |

**Supplementary Table 3. Comparative analysis of liver injury (Control vs Abrine)**

| **Control** | | **Abrine** | |
| --- | --- | --- | --- |
| **Sample** | **Description** | **Sample** | **Description** |
| 1 | Mild congestion with focal thrombosis, hepatocellular architecture mostly normal | 1 | Mild congestion and focal inflammatory infiltration |
| 2 | Mostly normal | 2 | Mild congestion and focal inflammatory infiltration |
| 3 | Mostly normal | 3 | Mild congestion |
| 4 | Mild congestion with focal thrombosis, hepatocellular architecture mostly normal | 4 | Smaller hepatocytes with condensed nuclear chromatin; slight sinusoidal dilation; mild microvesicular steatosis |
| Summary | Mostly normal | Summary | Mild injury |

**Supplementary Table 4. Comparative analysis of kidney injury (Control vs Abrine)**

| **Control** | | **Abrine** | |
| --- | --- | --- | --- |
| **Sample** | **Description** | **Sample** | **Description** |
| 1 | Mild congestion; renal parenchyma mostly normal | 1 | Mild congestion; renal parenchyma mostly normal |
| 2 | Mostly normal | 2 | Mild congestion; renal parenchyma mostly normal |
| 3 | Mild congestion; renal parenchyma mostly normal | 3 | Mild congestion; renal parenchyma mostly normal |
| 4 | Mild congestion; renal parenchyma mostly normal | 4 | Mild congestion; renal parenchyma mostly normal |
| Summary | Mostly normal | Summary | Mostly normal |

**Supplementary Table 5. Comparative analysis of spleen injury (Control vs Abrine)**

| **Control** | | **Abrine** | |
| --- | --- | --- | --- |
| **tissue** | **Description** | **tissue** | **Description** |
| 1 | Mostly normal | 1 | Congestion; focal hemosiderin deposition; focal mild fibrosis |
| 2 | White pulp expansion; lymphoid hyperplasia; focal hemosiderin deposition | 2 | Focal apoptotic bodies; hemosiderin deposition |
| 3 | Mostly normal | 3 | Focal apoptotic bodies; hemosiderin deposition; focal mild fibrosis |
| 4 | Mostly normal | 4 | Congestion; focal hemosiderin deposition; focal apoptotic bodies |
| Summary | Mostly normal | Summary | Mild injury |

**Supplementary Table 6. Comparative analysis of liver injury score (Abrine and/or anti-PD-1 treament)**

| **Group** | **Structure of hepatic lobule** | **Inflammation and focal necrosis in hepatic lobules** | **Inflammation of the manifold area** | **Infiltration of inflammatory cells in and around the central vein** | **Total Score** |
| --- | --- | --- | --- | --- | --- |
| Pld3^flox/flox^ | 0 | 0 | 0 | 0 | 0 |
| Pld3^ΔMKO^ | 0 | 0 | 0 | 0 | 0 |
| Pld3^flox/flox^ + αPD-1 | 0 | 1 | 1 | 1 | 3 |
| Pld3^ΔMKO^ + αPD-1 | 0 | 0 | 0 | 0 | 0 |
| Pld3^flox/flox^ + Abrine | 0 | 2 | 0 | 0 | 2 |
| Pld3^ΔMKO^ + Abrine | 0 | 0 | 0 | 0 | 0 |
| Pld3^flox/flox^ + Abrine  +αPD-1 | 0 | 1 | 1 | 1 | 3 |
| Pld3^ΔMKO^ + Abrine  +αPD-1 | 0 | 1 | 0 | 0 | 1 |

**Supplementary Table 7. Comparative analysis of kidney injury score (Abrine and/or anti-PD-1 treament)**

| **Group** | **Degree of glomerular dilatation** | **The number of cells in the glomerulus increased** | **Total Score** |
| --- | --- | --- | --- |
| Pld3^flox/flox^ | 0 | 0 | 0 |
| Pld3^ΔMKO^ | 1 | 0 | 1 |
| Pld3^flox/flox^ + αPD-1 | 0 | 0 | 0 |
| Pld3^ΔMKO^ + αPD-1 | 0 | 0 | 0 |
| Pld3^flox/flox^ + Abrine | 0 | 0 | 0 |
| Pld3^ΔMKO^ + Abrine | 0 | 0 | 0 |
| Pld3^flox/flox^ + Abrine  +αPD-1 | 0 | 0 | 0 |
| Pld3^ΔMKO^ + Abrine  +αPD-1 | 0 | 0 | 0 |

**Supplementary Table 8. Comparative analysis of spleen injury score (Abrine and/or anti-PD-1 treament)**

| **Group** | **Infiltration of inflammatory cells** | **Degree of white pulp atrophy and red pulp dilatation** | **Congestion** | **Degree of splenomegaly** | **Total Score** |
| --- | --- | --- | --- | --- | --- |
| Pld3^flox/flox^ | 0 | 0 | 1 | 0 | 1 |
| Pld3^ΔMKO^ | 0 | 0 | 0 | 0 | 0 |
| Pld3^flox/flox^ + αPD-1 | 0 | 0 | 0 | 0 | 0 |
| Pld3^ΔMKO^ + αPD-1 | 0 | 0 | 0 | 0 | 0 |
| Pld3^flox/flox^ + Abrine | 0 | 0 | 0 | 0 | 0 |
| Pld3^ΔMKO^ + Abrine | 0 | 0 | 0 | 0 | 0 |
| Pld3^flox/flox^ + Abrine  +αPD-1 | 0 | 0 | 0 | 0 | 0 |
| Pld3^ΔMKO^ + Abrine  +αPD-1 | 0 | 0 | 1 | 0 | 1 |

**Supplementary Table 9. Primers for RT-qPCR**

| **Gene** | **Species** | **Forward Primer** | **Reverse Primer** |
| --- | --- | --- | --- |
| Pld3 | Mouse | TTCTATGGGAATACGGGGACTT | CGTGGTGGCATTGGGAAACT |
| Pld3-Exon10-12 | Mouse | CGGTCCTTTGACACCCGATA | ACCGCATCGATGGATCAGAG |
| Gapdh | Mouse | TGACCTCAACTACATGGTCTACA | CTTCCCATTCTCGGCCTTG |
| β-actin | Mouse | ACTATTGGCAACGAGCGGTT | GGTGTAAAACGCAGCTCAGTAA |
| Trem2 | Mouse | CTGGAACCGTCACCATCACTC | CGAAACTCGATGACTCCTCGG |
| Spp1 | Mouse | AGCAAGAAACTCTTCCAAGCAA | GTGAGATTCGTCAGATTCATCCG |
| Apoe | Mouse | GACCCAGCAAATACGCCTG | CATGTCTTCCACTATTGGCTCG |
| Nos2 | Mouse | ACATCGACCCGTCCACAGTAT | CAGAGGGGTAGGCTTGTCTC |
| Arg1 | Mouse | GTGAAGAACCCACGGTCTGT | CTGGTTGTCAGGGGAGTGTT |
| p65 | Mouse | AGGCTTCTGGGCCTTATGTG | TGCTTCTCTCGCCAGGAATAC |
| Tp53 | Mouse | GTCACAGCACATGACGGAGG | TCTTCCAGATGCTCGGGATAC |
| P16 | Mouse | CGTACCCCGATTCAGGTGAT | TTGAGCAGAAGAGCTGCTACGT |
| Il6 | Mouse | TAGTCCTTCCTACCCCAATTTCC | TTGGTCCTTAGCCACTCCTTC |
| Il1α | Mouse | CGTGTTGCTGAAGGAGTTGC | AGGTCGGTCTCACTACCTGT |
| Il1β | Mouse | GCAACTGTTCCTGAACTCAACT | ATCTTTTGGGGTCCGTCAACT |
| Tnf | Mouse | GTTCTATGGCCCAGACCCTCAC | GGCACCACTAGTTGGTTGTCTTTG |
| Tgfb1 | Mouse | CTCCCGTGGCTTCTAGTGC | GCCTTAGTTTGGACAGGATCTG |
| Pld1 | Mouse | CCCAACCTTCCGTAGTGATAG | TGGCGTAGAGATGGCAAGAC |
| Pld2 | Mouse | ATCCTGAAGGCTCACGAACAGG | GAGAGAAGAAAGGGTGAAGGAGG |
| Pld4 | Mouse | TGGCCAAGACATCCACTGAC | ACCAAGTTCCTTCACCTGAGTC |
| Ido1 | Mouse | CACGAGGCTGGCAAAGAATC | GACTGGGGGAGCTGACTCTA |

**Supplementary Table 10. Primers for PCR**

| **Gene** | **Species** | **Forward Primer** | **Reverse Primer** |
| --- | --- | --- | --- |
| Pld3-Flox | Mouse | TTTAGGAGTCCGAGGCCAGA | TCCTTTCTCCGTCCCTCAGT |
| Lyz2-Cre-WT | Mouse | TTACAGTCGGCCAGGCTGAC | CTTGGGCTGCCAGAATTTCTC |
| Lyz2-Cre-Mut | Mouse | CCCAGAAATGCCAGATTACG | CTTGGGCTGCCAGAATTTCTC |

**Supplementary Table 11. Key Antibody Table**

| **Antibodies** | **SOURCE** | **IDENTIFIER** |
| --- | --- | --- |
| Anti-mouse/human CD68 antibody | Affinity | DF7518 |
| Anti-mouse/human PLD3 antibody | Affinity | DF9753 |
| Anti-mouse/human APOE antibody | Proteintech | 66830-1-Ig |
| Anti-mouse/human IκBα antibody | ABclonal | A19714 |
| Anti-mouse/human Phospho-IκBα-S36 antibody | ABclonal | AP0999 |
| Anti-mouse/human NF-kB p65/Rel Aantibody | ABclonal | A22331 |
| Anti-mouse/human Phospho-NF-kB p65/RelA-S536 antibody | ABclonal | AP1294 |
| Anti-mouse/human Ki67 antibody | Bioss | bsm-52455R |
| Anti-mouse/human CD107b/LAMP2 antibody | Proteintech | 66301-1-Ig |
| Anti-mouse/human Osteopontin antibody | Proteintech | 25715-1-AP |
| Anti-mouse/human CXCL9 antibody | Bioss | bs-2551R |
| Anti-mouse/human AKT1/2/3 antibody | HUABIO | ET1609-51 |
| Anti-mouse/human Phospho-AKT (S473) antibody | HUABIO | ET1607-73 |
| Anti-mouse/human p53 antibody | HUABIO | HA601315 |
| Anti-mouse/human Phospho-p53 (S392) antibody | HUABIO | ET1606-24 |
| Anti-mouse/human MRC1 antibody | ImmunoWay | YT5640 |
| Anti-mouse/human iNOS antibody | Proteintech | 22226-1-AP |
| Anti-mouse/human ARG1 antibody | Proteintech | 16001-1-AP |
| Anti-mouse/human α-tubulin antibody | Beijing Ray | MG2007 |
| Anti-mouse/human Histone H3 antibody | Proteintech | 17168-1-AP |
| Anti-mouse/human CD163 antibody | ABclonal | A8383 |
| Anti-mouse CD8α antibody | CST | 98941 |
| Anti-mouse CDKN2A/p16INK4a antibody | Abiowell | AWA13166 |
| Anti-mouse F4/80 antibody | HUABIO | RT1212 |
| Anti-mouse Ly6g antibody | HUABIO | IRS167RB |
| Rabbit IgG control Polyclonal antibody | Proteintech | 30000-0-AP |
| Fixable Viability Stain 700 | BD | 564997 |
| BV421 anti-mouse TNF antibody | BD | 563387 |
| PE/Cyanine7 anti-mouse CD366 (Tim-3) antibody | Biolegend | 119715 |
| BV785 anti-mouse PD1 antibody | Biolegend | 135225 |
| APC anti-mouse IFNg antibody | BD | 562018 |
| PE anti-mouse Ly6G antibody | Selleck | G4427 |
| FITC anti-Mouse Perforin antibody | Elabscience | E-AB-F1294C |
| PE594 anti-mouse/human Gzmb antibody | Biolegend | 372215 |
| PE anti-mouse CD80 antibody | invitrogen | 12-0801-81 |
| FITC anti-mouse F4/80 antibody | Biolegend | 157309 |
| BV605 anti-mouse CD11b antibody | Biolegend | 101237 |
| PE-Cy7 anti-mouse CD86 antibody | BD | 560582 |
| BV421 anti-mouse CD206 antibody | Biolegend | 141717 |
| PE anti-mouse CD8a antibody | BD | 553033 |
| PE-Cy7 anti-mouse IL10 antibody | Biolegend | 505025 |
| BV711 anti-mouse CD25 antibody | Biolegend | 102049 |
| BV421 anti-humam CD68 antibody | BD | 564943 |
| APC anti-human CD3 antibody | BD | 570896 |
| PE-Cy7 anti-human TNF antibody | BD | 560678 |
| PerCP-Cy5.5 anti-mouse/human CD11B antibody | Selleck | G4670 |
| BV421 anti-humam IFNG antibody | BD | 564791 |
| PE anti-human PD1 antibody | BD | 573394 |
| PE anti-human IL10 antibody | BD | 562035 |
| APC anti-human CD86 antibody | BD | 560956 |
| FITC anti-human CD206 antibody | BD | 551135 |
| Percp anti-mouse CD45 antibody | Biolegend | 103129 |
| APC anti-mouse NK1.1 (Klrb1c) antibody | Biolegend | 156505 |
| Anti-mouse PD-1(CD279)-InVivo | Selleck | RMP1-14 |

**Supplementary Table 12. Key Chemicals Table**

| **Chemicals** | **SOURCE** | **IDENTIFIER** |
| --- | --- | --- |
| Actin-Tracker Red-594 | Beyotime | C2205S |
| L-(+)-Abrine | MedChemExpress | HY-N1436 |
| IDO1-IN-18 | MedChemExpress | HY-144651 |
| Recombinant Murine IL-4 | Beyotime | 214-14-5UG |
| Lipopolysaccharide, LPS | Sigma-Aldrich | L2880 |
| Dylight 488, Goat Anti-Rabbit IgG | Abbkine | A23220 |
| Goat Anti-Rabbit IgG (H+L) Fluor647 | Affinity | S0013 |
| SC79 | GLPBIO | GC11645 |
| Opal 7-Color Manual IHC Kit | AKOYA | NEL811001KT |
| D-Luciferin | Goldbio | LUCK-1g |
| Anti-Mouse CD3 | BioGems | Clone:17A2 |
| Anti-Mouse CD28 | BioGems | Clone:37.51 |
| CollagenaseⅣ | Solarbio | C8160 |
| Hyaluronidase | Solarbio | H8030 |
| DNase I | Solarbio | D8071 |
| Protein A/G PLUS-Agarose | Santa | sc-2003 |
| ChamQ Universal SYBR qPCR Master Mix | Vazyme | Q711-02 |

**Supplementary Table 13. Key Critical commercial assays Table**

| **Critical commercial assays** | **SOURCE** | **IDENTIFIER** |
| --- | --- | --- |
| One Step Mouse Genotyping Kit | Vazyme | PD101-01 |
| Mouse CD8^+^T Cell Isolation Kit | Vazyme | CS103-01 |
| Cell Counting Kit-8(CCK-8) | NCM Biotech | C6005 |
| Senescence-Associated β-Galactosidase (SA-β-Gal) Staining Kit | Solarbio | G1580 |
| Mouse/Rabbit Polymer Detection IHC System | Zhongshan Golden Bridge | PV-6000 |
| SevenFast Total RNA Extraction Kit | Seven Innovation Biotechnology | SM130-02 |

**Supplementary Table 14. Cell Lines**

| **Cell lines** | **SOURCE** |
| --- | --- |
| MC38 | EallBio |
| MC38-Luc | the Department of Pathology, Southern Medical University |
| CT26 | the Department of Pathology, Southern Medical University |
| HEK-293T | the Department of Pathology, Southern Medical University |
| L929 | the Department of Pathology, Southern Medical University |
| RAW264.7 | the Department of Pathology, Southern Medical University |
| THP-1 | the Department of Pathology, Southern Medical University |
| Jurkat T | the Department of Pathology, Southern Medical University |
| SW480 | the Department of Pathology, Southern Medical University |
| CMT93 | the Department of Pathology, Southern Medical University |

**Supplementary Table 15. Organisms/strains**

| **Organisms/strains** | **SOURCE** |
| --- | --- |
| Mouse:C57BL/6J | Laboratory Animal Center, Southern Medical University |
| Mouse: BALB/c | Laboratory Animal Center, Southern Medical University |
| Pld3 fl/+ mouse | Shulaibao (Wuhan) Biotechnology |
| Lyz2 Cre+/- mouse | Shulaibao (Wuhan) Biotechnology |

**Supplementary Table 16. Oligonucleotides and sequence**

| **Oligonucleotides** | **Sequence** | **Vector** | **SOURCE** |
| --- | --- | --- | --- |
| shRNA targeting Pld3 (mouse) | CCTTGAATGAAATCGAGGCAT | pLKO.1-CMV-copGFP-PURO | Tsingke Biotechnology |
| siRNA targeting PLD3 (human) | UUCUGCAUGUCCACCAUGC | / | Tsingke Biotechnology |
| Pld3^fl/+^ mouse targeting Pld3 | ENSMUSG00000003363 Pld3-Exon 10-11 | / | Shulaibao (Wuhan) Biotechnology |

**supplemental PLD3+Macro Markers.csv**

**supplemental RNA seq_fpkm.csv**

**supplemental 4D-DIA Protein Seq.csv**

**supplemental CoIP-MS.csv**

**supplemental Vedio1 (Pld3-Lamp2).mp4**
