# Supplementary material for: Targeting PLD3 Reverses the Immunosuppressive Niche by Reprogramming Tumor‐Associated Macrophages and Potentiates Antitumor Immunity
Source: Adv Sci (Weinh). 2026 May 21:e75730. Online ahead of print. doi: 10.1002/advs.75730 (PMC13335905; doi:10.1002/advs.75730)

Figure 1G

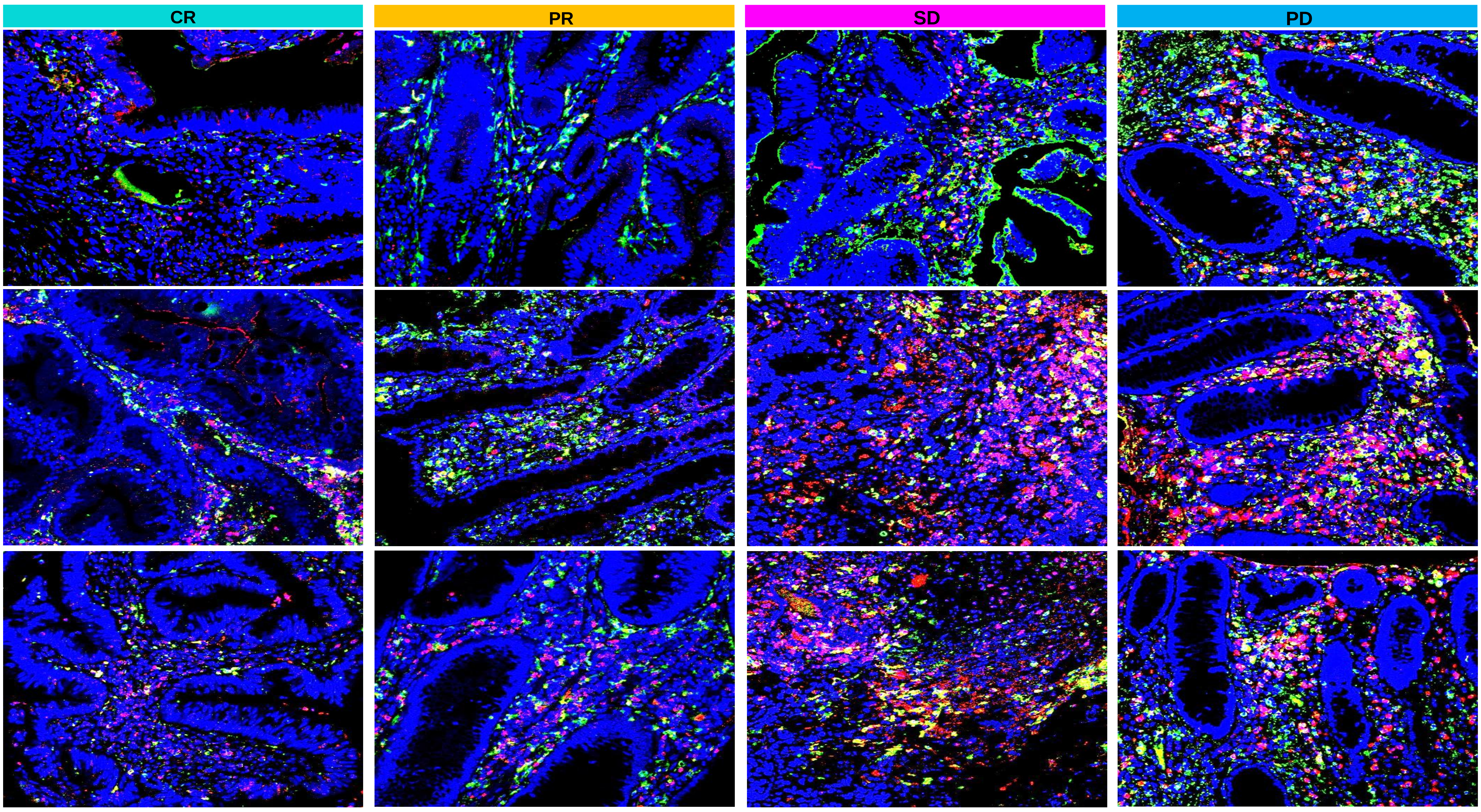

Figure 2A

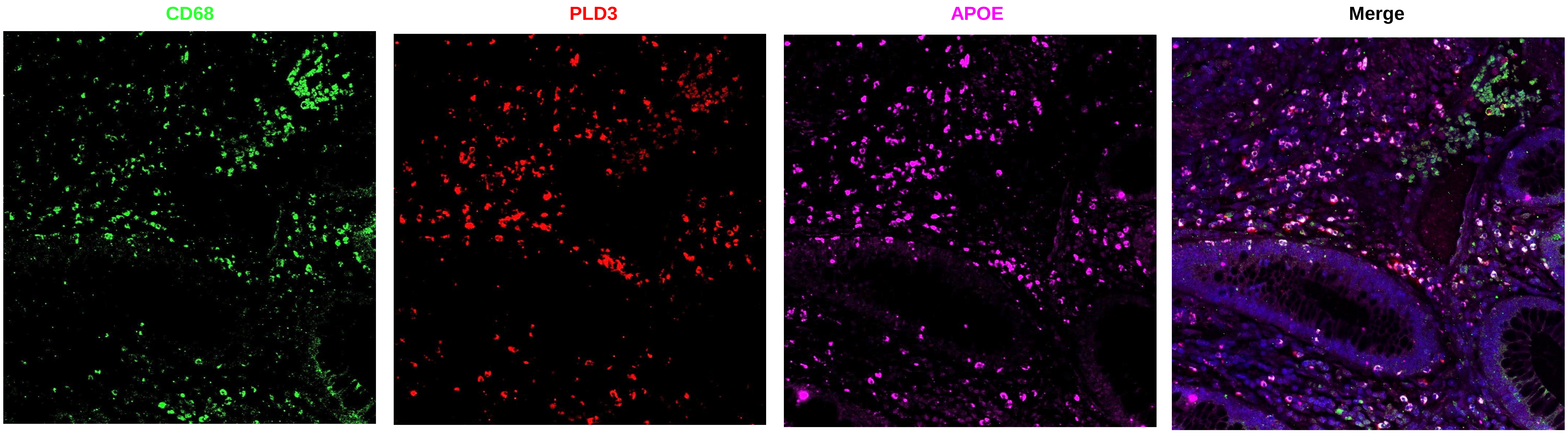

Figure 2E

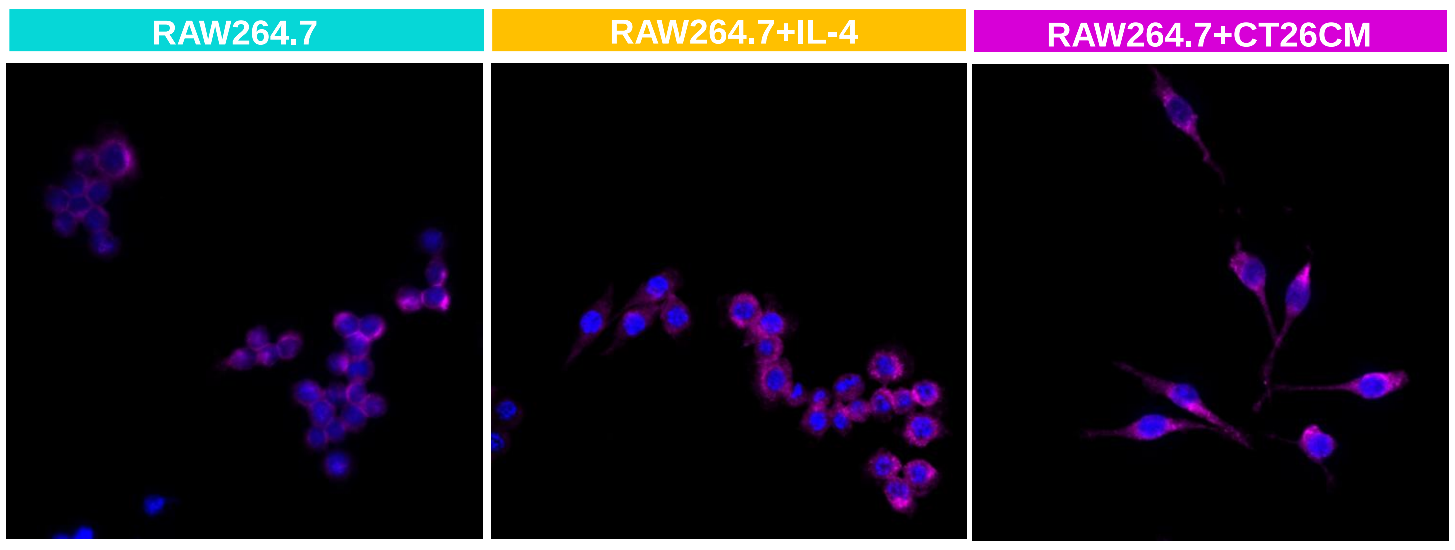

Figure 2F

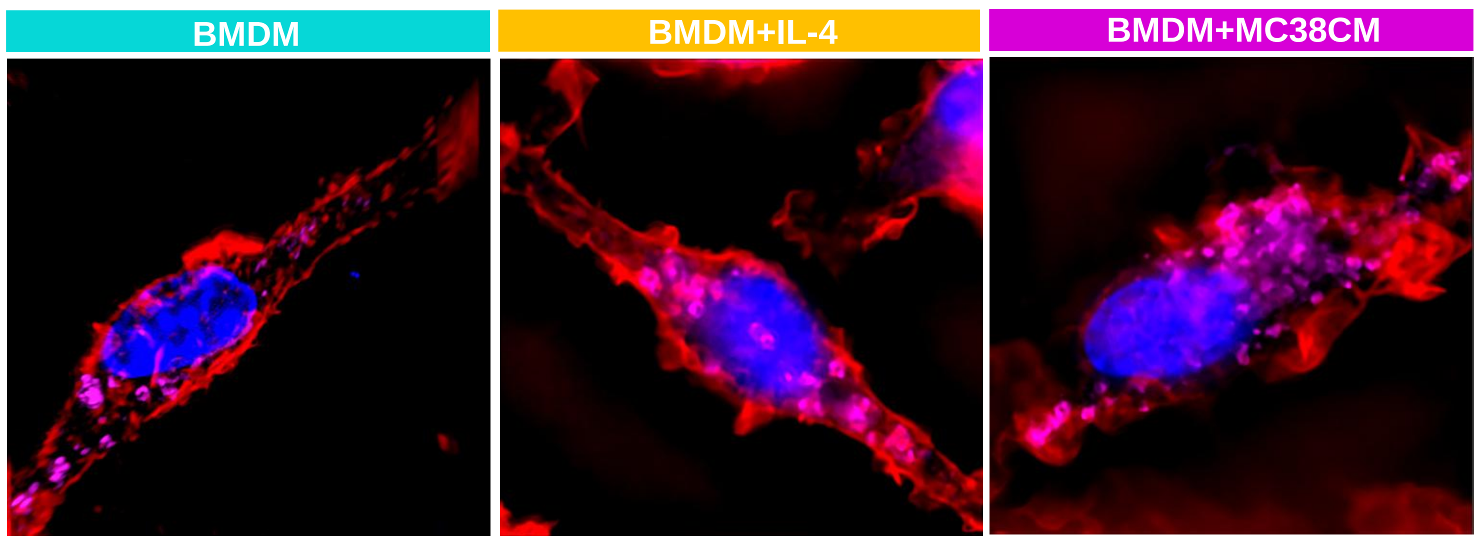

Figure 3B

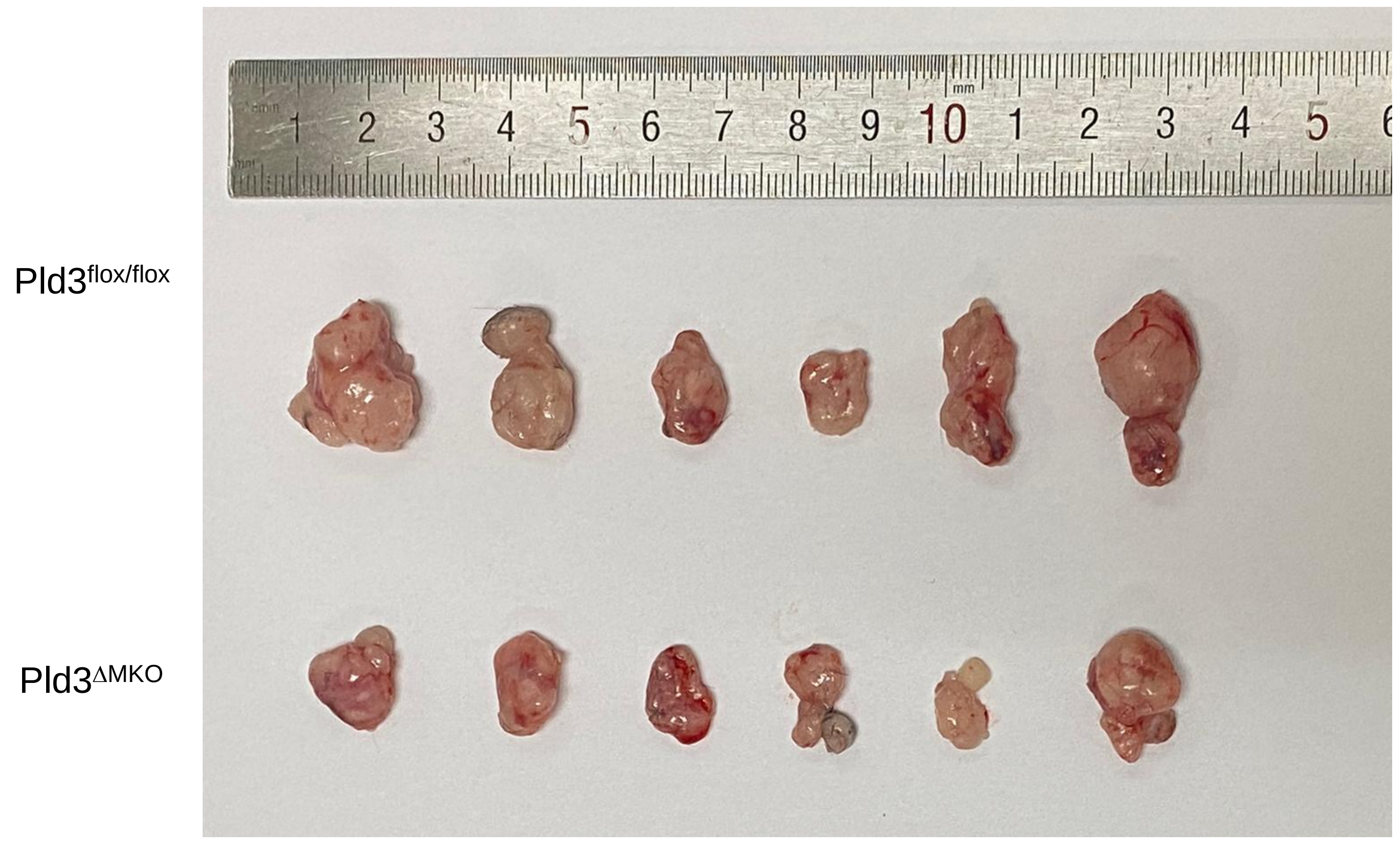

Figure 3L

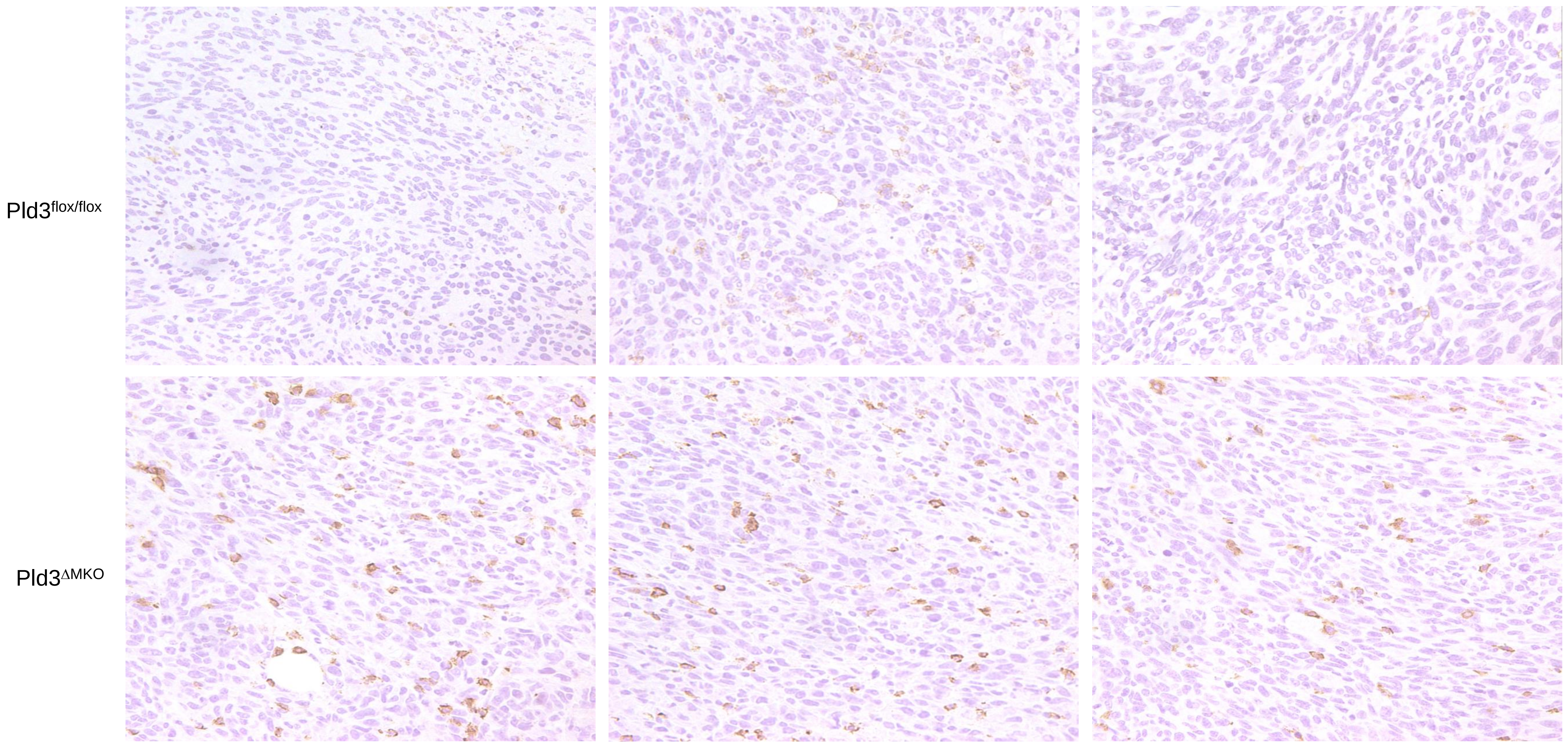

Figure 4C

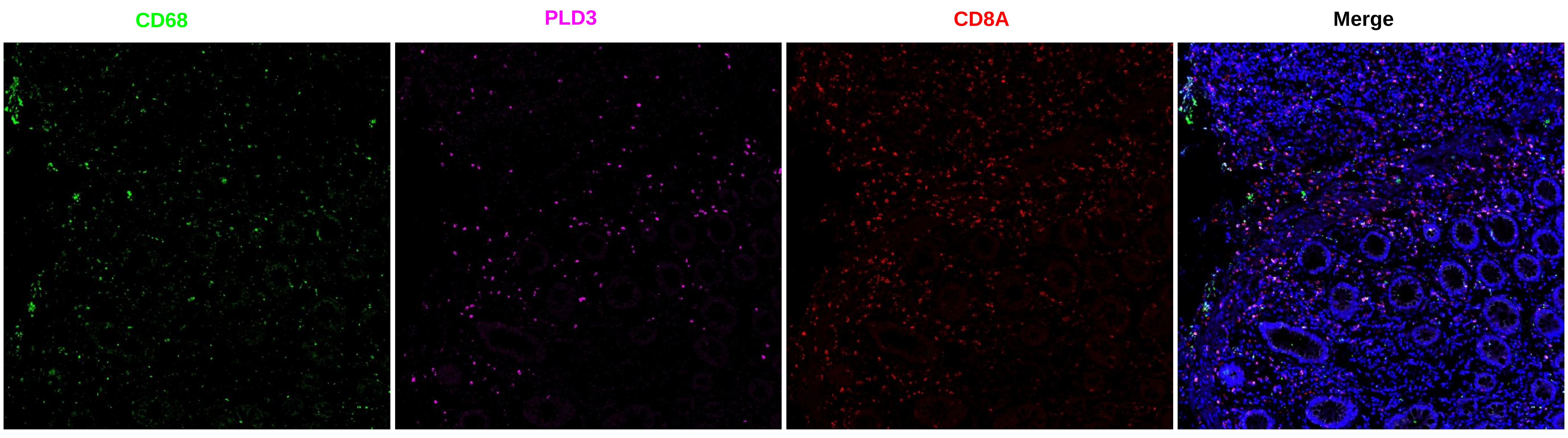

Figure 5A

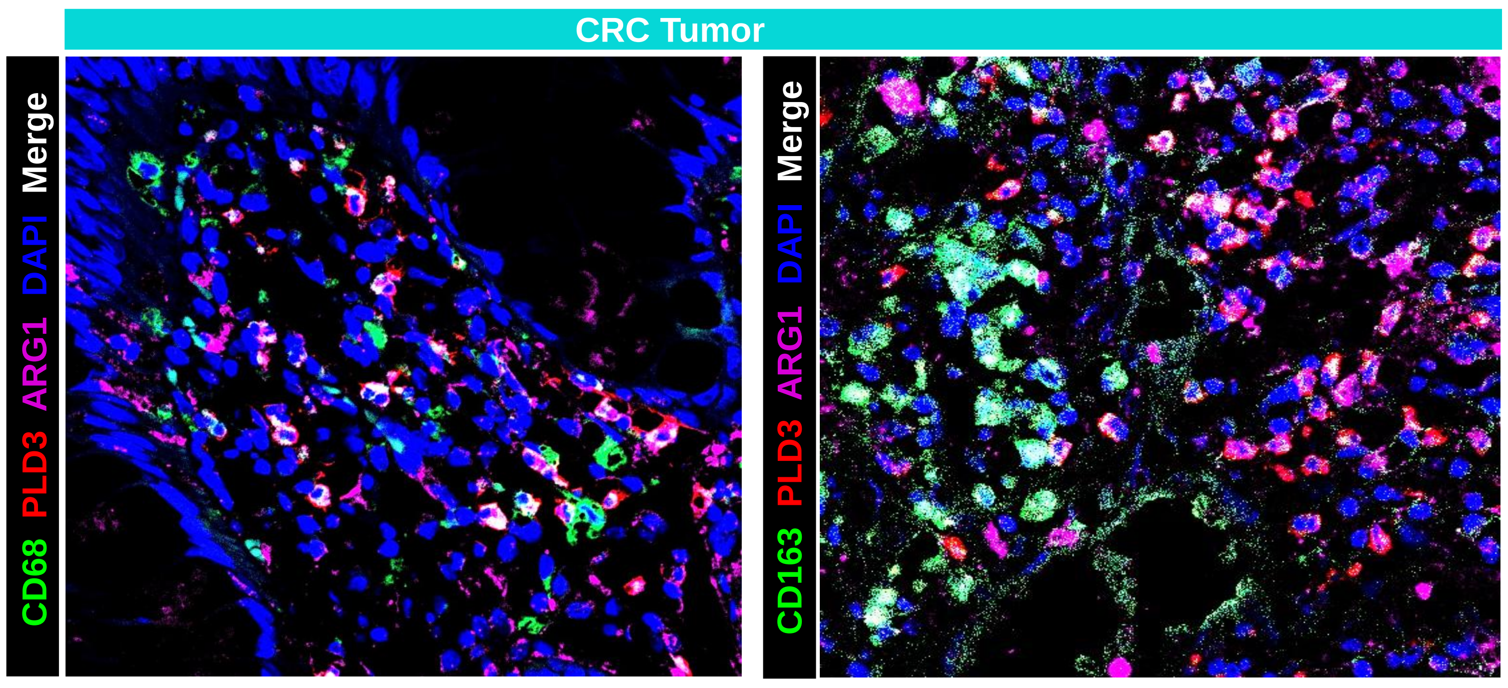

Figure 5G

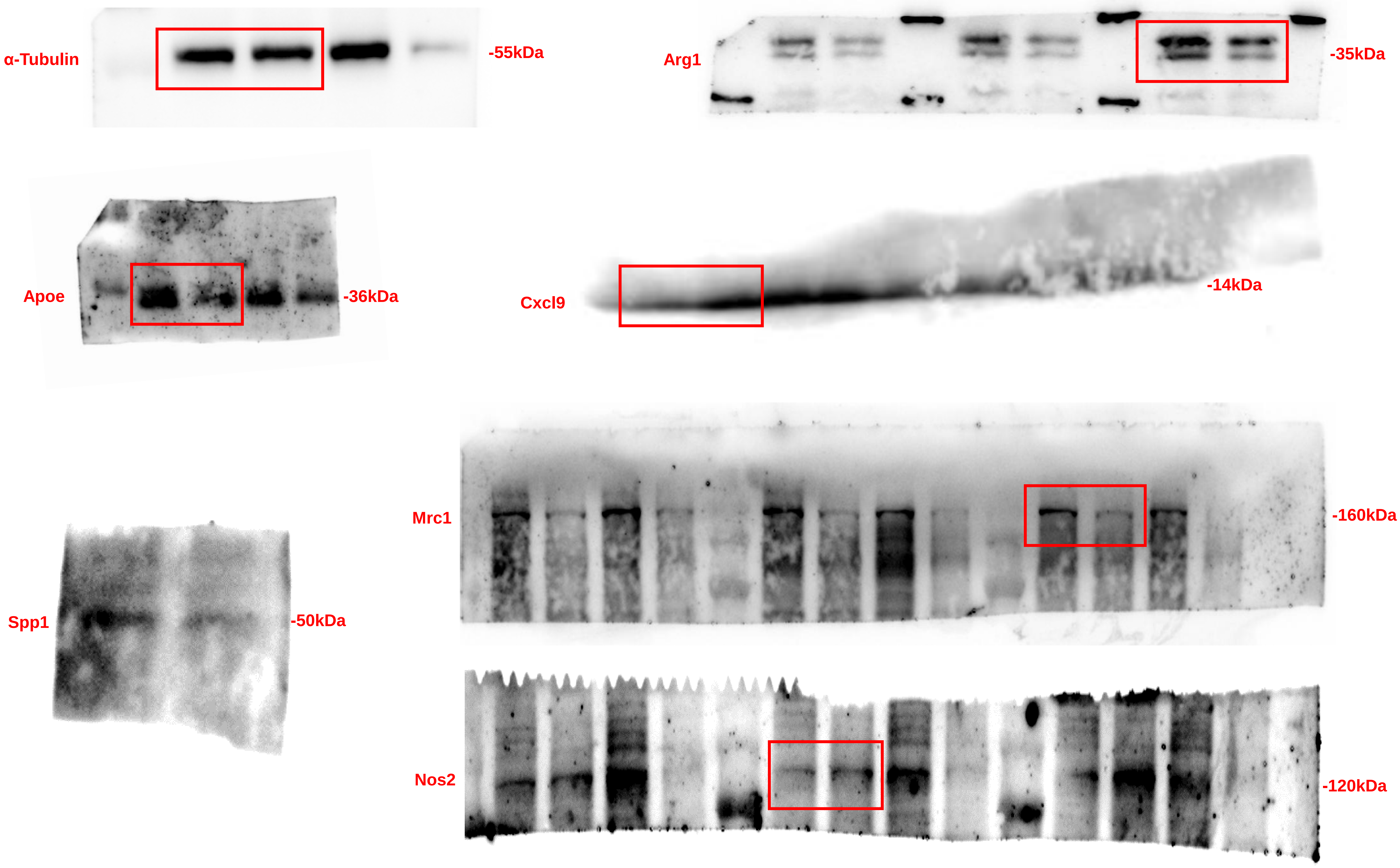

Figure 6D

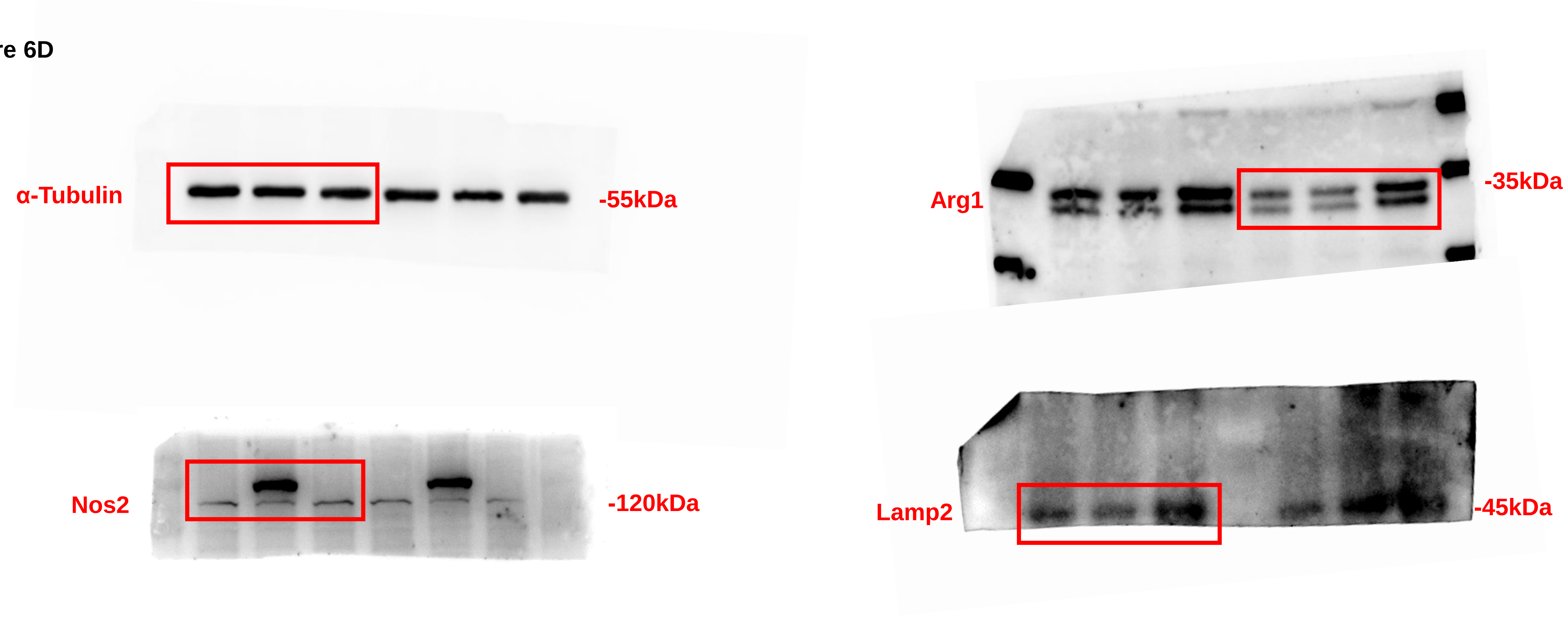

Figure 6E

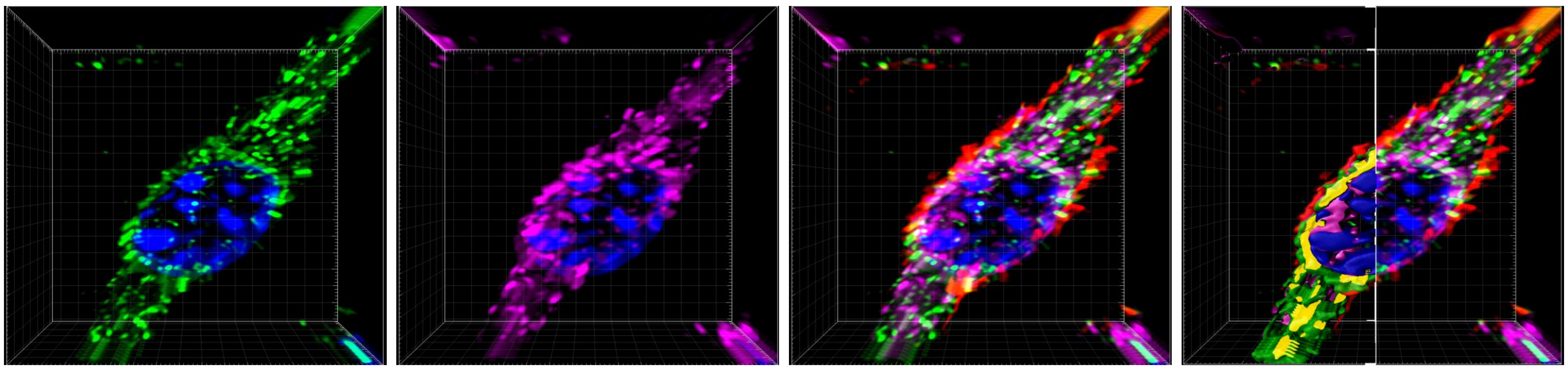

Figure 6G

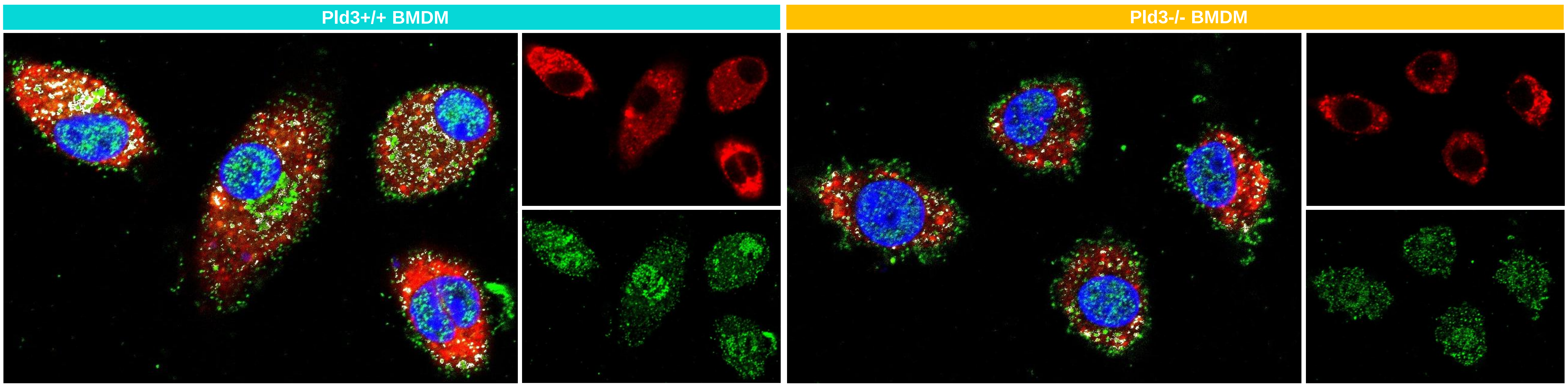

Figure 6J (Left), Pld3+/+ BMDM (Left) vs Pld3-/- BMDM (Right)

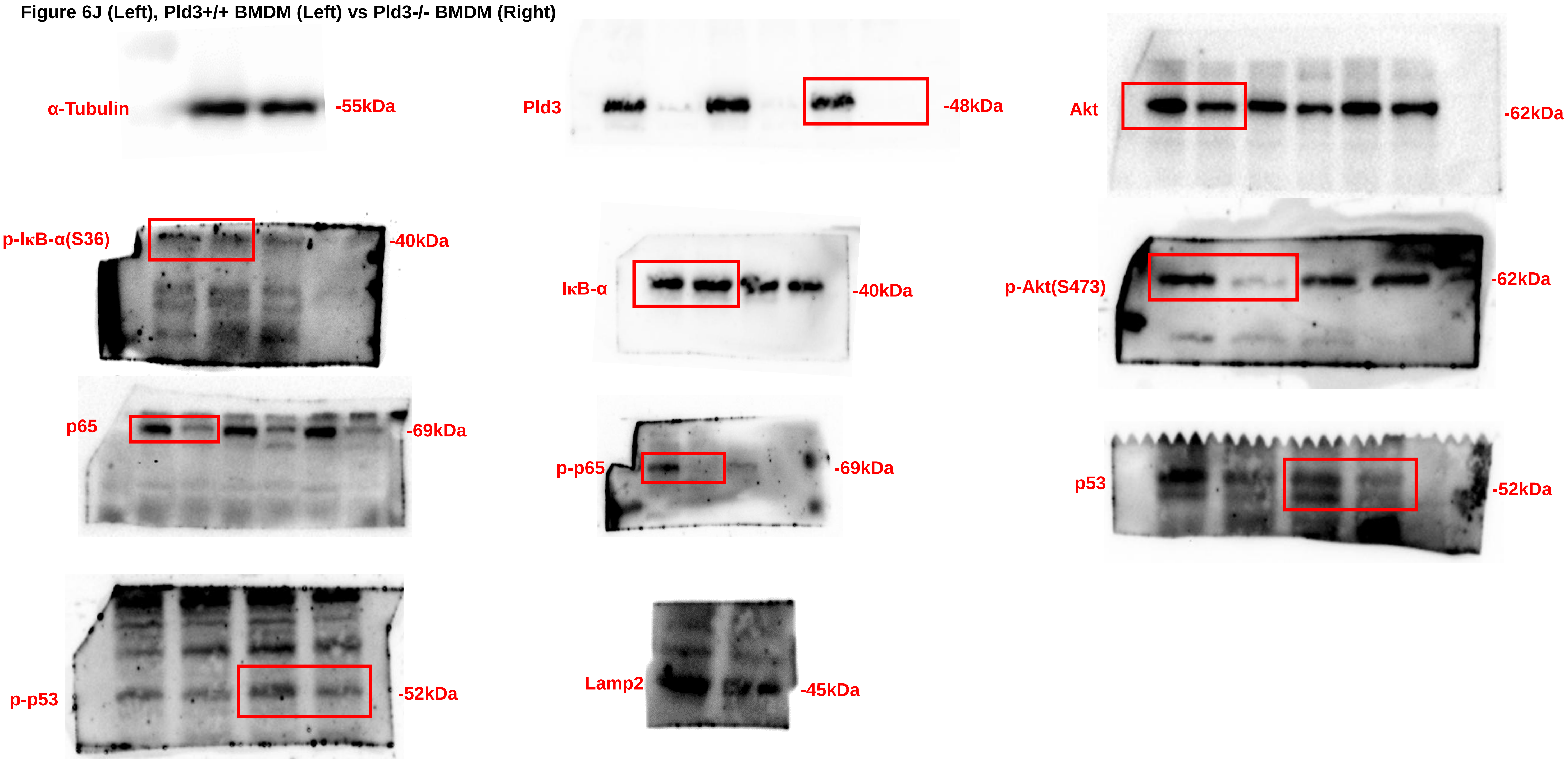

Figure 6J (Middle), RAW264.7-shNC (Left) vs RAW264.7-shPld3 (Right)

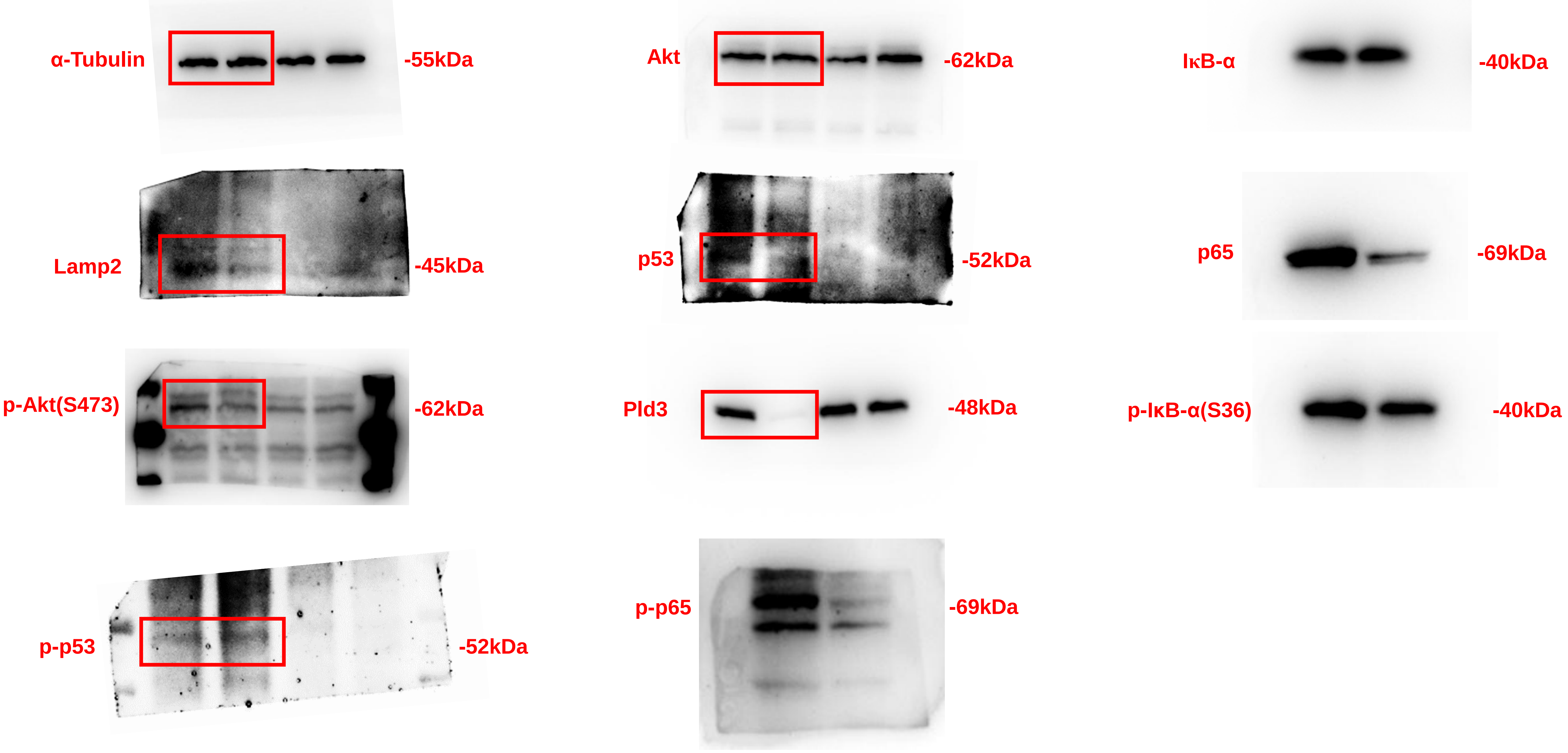

Figure 6J (Right), THP-1-NC (Left) vs THP-1-siPLD3 (Right)

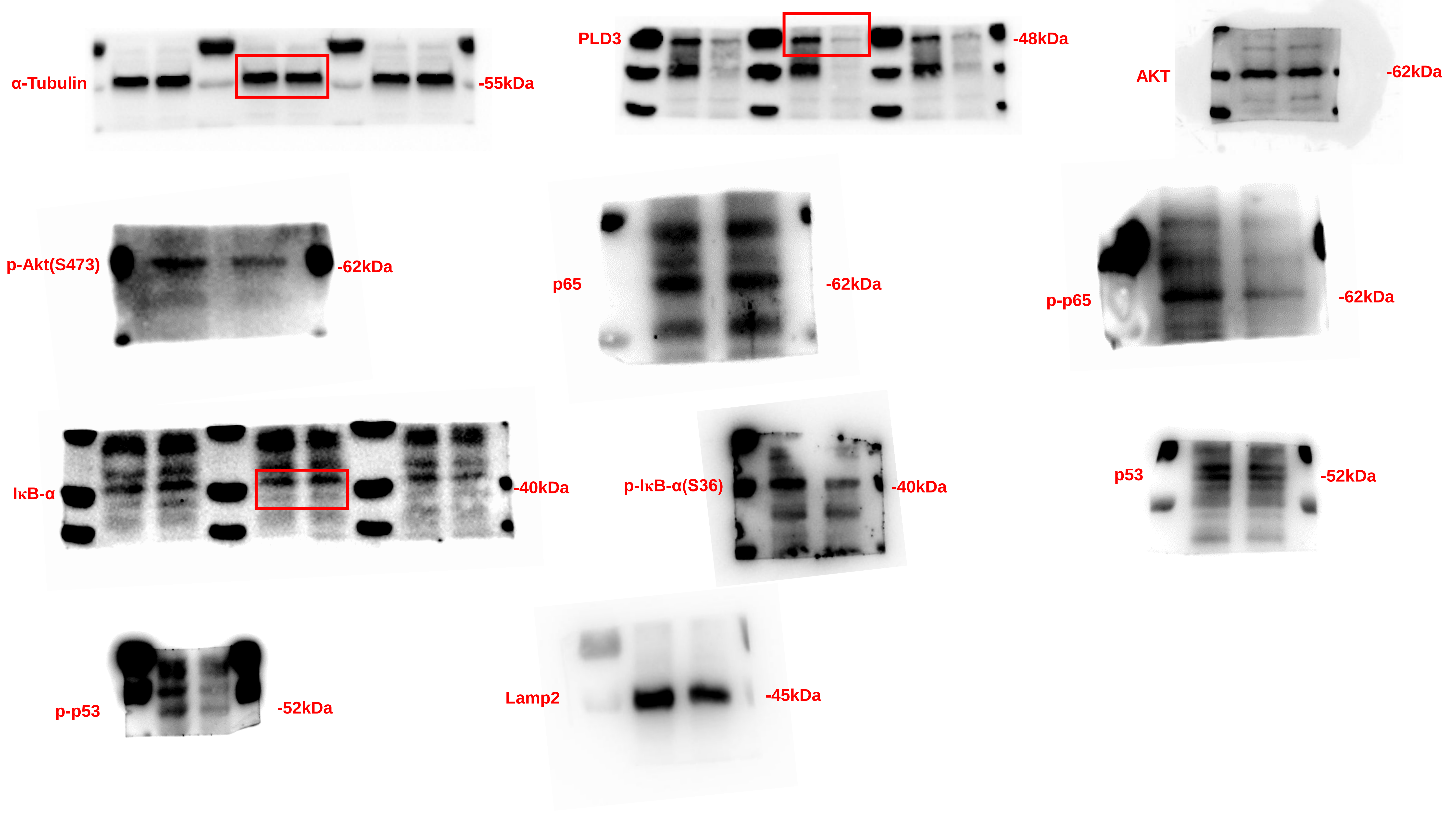

Figure 6K, (1)Pld3+/+ BMDM, (2)Pld3+/+ TAM, (3)Pld3-/- TAM

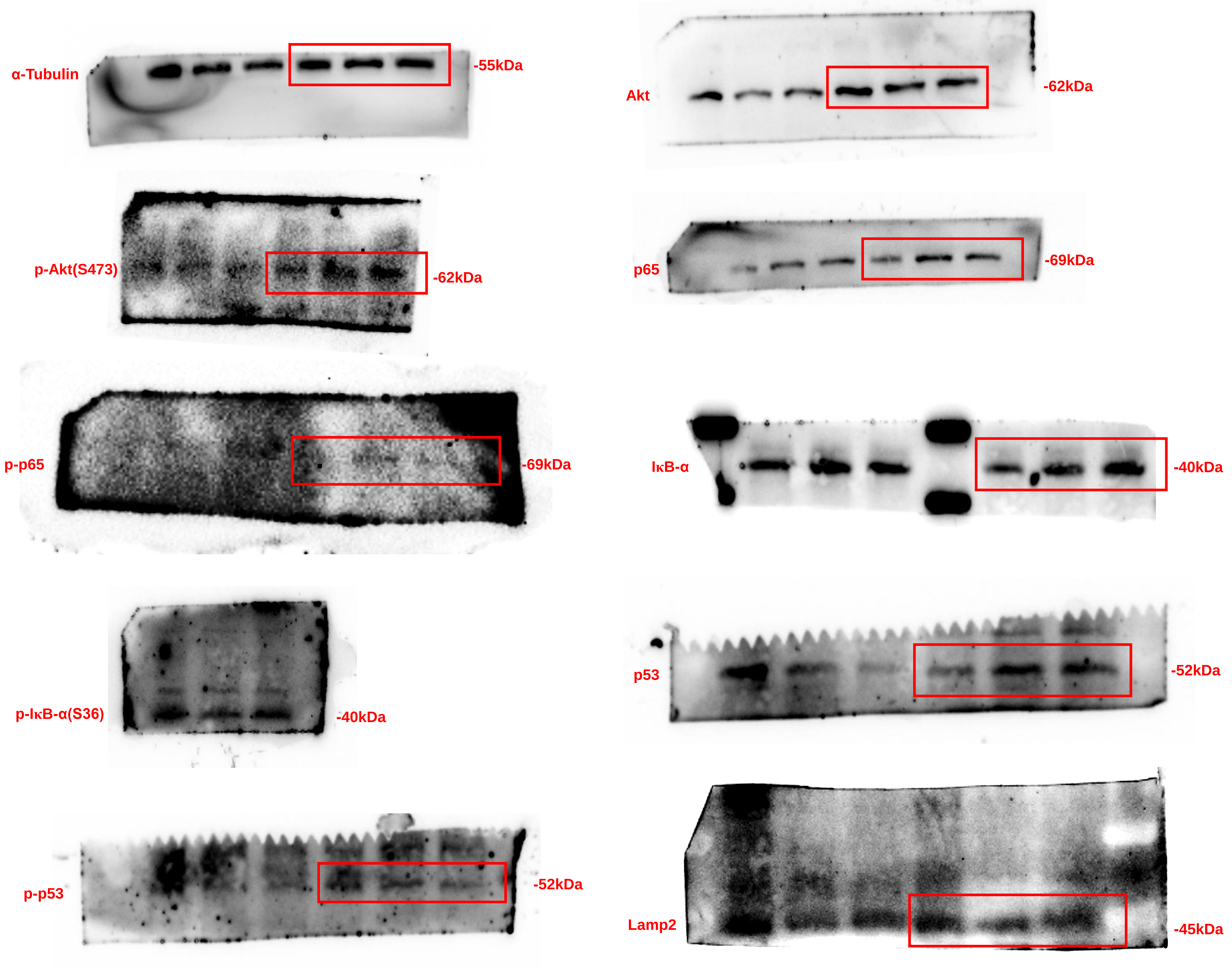

Figure 6L, (1)RAW-shNC-DMSO, (2)RAW-shPld3-DMSO, (3)RAW-shNC-10μMSC79, (4)RAW-shPld3-10μMSC79, (5)RAW-shNC-40μMSC79, (6)RAW-shPld3-40μMSC79

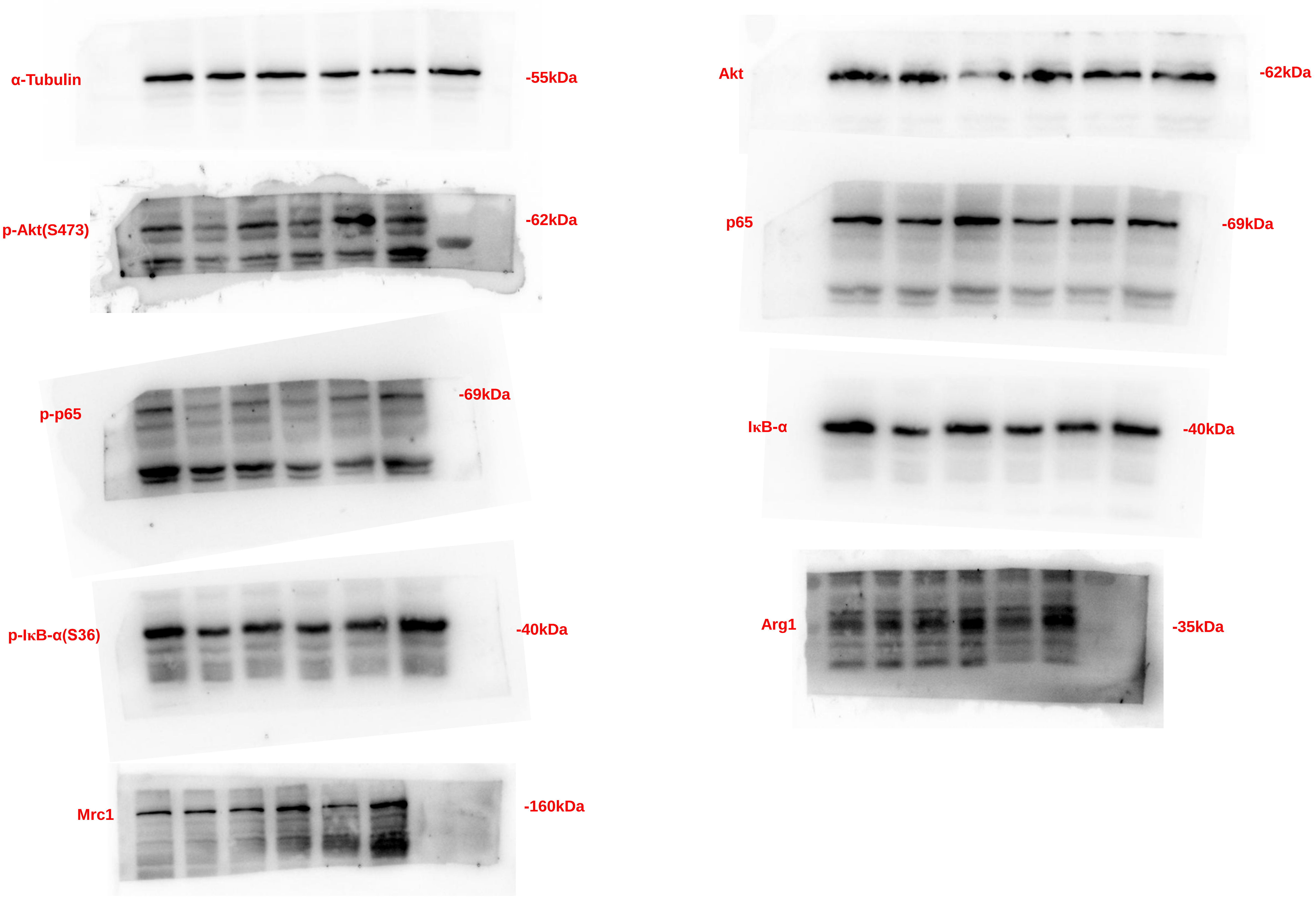

Figure 7A, Young-BMDM (Left) vs Senescence-BMDM (Right)

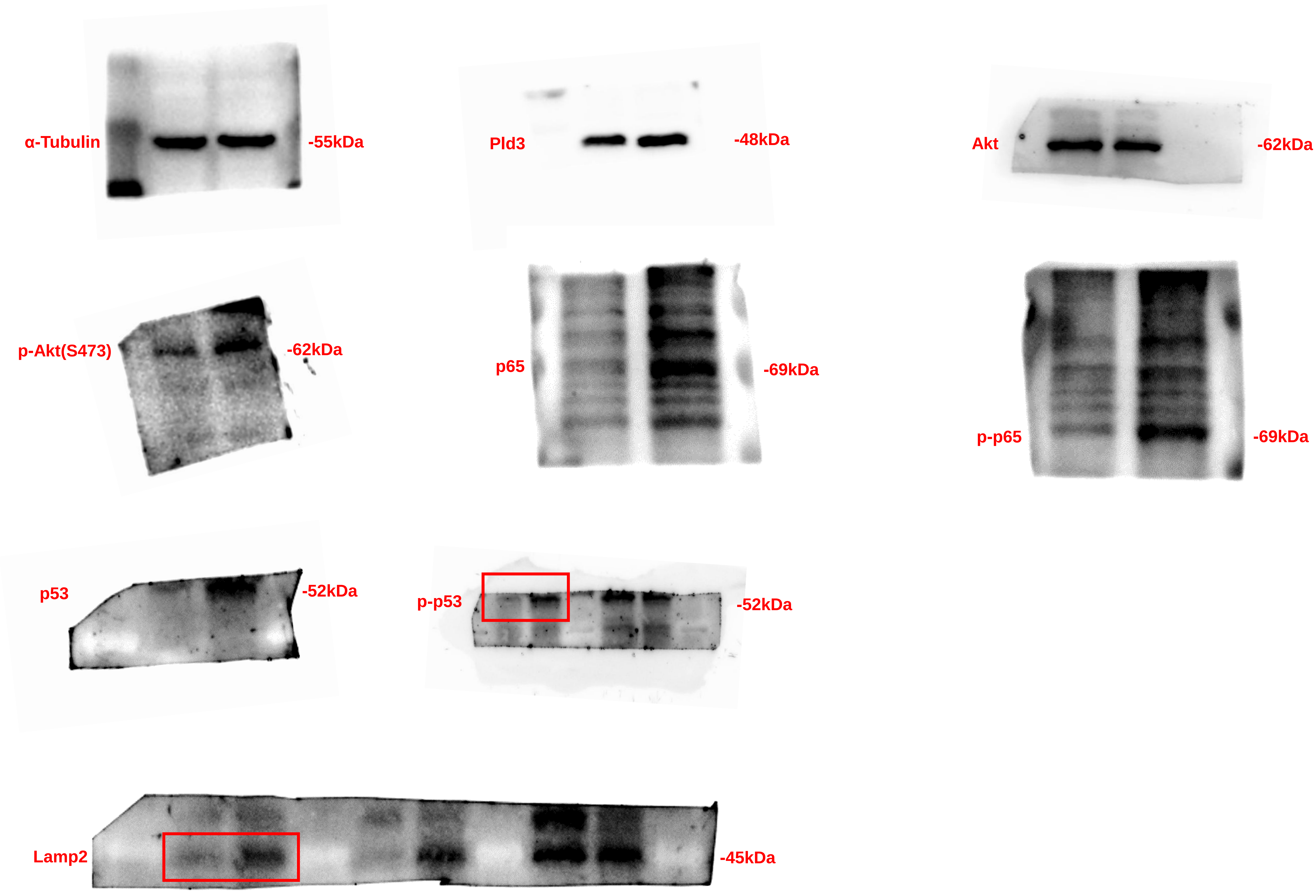

Figure 7B

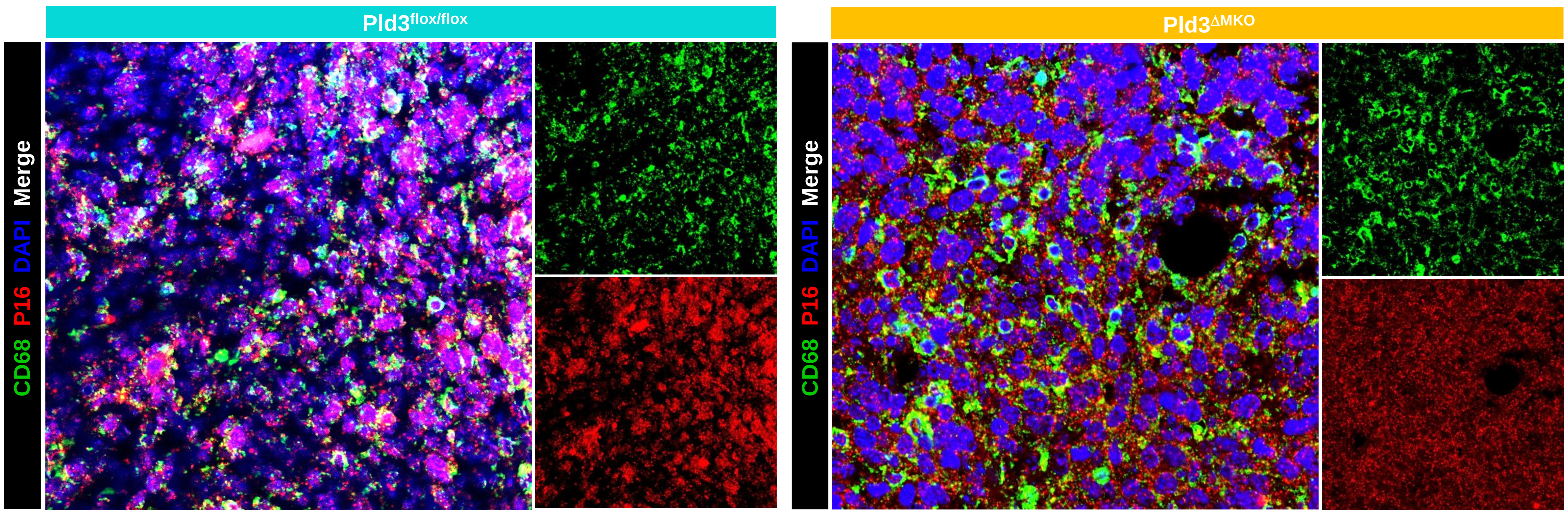

Figure 7G

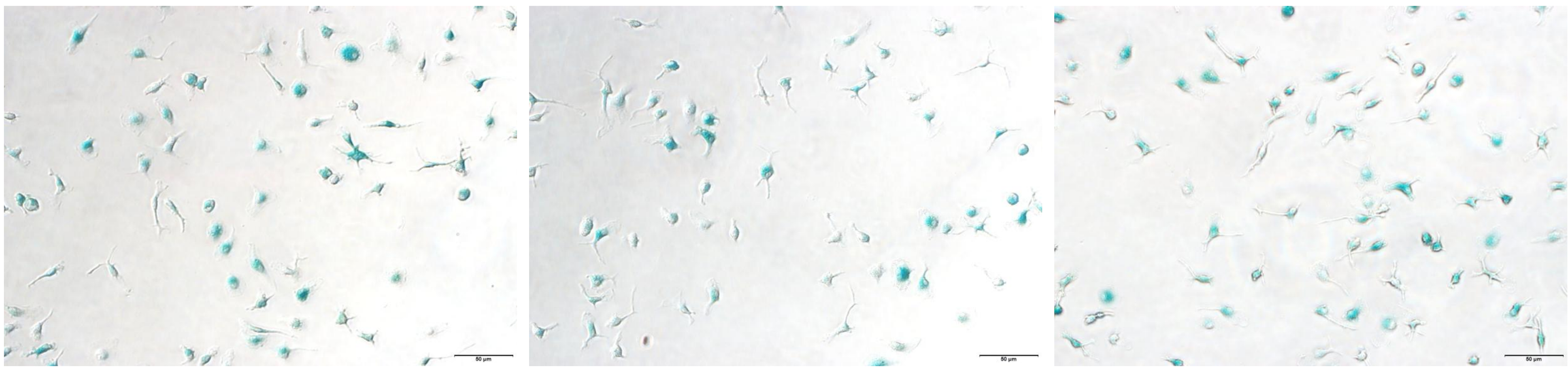

Figure 7H

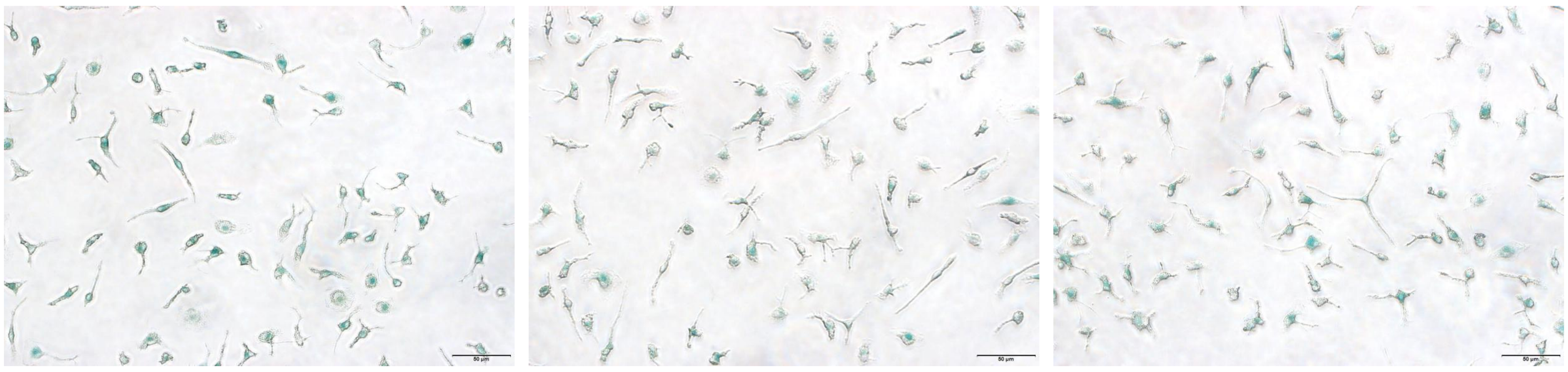

Figure 8I, (1) BMDM, (2) BMDM+Abrine, (3) BMDM+IDO1-IN-18, (4) BMDM+Abrine+IDO1-IN-18

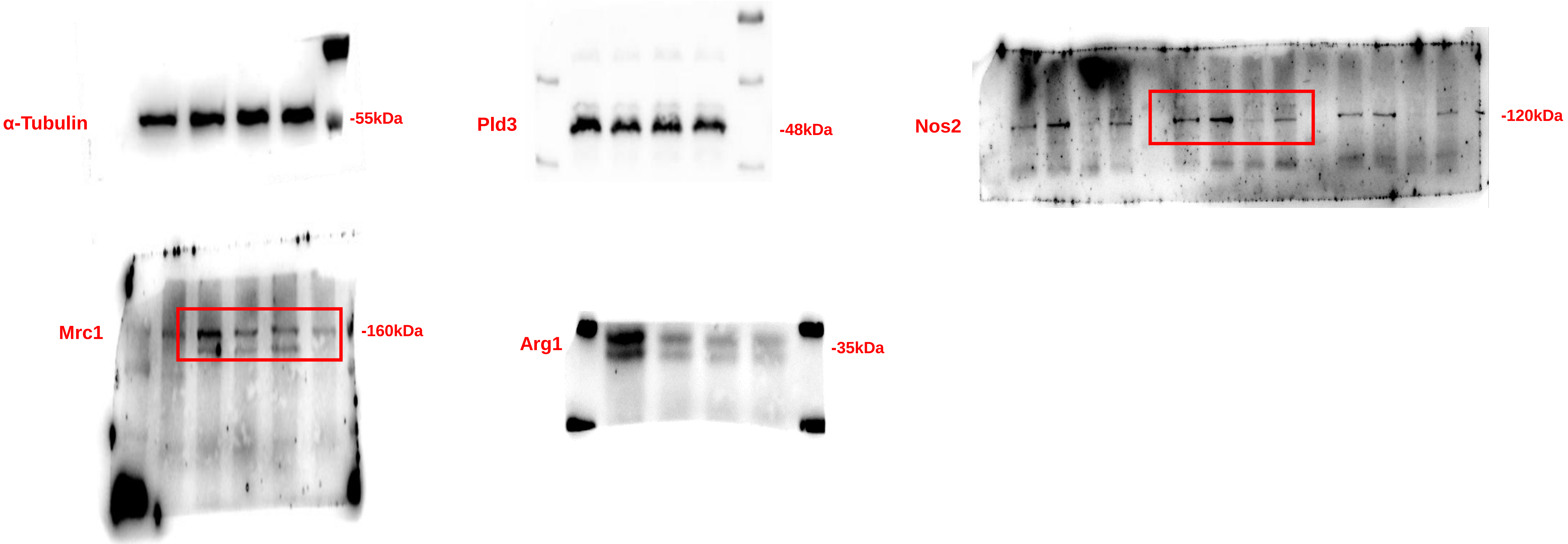

Figure 8K

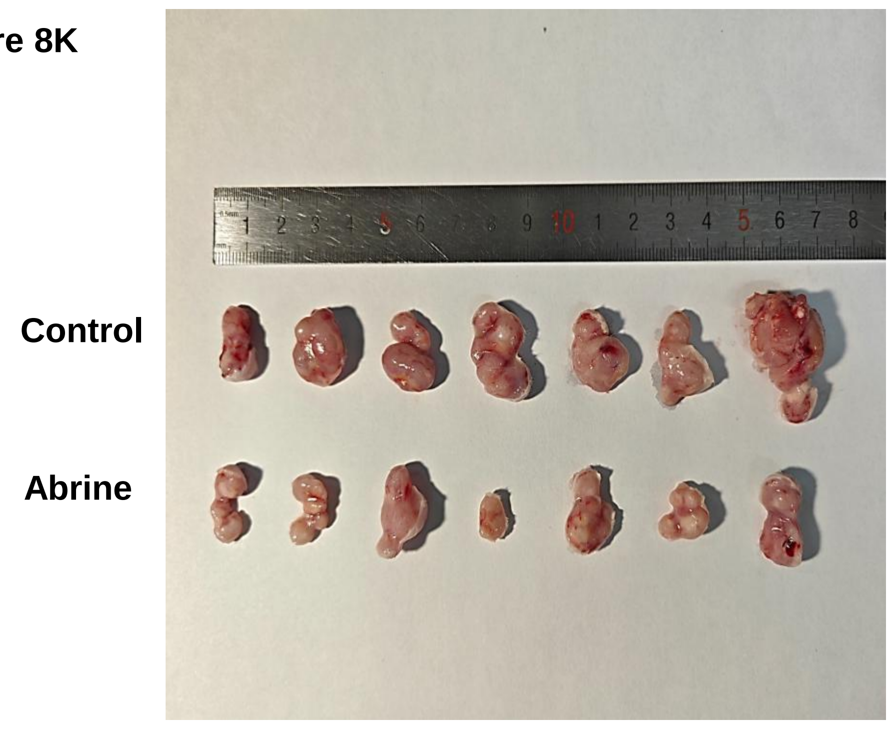

Figure 8N

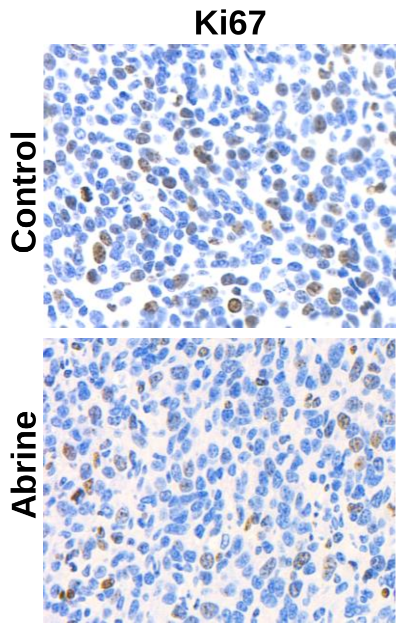

Figure 9M

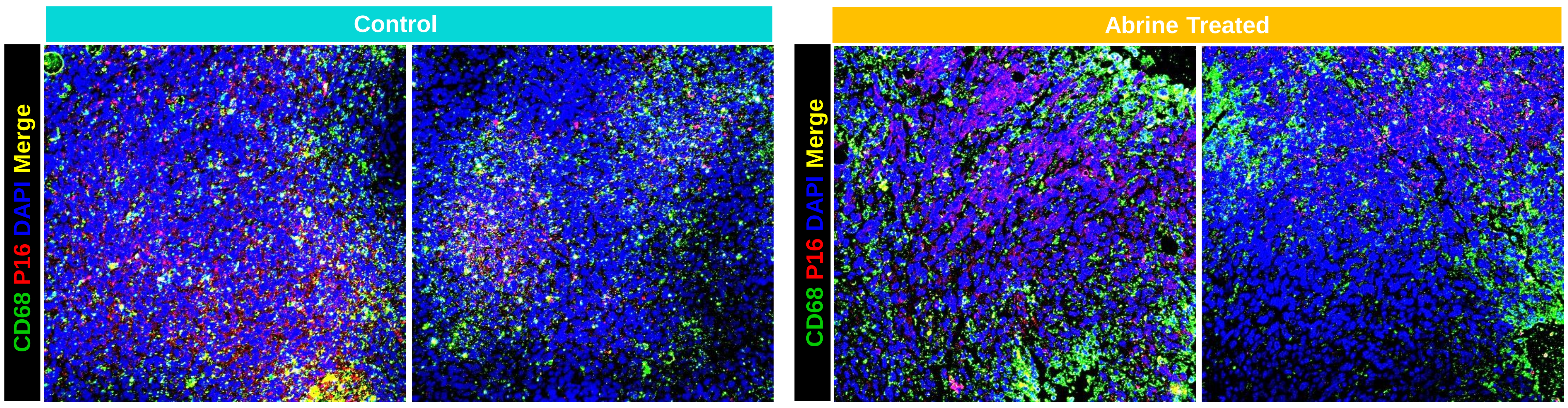

Figure 10B

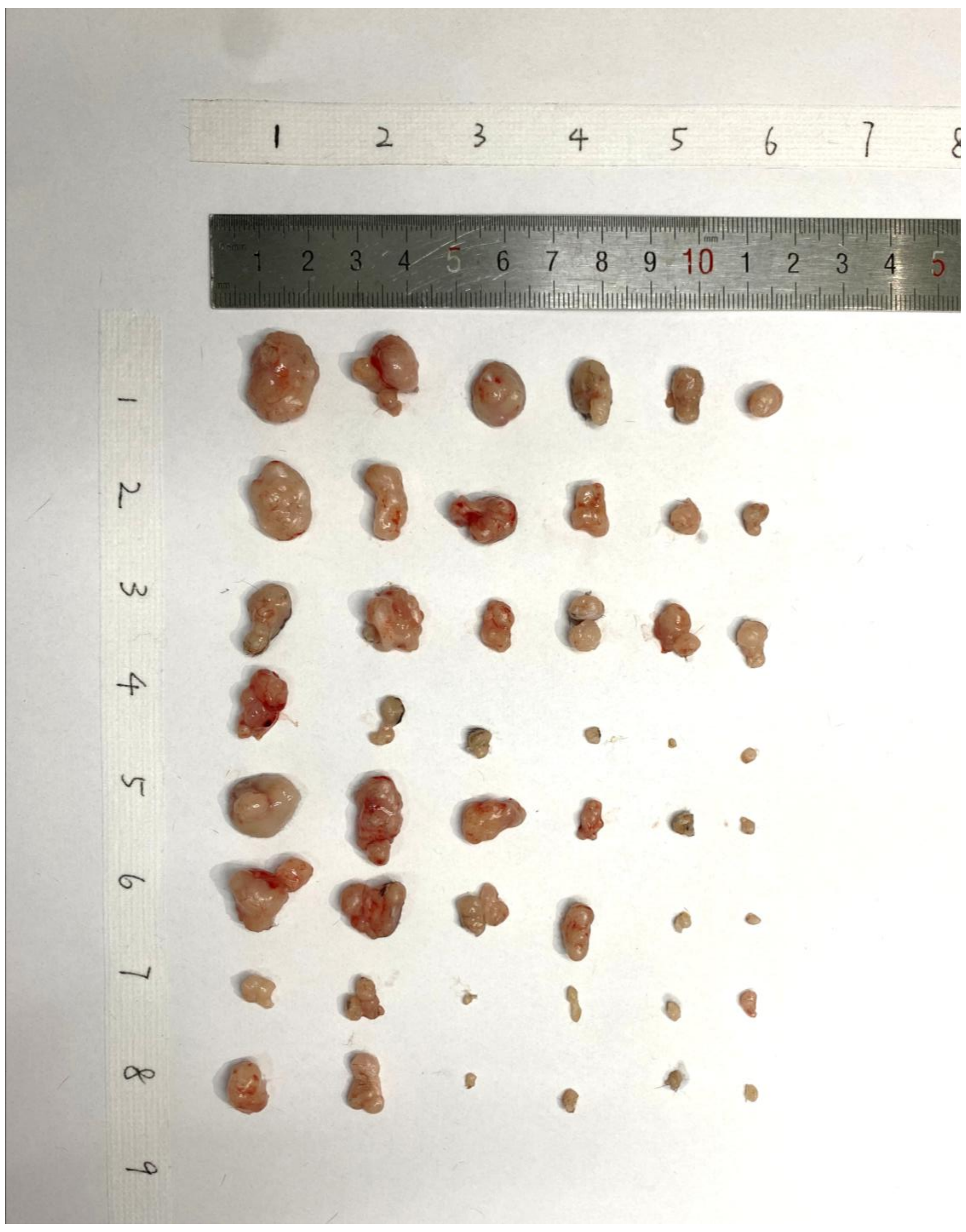

Figure 10G

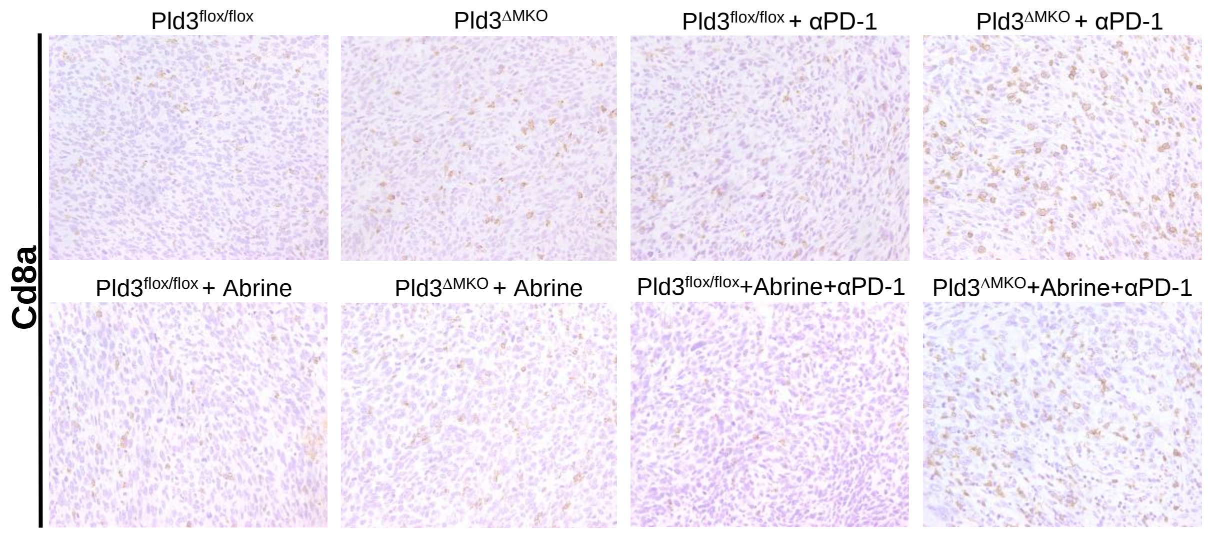

Figure 10H

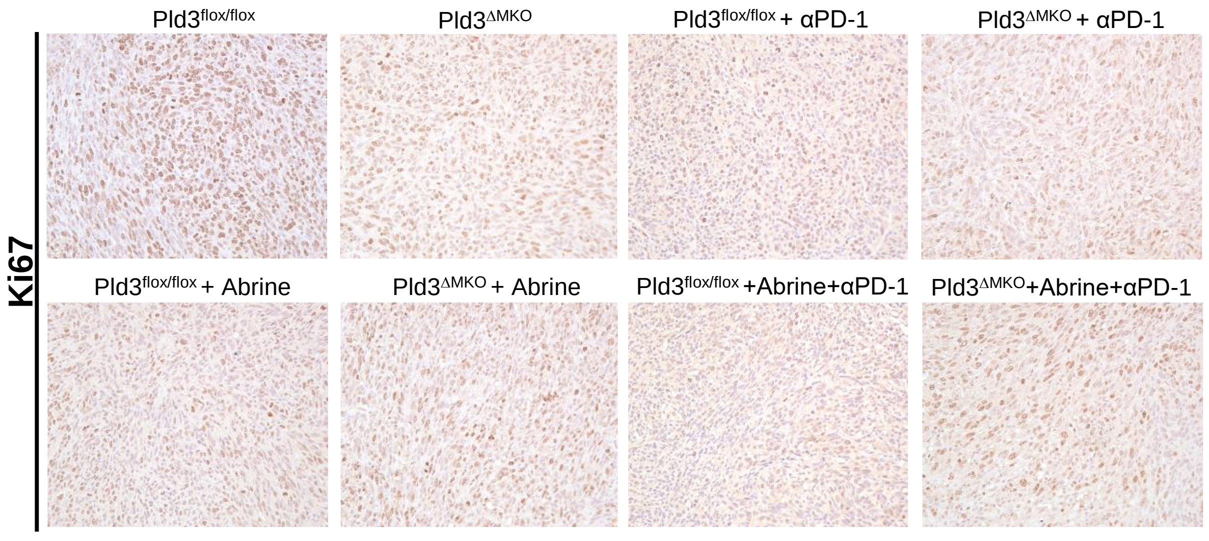

Figure 10L

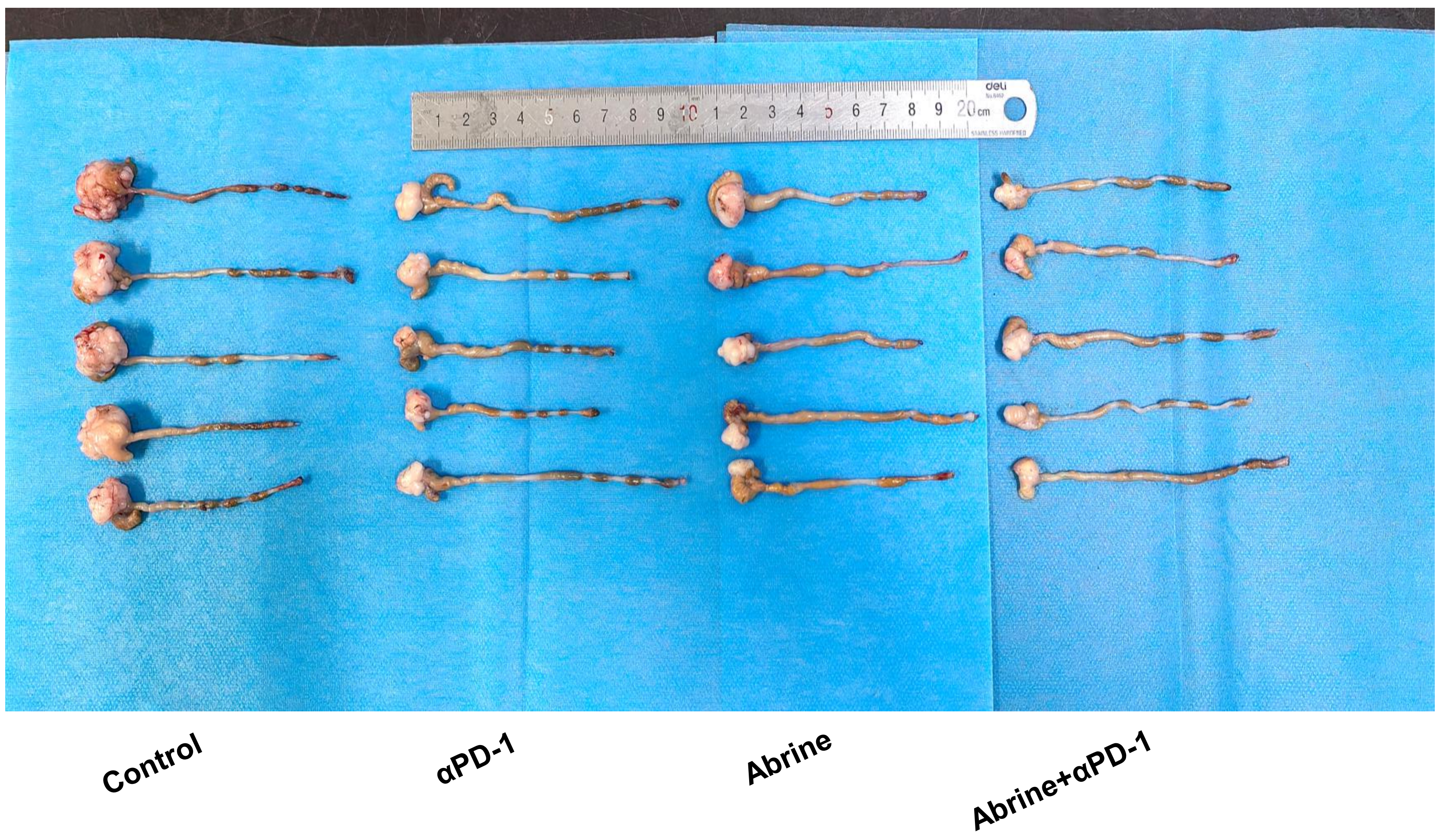

Figure 10M

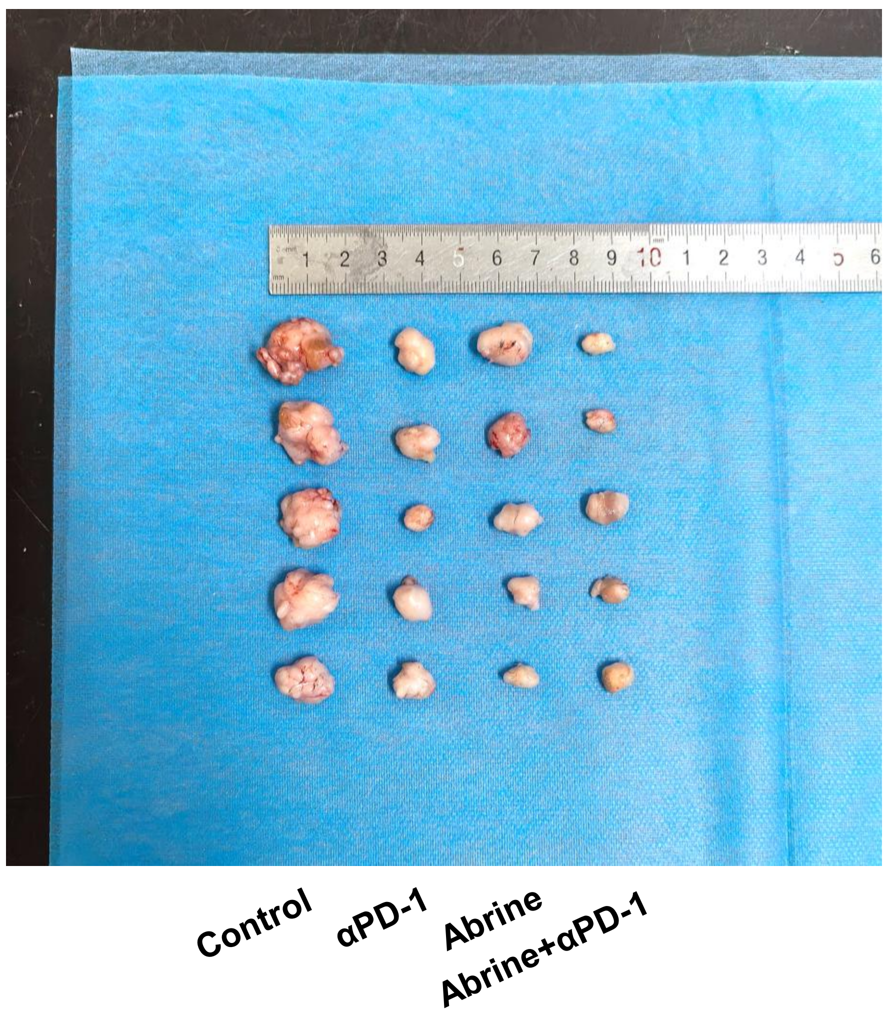

Figure S2A

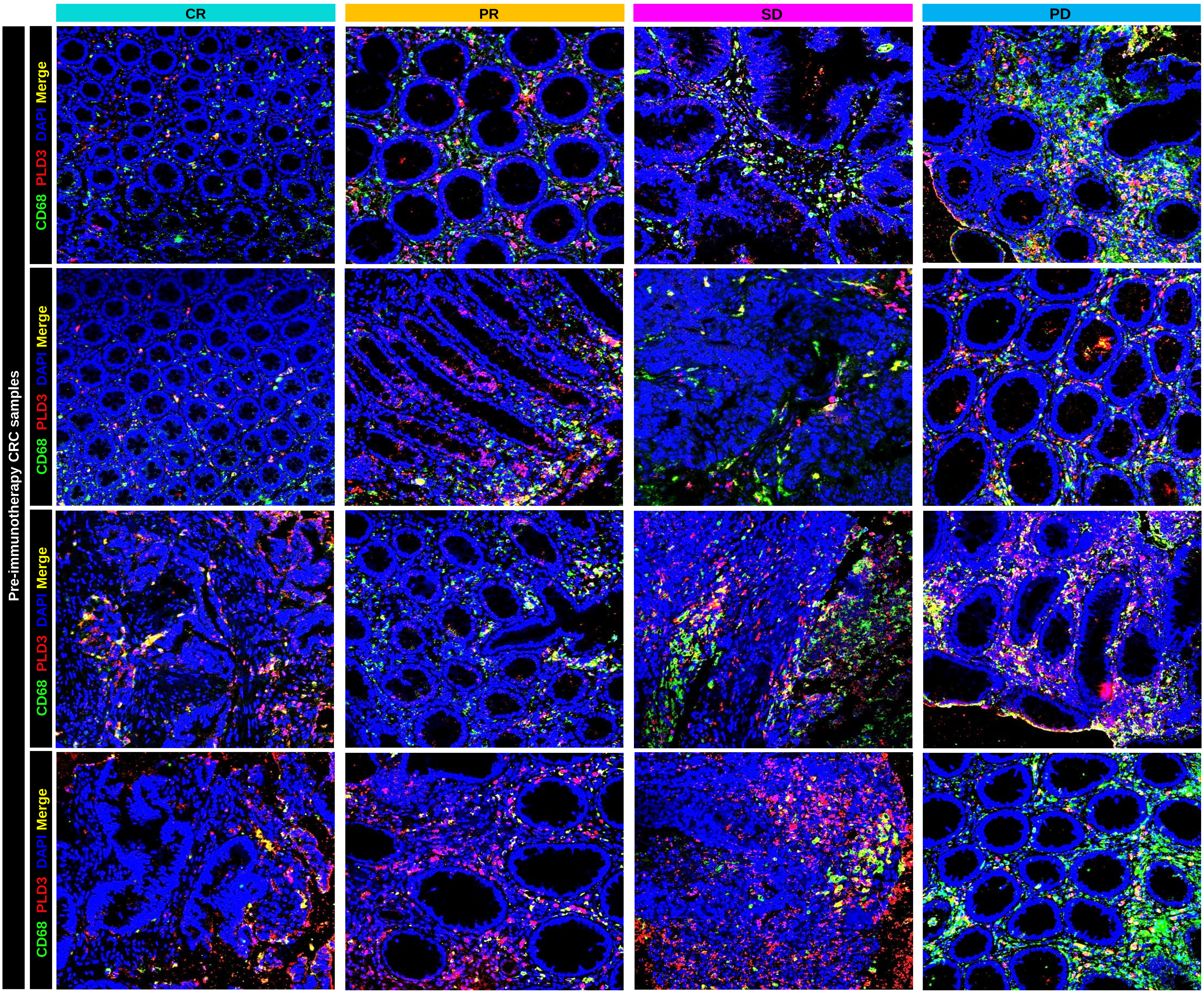

Figure S3G

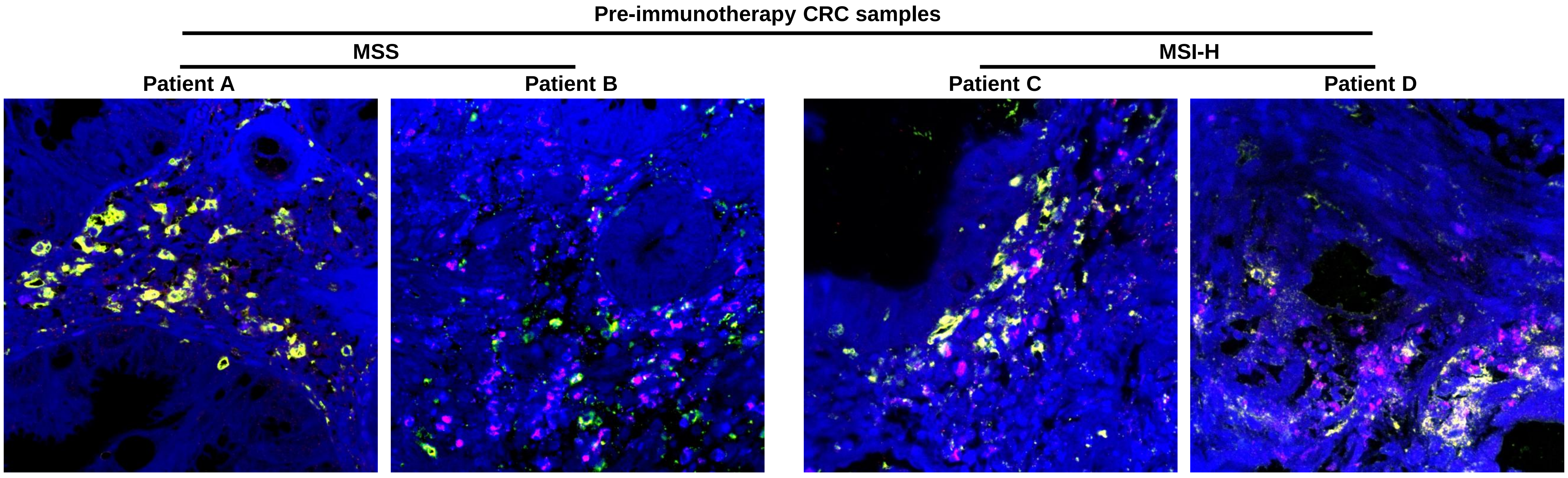

Figure S4E

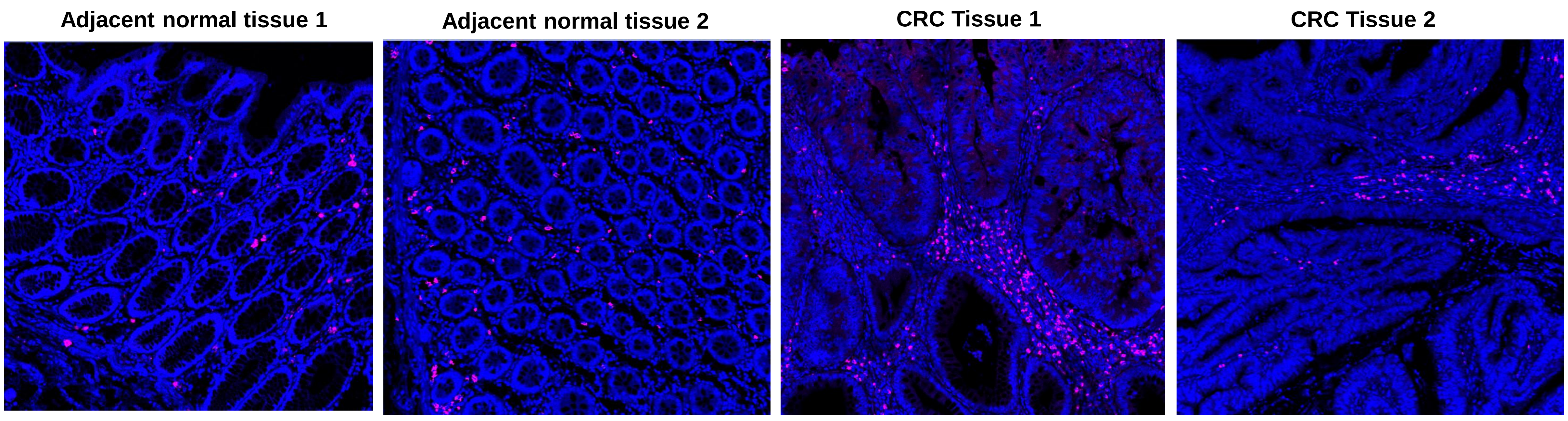

Figure S4I

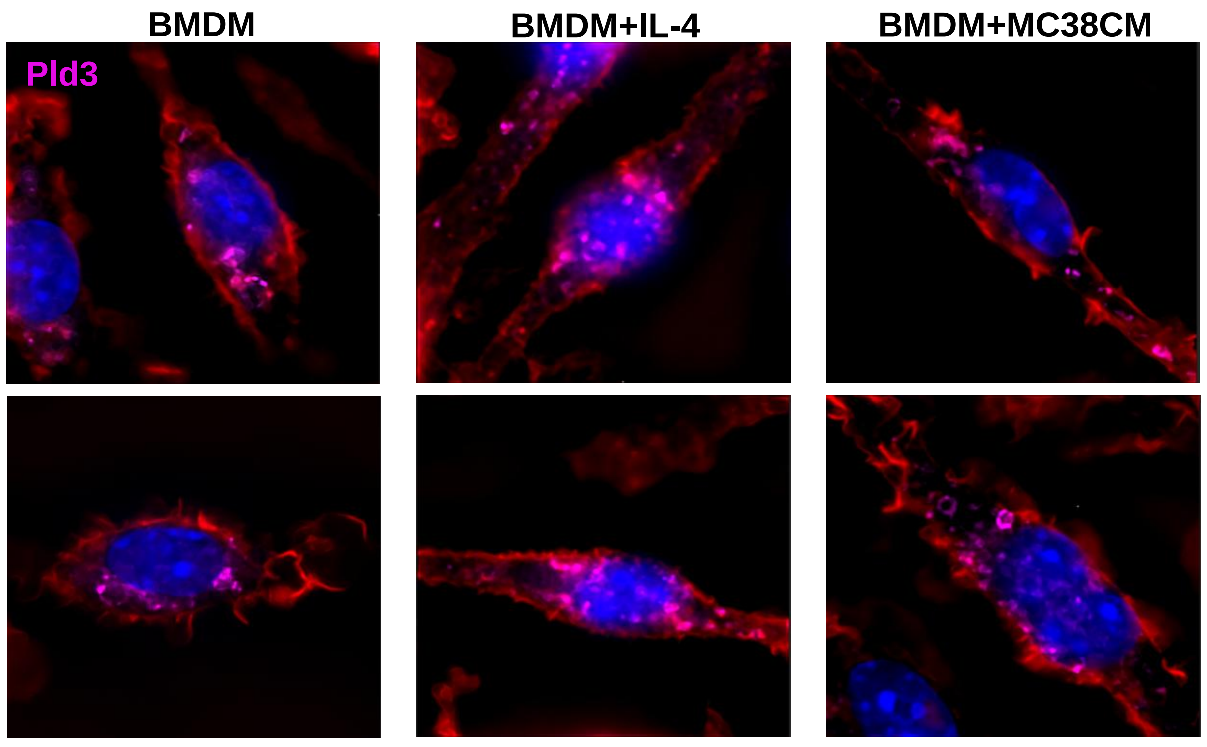

Figure S5A

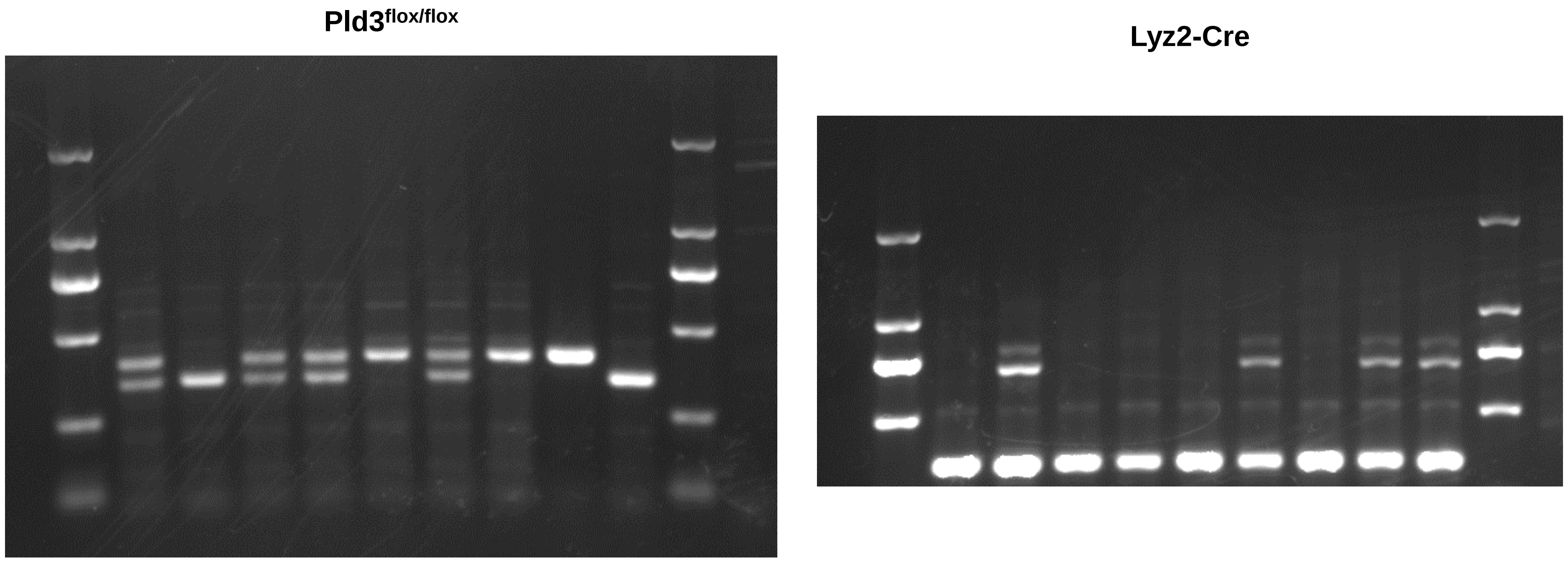

Figure S5B

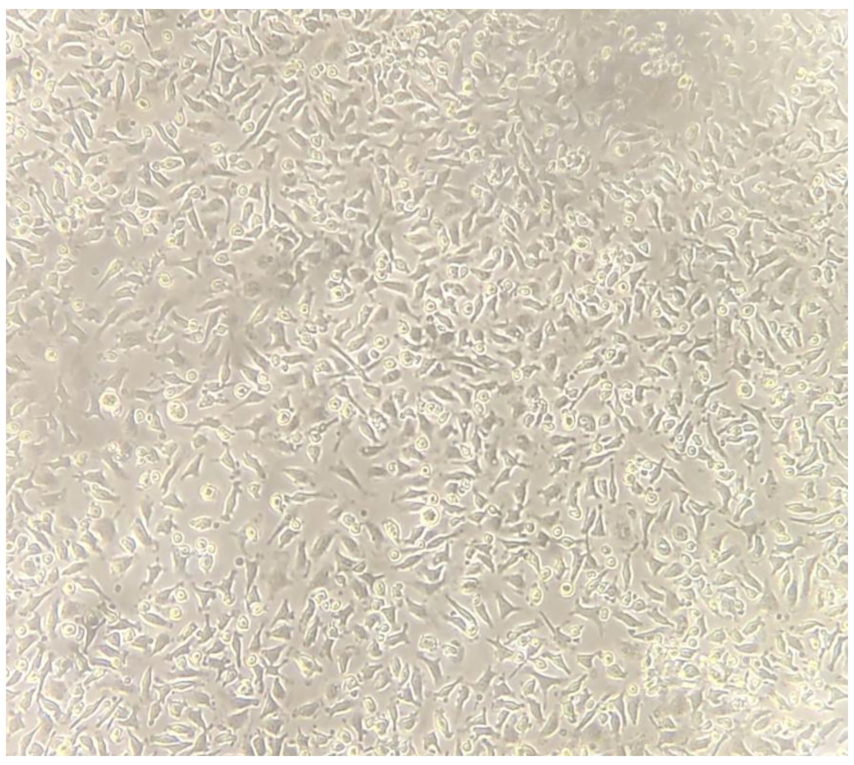

Figure S5E, Pld3+/+ BMDM (Left) vs Pld3-/- BMDM (Right)

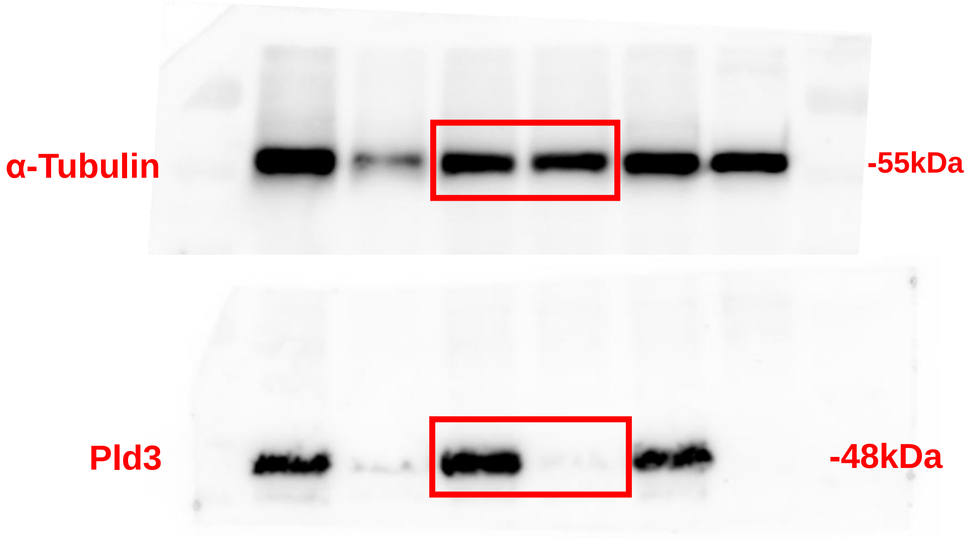

Figure S5F

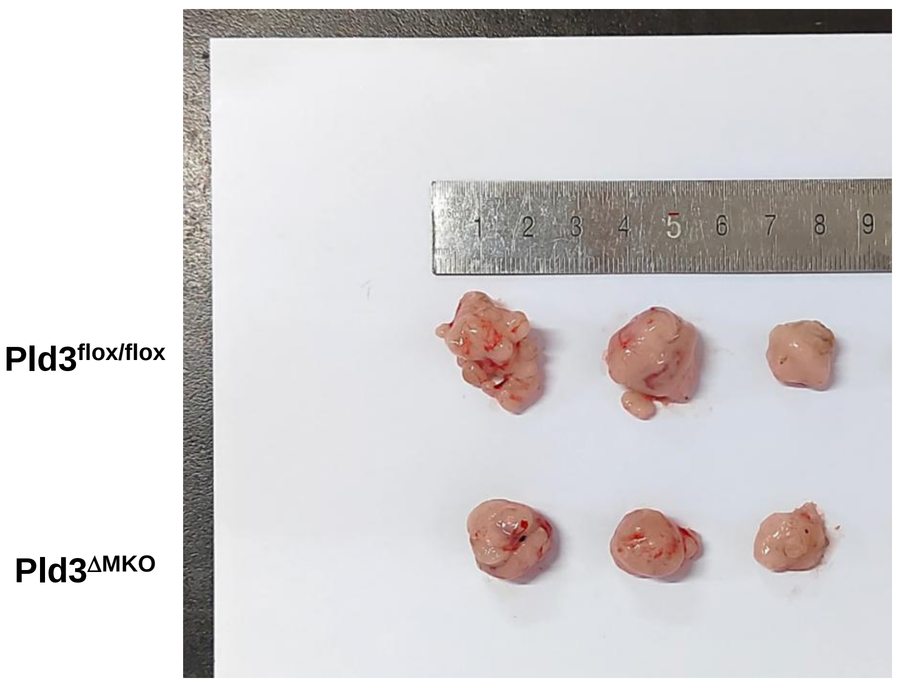

Figure S6A

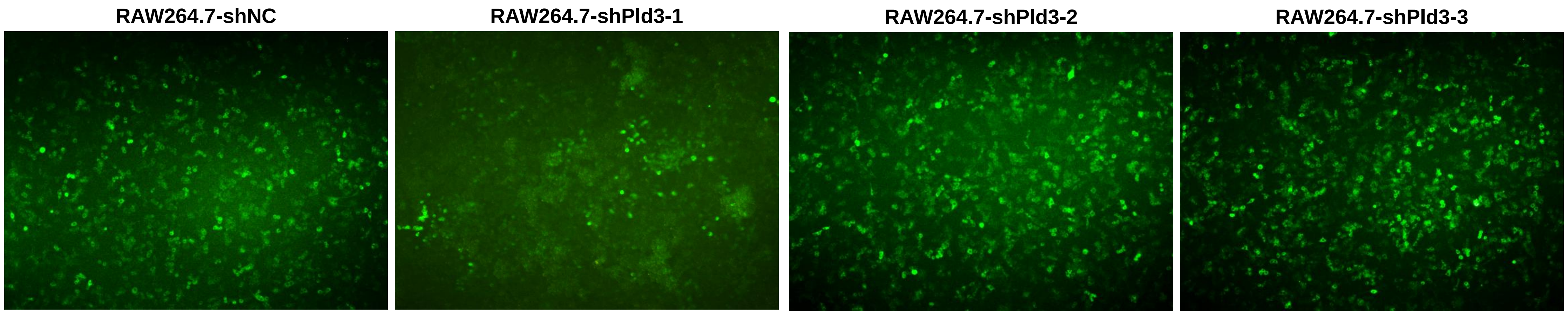

Figure S6C, RAW264.7-shNC (Left) vs RAW264.7-shPld3 (Right)

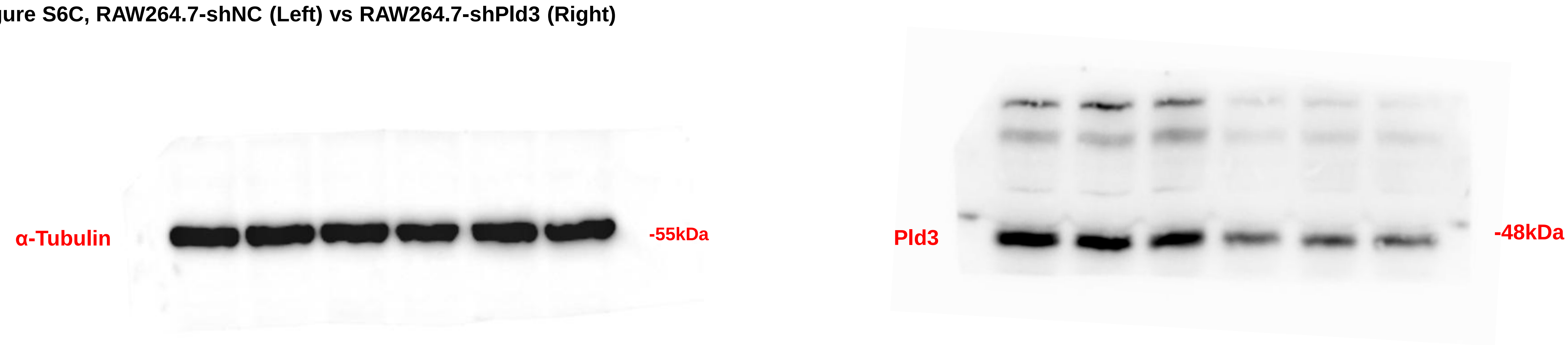

Figure S7B

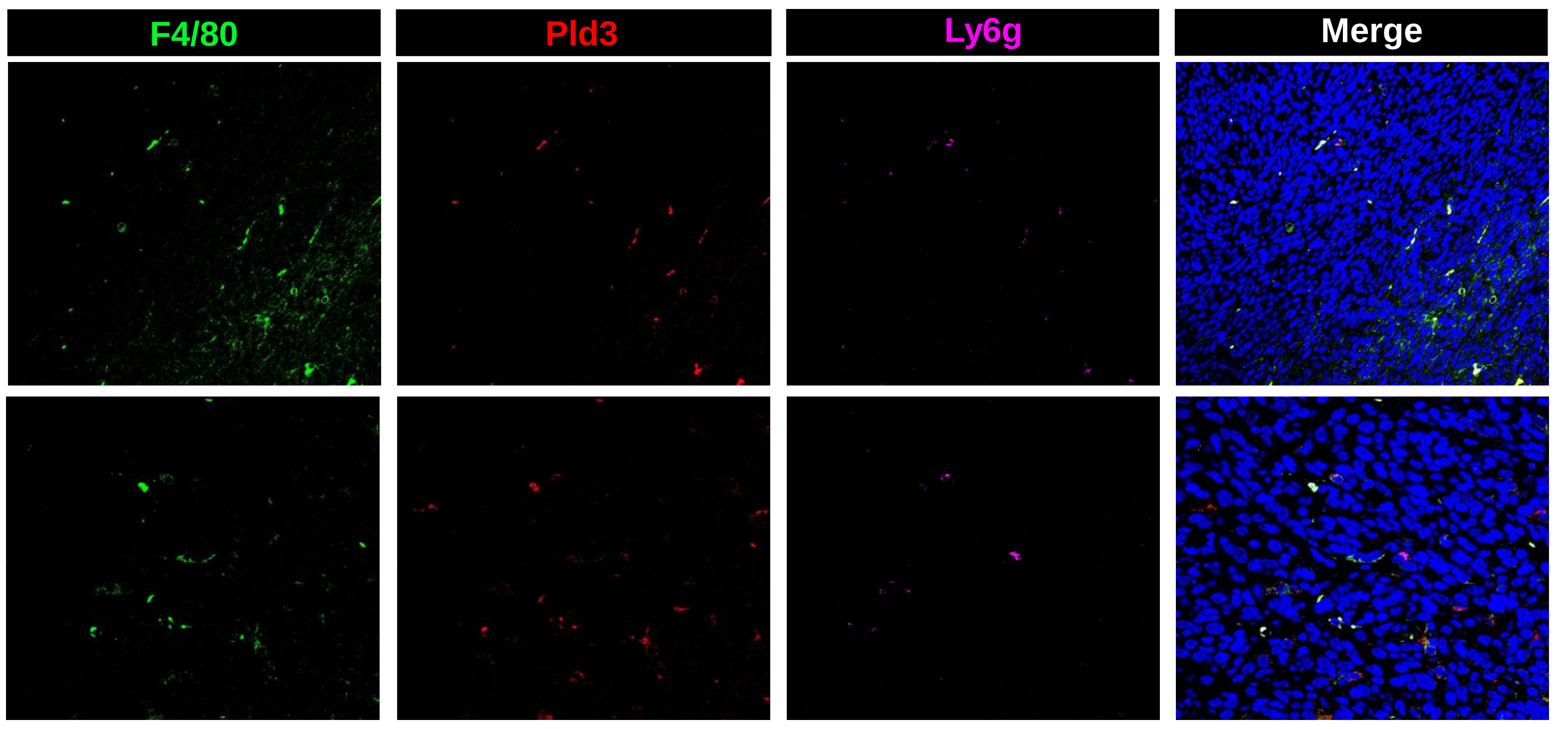

Figure S13A

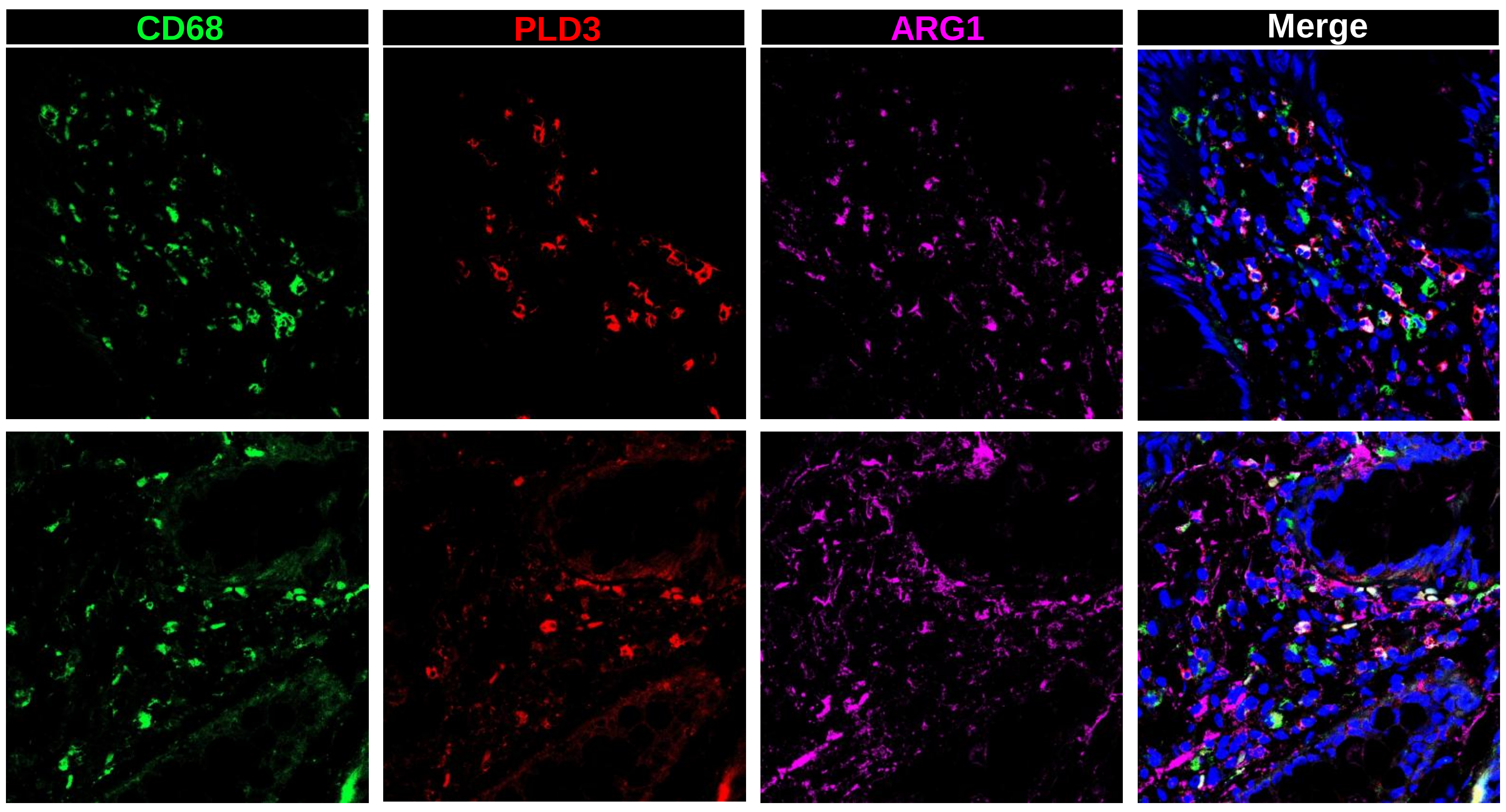

Figure S13B

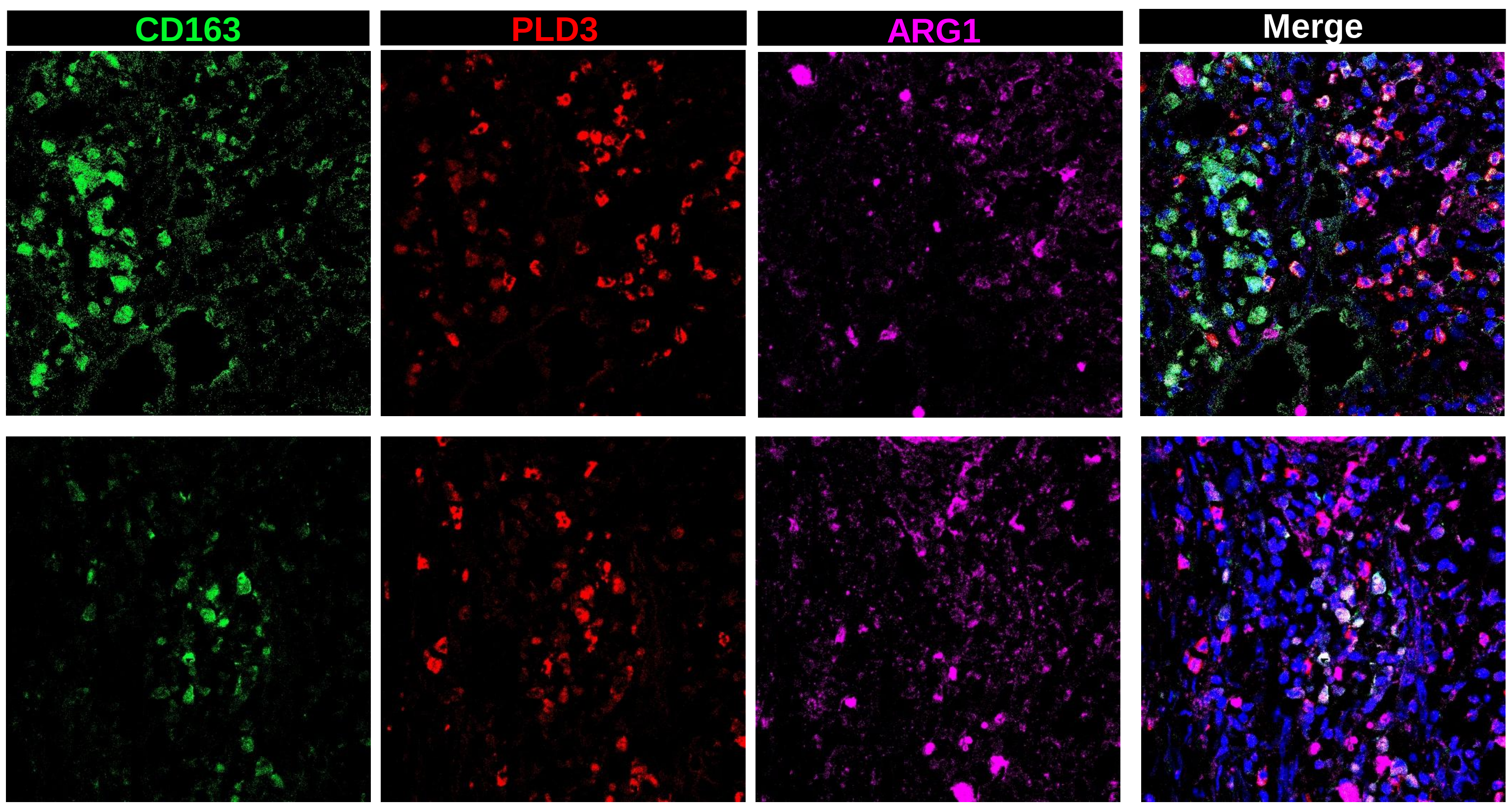

Figure S13C, (1)RAW264.7, (2) RAW264.7+LPS, (3) RAW264.7+IL4

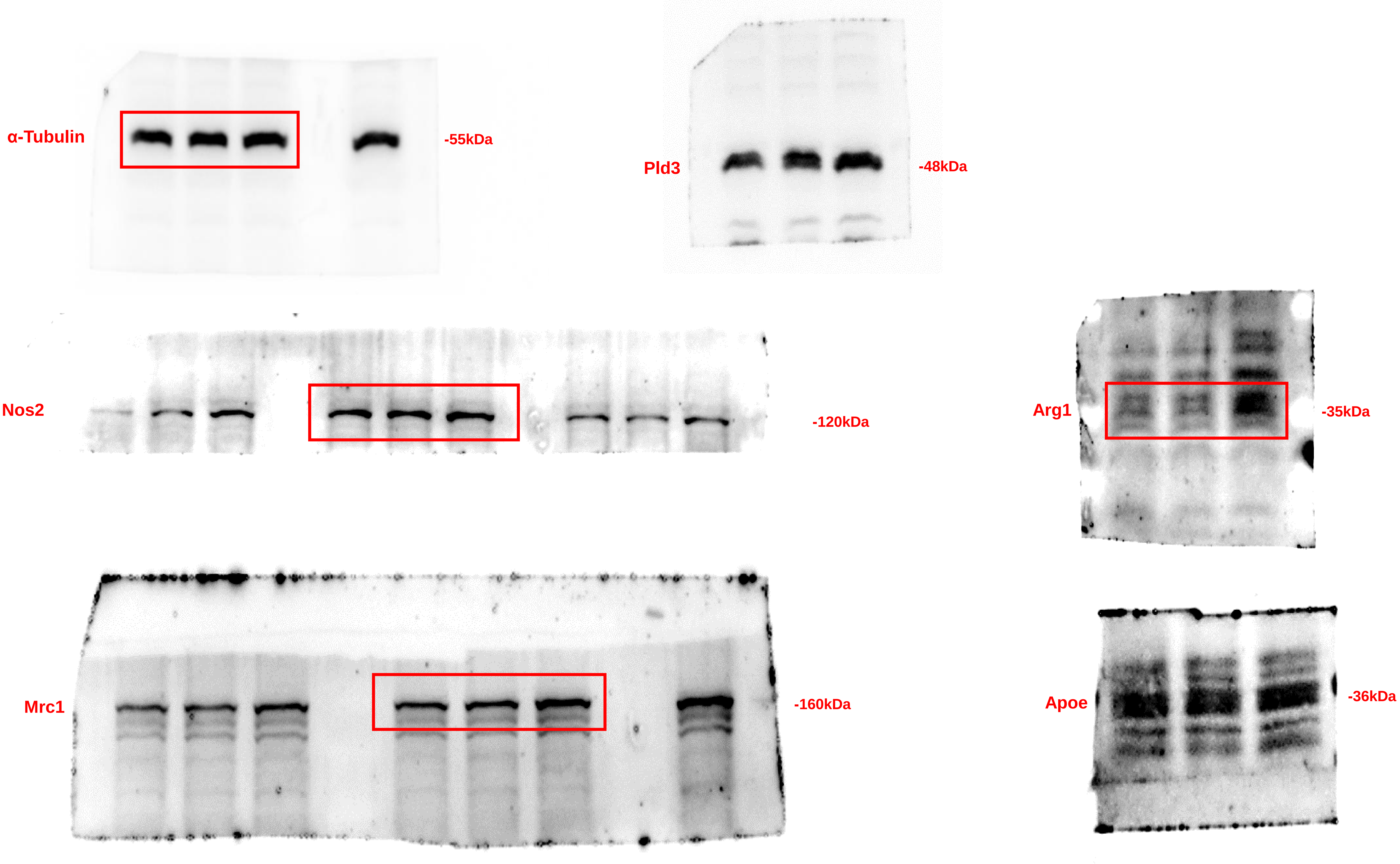

Figure S13F, (1)RAW264.7-Input, (2) RAW264.7-IgG, (3) RAW264.7-Anti-Pld3

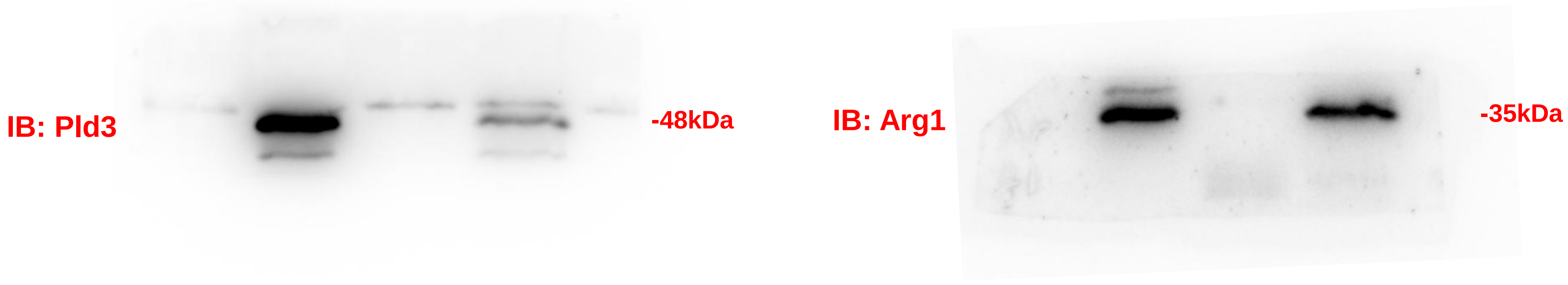

Figure S15B

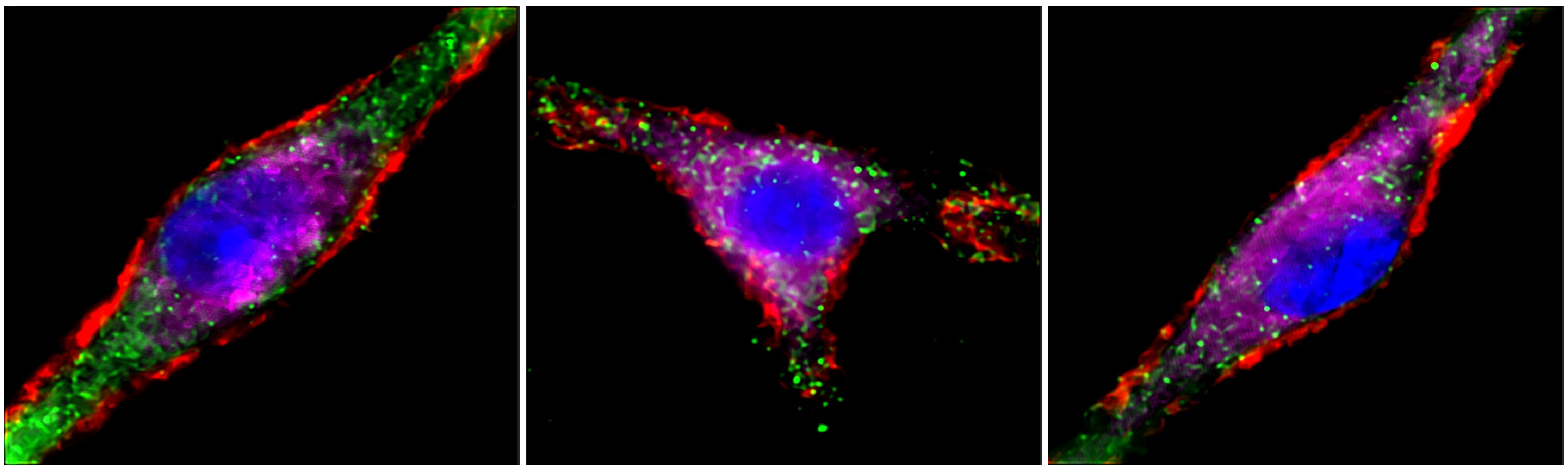

Figure S15C，(1) Whole cell, (2) Nucleus, (3) Cytoplasm

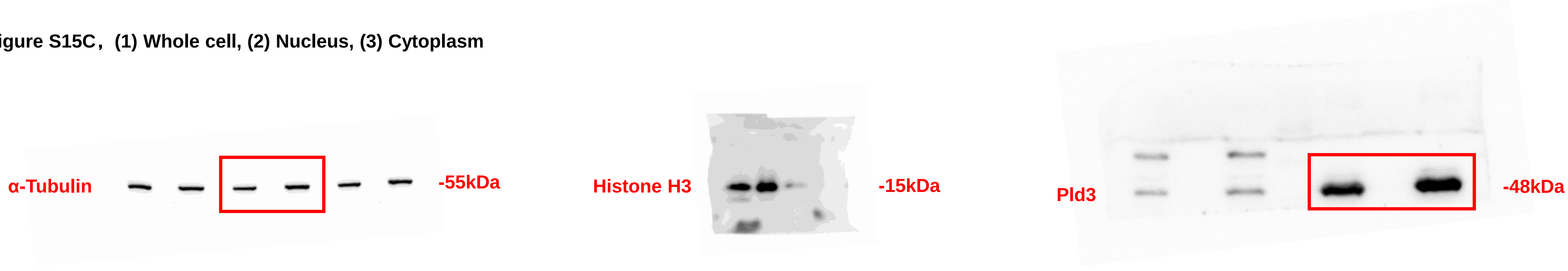

Figure S16D，(1) RAW264.7, (2) RAW+10μM Abrine, (3) RAW+40μM Abrine

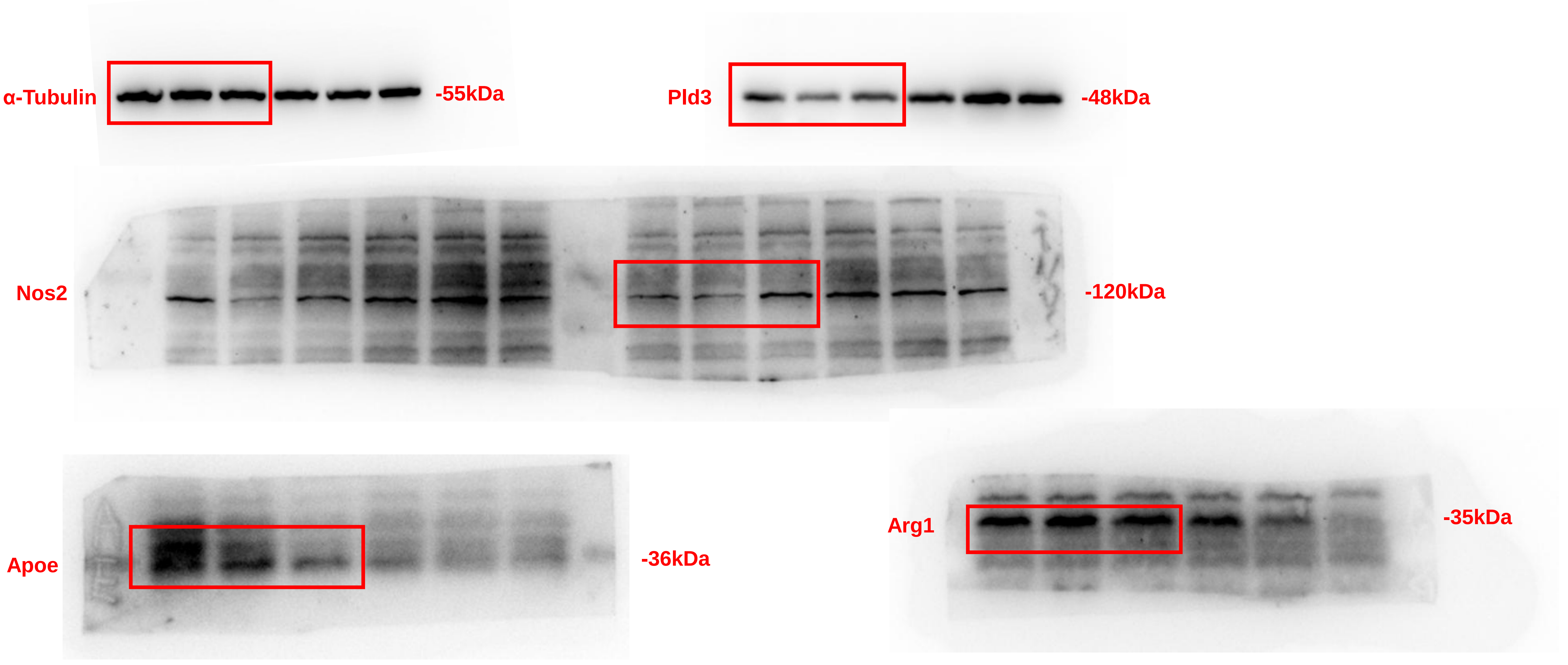

Figure S16G

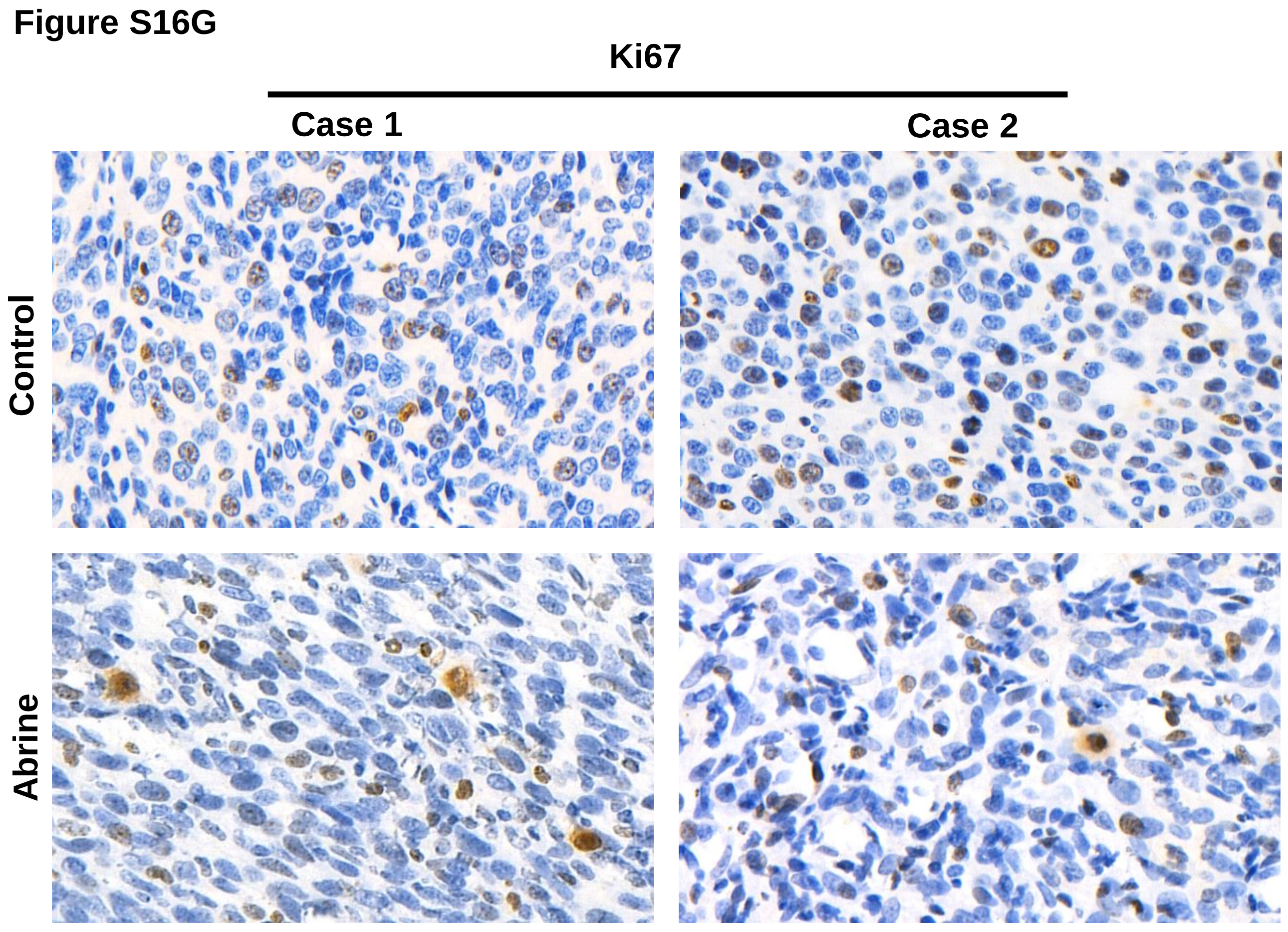

Figure S16I

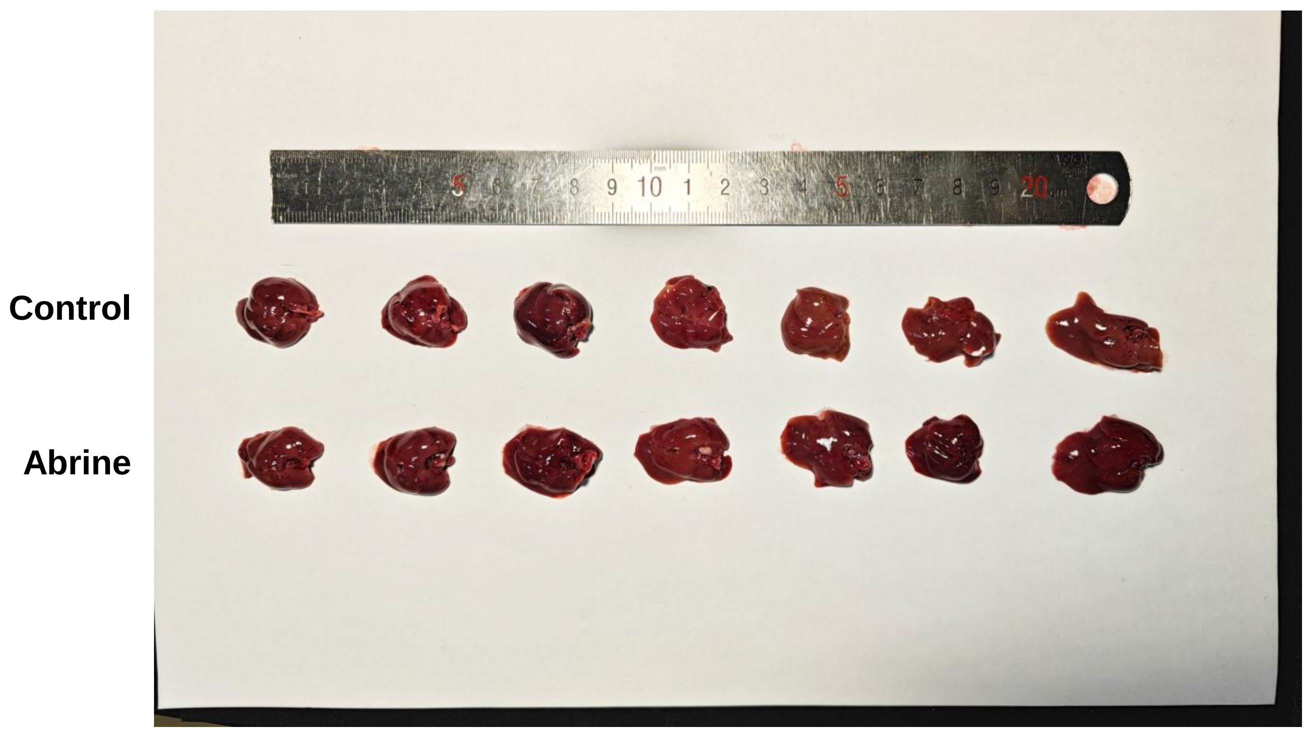

Figure S16J

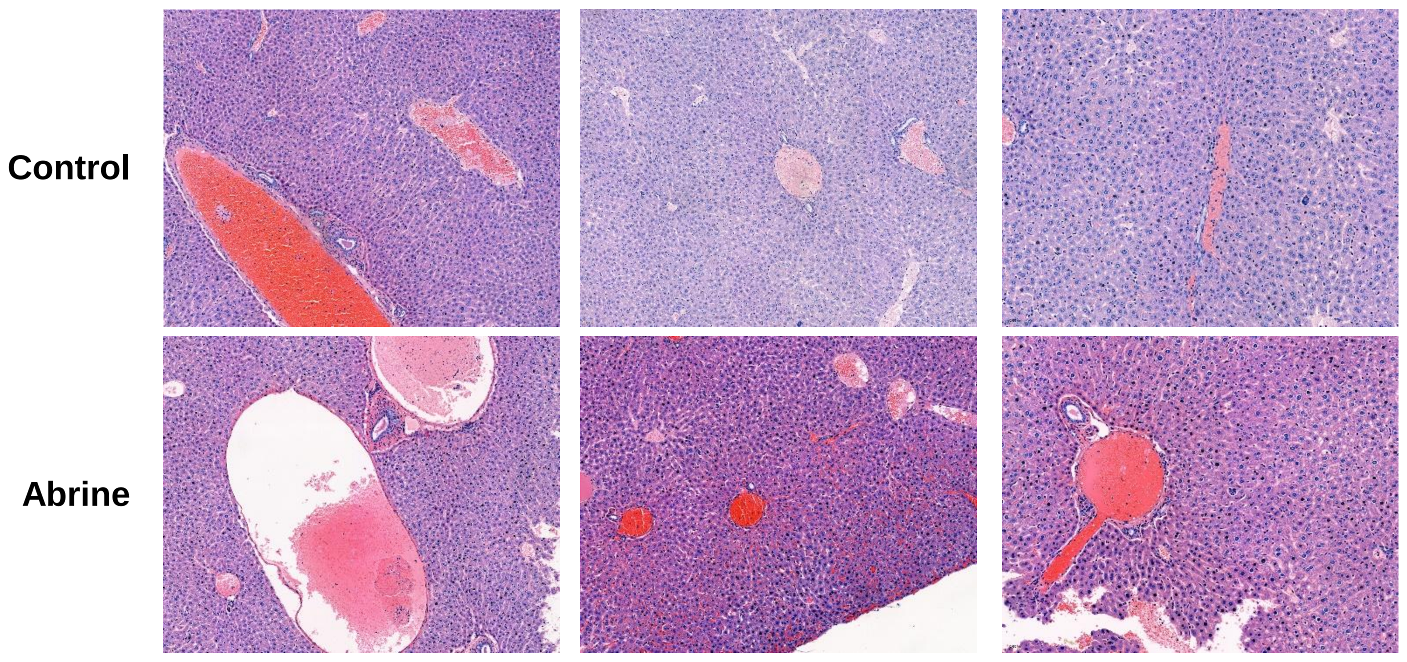

Figure S16K

Control

Abrine

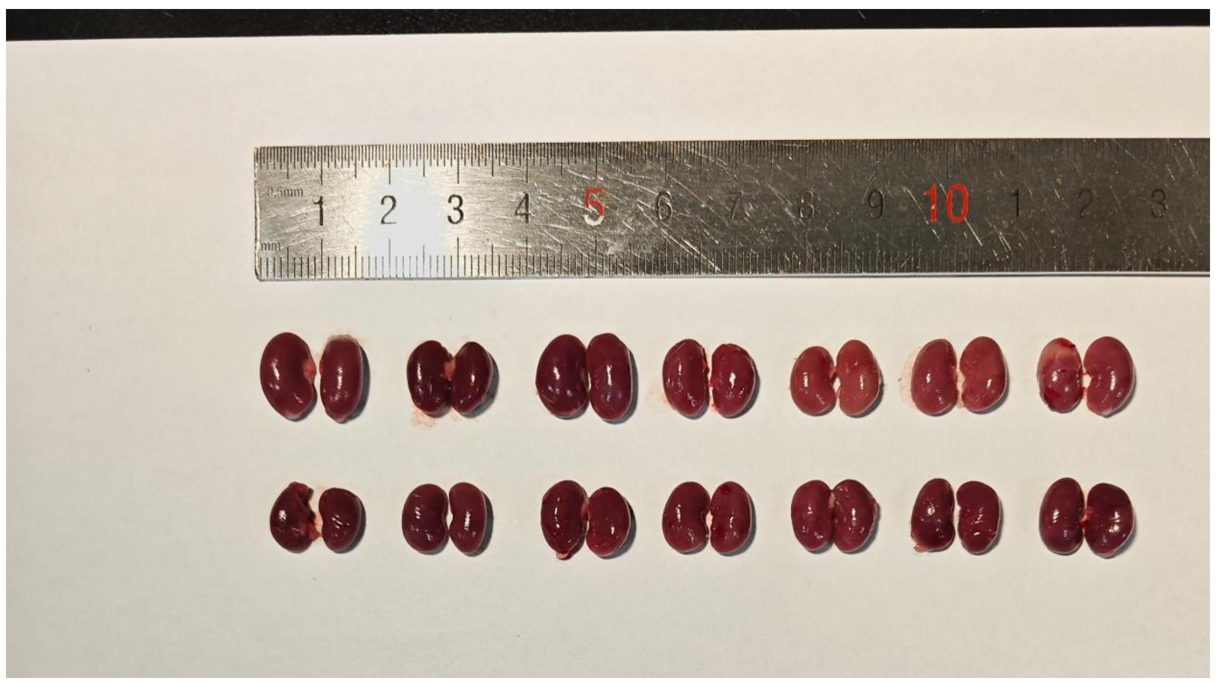

Figure S16L

Control

Abrine

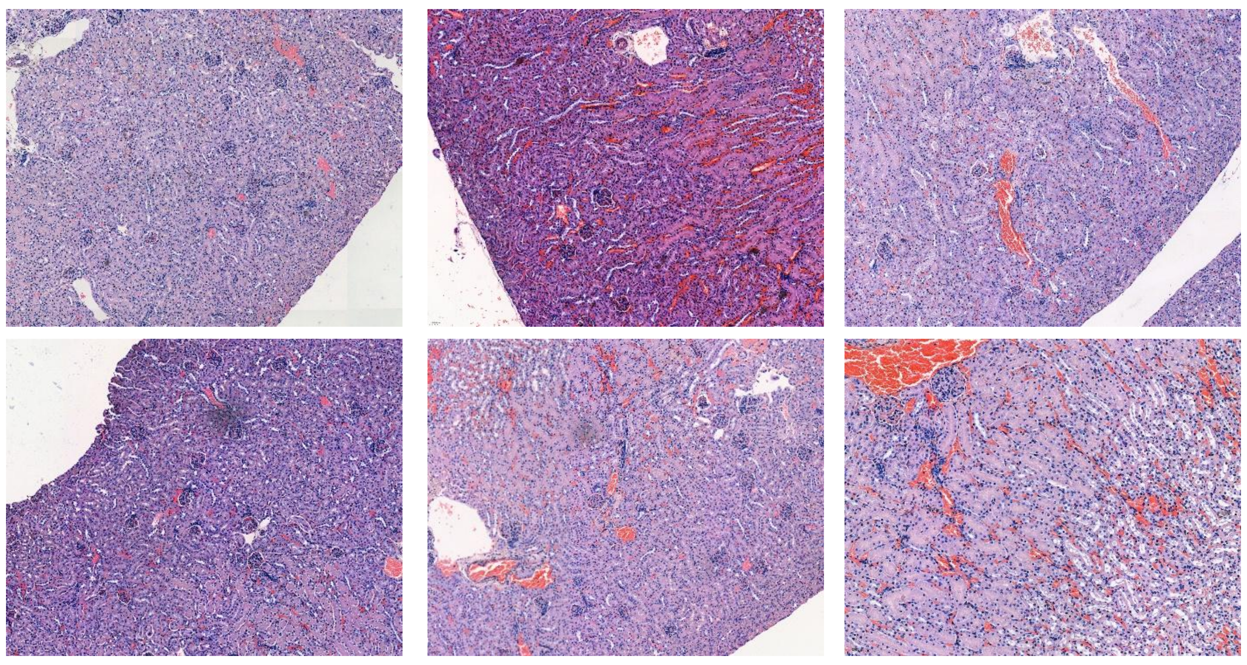

Figure S16M

Control

Abrine

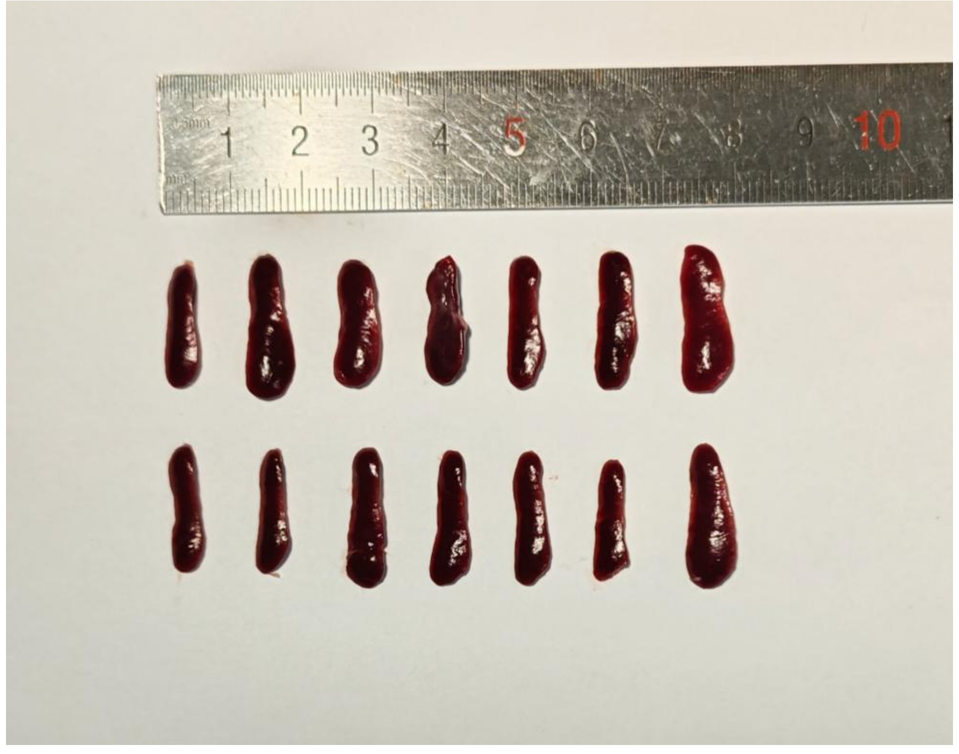

Figure S16N

Control

Abrine

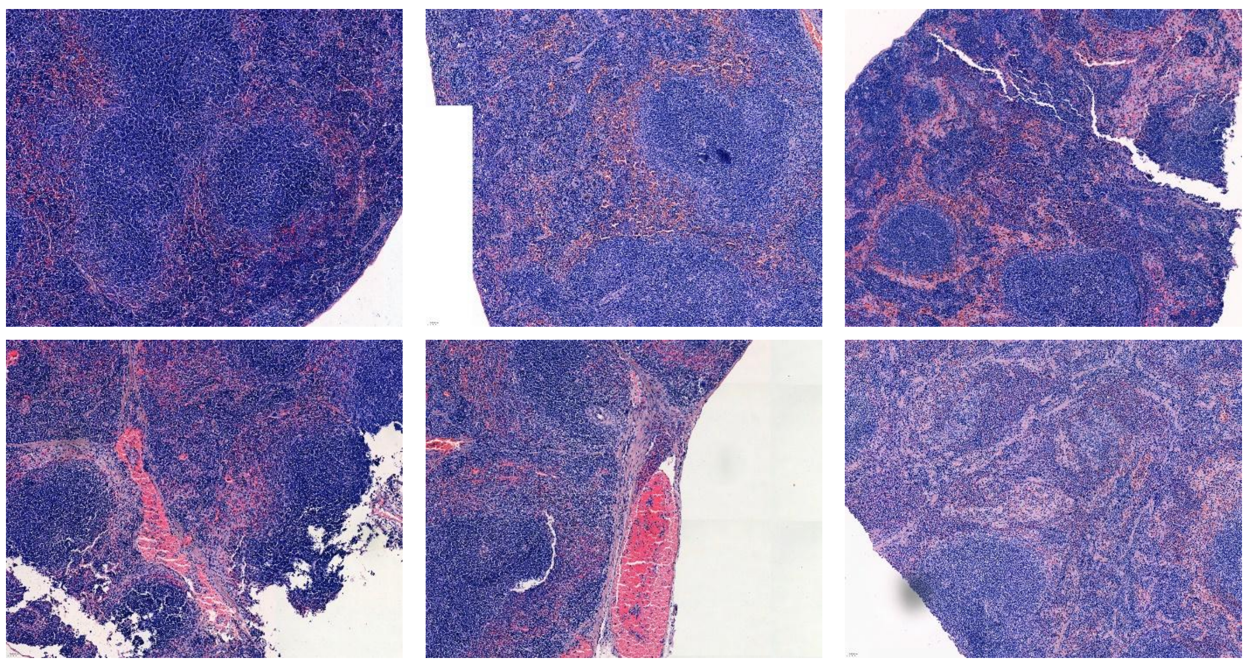

Figure S18A

Pld3$^{flox/flox}$

Pld3$^{\Delta MKO}$

Pld3$^{flox/flox}$ + αPD-1

Pld3$^{\Delta MKO}$ + αPD-1

Pld3$^{flox/flox}$ + Abrine

Pld3$^{\Delta MKO}$ + Abrine

Pld3$^{flox/flox}$ + Abrine + αPD-1

Pld3$^{\Delta MKO}$ + Abrine + αPD-1

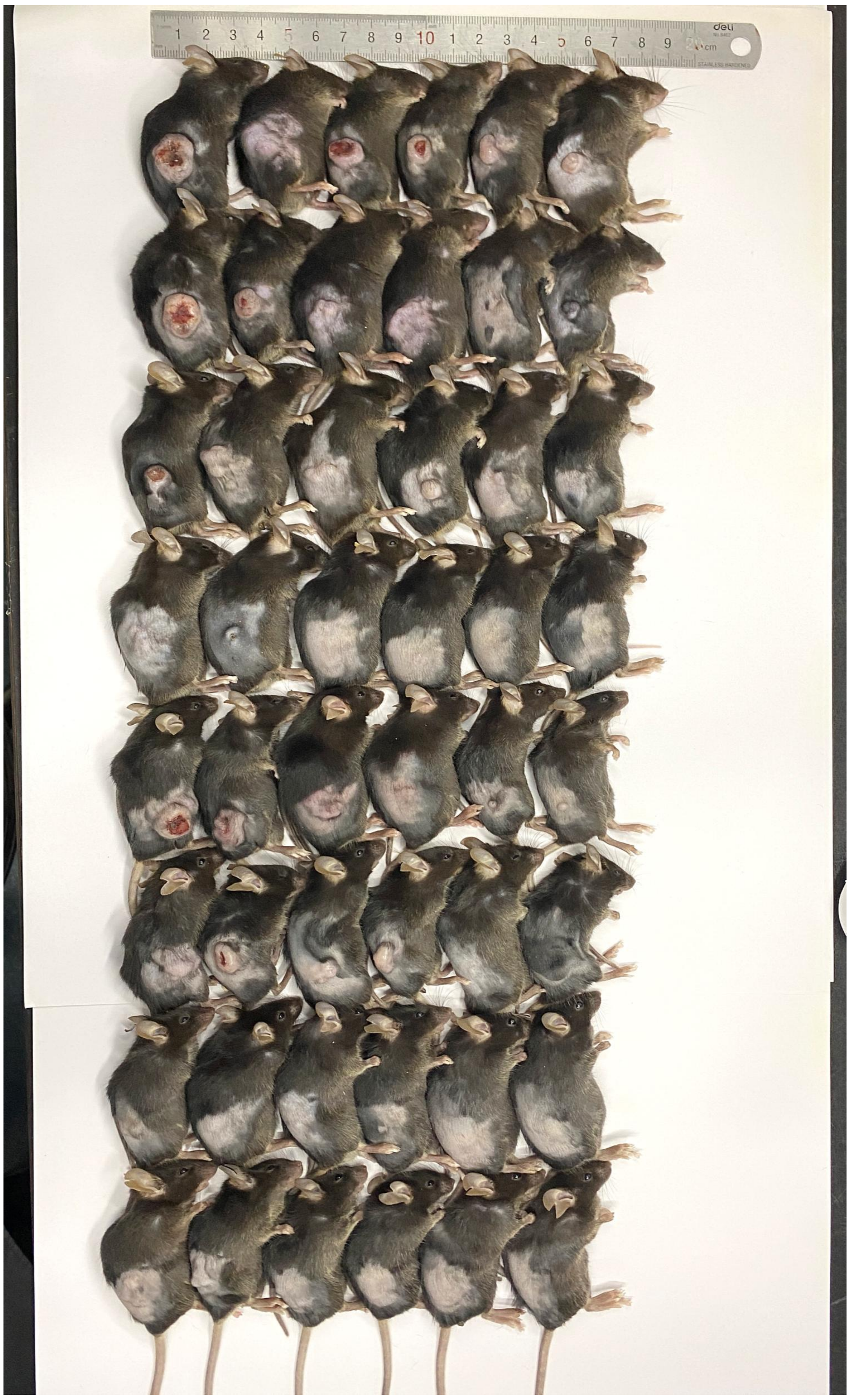

Figure S18C

Pld3$^{flox/flox}$

Pld3$^{\Delta MKO}$

Pld3$^{flox/flox}$ + αPD-1

Pld3$^{\Delta MKO}$ + αPD-1

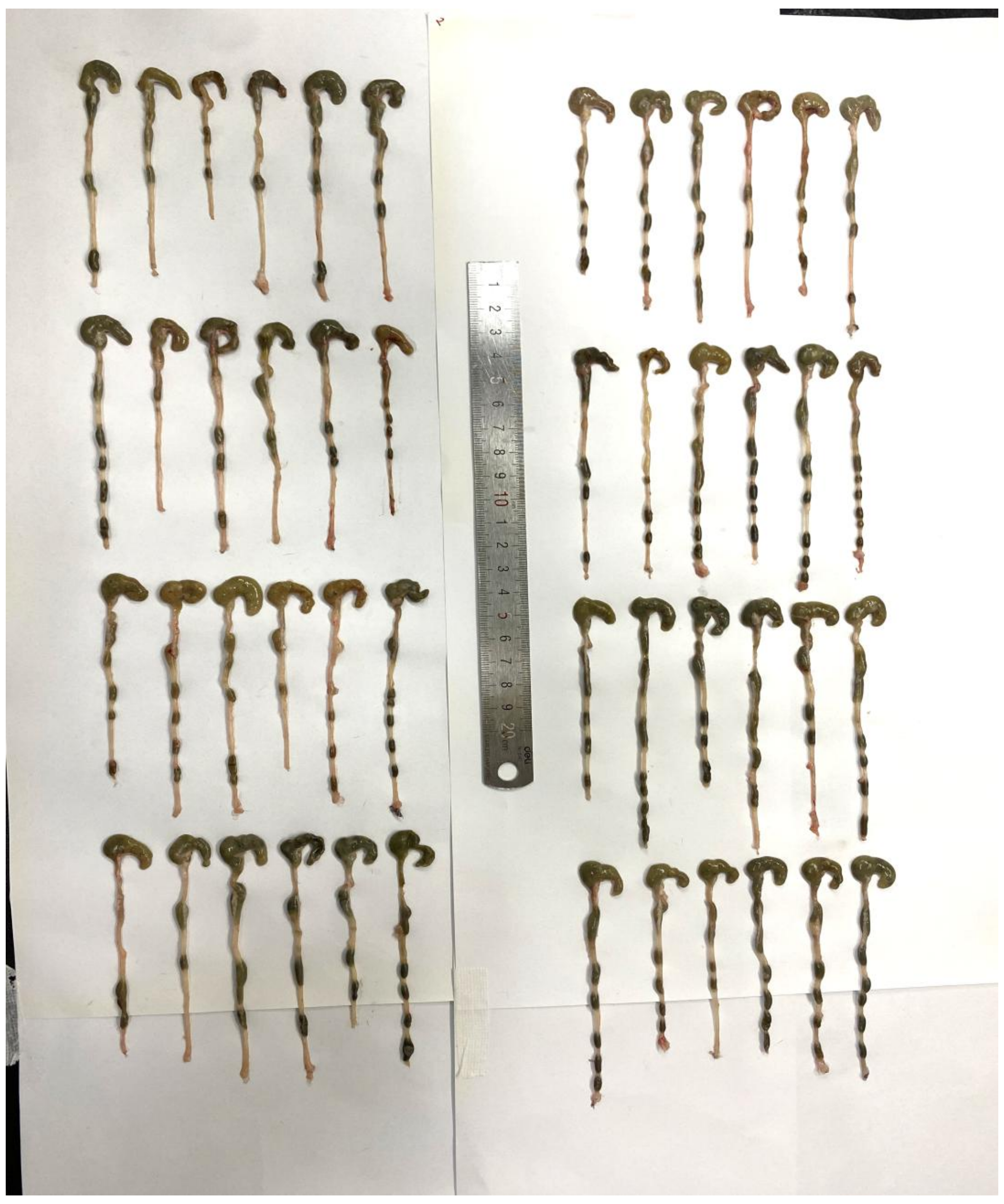

Pld3$^{flox/flox}$ + Abrine

Pld3$^{\Delta MKO}$ + Abrine

Pld3$^{flox/flox}$ + Abrine + αPD-1

Pld3$^{\Delta MKO}$ + Abrine+ αPD-1

Figure S18E

Pld3$^{flox/flox}$

Pld3$^{\Delta MKO}$

Pld3$^{flox/flox}$ + αPD-1

Pld3$^{\Delta MKO}$ + αPD-1

Pld3$^{flox/flox}$ + Abrine

Pld3$^{\Delta MKO}$ + Abrine

Pld3$^{flox/flox}$ + Abrine + αPD-1

Pld3$^{\Delta MKO}$ + Abrine + αPD-1

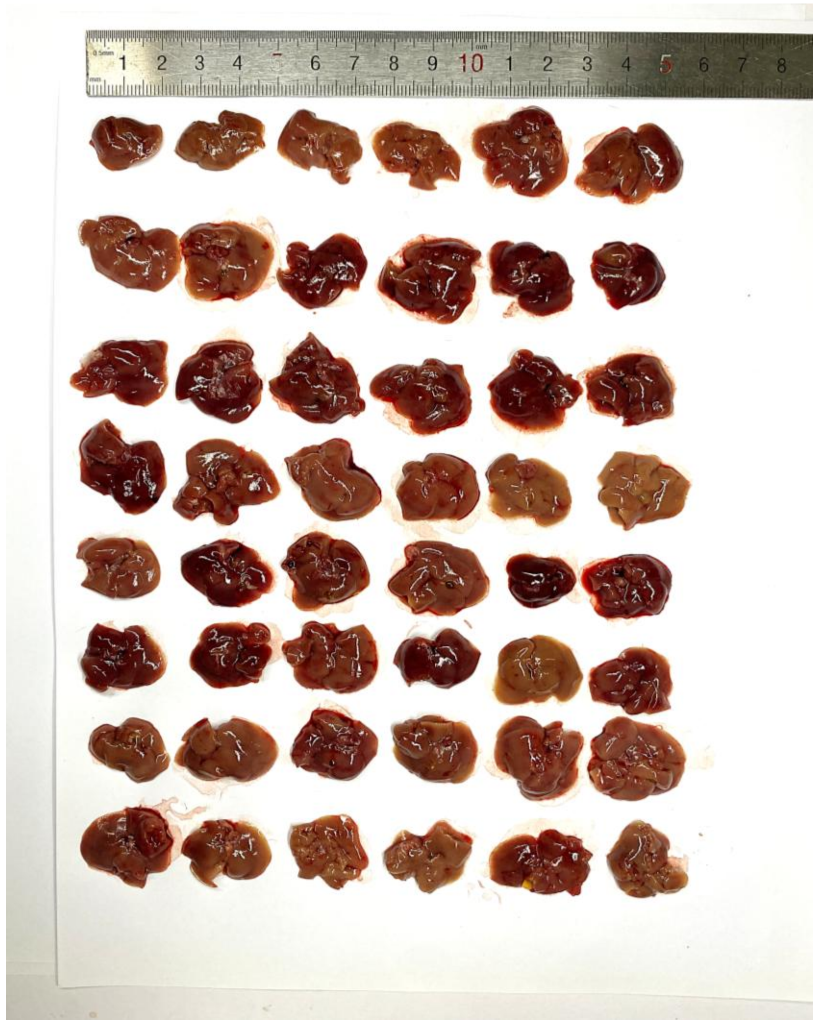

Figure S18F

Pld3$^{flox/flox}$

Pld3$^{\Delta MKO}$

Pld3$^{flox/flox}$ + αPD-1

Pld3$^{\Delta MKO}$ + αPD-1

Pld3$^{flox/flox}$ + Abrine

Pld3$^{\Delta MKO}$ + Abrine

Pld3$^{flox/flox}$ + Abrine + αPD-1

Pld3$^{\Delta MKO}$ + Abrine + αPD-1

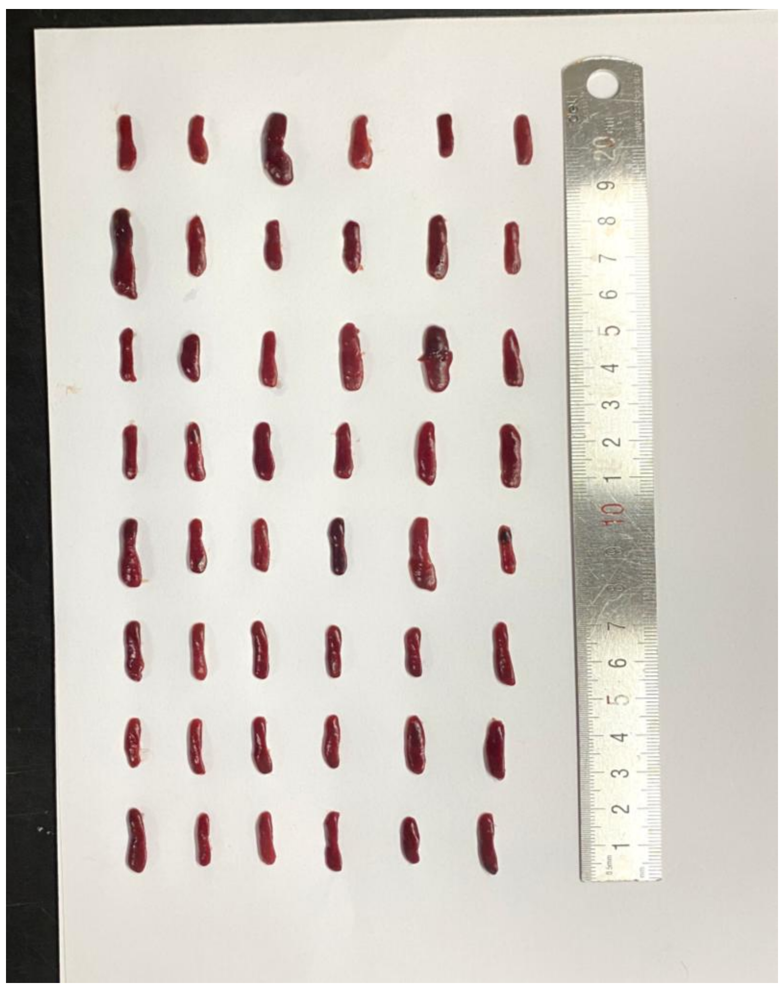

Figure S18G

Pld3$^{flox/flox}$

Pld3$^{\Delta MKO}$

Pld3$^{flox/flox}$ + αPD-1

Pld3$^{\Delta MKO}$ + αPD-1

Pld3$^{flox/flox}$ + Abrine

Pld3$^{\Delta MKO}$ + Abrine

Pld3$^{flox/flox}$ + Abrine + αPD-1

Pld3$^{\Delta MKO}$ + Abrine + αPD-1

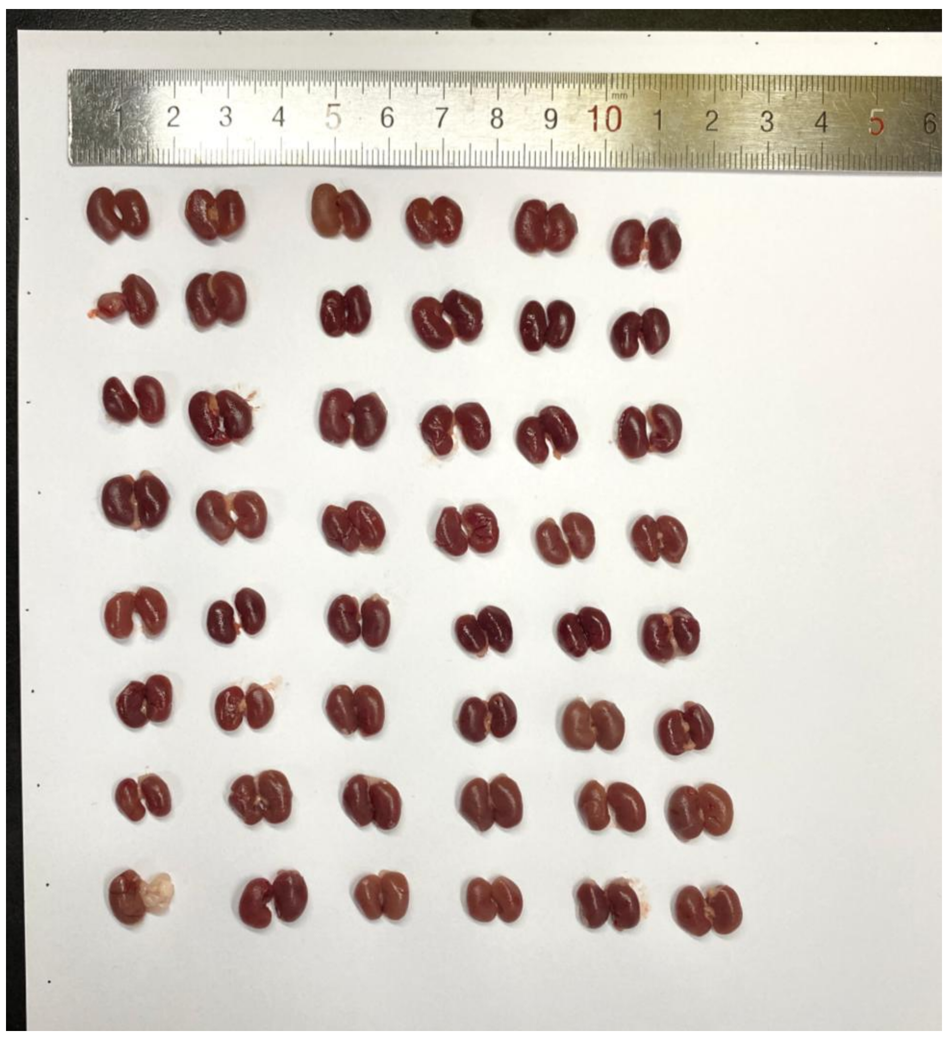

Figure S18I

Pld3$^{flox/flox}$ | Pld3$^{\Delta MKO}$ | Pld3$^{flox/flox}$ + αPD-1 | Pld3$^{\Delta MKO}$ + αPD-1 | Pld3$^{flox/flox}$ + Abrine | Pld3$^{\Delta MKO}$ + Abrine | Pld3$^{flox/flox}$+Abrine + αPD-1 | Pld3$^{\Delta MKO}$+Abrine + αPD-1

Liver

Kidney

Spleen

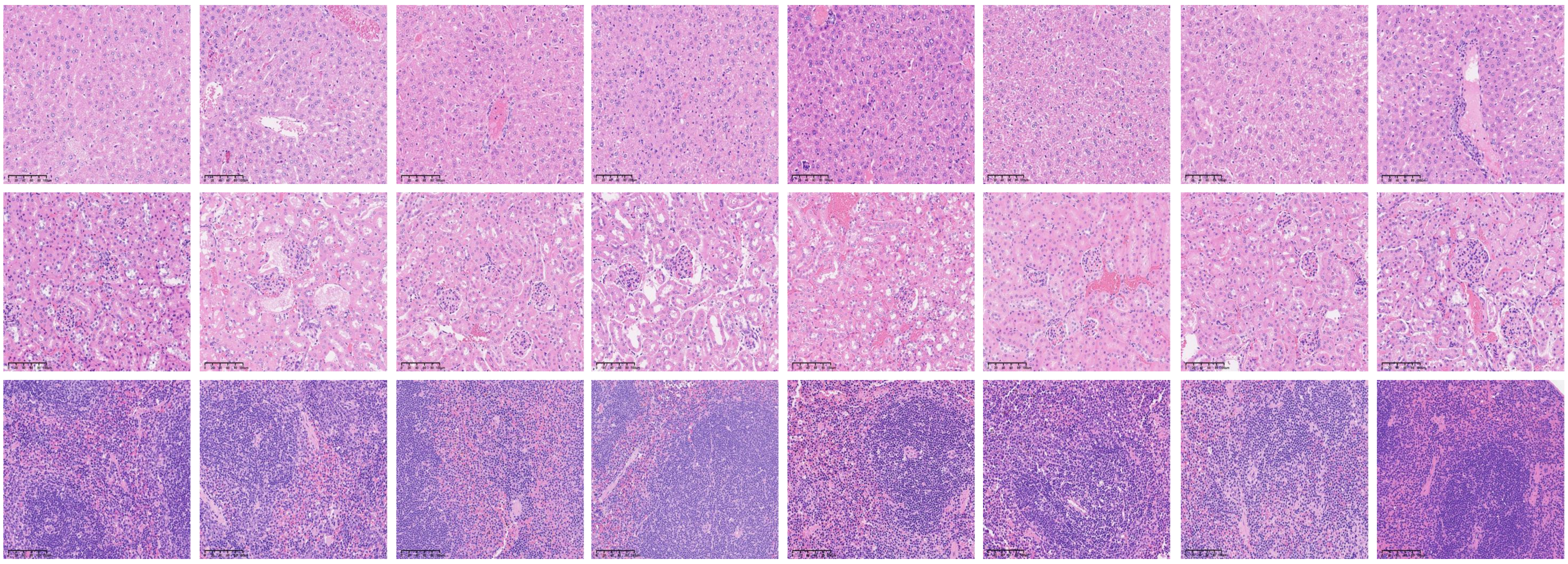

Figure S19A

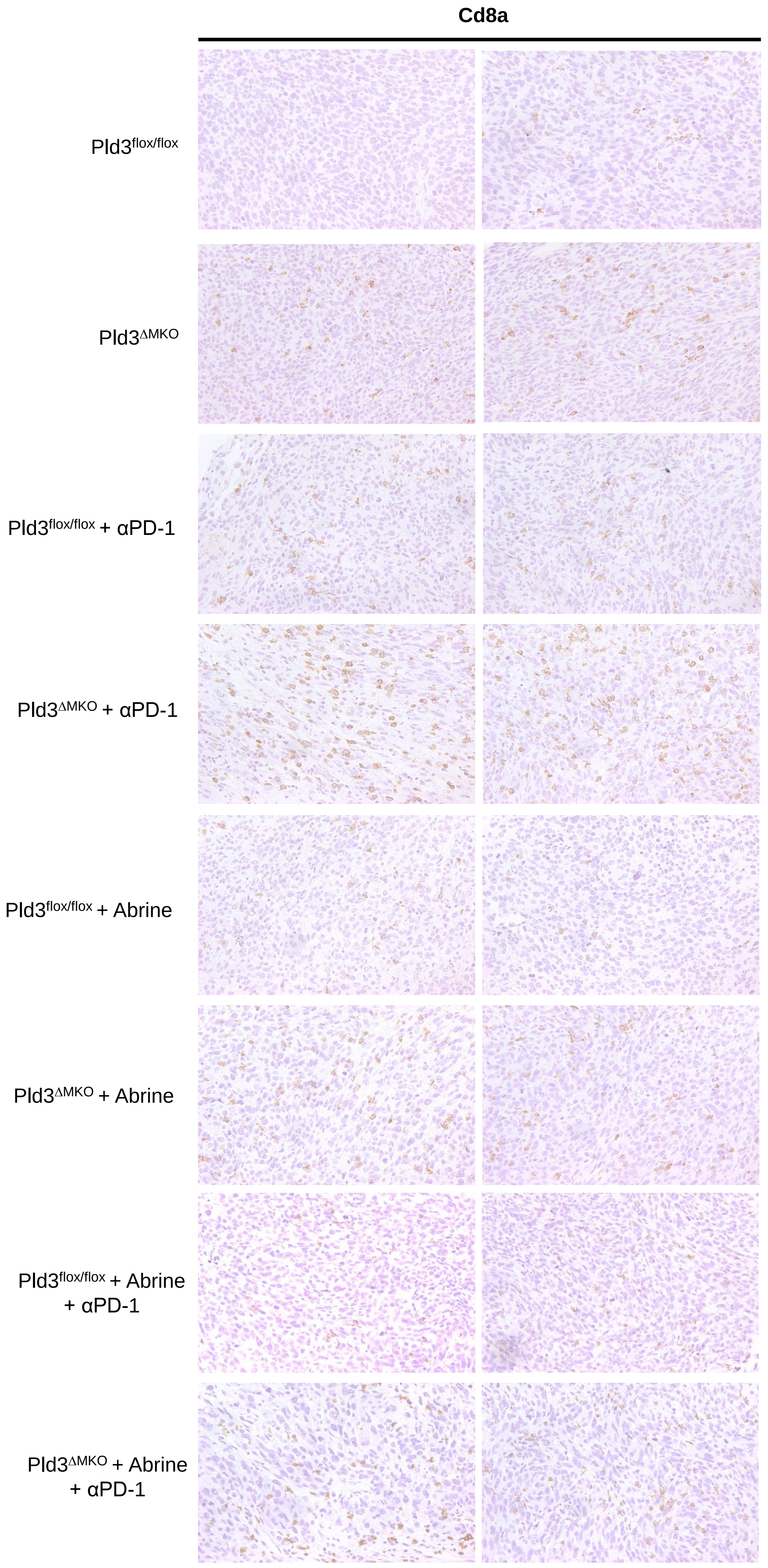

Figure S19B

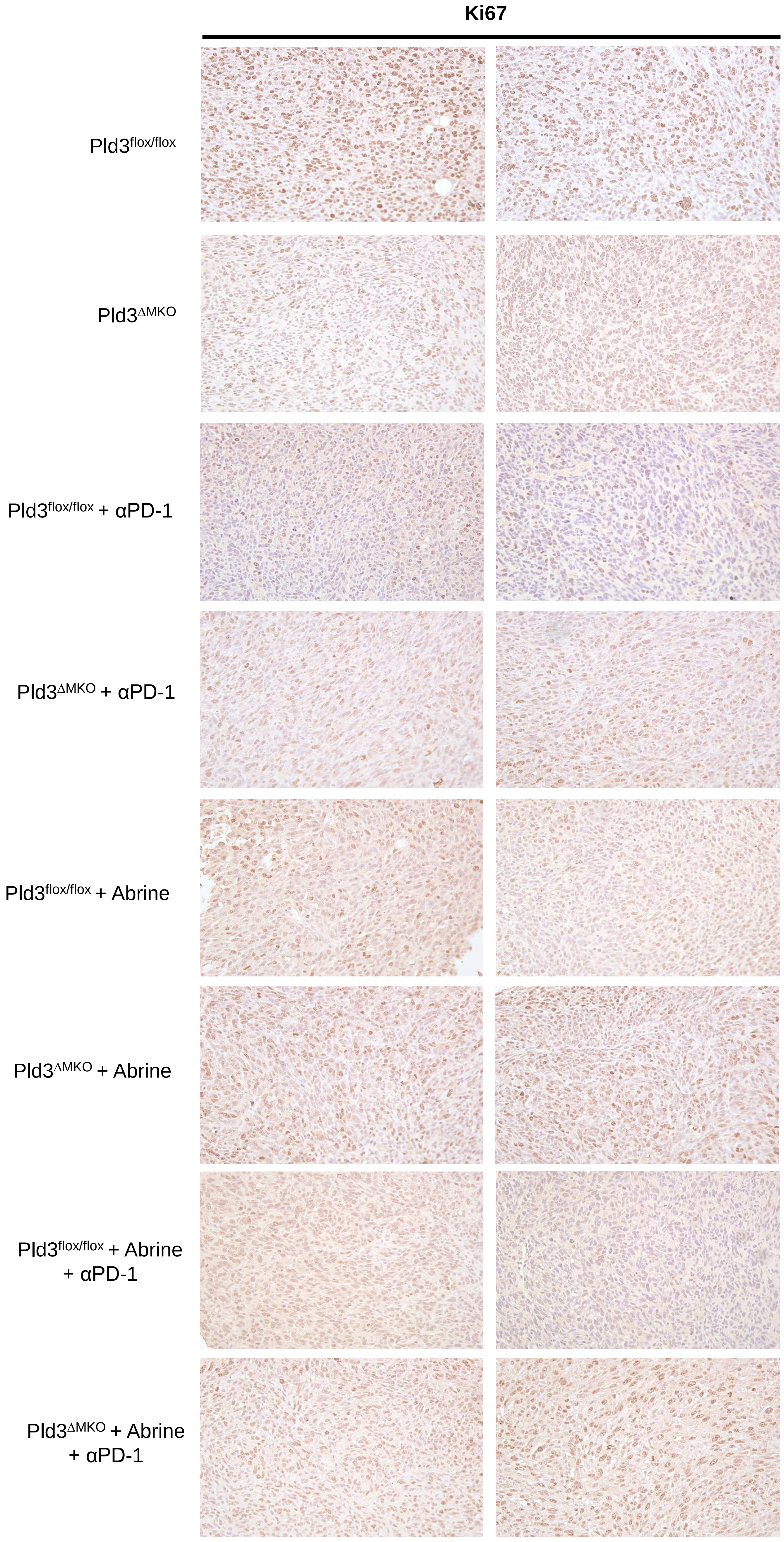

Figure S19C

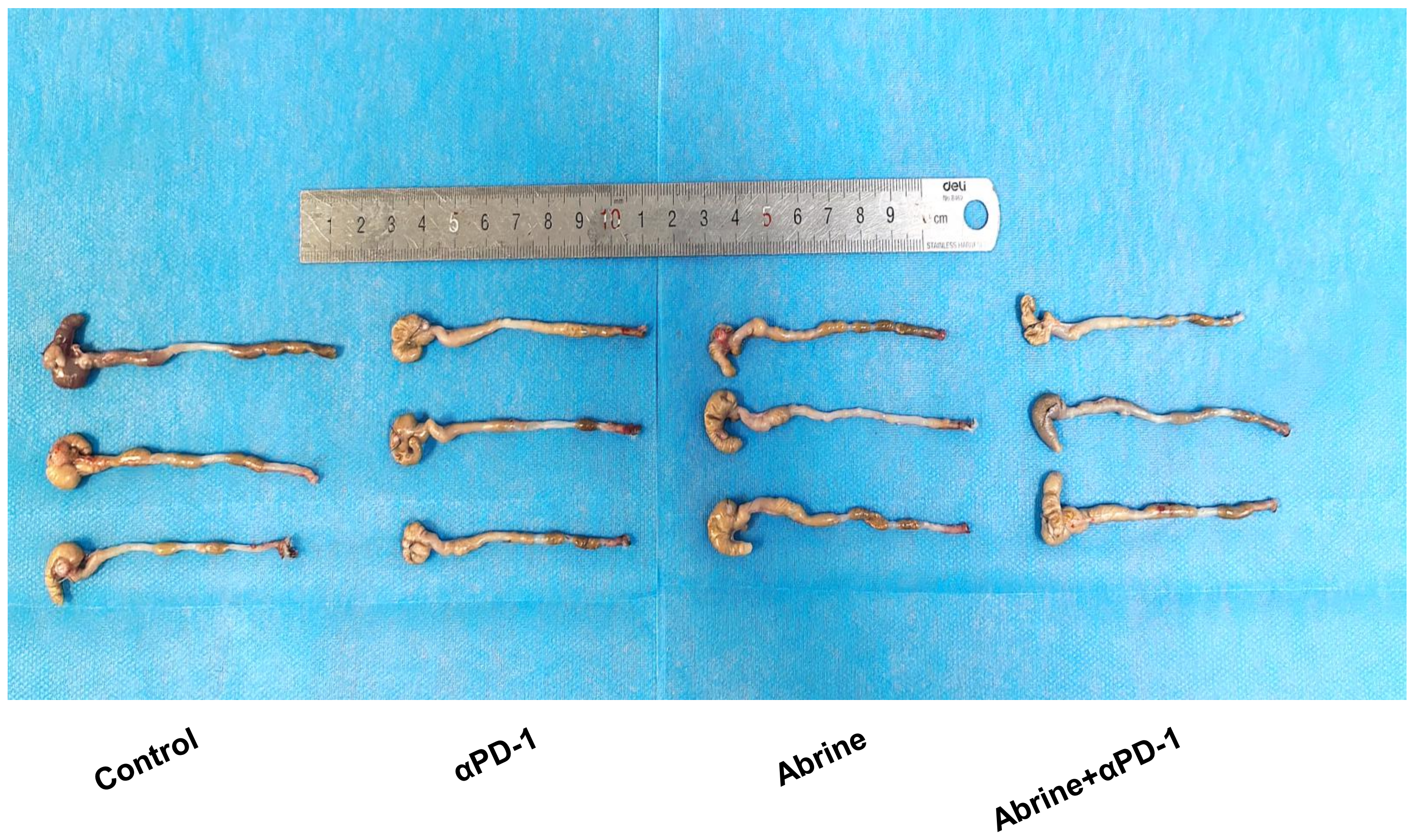

Figure S19D

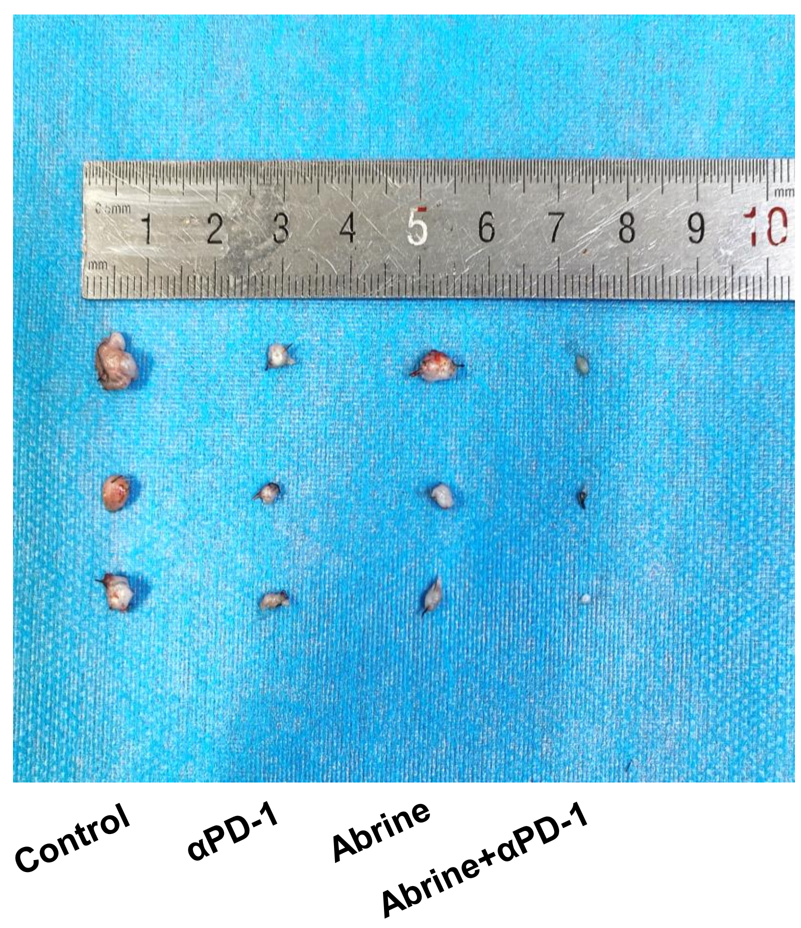

Supplement: Supplementary file 2 — Supporting File 2: advs75730‐sup‐0002‐Data.zip. [file ADVS-9999-e75730-s001.zip › advs75730-sup-0002-Data/Raw data.pdf]
