# Supplementary material for: Targeting PLD3 Reverses the Immunosuppressive Niche by Reprogramming Tumor‐Associated Macrophages and Potentiates Antitumor Immunity
Source: Adv Sci (Weinh). 2026 May 21:e75730. Online ahead of print. doi: 10.1002/advs.75730 (PMC13335905; doi:10.1002/advs.75730)

Figure S1. PLD3+Macro predicts CRC immunotherapy outcomes

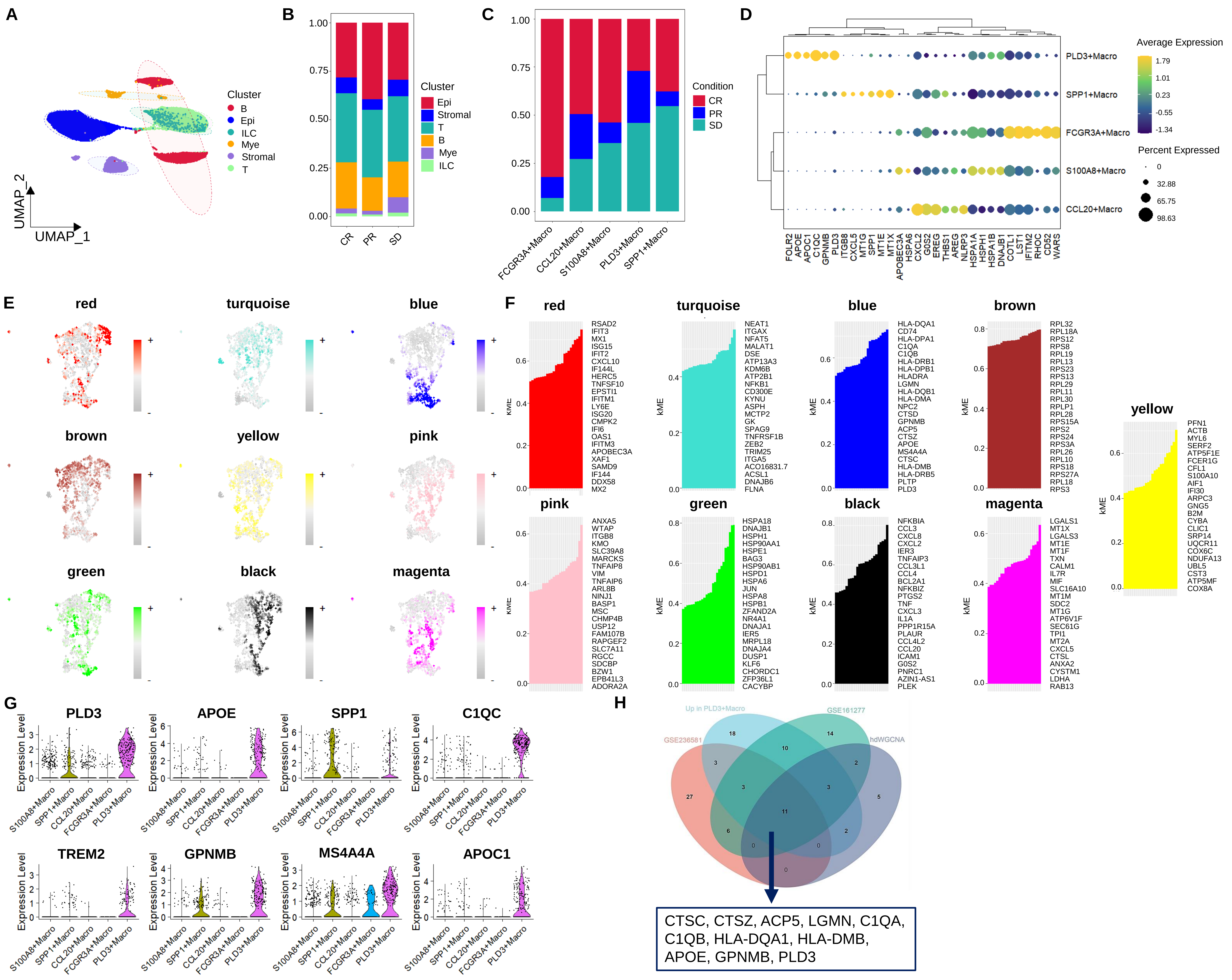

I

Functional Module	Genes Involved	Core Biological Processes
Lysosomal Core Enzymes	CTSC, CTSZ, ACP5, LGMN	Protein degradation, Antigen processing, Lysosomal activation
Complement System	C1QA, C1QB	Immune recognition, Apoptotic cell clearance, Synaptic pruning
Antigen Presentation	HLA-DQA1, HLA-DMB	MHC class II molecules, T cell activation, Immune regulation
Lipid Metabolism	APOE	Lipid transport, Cholesterol efflux, Lipid-associated macrophages
Transmembrane Signaling	GPNMB, PLD3	Cell adhesion, Tissue remodeling, Neuroinflammation

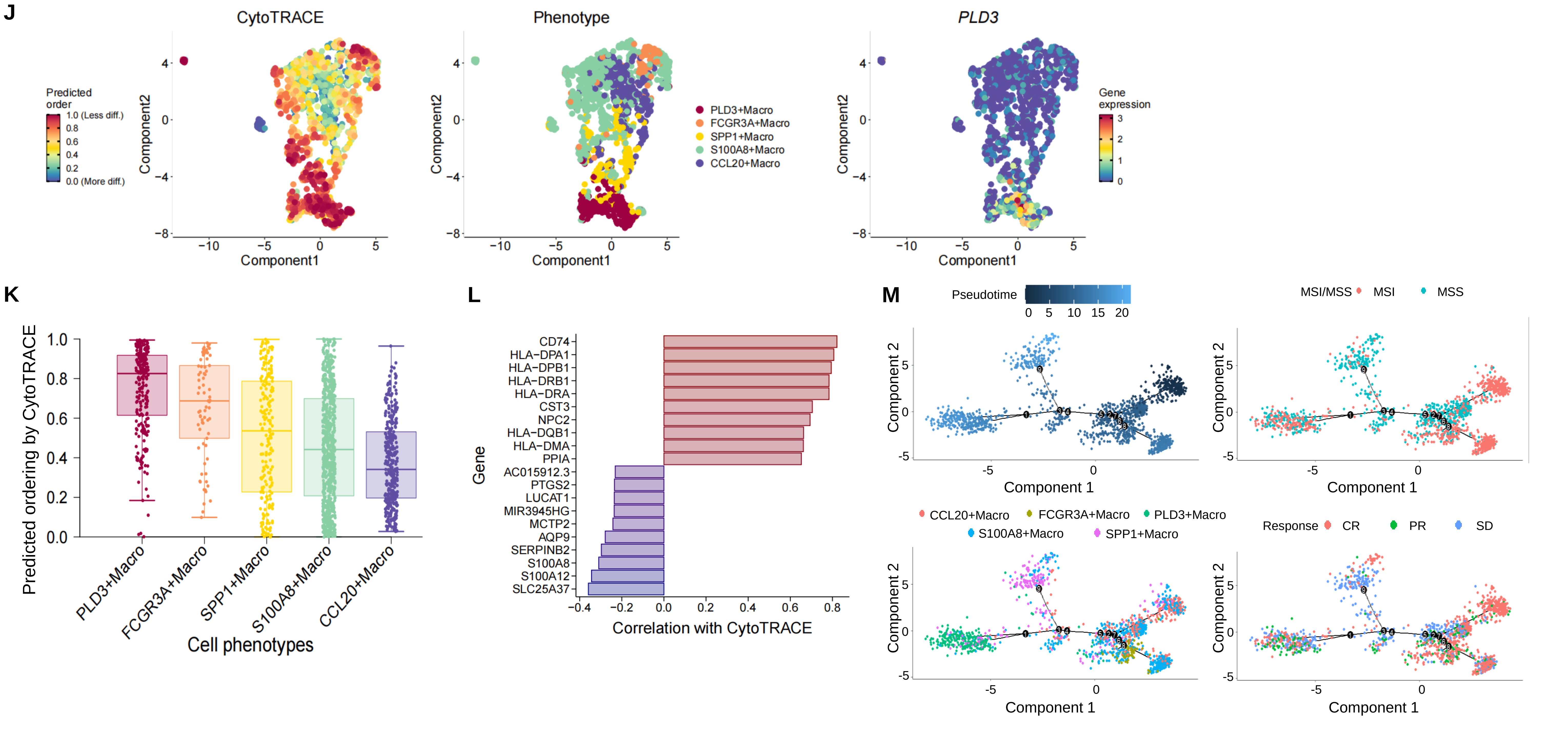

Figure S2. Spatial infiltration dynamics of PLD3+Macro dictate immunotherapy outcomes in colorectal cancer

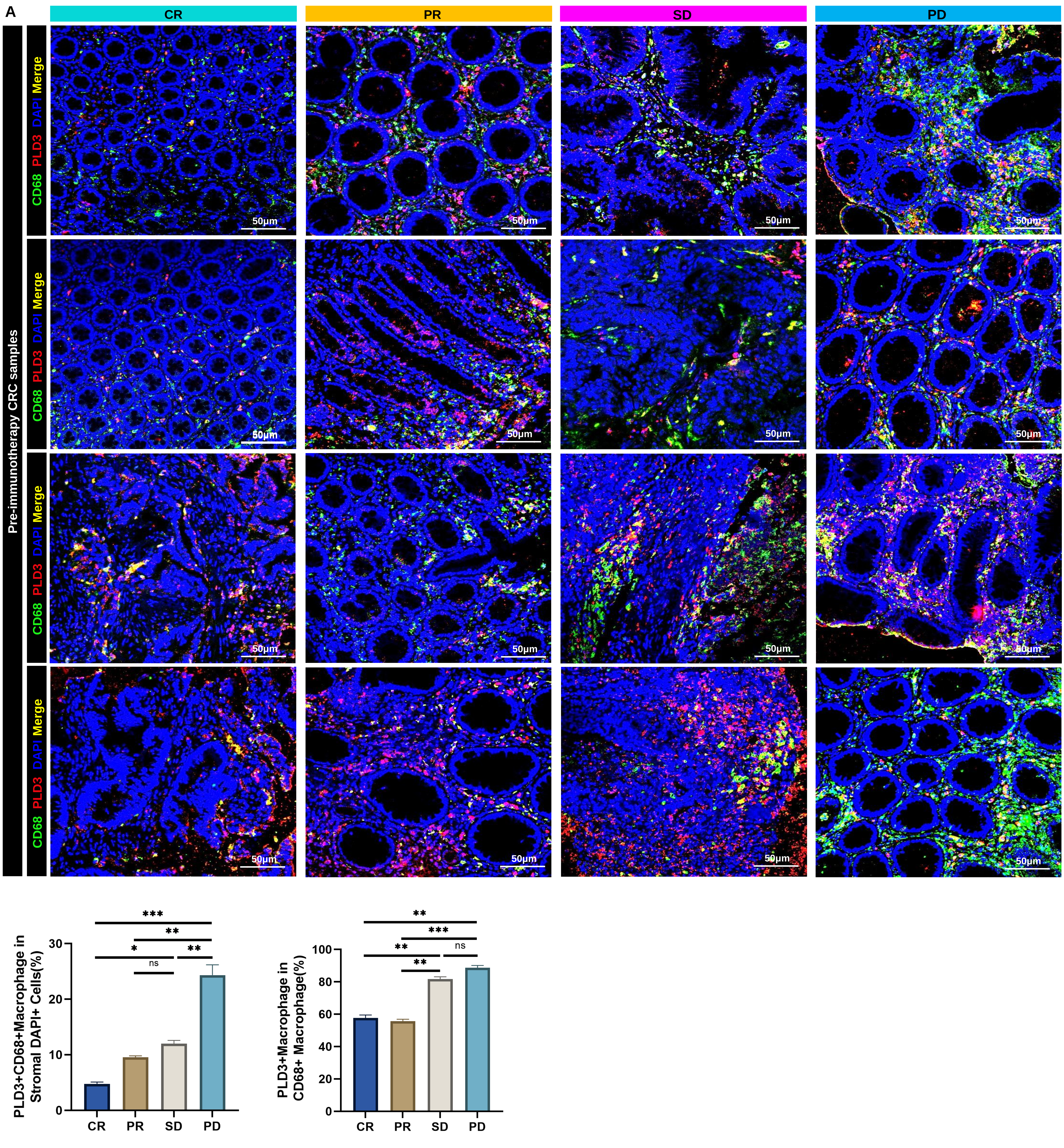

Figure S3. Single-cell sequencing validation of PLD3+Macro in colorectal cancer

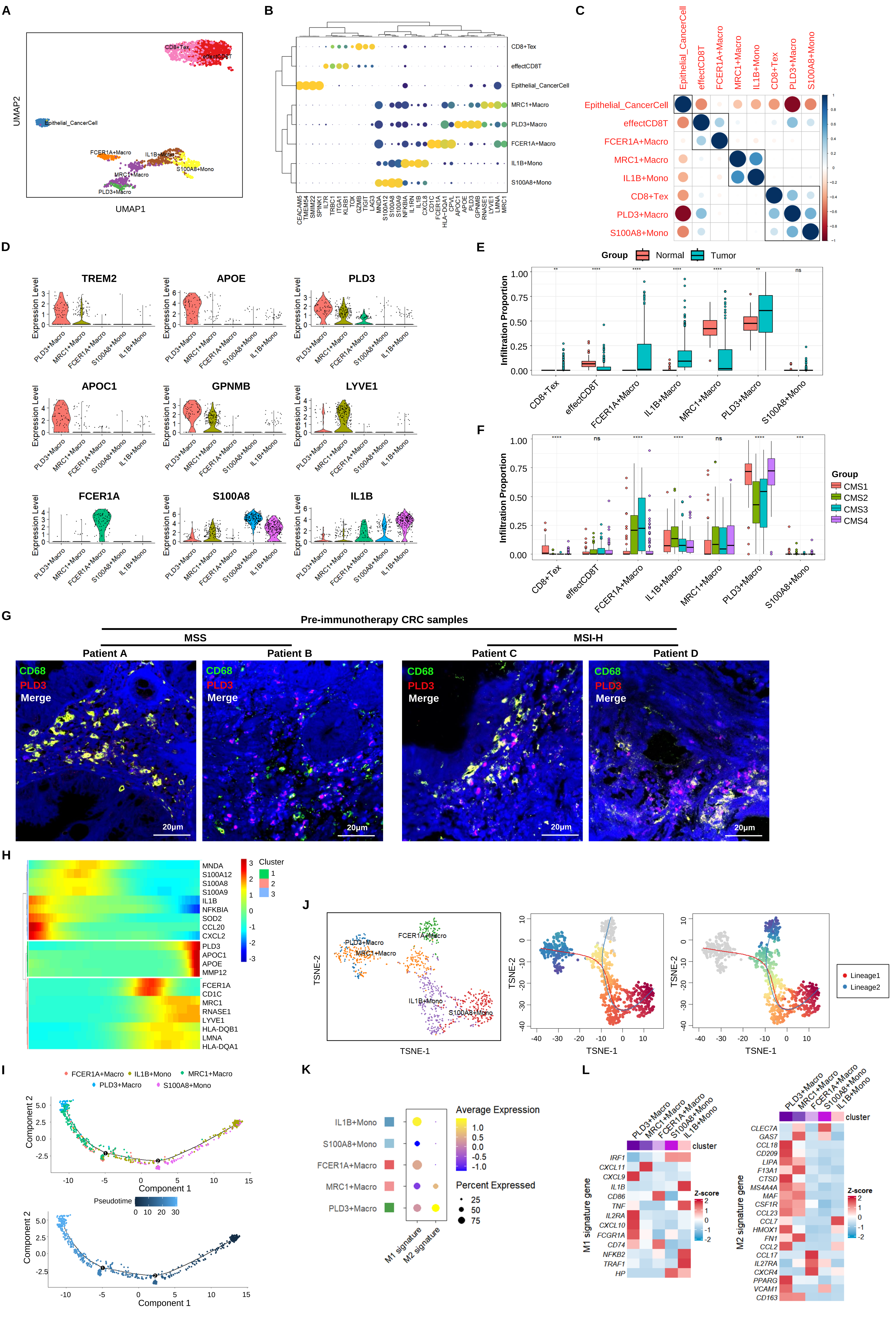

Figure S4. TME promotes PLD3 expression in macrophages correlating positively with TAMs markers

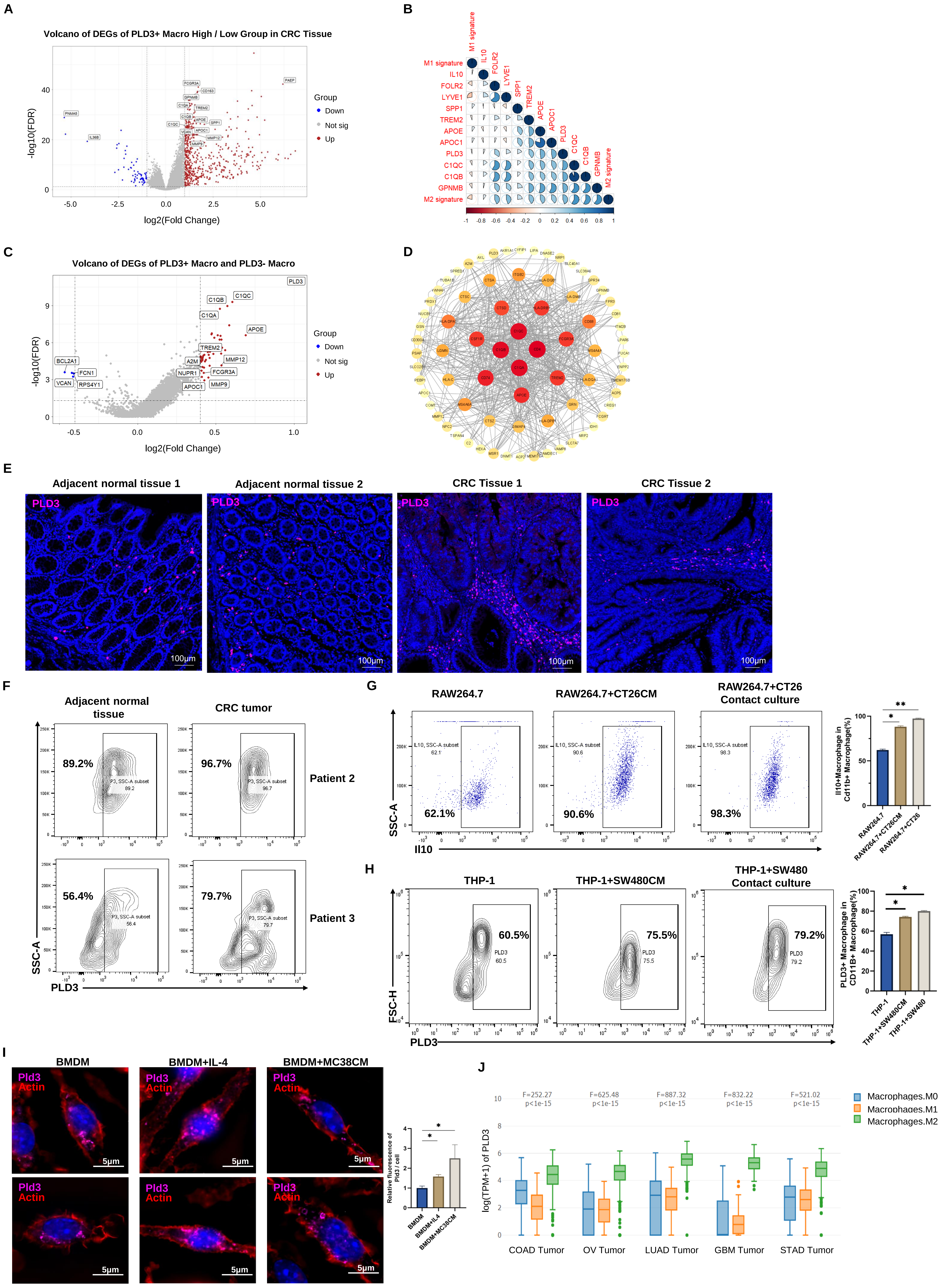

Figure S5. Construction of myeloid-specific Pld3 knockout mouse model and scRNA-seq analysis of subcutaneous tumors

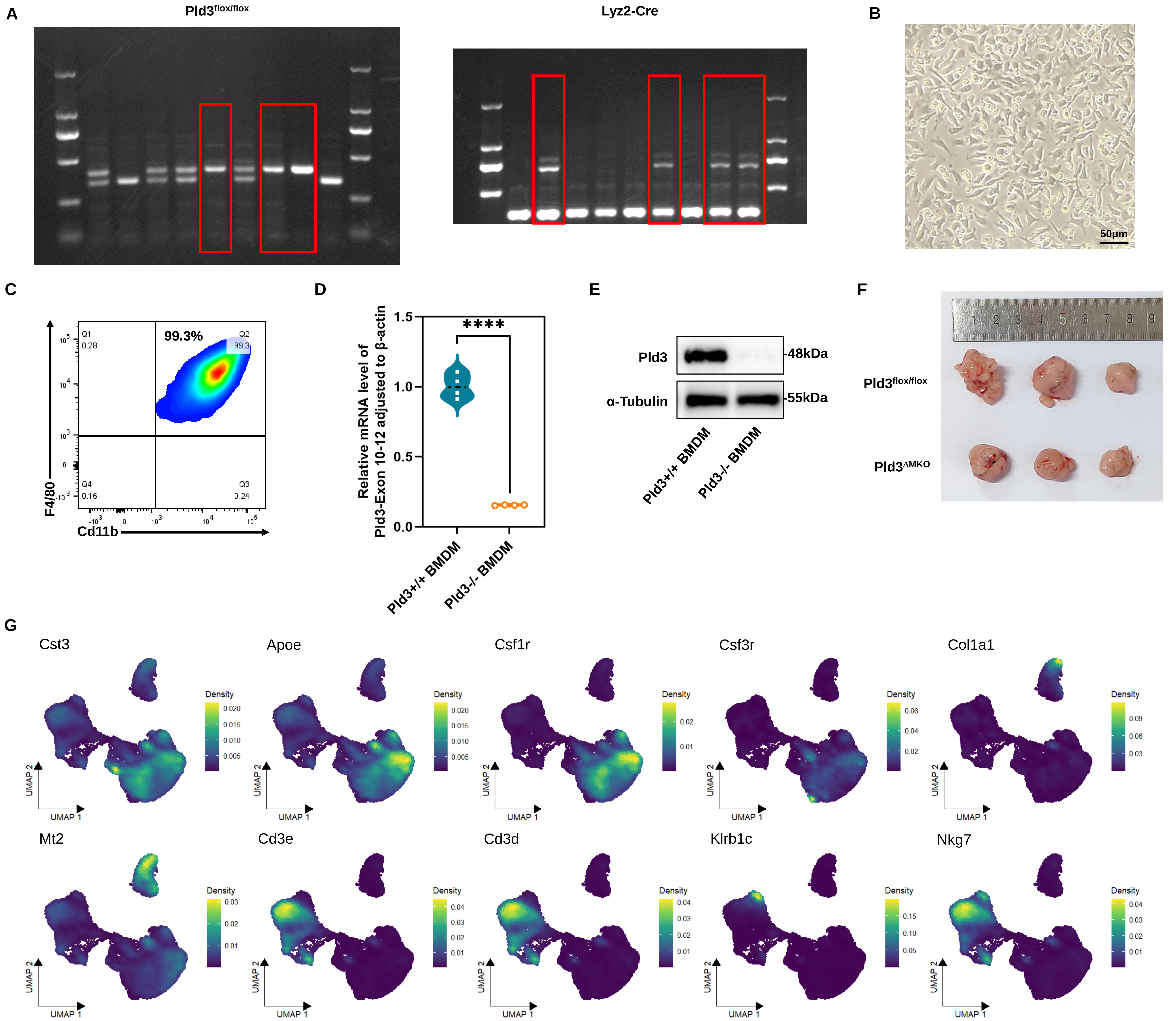

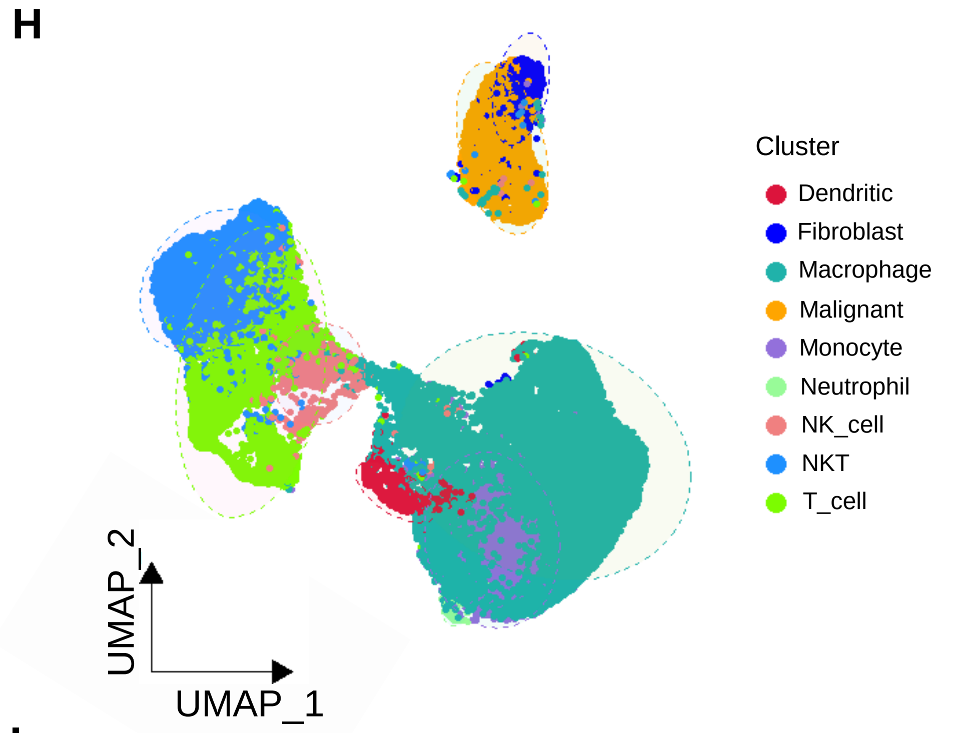

I

Celltype	Markers
Malignant	Ankrd1, Mt2, Cavin3, Bgn, Nupr1, Cavin1, Igfbp6
Dendritic	Ccl17, Cst3, Ramp3, Ccr7, Syngr2, Napsa, H2-DMb2
Fibroblast	Col3a1, Dcn, Sparc, Col1a2, Gsn, Bgn, Ccn1
Macrophage	Apoe, Trem2, C1qc, Ctsb, Lyz2, Arg1, Cd68
Monocyte	Il1b, Cd14, Cxcl2, H2-Ab1, H2-Aa, Cd74, H2-Eb1
Neutrophil	S100a9, Retnlg, Lcn2, G0s2, Cxcr2, Ly6g, Csf3r
NK_cell	Tmsb4x, Malat1, Rpl10, Fth1, Tpt1, B2m, Cox8a
NKT	Nkg7, Gzmb, Cd8b1, Cd3g, Cd8a, Ifng, Klrd1
T_cell	Trac, Sdf4, Icos, Cd3d, Cd3g, Cd4, Cd3e

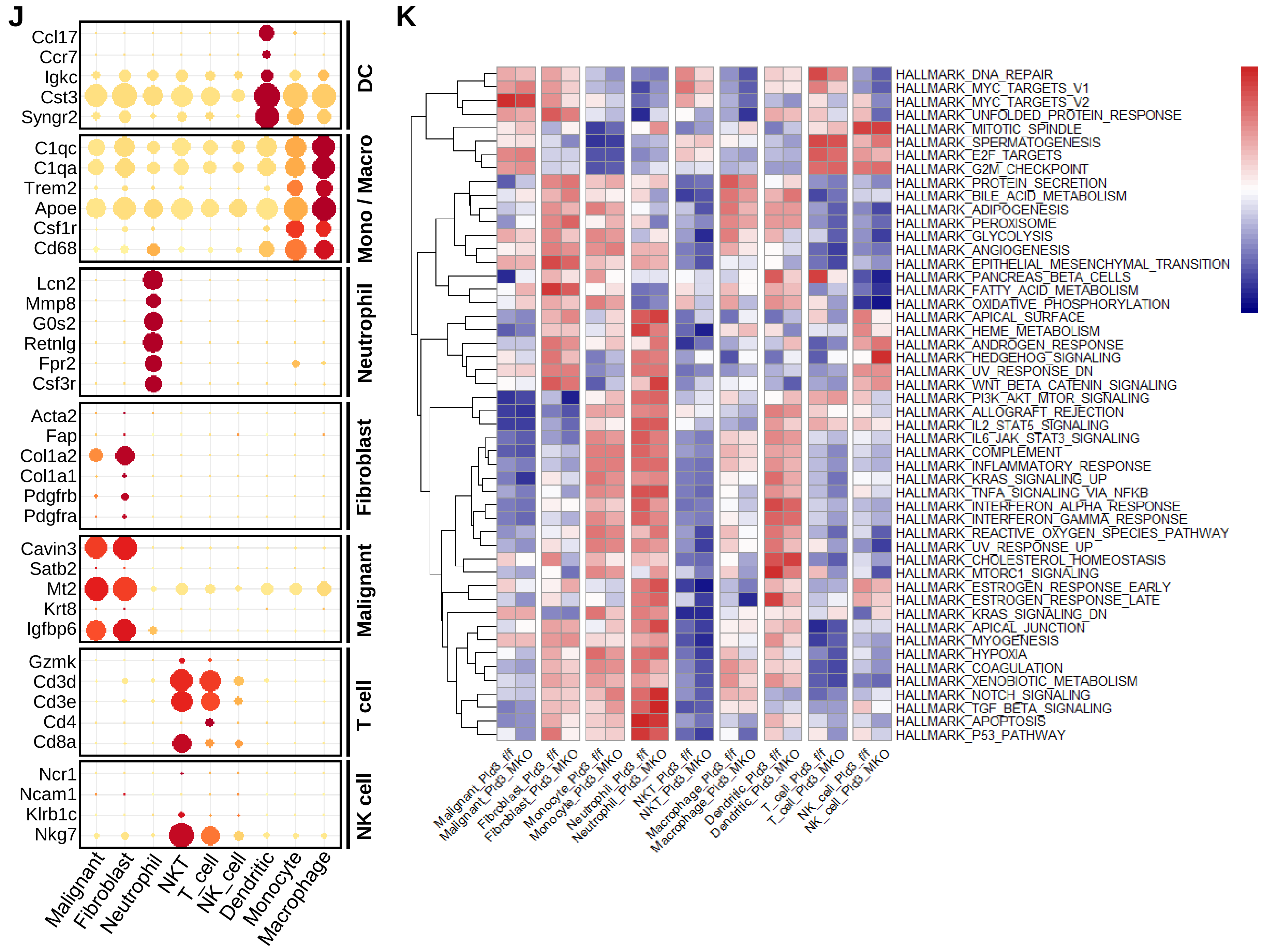

Figure S6. Construction and functional analysis of RAW264.7 stable Pld3 knockout cell line

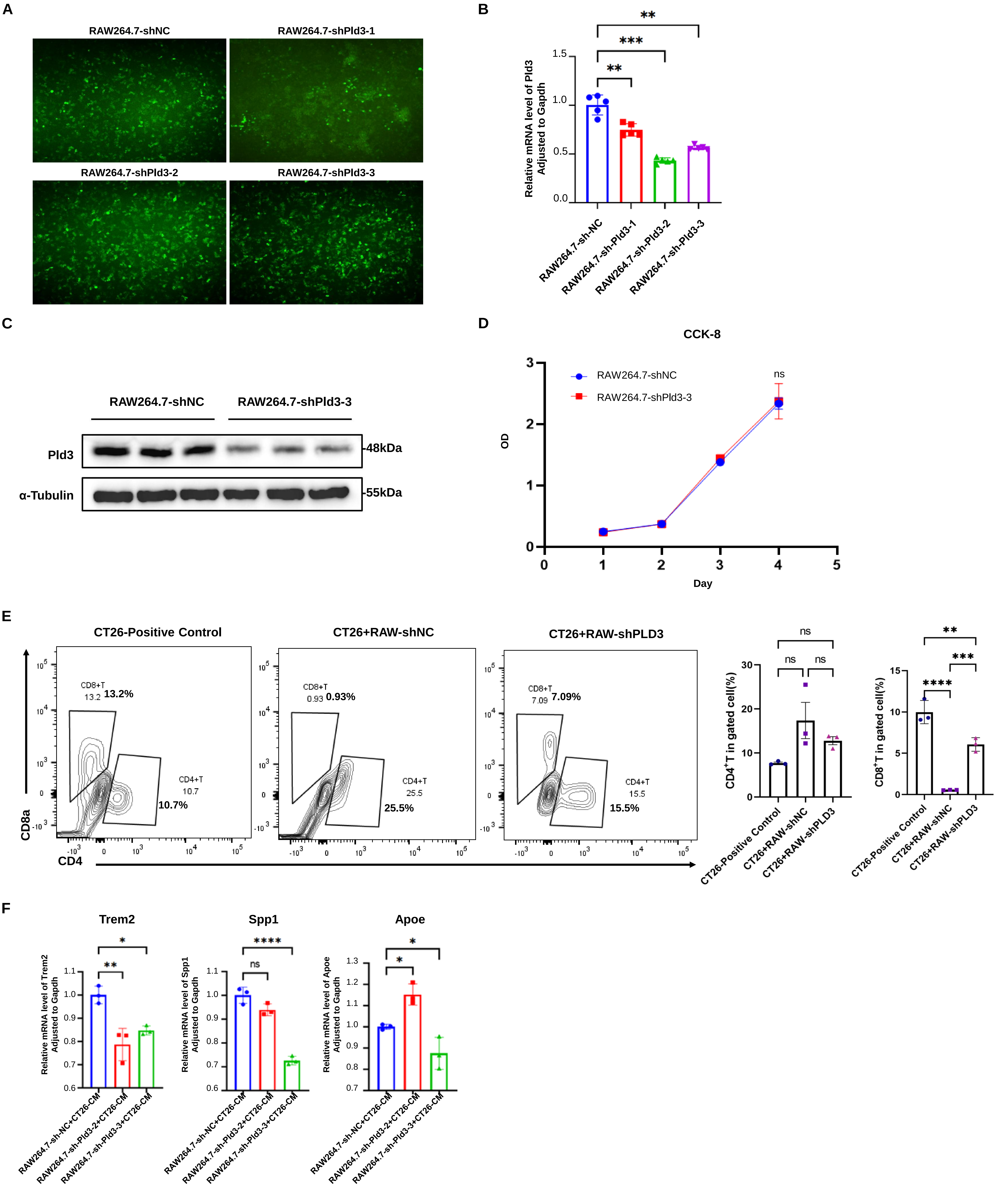

Figure S7. PLD3 is selectively expressed in macrophages but not neutrophils

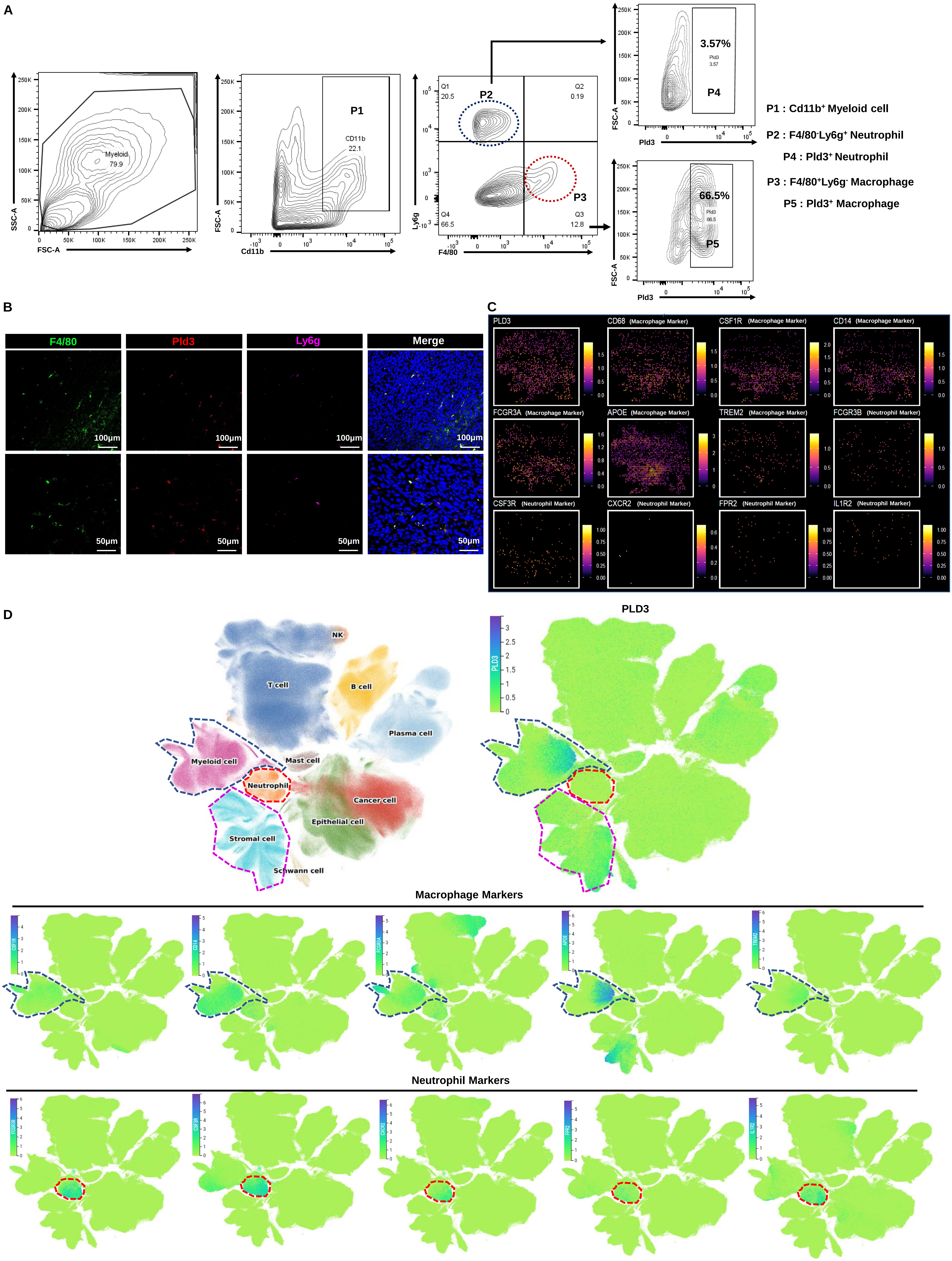

Figure S8. Single-cell validation reveals PLD3 is selectively expressed in macrophages but not neutrophils

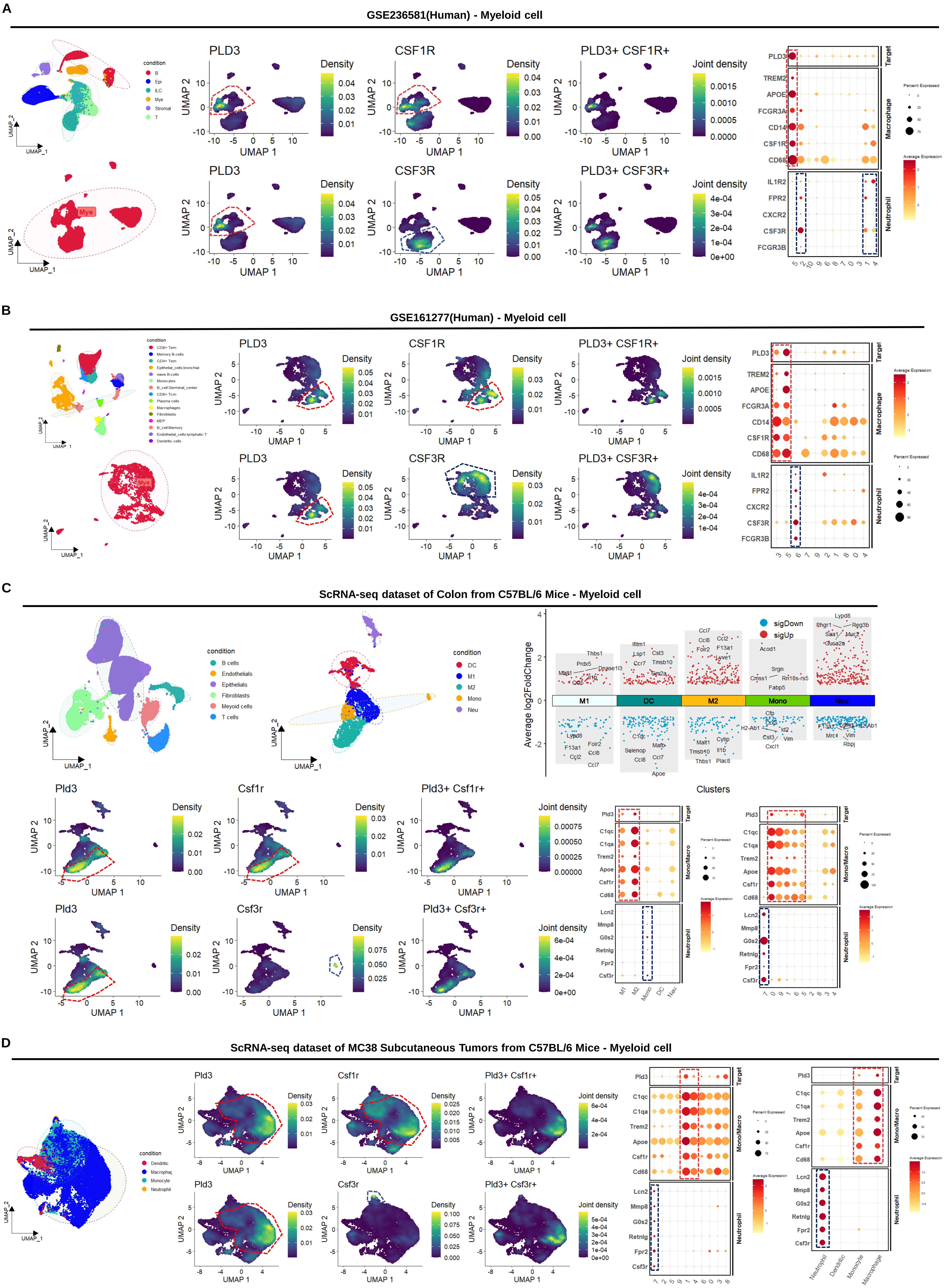

Figure S9. The gating strategy for flow cytometry data

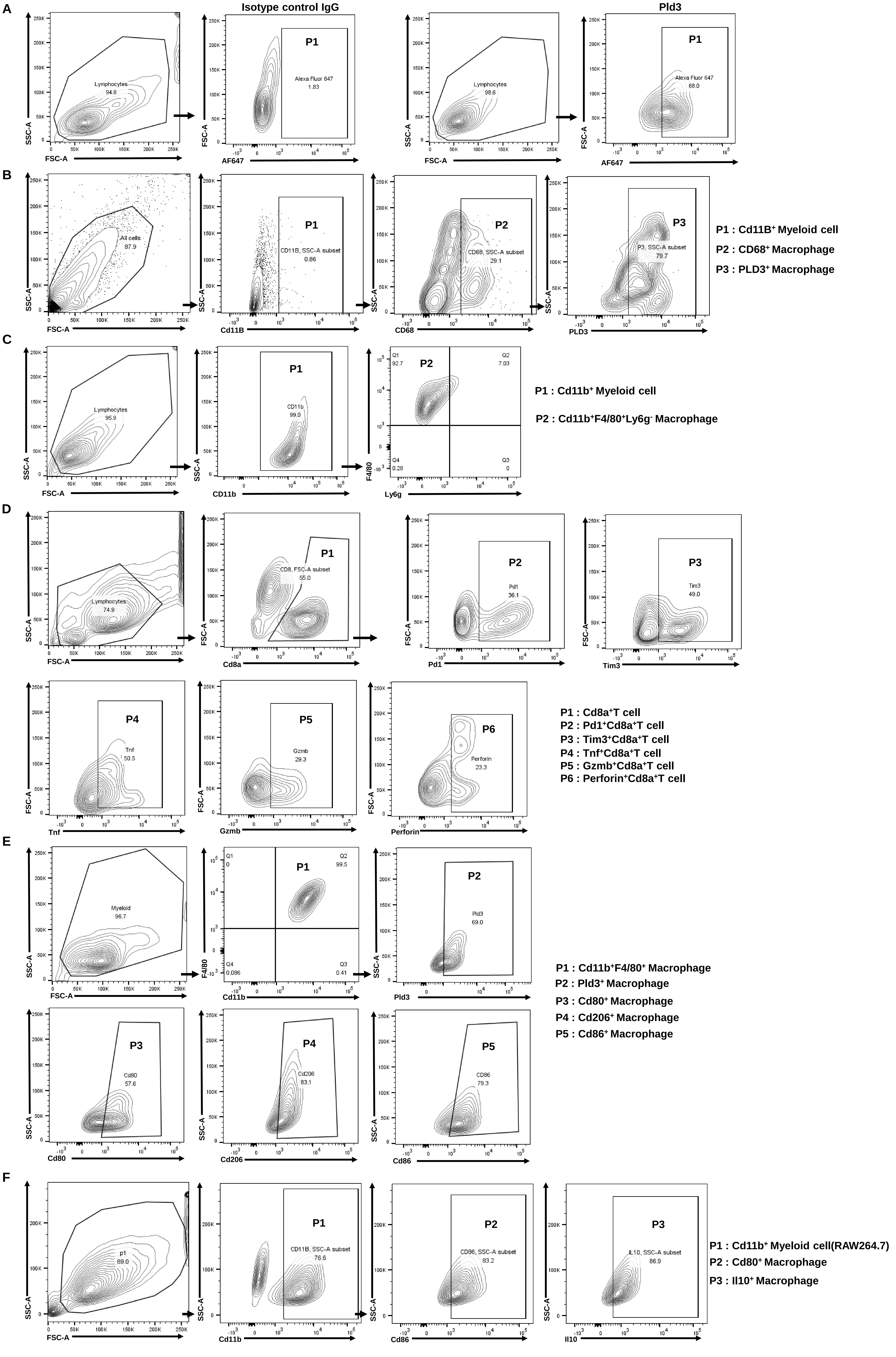

Figure S10. The gating strategy for flow cytometry data

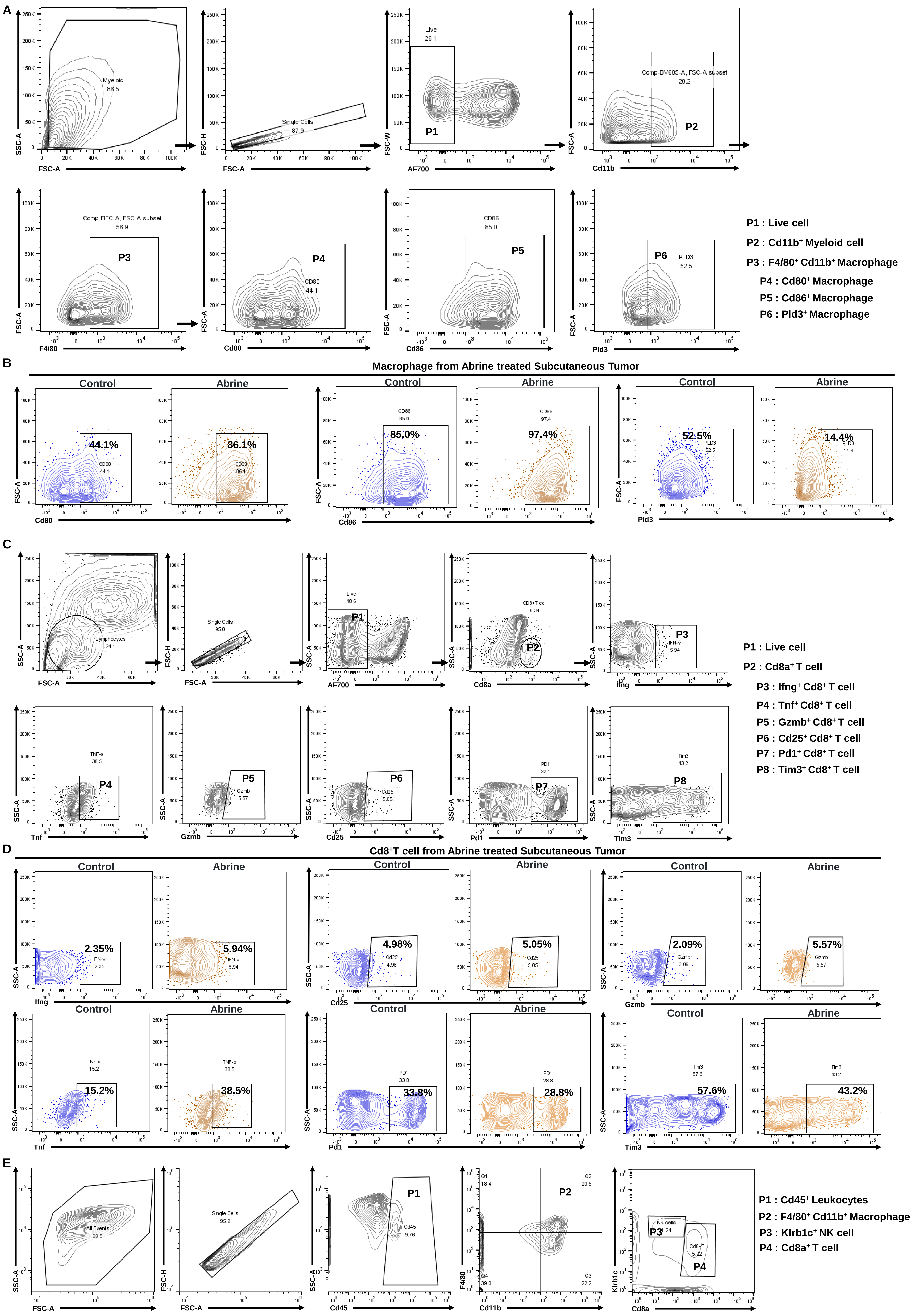

Figure S11. Pld3-deficient macrophage restores T cell-mediated antitumor immunity

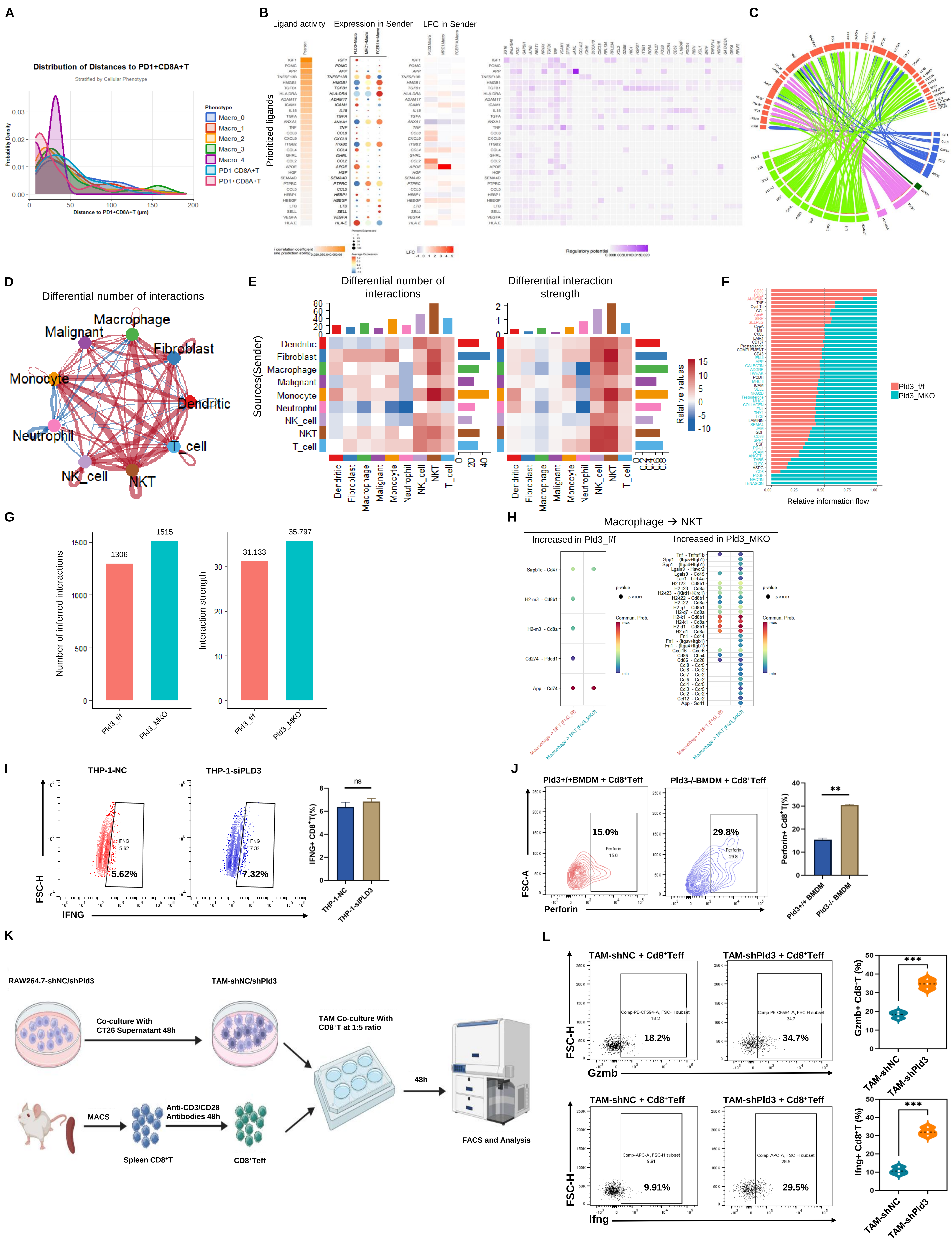

Figure S12. Pld3-deficient macrophage reduces the anti-inflammatory phenotype of tumor-associated macrophages

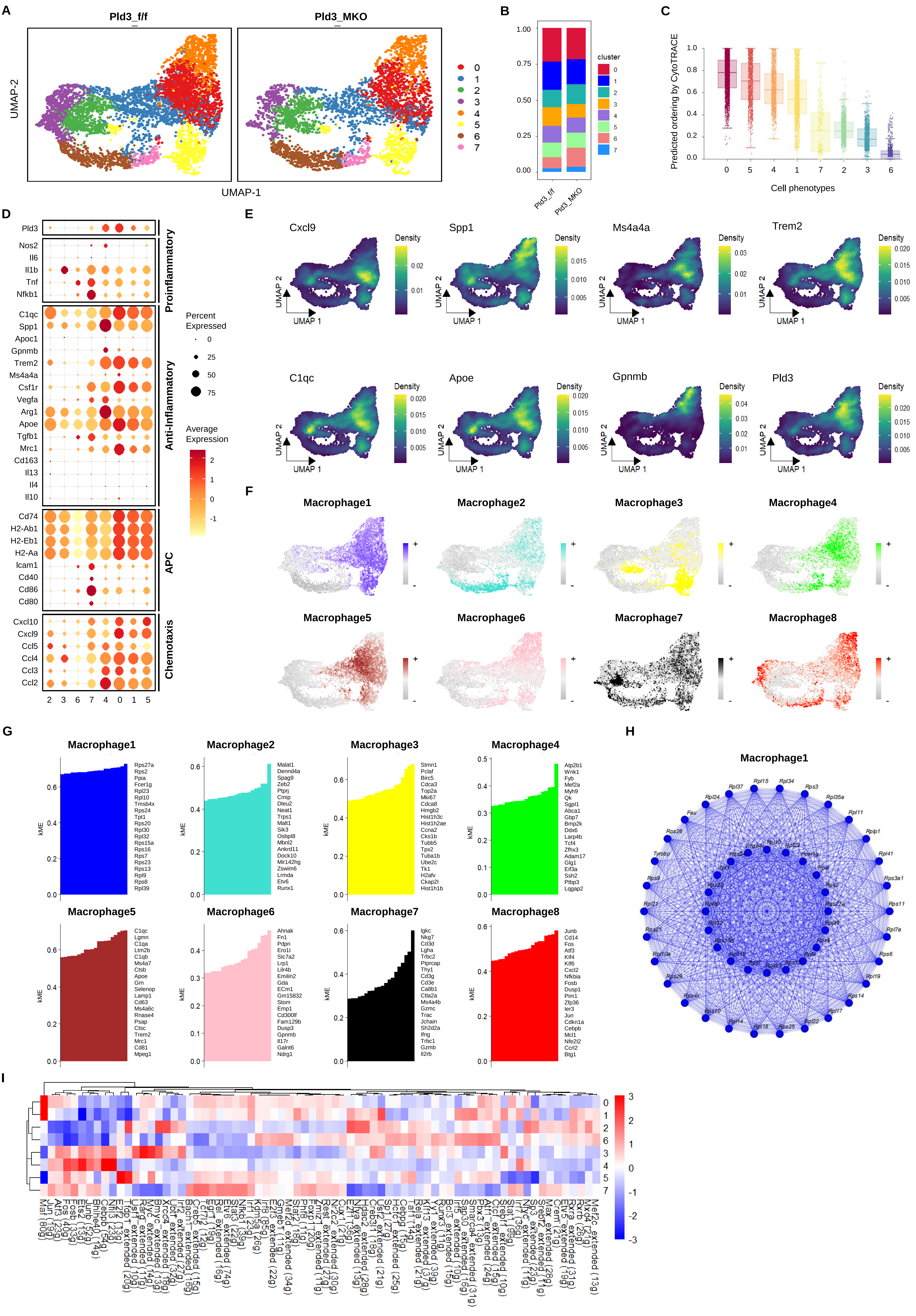

Figure S13. PLD3 interacts with TAMs markers to drive immunosuppressive macrophage polarization

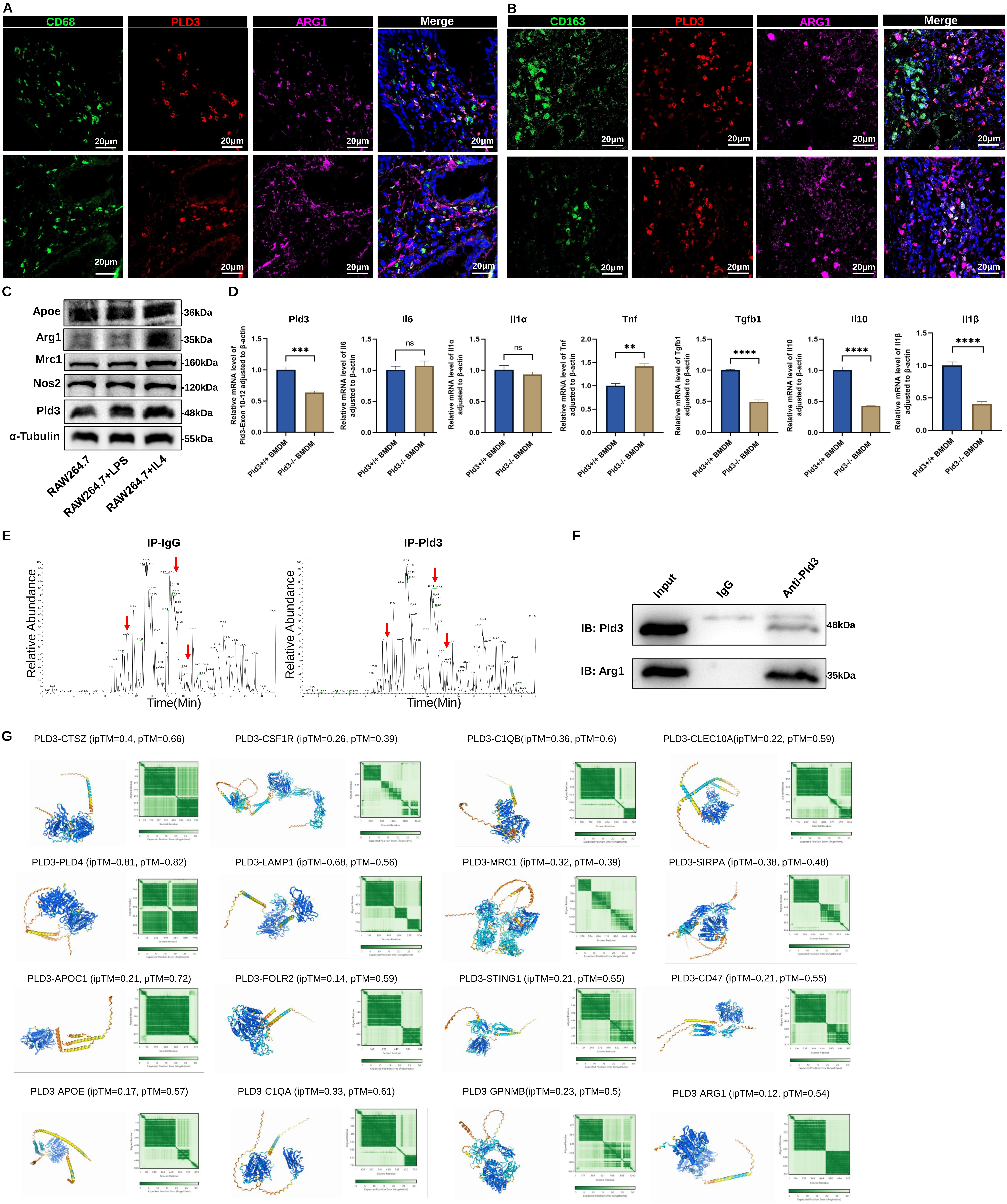

Figure S14. PLD3 drives cellular senescence, exerts pro-tumorigenic immunosuppressive capacity by orchestrating the lysosomal-AKT-NF-κB signaling axis

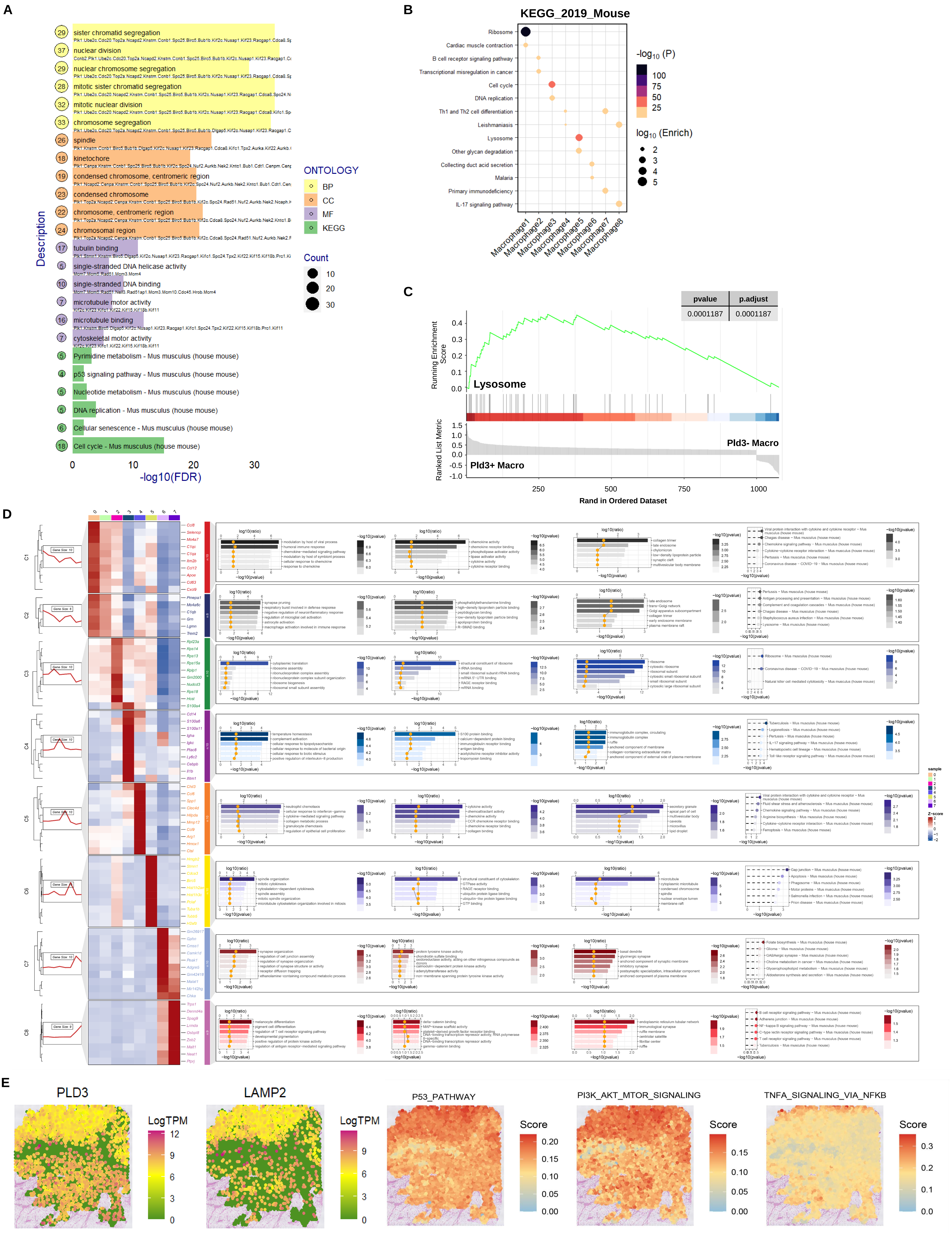

Figure S15. Localization of Pld3 in macrophages and its relationship with Lamp2, and effect of Pld3 on senescent macrophages

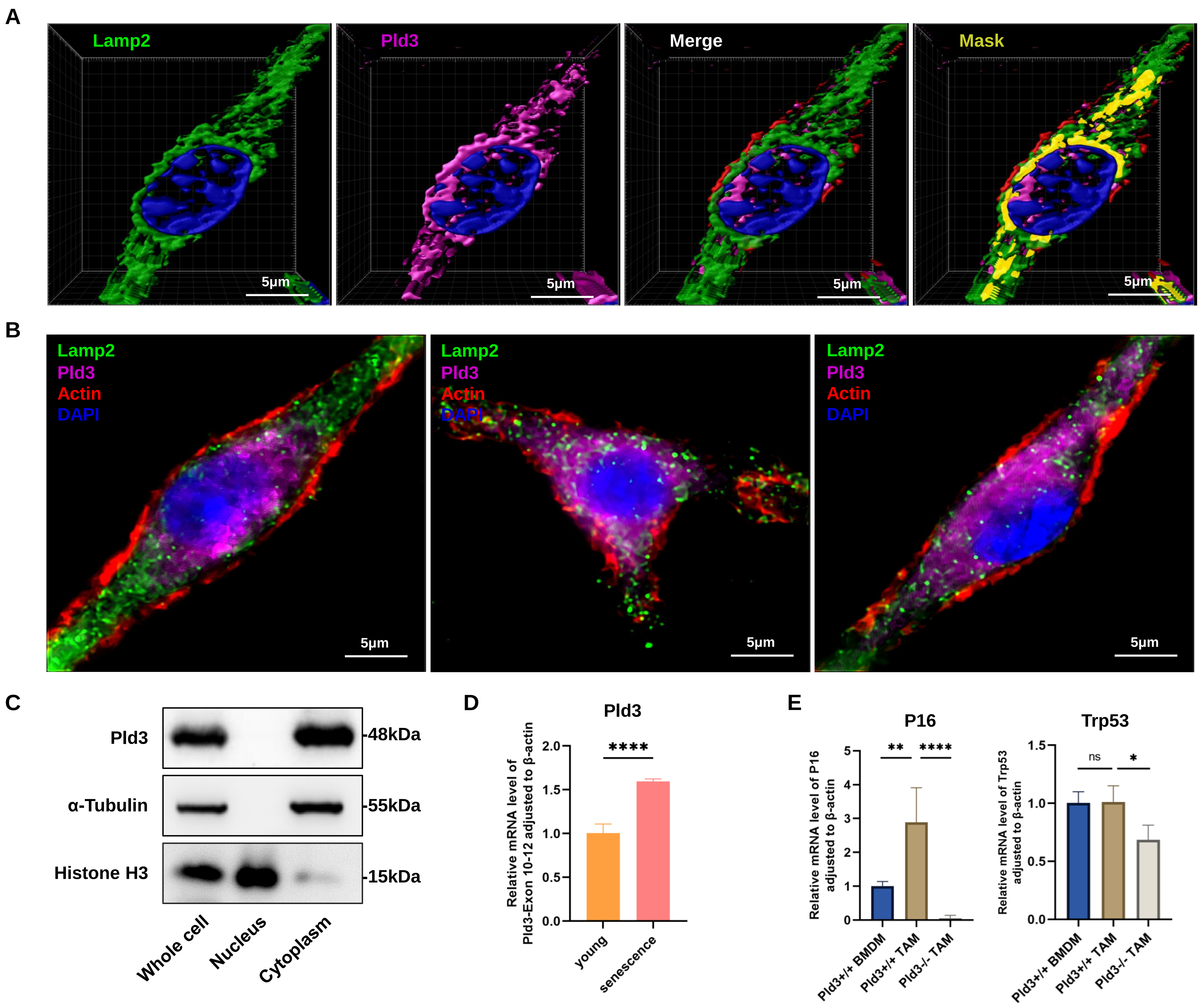

Figure S16. Abrine enhances anti-tumor responses by targeting PLD3 in tumor-associated macrophages

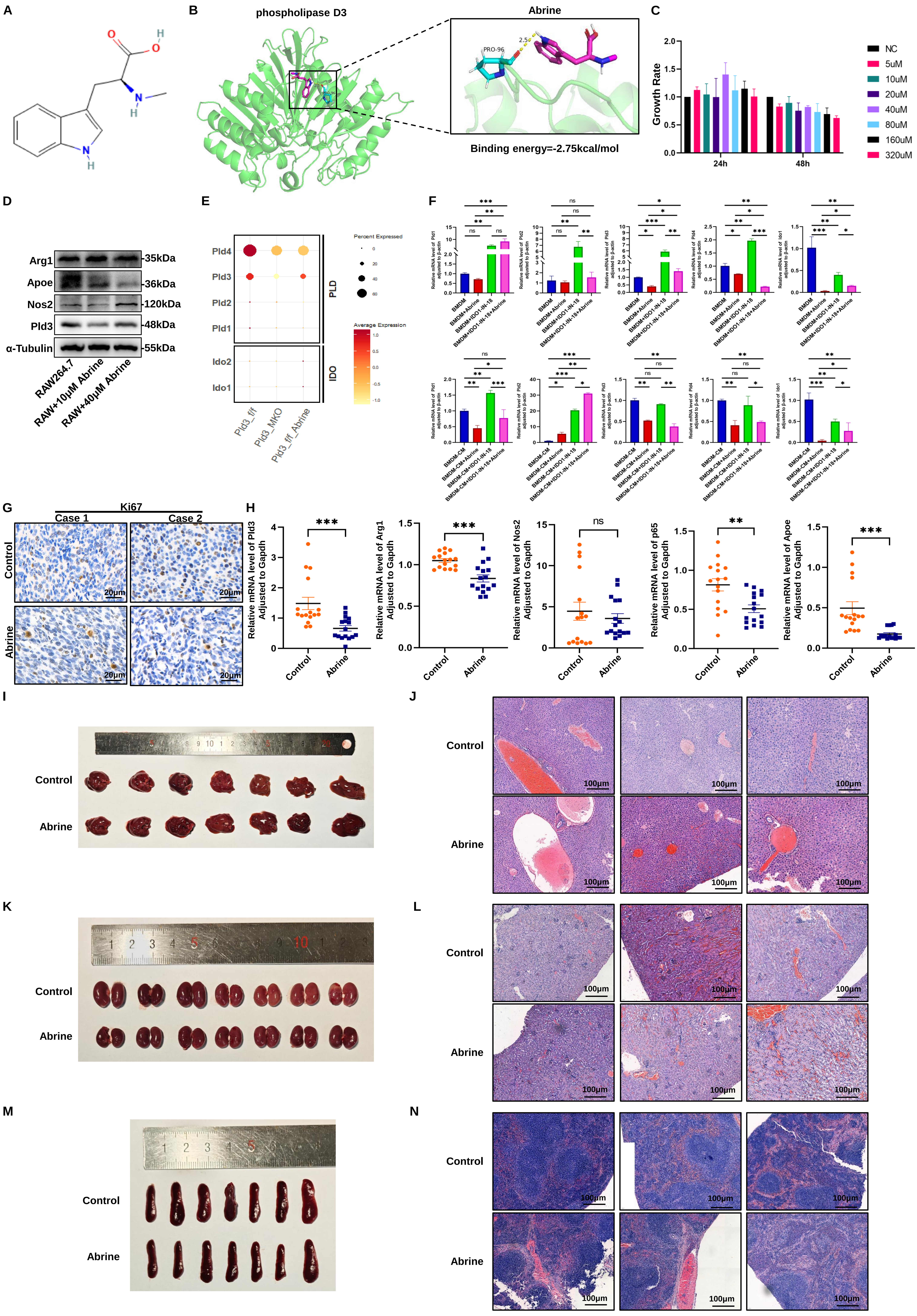

Figure S17. Abrine-mediated modulation of immune cells in the tumor microenvironment

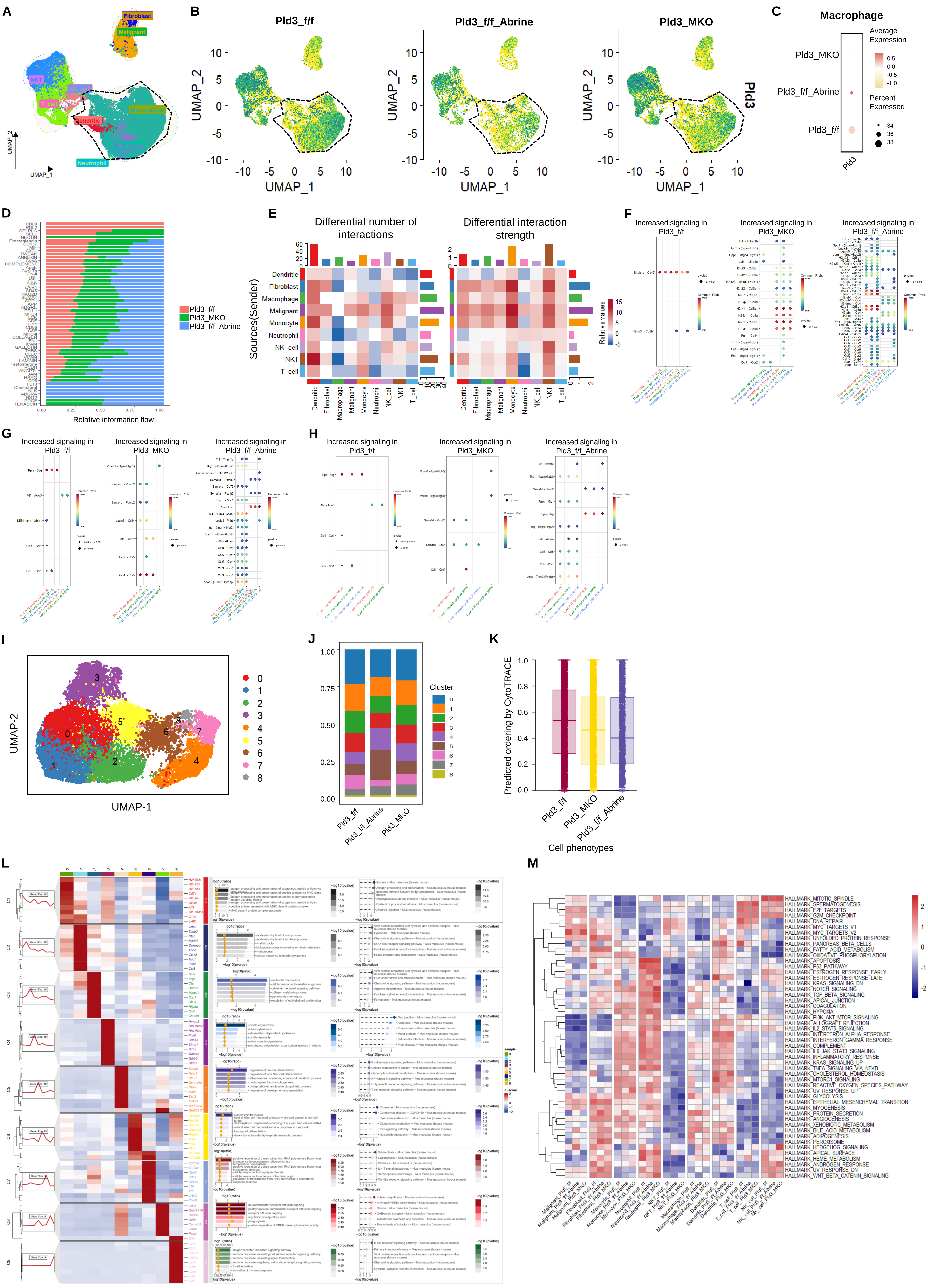

Figure S18. Targeting PLD3 improves the therapeutic benefit of anti-PD-1 immunotherapy to enhance anti-tumor efficacy

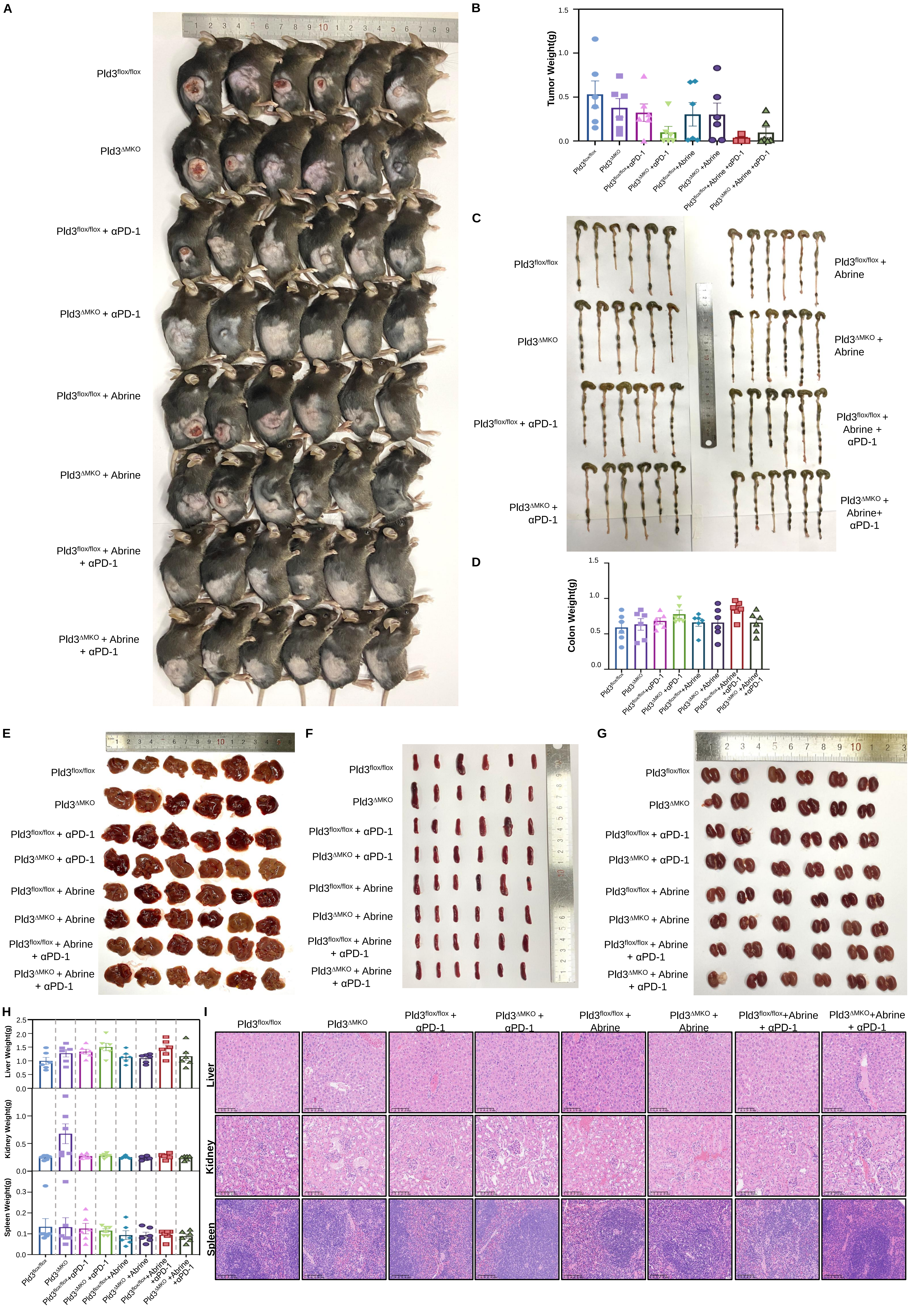

Figure S19. Abrine and PD-1 inhibitors promote CD8^{+} T cell infiltration and suppress tumor proliferation.

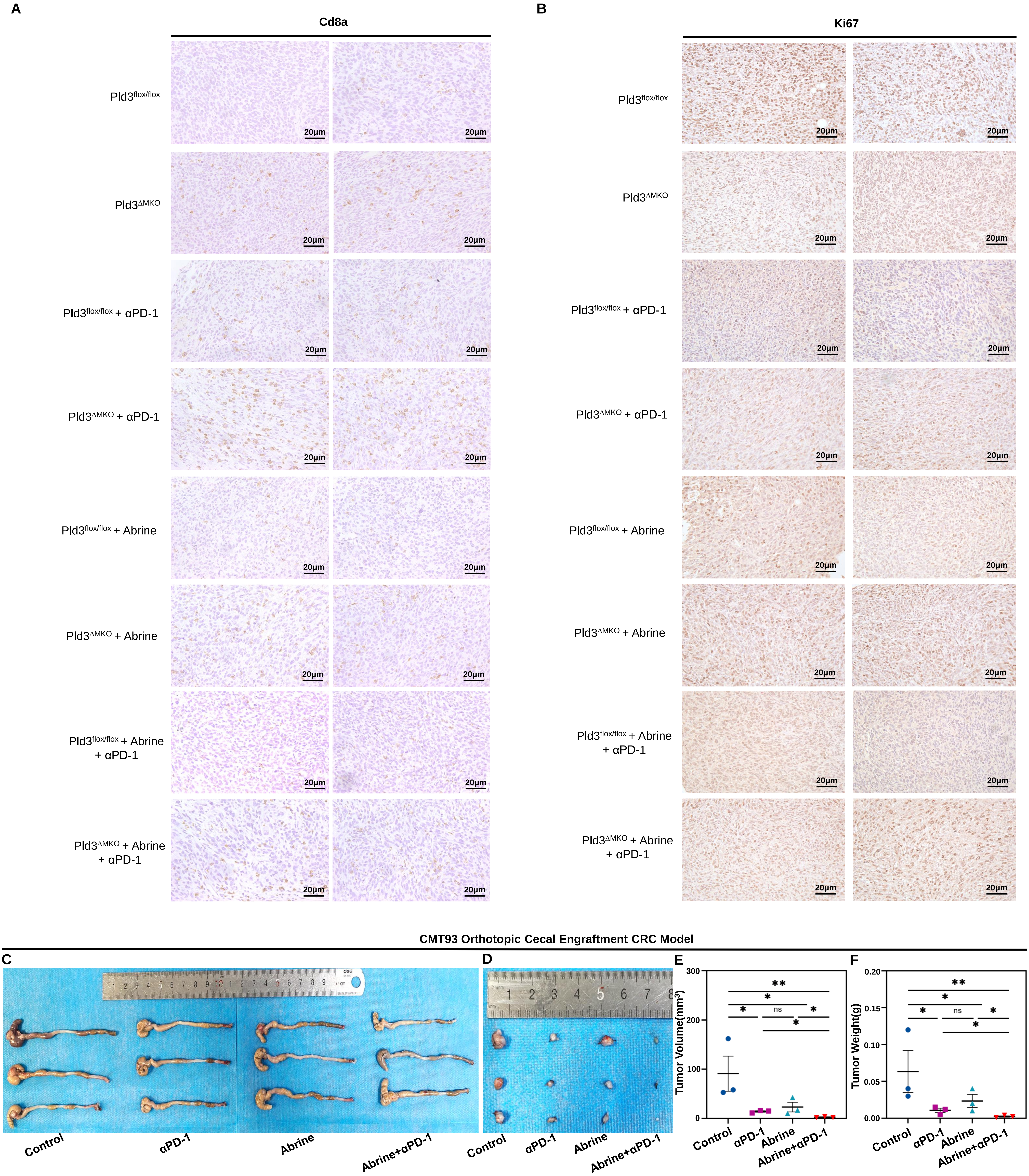

Supplement: Supplementary file 4 — Supporting File 4: advs75730‐sup‐0004‐FigureS1–S19.pdf. [file ADVS-9999-e75730-s003.pdf]
